# Global burden and trends of childhood non-Hodgkin lymphoma from 1990 to 2021

**DOI:** 10.3389/fped.2025.1618810

**Published:** 2025-06-26

**Authors:** Jing Xie, Jian-She Xu, Jiang-Wei Huang, Yi-Shan Zheng

**Affiliations:** ^1^ Department of Intensive Care Unit, The Second Hospital of Nanjing, Affiliated to Nanjing University of Chinese Medicine, Nanjing, China; ^2^ School of Public Health, Nanjing Medical University, Nanjing, China

**Keywords:** childhood non-Hodgkin lymphoma, incidence, mortality, health disparities, disability-adjusted life years (DALYs)

## Abstract

**Background:**

Childhood non-Hodgkin lymphoma (NHL) is a significant contributor to pediatric cancer morbidity and mortality worldwide. This study aims to assess the global, regional, and national burden of childhood NHL from 1990 to 2021, using data from the Global Burden of Disease (GBD) 2021 study.

**Methods:**

Incidence, mortality, and disability-adjusted life years (DALYs) related to childhood NHL were analyzed across 204 countries and 21 geographic regions using standardized methods from the GBD 2021 study. Estimated annual percentage change (EAPC) and average annual percentage change (AAPC) were calculated to evaluate long-term trends. Frontier and inequality analyses were applied to assess the relationship between sociodemographic development and disease burden.

**Results:**

In 2021, there were 20,788.76 (95% uncertainty intervals, UI: 17,199.49–25,305.83) new childhood NHL cases globally, with an incidence rate of 1.03 per 100,000 (95% UI: 0.85–1.26). The global mortality rate decreased from 0.75 (95% UI: 0.57–0.89) in 1990 to 0.45 (95% UI: 0.36–0.56) in 2021 (EAPC: −1.49, 95% confidence interval, CI: −1.60 to −1.39). DALYs rates also declined from 63.38 (95% UI: 48.09–75.72) in 1990 to 37.83 (95% UI: 29.95–47.17) in 2021 (EAPC: −1.51, 95% CI: −1.61 to −1.40). Burden reductions were most significant in high-SDI regions. In contrast, several low- and middle-SDI regions, particularly in Sub-Saharan Africa, experienced either slow declines or rising burden, with Southern Sub-Saharan Africa showing the largest increase in DALYs rate.

**Conclusions:**

While advancements in treatment and healthcare infrastructure have contributed to declining childhood NHL mortality, persistent disparities highlight the need for targeted interventions in low-SDI regions. Strengthening pediatric oncology capacity, expanding treatment accessibility, and enhancing early detection efforts are crucial for reducing global inequalities in childhood NHL outcomes.

## Introduction

Non-Hodgkin lymphoma (NHL) ranks among the most prevalent non-solid tumors in children worldwide, representing a substantial proportion of pediatric cancer incidence and mortality (1–3). According to the latest Global Burden of Disease (GBD) 2021 study, NHL accounts for 11% of all childhood malignancies and 11% of pediatric cancer-related deaths (2). Childhood NHL constitutes a heterogeneous spectrum of highly aggressive lymphoid malignancies, characterized by distinct biological, molecular, and clinical features (4). The primary subtypes of childhood NHL include Burkitt lymphoma (BL) (4), diffuse large B-cell lymphoma (DLBCL) (5), and anaplastic large cell lymphoma (ALCL) (6), each differing in cellular origin, molecular signatures, and clinical course, necessitating tailored therapeutic strategies.

Childhood NHL is a highly aggressive malignancy, often presenting with nonspecific early symptoms that hinder timely detection and contribute to delayed diagnosis in a significant proportion of cases (3, 7). Its clinical manifestations are diverse, with painless lymphadenopathy, fever, night sweats, and unexplained weight loss being the most frequently observed symptoms. Unlike solid tumors, NHL and other non-solid malignancies often exhibit widespread systemic involvement, including bone marrow infiltration and central nervous system (CNS) dissemination, which further complicates treatment strategies (7). Beyond the acute phase, childhood NHL imposes substantial long-term health consequences on survivors. Many experience persistent late effects, including secondary malignancies, chronic organ toxicity (e.g., chemotherapy-induced cardiotoxicity), endocrine dysfunction, and neurocognitive impairments, which may persist into adulthood, impacting quality of life and long-term health outcomes (3, 4, 8). NHL places significant emotional and economic burdens not only on affected children but also on their families and the broader healthcare system.

This study aims to provide a comprehensive assessment of the global burden and temporal trends of childhood NHL from 1990 to 2021. While NHL is one of the most common pediatric cancers worldwide, it remains a major public health concern due to its aggressive nature and stark survival disparities across countries. These inequalities are further compounded by the lack of high-quality data in many settings, making the true burden difficult to quantify and interpret. Although previous studies have explored the epidemiology of childhood NHL, most are limited to single countries, narrow time periods, or small sample sizes. Few have systematically examined long-term incidence and burden trends across multiple countries or regions, particularly using harmonized and comparable metrics. The lack of multicountry comparative analyses constrains our understanding of geographic disparities and impedes the development of globally informed health policies. By leveraging the GBD 2021 dataset, this study seeks to identify patterns in burdens, assess global inequalities, and quantify performance gaps in burden reduction relative to national development levels. This disease-specific analysis aims to bridge the evidence gap and provide meaningful insights to guide future research and cancer control strategies, particularly in under-resourced settings.

## Methods

### Data sources and overview

The data for this study were obtained from the GBD 2021 study, conducted by the Institute for Health Metrics and Evaluation (IHME) at the University of Washington. The GBD study compiles information from multiple sources including population-based cancer registries, hospital-based registries, vital registration systems, and survey data. Where primary data are not available or incomplete, GBD applies standardized modeling techniques such as CODEm (Cause of Death Ensemble Modeling) and DisMod-MR 2.1 to estimate incidence, mortality, and DALYs. Disability weights (DWs) used for NHL were derived from the GBD 2019 disability weight measurement study, which elicited health state valuations through large-scale population surveys. The same DWs are applied uniformly across all countries to ensure comparability, although this may not fully reflect cultural or regional variation in disease perception. For this analysis, we extracted key epidemiological indicators for childhood NHL, specifically incidence, mortality, and DALYs. These indicators were stratified by gender, age groups (under 1 year, 1–2 years, 2–4 years, 5–9 years, and 10–14 years), and geographic location for the period 1990–2021. The GBD dataset used in this study was accessed and downloaded on December 27, 2024. In this study, the term “regional” refers to the 21 geographic regions as defined by the GBD Study, which correspond to supranational groupings such as UN subregions (e.g., Eastern Sub-Saharan Africa, Western Europe).

### Sociodemographic index

The Sociodemographic Index (SDI) is a composite indicator employed in the GBD study to quantify the socioeconomic development of countries and regions. It is computed based on three core determinants: income per capita, mean years of schooling for individuals aged 15 and older, and the fertility rate among women under 25 years (9). The SDI scale ranges from 0 to 1, with higher values signifying greater socioeconomic development. Under the GBD classification, countries are grouped into five SDI tiers: low, low-middle, middle, high-middle, and high. As a standardized measure, SDI enables a systematic evaluation of the relationship between socioeconomic status and disease burden, allowing for cross-national comparisons and a more granular understanding of global health disparities (10). In this study, SDI was leveraged to assess regional variations in the incidence, mortality, and DALY rates of childhood NHL, offering insights into how socioeconomic determinants shape the burden of pediatric NHL across different geographic contexts.

### Cross-country health inequality analysis

To evaluate both absolute and relative inequalities in the burden of childhood NHL across countries, we employed the slope index and the concentration index (11–15). The slope index quantifies absolute inequality by ranking populations based on weighted SDI values, ranging from the lowest to the highest subgroup. A regression analysis is then performed between the burden indicators and the midpoint of each SDI subgroup. The slope index value is calculated as the difference between the predicted burden in the highest and lowest SDI regions, where a positive slope index indicates a greater burden in high-SDI regions, while a negative slope index suggests a higher burden in low-SDI regions (13).

In contrast, the concentration index assesses relative inequality by plotting the cumulative disease burden against the ranked SDI distribution. The concentration curve is generated by connecting these cumulative data points, while the line of equality represents a perfectly equitable distribution. If the concentration curve lies above the equality line, it signifies that the disease burden is disproportionately concentrated in low-SDI regions. Conversely, when the curve falls below the equality line, it indicates that the burden is skewed toward high-SDI regions. By integrating these indices, our analysis provides a systematic and quantitative assessment of how socioeconomic disparities shape the global distribution of childhood NHL burden, offering valuable insights for public health policy and resource allocation (13).

### Frontier analysis

We applied frontier analysis to develop a frontier model for incidence, mortality, and DALYs rates, using the SDI as a reference variable. Unlike traditional regression models, which predominantly establish linear relationships or generate predictive estimates, frontier analysis accounts for the inherent non-linearity between SDI and disease burden (16). This approach enables the identification of the lowest theoretically attainable NHL burden in relation to a country's developmental status, as indicated by SDI. We constructed efficiency frontiers using stochastic frontier modeling, with SDI as the independent variable and crude rates of incidence, mortality, or DALYs as dependent variables. The model estimates the minimum achievable burden at each level of SDI, assuming a production frontier of optimal performance. The distance from each country's observed burden to the frontier reflects the effective performance gap.

The frontier curve serves as a benchmark, delineating the performance threshold set by countries or territories that achieve the most favorable NHL outcomes relative to their SDI level—those effectively pushing the limits of attainable health outcomes. The distance from this frontier, termed the “effective difference,” quantifies the discrepancy between a country's actual NHL burden and its minimal theoretically achievable burden at the same SDI level. A larger effective difference signals untapped potential for further reductions in NHL burden, contingent upon available sociodemographic resources. To enhance the robustness of our estimates, we conducted 1,000 bootstrap simulations and computed the mean NHL burden rate at each SDI level, ensuring statistical reliability in the derived frontier estimates. Although incidence and mortality rates are ultimately derived from count data and may follow Poisson or other non-normal distributions, our application of frontier analysis treats age-standardized rates as continuous variables, which is consistent with previous global burden and benchmarking studies. This method is not used here for inferential statistical modeling, but rather as a descriptive tool to assess potential performance gaps across countries.

### Statistical analysis

The incidence, mortality, and DALYs cases and rates served as the primary epidemiological indicators for assessing the burden of childhood NHL. All rates were expressed per 100,000 population and were reported with 95% uncertainty intervals (UI), derived from the 25th and 975th ordered values obtained from 1,000 posterior distribution draws, ensuring statistical robustness and reliability (1, 17, 18).

To evaluate temporal trends, we employed the Joinpoint regression model to compute the Annual Percent Change (APC) and its corresponding 95% confidence interval (CI) (19). The APC metric was utilized to assess trends in incidence, mortality, and DALY rates of childhood NHL, where a positive APC value denotes an increasing trend, while a negative APC value signifies a declining trend in the respective indicator. To provide a comprehensive assessment of long-term trends, we also calculated the Average Annual Percentage Change (AAPC), which represents the overall rate of change across a specified period by averaging annual percentage fluctuations (20). Unlike APC, which measures interannual variability, AAPC smooths short-term fluctuations and offers a more stable reflection of cumulative trends over time.

We applied the Estimated Annual Percentage Change (EAPC) to quantify the average annual rate of change over the study period (21). This was achieved using a linear regression model fitted to the natural logarithm of the rates, with the estimated slope coefficient representing the EAPC. Statistical significance was determined based on 95% CI interpretation, where a lower bound greater than 0 indicated a statistically significant increasing trend, an upper bound below 0 suggested a significant declining trend, and a CI containing 0 implied a non-significant temporal change. To estimate the Estimated Annual Percentage Change (EAPC), we fitted a log-linear regression model to the rate for each year: ln(Rate) = α + β  ×  year + ε, where Rate refers to the crude incidence, mortality, or DALYs rate among children aged 0–14 years, and year is the calendar year (treated as a continuous variable). The EAPC was then calculated as: EAPC = 100 × (exp^β^-1).

All statistical analyses and data visualizations were performed using R software (version 4.4.2). A two-sided *p*-value <0.05 was considered to indicate statistical significance.

## Results

### Global burden of non-Hodgkin lymphoma in children

#### Incidence

**Figure 1:**
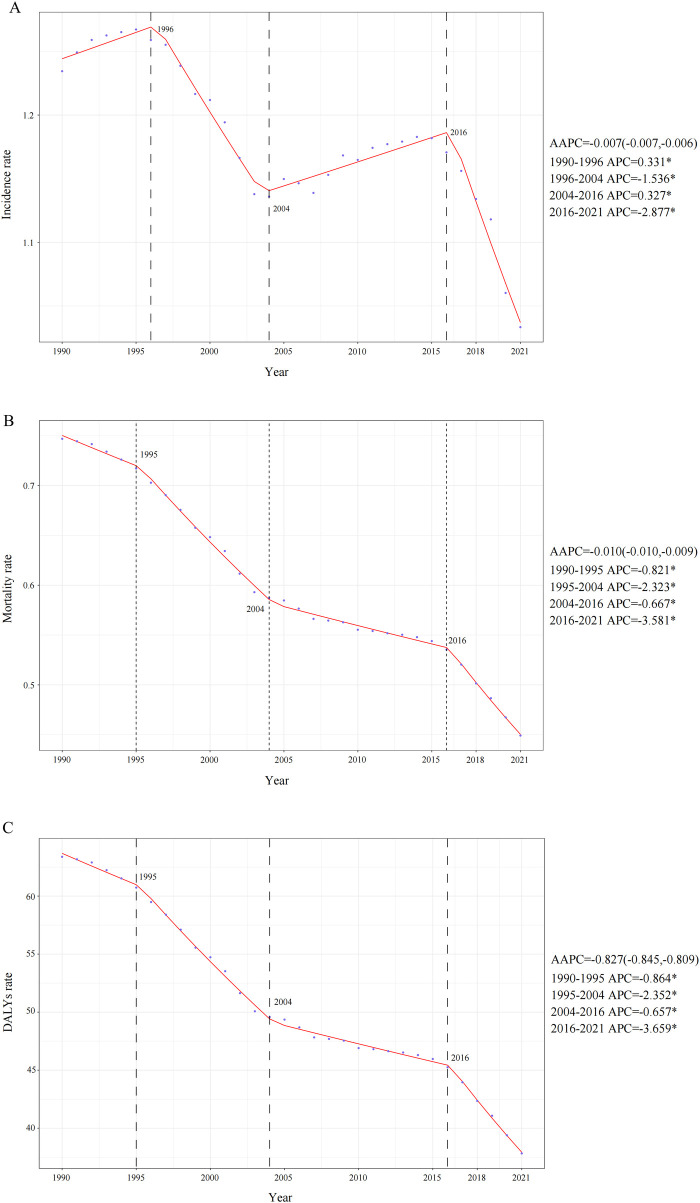
Annual percent change (APC), average annual percentage change (AAPC)and trends in global childhood non-hodgkin lymphoma incidence, mortality, and disability-adjusted life years (DALYs) from 1990 to 2021. **(A)** Incidence rate. **(B)** Mortality rate. **(C)** DALYs rate.

In 2021, a total of 20,788.76 (95% UI: 17,199.49–25,305.83) childhood NHL cases were reported globally, marking a 3.17% decrease (95% UI: −20.21%–37.22%) compared to 1990 (Table 1). Additionally, the incidence rate declined from 1.23 (95% UI: 1.00–1.44) in 1990 to 1.03 (95% UI: 0.85–1.26) in 2021, with an EAPC of −0.43 (95% CI: −0.55 to −0.32) and an AAPC of −0.007 (95% CI: −0.007 to −0.006) (Table 1 and Figure 1A). Notably, the most pronounced decline occurred between 2016 and 2021, with an APC of −2.877 (95% CI: −3.328 to −2.424) (Figure 1A).

**Table 1:** Incidence of Non-hodgkin lymphoma in children between 1990 and 2021 at the global and regional level.

Location	Number (95% UI)		Percentage change (95% UI)	Rate (95% UI)		EAPC (95% CI)
	1990	2021		1990	2021	
Global	21,469.72 (17,405.39, 24,965.96)	20,788.76 (17,199.49, 25,305.83)	−3.17 (−20.21, 37.22)	1.23 (1.00, 1.44)	1.03 (0.85, 1.26)	−0.43 (−0.55,−0.32)
Low SDI	4,377.35 (2,848.30, 5,768.16)	5,932.40 (3,988.74, 8,047.35)	35.52 (−0.41, 152.34)	1.91 (1.24, 2.52)	1.29 (0.87, 1.75)	−1.16 (−1.28,−1.04)
Low-middle SDI	4,970.40 (3,345.75, 6,266.44)	5,277.19 (4,217.08, 6,668.60)	6.17 (−21.64, 74.46)	1.05 (0.71, 1.33)	0.91 (0.73, 1.15)	−0.34 (−0.43,−0.24)
Middle SDI	5,677.01 (4,774.10, 6,530.52)	4,799.77 (3,948.21, 5,966.06)	−15.45 (−31.64, 15.17)	0.98 (0.83, 1.13)	0.85 (0.70, 1.05)	−0.26 (−0.44,−0.08)
High-middle SDI	3,779.54 (3,389.04, 4,173.63)	2,510.88 (2,191.23, 2,927.62)	−33.57 (−42.99, −18.64)	1.38 (1.24, 1.53)	1.09 (0.95, 1.27)	−0.53 (−0.72,−0.34)
High SDI	2,645.39 (2,521.37, 2,767.43)	2,249.56 (2,041.06, 2,443.45)	−14.96 (−22.04, −6.65)	1.42 (1.36, 1.49)	1.30 (1.18, 1.42)	−0.31 (−0.55,−0.08)
East Asia	3,701.78 (3,060.56, 4,553.18)	2,028.44 (1,606.73, 2,618.11)	−45.20 (−57.99, −22.60)	1.12 (0.93, 1.38)	0.76 (0.60, 0.98)	−1.15 (−1.56,−0.74)
Southeast Asia	1,181.40 (780.70, 1,556.84)	1,071.56 (867.43, 1,427.84)	−9.30 (−28.01, 37.79)	0.69 (0.46, 0.91)	0.62 (0.50, 0.83)	−0.35 (−0.43,−0.27)
Oceania	23.78 (15.62, 34.43)	63.97 (39.80, 94.99)	169.05 (73.86, 299.76)	0.89 (0.58, 1.28)	1.26 (0.78, 1.87)	1.14 (0.91,1.37)
Central Asia	324.46 (265.47, 382.95)	298.46 (242.89, 371.99)	−8.01 (−29.57, 19.49)	1.30 (1.06, 1.53)	1.08 (0.88, 1.34)	−0.60 (−0.83,−0.36)
Central Europe	321.12 (295.65, 347.57)	193.03 (169.75, 219.87)	−39.89 (−48.56, −28.32)	1.09 (1.00, 1.18)	1.09 (0.96, 1.24)	0.29 (−0.10,0.69)
Eastern Europe	1,100.28 (1,037.65, 1,171.54)	443.59 (405.55, 486.16)	−59.68 (−63.97, −54.49)	2.14 (2.02, 2.28)	1.25 (1.14, 1.37)	−1.66 (−2.37,−0.95)
High-income Asia Pacific	328.52 (285.55, 383.14)	236.81 (193.98, 286.82)	−27.92 (−43.77, −8.96)	0.93 (0.81, 1.09)	1.06 (0.86, 1.28)	0.36 (0.11,0.62)
Australasia	75.26 (63.65, 89.03)	67.16 (52.43, 84.09)	−10.76 (−32.84, 17.43)	1.64 (1.39, 1.94)	1.17 (0.91, 1.47)	−1.35 (−2.14,−0.54)
Western Europe	1,508.30 (1,381.87, 1,663.71)	1,421.96 (1,252.50, 1,601.73)	−5.72 (−19.06, 8.77)	2.12 (1.95, 2.34)	2.09 (1.84, 2.35)	−0.21 (−0.57,0.15)
Southern Latin America	127.32 (108.81, 149.47)	114.08 (92.04, 141.44)	−10.40 (−31.88, 18.63)	0.85 (0.73, 1.00)	0.79 (0.63, 0.98)	0.03 (−0.36,0.43)
High-income North America	972.61 (919.58, 1,029.72)	683.85 (605.47, 757.17)	−29.69 (−37.83, −20.84)	1.58 (1.49, 1.67)	1.04 (0.92, 1.15)	−1.43(−1.58,−1.27)
Caribbean	231.64 (161.79, 289.06)	183.94 (126.81, 247.11)	−20.59 (−40.23, 1.96)	2.03 (1.42, 2.53)	1.60 (1.10, 2.15)	−0.56 (−0.66,−0.45)
Andean Latin America	169.78 (141.72, 210.93)	218.50 (160.85, 287.45)	28.70 (−7.46, 78.97)	1.14 (0.95, 1.42)	1.21 (0.89, 1.59)	0.46 (0.25,0.66)
Central Latin America	566.05 (522.70, 615.90)	485.28 (406.15, 575.58)	−14.27 (−28.89, 2.95)	0.88 (0.81, 0.96)	0.76 (0.64, 0.91)	0.05 (−0.27,0.36)
Tropical Latin America	482.92 (428.99, 538.39)	292.47 (234.72, 348.22)	−39.44 (−51.08, −25.86)	0.90 (0.80, 1.00)	0.58 (0.47, 0.69)	−0.93 (−1.43,−0.43)
North Africa and Middle East	1,528.65 (1,142.55, 1,980.11)	1,848.35 (1,540.59, 2,309.64)	20.91 (−13.17, 73.84)	1.09 (0.81, 1.41)	1.01 (0.84, 1.26)	0.10 (−0.12,0.33)
South Asia	4,446.04 (2,790.87, 5,707.71)	4,514.77 (3,508.85, 5,872.59)	1.55 (−28.12, 90.16)	1.03 (0.64, 1.32)	0.89 (0.69, 1.16)	−0.34 (−0.49,−0.18)
Central Sub-Saharan Africa	227.93 (67.60, 349.57)	243.55 (158.69, 340.70)	6.85 (−24.16, 181.73)	0.90 (0.27, 1.38)	0.42 (0.27, 0.58)	−2.15 (−2.34,−1.96)
Eastern Sub-Saharan Africa	2,328.46 (1,629.73, 3,042.01)	2,916.18 (1,898.38, 4,221.69)	25.24 (−11.89, 125.44)	2.57 (1.80, 3.36)	1.63 (1.06, 2.37)	−1.28 (−1.43,−1.13)
Southern Sub-Saharan Africa	124.50 (97.76, 159.74)	249.55 (186.77, 317.54)	100.44 (47.37, 165.54)	0.60 (0.47, 0.77)	1.04 (0.78, 1.32)	2.25 (1.72,2.78)
Western Sub-Saharan Africa	1,698.91 (1,188.70, 2,245.72)	3,213.26 (1,906.57, 4,577.45)	89.14 (34.00, 201.52)	1.93 (1.35, 2.56)	1.50 (0.89, 2.13)	−0.66 (−0.80,−0.51)

EAPC, estimated annual percentage change; SDI, sociodemographic index; UI, uncertainty interval; CI, confidence interval.

**Figure 2:**
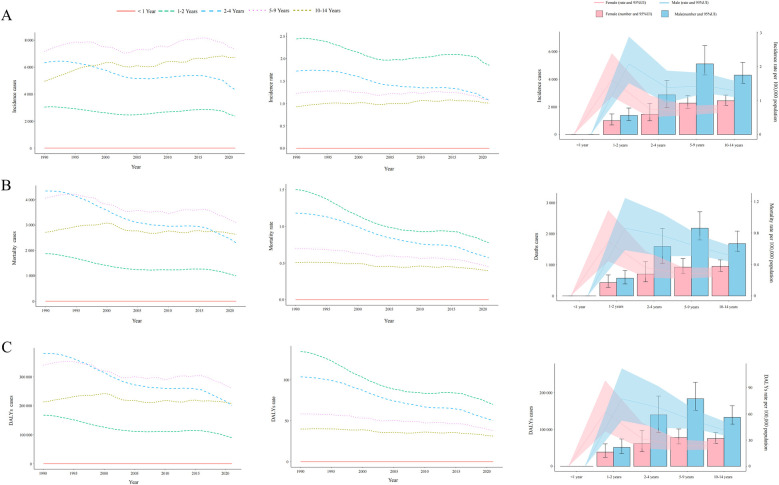
Trends in incidence, mortality, and disability-adjusted life years (DALYs) of childhood non-hodgkin lymphoma by age and sex, 1990–2021. **(A)** Incidence cases and rates. **(B)** Mortality cases and rates. **(C)** DALYs cases and rates.

Between 1990 and 2021, no consistent long-term upward or downward trend was observed in NHL incidence rates across different pediatric age groups (Figure 2A). Throughout this period, the highest number of incident cases was recorded in children aged 5–9 years, whereas no reported cases (both in absolute numbers and incidence rates per 100,000 population) were consistently documented among infants under 1 year of age. By 2021, the incidence burden remained higher in males than females across all pediatric age groups. Furthermore, despite the highest absolute number of cases occurring in the 5–9-year age group, the highest incidence rate per 100,000 population throughout 1990–2021 was consistently observed in children aged 1–2 years (Figure 2A).

#### Mortality

The global childhood NHL mortality burden exhibited a substantial decline, decreasing by 30.43% (95% UI: −43.66%–6.80%) from 12,990.00 deaths (95% UI: 9,904.43–15,509.02) in 1990 to 9,036.49 deaths (95% UI: 7,197.45–11,251.98) in 2021 (Supplementary Table S1). The mortality rate per 100,000 population also showed a consistent downward trend, decreasing from 0.75 (95% UI: 0.57–0.89) in 1990 to 0.45 (95% UI: 0.36–0.56) in 2021, with an EAPC of −1.49 (95% CI: −1.60 to −1.39). The overall decline was further supported by an AAPC of −0.010 (95% CI: −0.010 to −0.009), with a gradual reduction from 2004 to 2016 (APC: −0.667, 95% CI: −0.760 to −0.573), followed by a steeper decline from 2016 to 2021 (APC: −3.581, 95% CI: −3.923 to −3.238) (Figure 1B). Throughout the entire study period, no NHL-related mortality cases (both in absolute numbers and mortality rates) were recorded among infants under 1 year of age. In contrast, all other age groups demonstrated a persistent downward trend in mortality rates (Figure 2B). By 2021, mortality remained highest in children aged 5–9 years, and across all age groups, mortality rates in males exceeded those in females (Figure 2B).

#### Disability-adjusted life years

Mirroring the trends observed in mortality, the DALYs rate associated with childhood NHL demonstrated a consistent decline from 1990 to 2021, with an AAPC of −0.827 (95% CI: −0.845 to −0.809). This downward trajectory included a gradual reduction from 2004 to 2016 (APC: −0.657, 95% CI: −0.749 to −0.565), followed by the steepest decline from 2019 to 2021 (APC: −3.659, 95% CI: −3.995 to −3.323) (Figure 1C). The EAPC was −1.51 (95% CI: −1.61 to −1.40), with DALYs per 100,000 population decreasing from 63.38 (95% UI: 48.09–75.72) in 1990 to 37.83 (95% UI: 29.95–47.17) in 2021 (Supplementary Table S2). The global DALYs burden also exhibited a marked reduction, with total DALYs cases decreasing by 30.96% (95% UI: −44.26%–6.73%), from 1,102,346.32 (95% UI: 836,303.95–1,316,917.95) in 1990 to 761,085.36 (95% UI: 602,606.50–948,912.73) in 2021 (Supplementary Table S2). Consistently, no DALYs cases or rates were recorded in infants under 1 year of age, whereas all other age groups experienced a declining trend in DALYs rates (Figure 2C). This absence likely reflects the extreme rarity of NHL in infants combined with the GBD 2021 modeling framework, which may generate zero estimates for age groups with exceptionally sparse or inconsistent data inputs. When compared to other age groups, children aged 1–2 years exhibited the highest DALYs rate throughout the study period, indicating a greater disease burden relative to population size. By 2021, across all age groups, male children had a higher DALYs burden than females (Figure 2C).

### Burden of childhood non-Hodgkin lymphoma in sociodemographic index regions

#### Incidence

**Figure 3:**
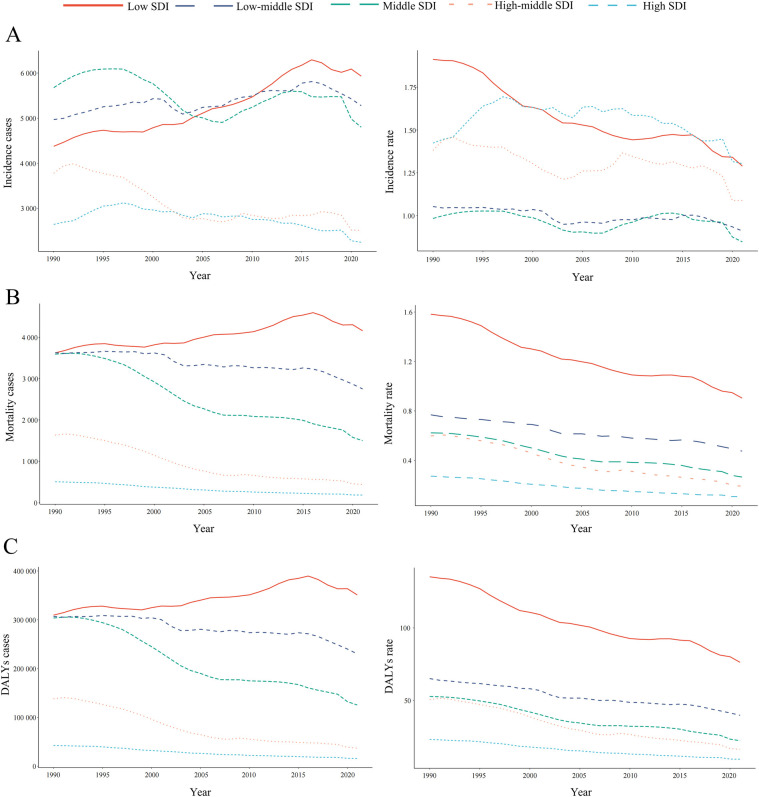
Epidemiologic trends in childhood non-hodgkin lymphoma incidence, mortality, and disability-adjusted life years (DALYs) rates across five sociodemographic Index (SDI) areas from 1990 to 2021. **(A)** Incidence. **(B)** Deaths. **(C)** DALYs.

In 2021, the low-SDI region recorded the highest number of childhood NHL incidence cases, totaling 5,932.40 (95% UI: 3,988.74–8,047.35) (Table 1 and Figure 3A). The greatest increase in incidence cases over time was also observed in this region, with a relative increase of 35.52% (95% UI: −0.41%–152.34%) compared to 1990 (Table 1 and Figure 3A). Notably, while the incidence rates declined across all five SDI regions, the most pronounced decline was observed in the low-SDI region, with an EAPC of −1.16 (95% CI: −1.28 to −1.04) (Table 1 and Figure 3A).

#### Mortality

Among the five SDI regions, only the low-SDI region exhibited an increase in NHL-related mortality cases, with a relative rise of 14.76% (95% UI: −12.81%–108.52%) (Supplementary Table S1). The high-SDI region consistently reported the lowest NHL-related mortality burden, whereas the low-SDI region had the highest mortality cases and rates from 1990 to 2021 (Supplementary Table S1 and Figure 3B). A particularly notable decline in mortality rates was observed in the high-middle SDI region, where the rate decreased from 0.60 (95% UI: 0.52–0.68) in 1990 to 0.19 (95% UI: 0.16–0.23) in 2021, corresponding to an EAPC of −3.69 (95% CI: −3.88 to −3.51) (Supplementary Table S1 and Figure 3B).

#### Disability-adjusted life years

Between 1990 and 2021, all SDI regions exhibited a downward trend in NHL-related DALYs rates, as reflected by negative EAPC values (Supplementary Table S2 and Figure 3C). However, the low-SDI region was the only one to experience an increase in total DALYs cases, with a relative rise of 14.76% (95% UI: −12.81% to 108.52%) (Supplementary Table S2). The low-SDI region consistently bore the highest DALYs burden, with cases increasing from 3,622.77 (95% UI: 2,370.30–4,792.28) in 1990 to 4,157.48 (95% UI: 2,868.69–5,570.47) in 2021 (Supplementary Table S2 and Figure 3C). Moreover, this region also reported the highest DALYs rate, decreasing from 1.58 (95% UI: 1.04–2.09) in 1990 to 0.90 (95% UI: 0.62–1.21) in 2021 (Supplementary Table S2 and Figure 3C).

### Burden of childhood non-Hodgkin lymphoma across 21 geographic regions

#### Incidence

**Figure 4:**
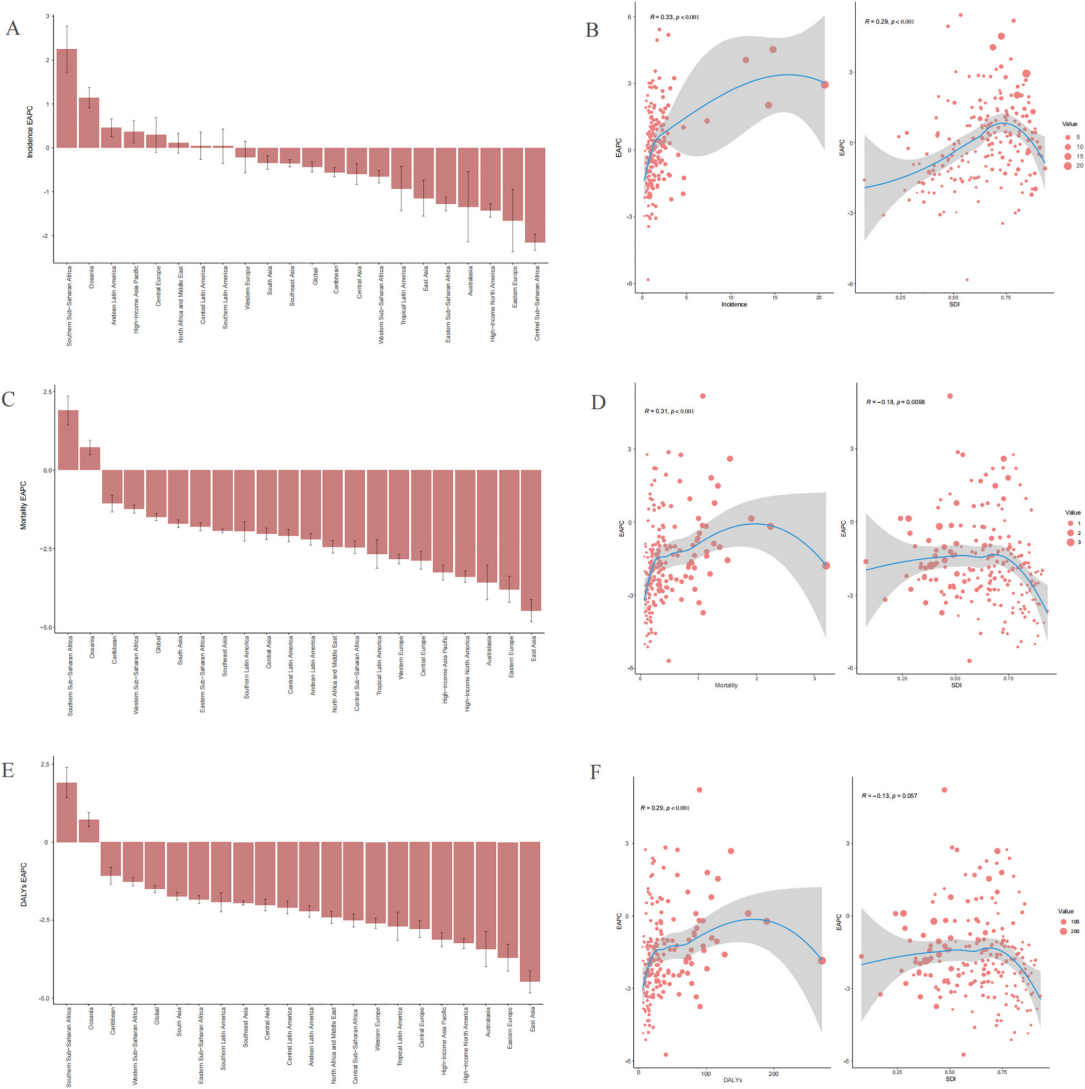
Trends in the estimated annual percentage changes (EAPC) of incidences, deaths, and disability-adjusted life years (DALYs) rate **(A)**, incidence EAPC. **(C)**, Mortality EAPC. **(E)** DALYs EAPC. The correlation between EAPC and sociodemographic index (SDI) of childhood non-Hodgkin lymphoma in 2021 **(B)** Incidence rate. **(D)** Mortality rate. **(F)** DALYs rate.

**Figure 5:**
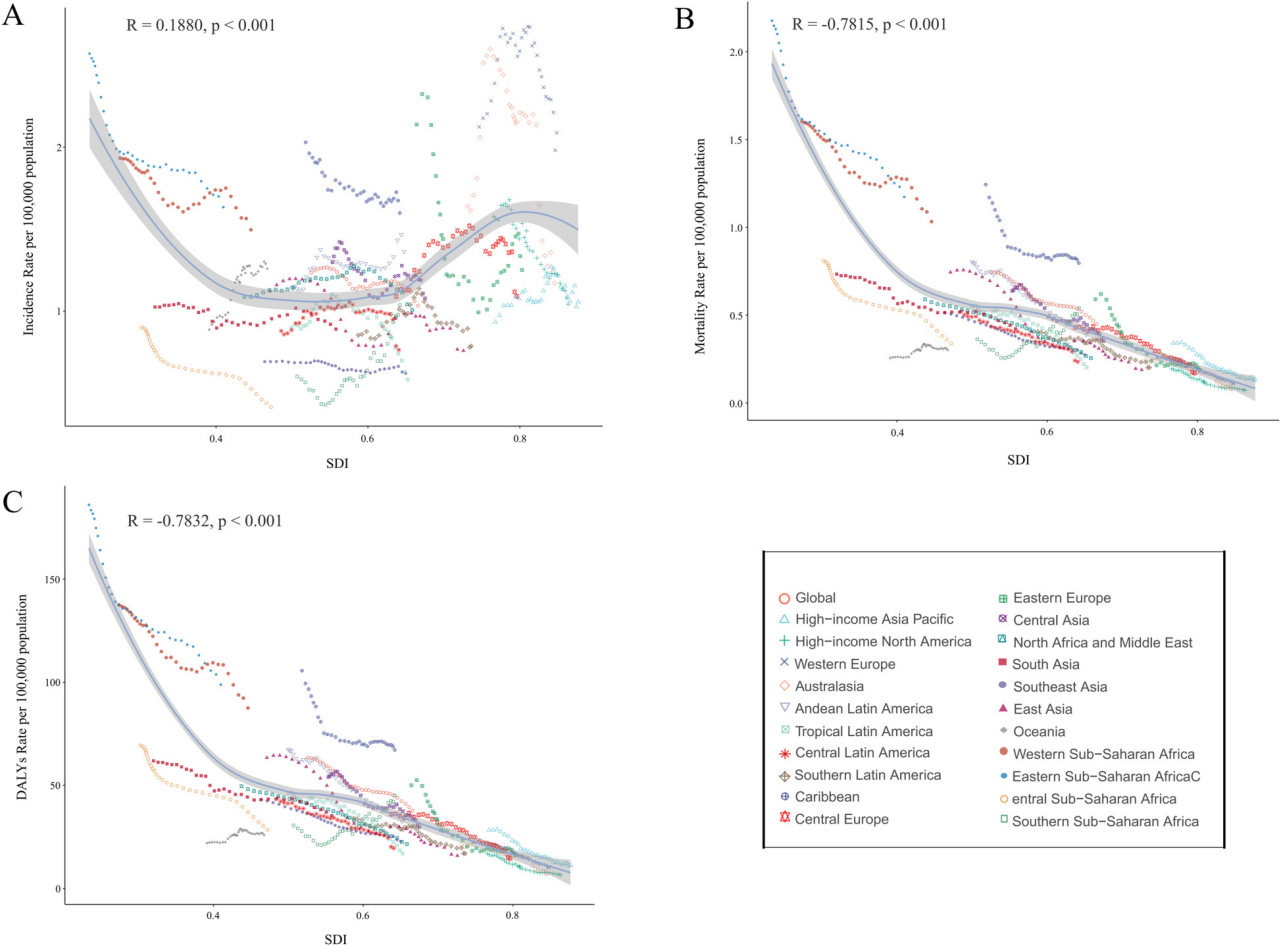
Association between incidence, mortality, and disability-adjusted life years (DALYs) rates of childhood non-hodgkin lymphoma and sociodemographic Index (SDI), 1990–2021. **(A)** Incidence rate. **(B)** Mortality rate. **(C)** DALYs rates.

Between 1990 and 2021, the highest number of childhood NHL incidence cases was reported in South Asia, far exceeding those in other regions (Table 1). In contrast, Oceania had the lowest recorded incidence cases, with only 36.97 cases (95% UI: 39.80–94.99) in 2021. The APC and AAPC trends in incidence rates across 21 regions from 1990 to 2021 are depicted in Supplementary Figures S1–S21. The most pronounced increase in incidence was observed in Southern Sub-Saharan Africa, with an EAPC of 2.25 (95% CI: 1.72–2.78) and an AAPC of 0.014 (95% CI: 0.012–0.016) (Table 1 and Supplementary Figure S20). Conversely, the steepest decline in AAPC was recorded in Eastern Sub-Saharan Africa, decreasing by −0.030 (95% CI: −0.031 to −0.029) from 1990 to 2021 (Supplementary Figure S19), whereas the largest decrease in EAPC was found in Central Sub-Saharan Africa, with a decline of −2.15 (95% CI: −2.34 to −1.96) (Table 1). Across the study period, 8 regions exhibited a positive EAPC, while 13 regions showed a negative EAPC (Table 1 and Figure 4A). By 2021, 13 regions (e.g., Western Europe and the Caribbean) had incidence rates exceeding the global mean, whereas 8 regions (e.g., East Asia and Central Latin America) had lower incidence rates than the global mean of 1.03 per 100,000 population (Table 1 and Figure 5A).

#### Mortality

The highest childhood NHL mortality rate in 2021 was observed in Eastern Sub-Saharan Africa, reaching 1.17 (95% UI: 0.79–1.66), while High-income North America reported the lowest mortality rate, at 0.07 per 100,000 (95% UI: 0.07–0.08) (Supplementary Table S1). The largest absolute decline in mortality cases was noted in East Asia, which experienced a 79.48% reduction (95% UI: −84.12% to −71.76%) from 2,454.63 deaths (95% UI: 2,046.08–2,989.85) in 1990 to 503.68 deaths (95% UI: 406.26–645.39) in 2021. This decline was accompanied by an AAPC of −0.018 (95% CI: −0.019 to −0.018) (Supplementary Table S1, Figure S22). Similarly, the mortality rate in East Asia showed a significant decrease, from 0.74 (95% UI: 0.62–0.91) in 1990 to 0.19 (95% UI: 0.15–0.24) in 2021, with an EAPC of −4.47 (95% CI: −4.82 to −4.12) (Supplementary Table S1). The APC and AAPC trends in mortality rates across 21 regions from 1990 to 2021 are presented in Supplementary Figures S22–S42. Notably, only two regions, Oceania and Southern Sub-Saharan Africa, exhibited an increasing mortality rate over the study period. The estimated EAPC values were 0.72 (95% CI: 0.49–0.95) for Oceania and 1.90 (95% CI: 1.44–2.36) for Southern Sub-Saharan Africa (Supplementary Table S1 and Figure 4C). By 2021, only four regions (e.g., Southern Sub-Saharan Africa) had mortality rates exceeding the global mean, whereas 17 regions (e.g., Central Europe) had lower mortality rates than the global mean of 0.45 per 100,000 population (Supplementary Table S1 and Figure 5B).

#### Disability-adjusted life years

The trends in DALYs burden across 21 regions from 1990 to 2021 closely mirrored those of mortality. The APC and AAPC of DALYs rates for childhood NHL across 21 regions are displayed in Supplementary Figures S43–S63. A 79.68% decrease (95% UI: −84.31% to −71.97%) in DALYs cases was observed in East Asia, declining from 209,159.50 (95% UI: 173,974.04–255,126.65) in 1990 to 42,491.44 (95% UI: 34,061.03–54,539.66) in 2021, with an AAPC of −1.561 (95% CI: −1.607 to −1.515) (Supplementary Table S2, Figure S43). The largest increase in DALYs rates was recorded in Southern Sub-Saharan Africa, with an AAPC of 0.461 (95% CI: 0.384–0.538) (Supplementary Table S2, Figure S62). Similar to mortality trends, only Oceania and Southern Sub-Saharan Africa exhibited an increasing DALYs rate from 1990 to 2021. The estimated EAPC values were 0.73 (95% CI: 0.50–0.96) for Oceania and 1.91 (95% CI: 1.43–2.40) for Southern Sub-Saharan Africa (Supplementary S2 and Figure 4E). By 2021, four regions (e.g., Southern Sub-Saharan Africa) had DALYs rates higher than the global mean, while 17 regions (e.g., Central Latin America) had lower DALYs rates than the global mean of 37.83 per 100,000 population (Supplementary Table S2 and Figure 5C).

### Burden of childhood non-Hodgkin lymphoma across 204 countries

#### Incidence

**Figure 6:**
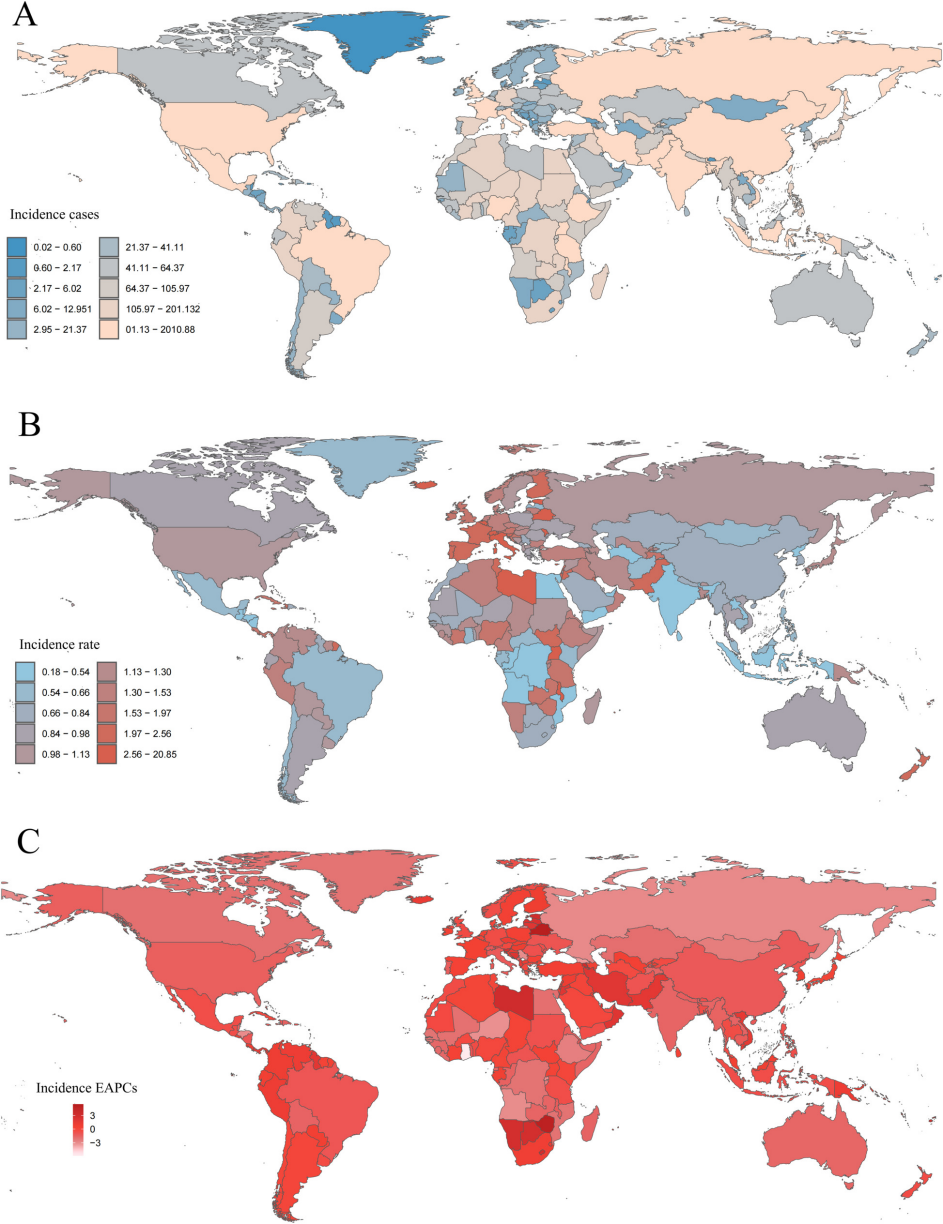
Incidence of childhood non-hodgkin lymphoma across 204 countries and territories. **(A)** Number of incidence cases. **(B)** Incidence rate. **(C)** Estimated annual percentage change (EAPC) in incidence.

In 2021, the Islamic Republic of Pakistan reported the highest number of childhood NHL cases, totaling 1,990.97 (95% UI: 1,340.98–2,880.86), whereas the Republic of Estonia had the highest incidence rate, reaching 20.64 per 100,000 (95% UI: 13.38–30.62) (Figures 6A,B and Supplementary Table S3). Between 1990 and 2021, incidence rates declined in 98 countries, while 106 countries experienced an increase (Supplementary Table S3). The Republic of Cabo Verde exhibited the largest increase in childhood NHL incidence rates (EAPC: 5.43, 95% CI: 4.55–6.32), whereas the Republic of Ghana showed the steepest decline (EAPC: −5.84, 95% CI: −6.91 to −4.76) (Figure 6C and Supplementary Table S3). By 2021, the Republic of Estonia maintained the highest incidence rate, at 20.64 per 100,000 (95% UI: 13.38–30.62), while the Republic of Honduras had the lowest, at 0.18 per 100,000 (95% UI: 0.09–0.32) (Supplementary Figure S64A, Table S3). The global incidence rate of childhood NHL in 2021 was 1.03 per 100,000 (95% UI: 0.85–1.26), with 112 countries exceeding the global mean, while 92 countries reported lower incidence rates (Supplementary Figure S64A, Table S3).

#### Mortality

In 2021, the Federal Republic of Nigeria recorded the highest number of childhood NHL-associated deaths, with 1,285.39 deaths (95% UI: 732.11–1,831.87). The Republic of Malawi had the highest mortality rate, at 3.21 per 100,000 (95% UI: 1.62–6.68), whereas the Hellenic Republic reported the lowest, at 0.06 per 100,000 (95% UI: 0.05–0.07) (Supplementary Table S4, Figure S65A,B). Between 1990 and 2021, 175 countries experienced a decline in mortality rates, while 29 countries saw an increase (Supplementary Table S4). The Republic of Zimbabwe recorded the largest increase in mortality rate (EAPC: 5.16, 95% CI: 4.00–6.34), whereas the Republic of Ghana demonstrated the greatest decline (EAPC: −5.70, 95% CI: −6.63 to −4.76) (Supplementary Table S4, Figure S65C). By 2021, the Republic of Malawi remained the country with the highest NHL-associated mortality rate, at 3.21 per 100,000 (95% UI: 1.62–6.68), while the Hellenic Republic had the lowest, at 0.06 per 100,000 (95% UI: 0.05–0.07) (Supplementary Figure S64B, Table S4). The global childhood NHL-associated mortality rate in 2021 was 0.45 per 100,000 (95% UI: 0.36–0.56), with 53 countries surpassing the global mean, while 151 countries reported lower mortality rates (Supplementary Figure S64B, Table S4).

#### Disability-adjusted life years

In 2021, the Federal Republic of Nigeria recorded the highest number of childhood NHL-associated DALYs, totaling 109,622.16 (95% UI: 61,684.54–157,384.74), whereas the Republic of Malawi had the highest DALYs rate, reaching 270.85 per 100,000 (95% UI: 135.35–569.42) (Supplementary Table S5, Figures S66A,B). Between 1990 and 2021, 174 countries experienced a decrease in DALYs rates, while 30 countries exhibited an increase. The Republic of Zimbabwe recorded the largest rise in DALYs rate (EAPC: 5.22, 95% CI: 4.03–6.42), while the Republic of Ghana saw the sharpest decline (EAPC: −5.74, 95% CI: −6.68 to −4.80) (Supplementary Table S5, Figure S66C). By 2021, the Republic of Malawi maintained the highest NHL-associated DALYs rate, at 270.85 per 100,000 (95% UI: 135.35–569.42), whereas the Hellenic Republic had the lowest, at 5.57 per 100,000 (95% UI: 4.80–6.43) (Supplementary Figure S64C, Table S5). The global childhood NHL-associated DALYs rate in 2021 was 37.83 per 100,000 (95% UI: 29.95–47.17), with 52 countries exceeding the global mean, while 152 countries had lower DALYs rates (Supplementary Figure S64C, Table S5).

Notably, several countries exhibited dramatic increases in childhood NHL burden over the study period. For example, the Republic of Cabo Verde and Zimbabwe showed over 200% increases in incidence and DALYs rates, respectively, while Estonia and Malawi remained among the highest-burden countries in 2021. These findings underscore the considerable cross-national disparities in disease burden and the urgent need for region-specific cancer control strategies.

#### Correlation of EAPC with sociodemographic index

In 2021, across 204 countries, the incidence rate, mortality rate, and DALYs rate, along with their corresponding EAPCs, demonstrated a statistically significant positive correlation (R = 0.33, *p* < 0.001; R = 0.31, *p* < 0.001; R = 0.29, *p* < 0.001) (Figures 4B,D,F). The EAPCs for mortality rate and DALYs rate exhibited a negative correlation with SDI (R = −0.18, *p* = 0.0098; R = −0.13, *p* = 0.057), whereas the EAPC for incidence rate showed a positive correlation with SDI (R = 0.29, *p* < 0.001) (Figures 4B,D,F).

### Relation between sociodemographic Index and incidence, mortality, and disability-adjusted life year rates

Between 1990 and 2021, within 21 regions of the GBD geographic classification, SDI displayed a strong negative correlation with mortality and DALYs rates (R = −0.7815, *p* < 0.001; R = −0.7832, *p* < 0.001), while the incidence rate was positively correlated with SDI (R = 0.1880, *p* < 0.001) (Figure 5). Similarly, across 204 countries, SDI exhibited a strong negative correlation with mortality and DALYs rates (R = −0.6979, *p* < 0.001; R = −0.6749, *p* < 0.001), whereas the incidence rate maintained a positive correlation with SDI (R = 0.2816, *p* < 0.001) (Supplementary Figure S64).

### Cross-country health inequality analysis

**Figure 7:**
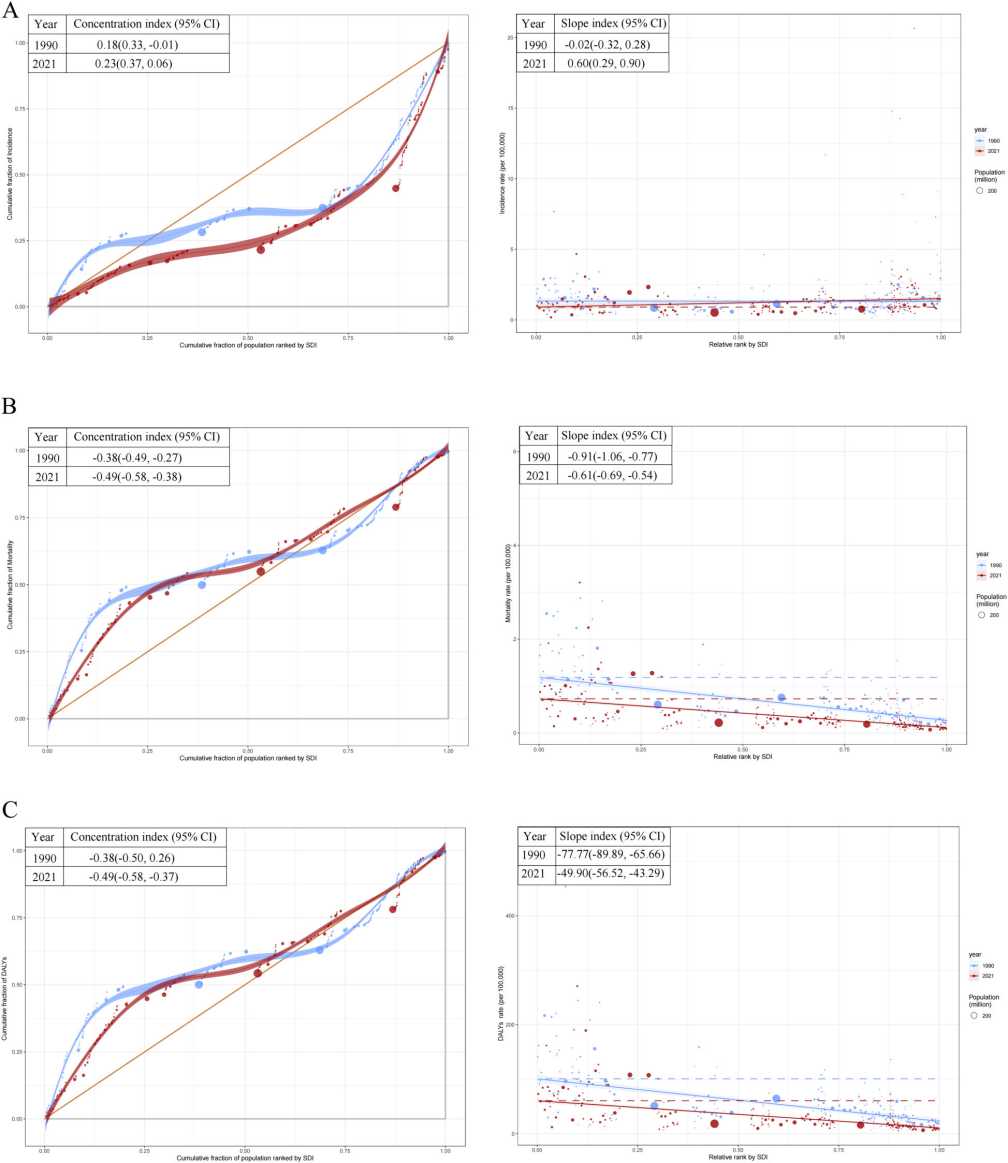
Health inequality regression curves and concentration curves for the incidence, mortality, and disability-adjusted life years (DALYs) rates of childhood non-hodgkin lymphoma. **(A)** Incidence rate. **(B)** Mortality rate. **(C)** DALYs rates. SDI, socio-demographic index.

The slope index revealed an increasing disparity in incidence rates, shifting from −0.02 (95% CI: −0.32–0.28) in 1990 to 0.60 (95% CI: 0.29–0.90) in 2021. Additionally, the concentration index for incidence rates increased from 0.18 (95% CI: −0.01–0.33) in 1990 to 0.23 (95% CI: 0.06–0.37) in 2021 (Figure 7A). Countries with lower SDI exhibited a higher burden of mortality and DALYs, both in absolute and relative terms, in 1990 and 2021 (Figures 7B,C). For mortality rates, the slope index difference between the highest and lowest SDI countries narrowed from −0.91 (95% CI: −1.06 to −0.77) in 1990 to −0.61 (95% CI: −0.69 to −0.54) in 2021. The concentration index for mortality rates shifted from −0.38 (95% CI: −0.49 to −0.27) in 1990 to −0.49 (95% CI: −0.58 to −0.38) in 2021 (Figure 7B). For DALYs rates, the slope index difference between high- and low-SDI countries decreased from −77.77 (95% CI: −89.89 to −65.66) in 1990 to −49.90 (95% CI: −56.52 to −43.29) in 2021. Likewise, the concentration index for DALYs shifted from −0.38 (95% CI: −0.50 to −0.26) in 1990 to −0.49 (95% CI: −0.58 to −0.37) in 2021 (Figure 7C).

### Frontier analysis

**Figure 8:**
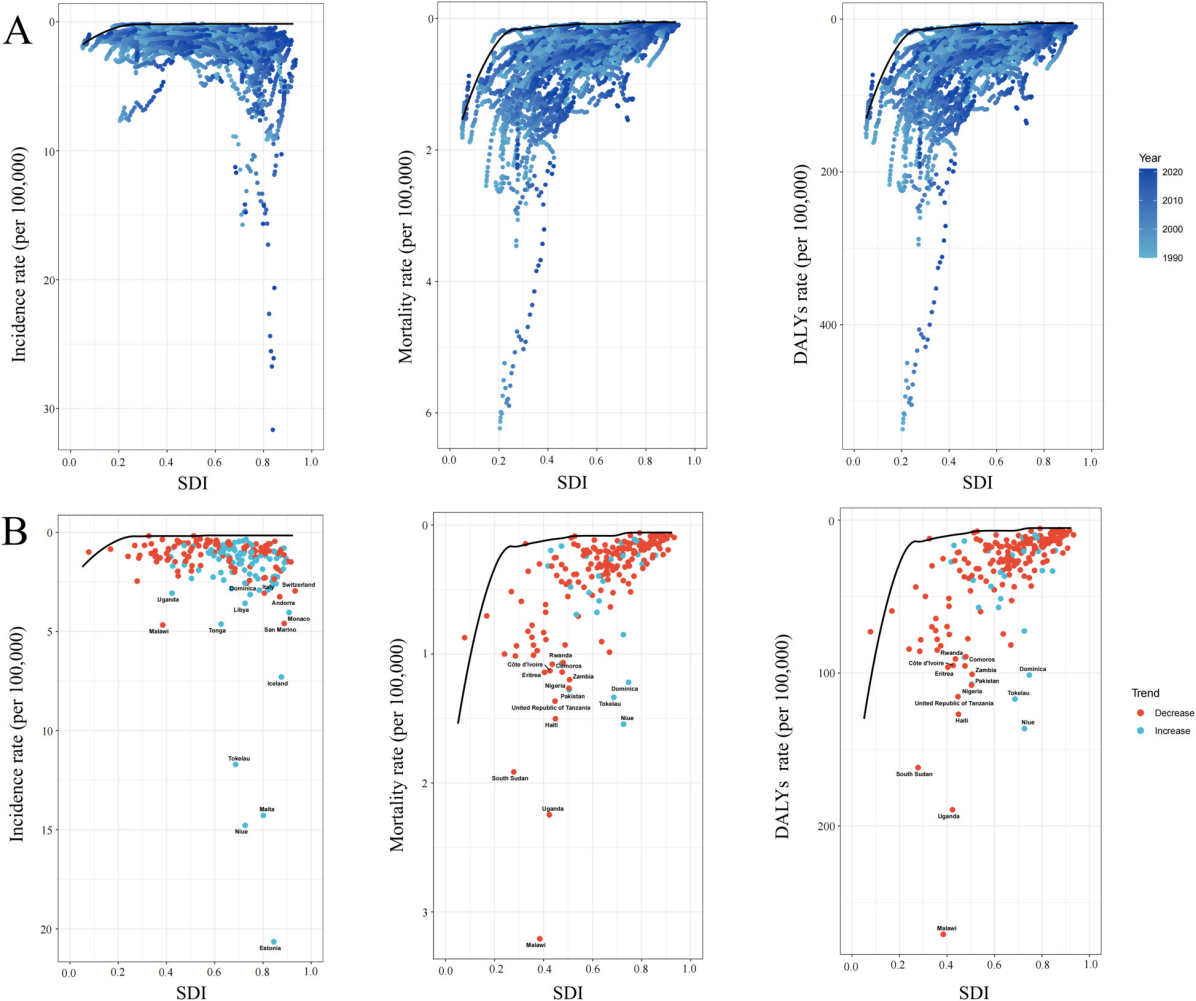
Frontier analysis exploring the relationship between sociodemographic index (SDI) and the incidence mortality and disability-adjusted life years (DALYs) of childhood non-hodgkin lymphoma across 204 countries and territories. **(A)** The frontier curves (black lines) represent the lowest theoretically achievable burden for a given SDI level from 1990 to 2021, with individual countries plotted as dots. The color gradient from light blue (1990) to dark blue (2021) indicates the temporal progression. **(B)** The burden trends of incidence, mortality, and DALYs rates in 2021, with each dot representing a country. Red dots indicate a decrease in burden from 1990 to 2021, while blue dots indicate an increase. The top countries with the largest differences from the frontier are labeled.

Using data from 1990 to 2021, a frontier analysis was conducted to evaluate the potential for burden reduction in childhood NHL by examining incidence, mortality, and DALYs rates in relation to SDI at national and regional levels (Figure 8). The color scale represents the temporal progression from 1990 (light blue) to 2021 (dark blue). The frontier curve (solid black line) delineates the lowest theoretically achievable burden levels for a given SDI, serving as a benchmark for optimal performance (Figures 8A,B). Each country or territory is represented as a dot, where those positioned near the frontier indicate an optimal balance between NHL burden and development level, while those situated far above the frontier demonstrate a disproportionately high burden, reflecting higher-than-expected incidence, mortality, or DALYs rates. The temporal trend analysis further illustrates burden dynamics, where red dots signify a reduction in NHL burden between 1990 and 2021, whereas blue dots indicate an increasing burden over time.

The analysis revealed a general trend where the effective difference in mortality and DALYs rates for a given SDI level decreased and exhibited lower variance as SDI increased, suggesting that higher-SDI countries tend to have more consistent NHL outcomes (Figure 8B). However, incidence rates showed an opposing pattern, with greater variability observed in more developed regions. Notably, Estonia, Malta, Niue, and Tokelau, despite having relatively high SDI, exhibited disproportionately high increases in incidence rates, significantly surpassing the expected levels for their developmental status. In contrast, in low-SDI regions, Malawi, Uganda, and South Sudan displayed markedly higher mortality and DALYs rates compared to other countries with similar sociodemographic profiles, highlighting substantial unmet needs in pediatric NHL care within these settings.

## Discussion

Childhood NHL is a prevalent non-solid tumor in children under 15, imposing significant physical, psychological, and economic burdens on affected individuals, families, and healthcare systems. This study systematically assessed the global, regional, and national burden of childhood NHL from 1990 to 2021 using GBD 2021 data. By incorporating EAPC and AAPC, we quantified long-term trends, revealing a complex incidence pattern with substantial heterogeneity across SDI levels and geographic regions, while mortality and DALYs have markedly declined over the past three decades. These findings provide critical epidemiological insights to inform targeted prevention and control strategies, supporting ongoing efforts to alleviate the global childhood NHL burden. The data serve as a foundation for policy development, resource optimization, and healthcare planning, facilitating more effective interventions and ultimately improving outcomes for affected children worldwide.

Our findings reveal a marked decline in childhood NHL incidence from 2016 to 2021, following a prolonged period of stability or increase. This shift likely reflects multiple factors, particularly advancements in infection prevention and control. Given NHL's strong association with immune dysfunction and viral infections—most notably Epstein–Barr virus (EBV) and human immunodeficiency virus (HIV)—improvements in public health interventions may have significantly influenced these trends (22–24). Expanded antiretroviral therapy programs over the past decade have markedly reduced perinatal HIV transmission, lowering HIV-associated NHL incidence (25). Additionally, improved hygiene, broader vaccination coverage, and enhanced antiviral strategies may have mitigated EBV-related NHL burden (26, 27). Advances in cancer screening and diagnostic precision may have also impacted incidence trends by facilitating early detection of precursor conditions or refining disease classification (28, 29). Collectively, these factors likely contributed to the observed decline in childhood NHL incidence.

The global decline in childhood NHL mortality and DALYs rates from 1990 to 2021 reflects major advancements in cancer treatment, supportive care, and healthcare accessibility. The introduction of more effective chemotherapy regimens, immunotherapy agents (e.g., rituximab), and hematopoietic stem cell transplantation has significantly improved survival (30, 31). Enhanced management of treatment-related toxicities, infections, and supportive care, including prophylactic antibiotics, antifungal agents, and optimized transfusion protocols, has further reduced mortality (32). Additionally, earlier diagnosis, driven by greater awareness and expanded cancer screening programs, has facilitated timely interventions, contributing to the decline in NHL-related deaths (30, 33).

Beyond clinical advancements, socioeconomic and healthcare policy reforms have been pivotal in reducing childhood NHL mortality and DALYs. Expanded access to pediatric oncology centers, broader healthcare coverage, and global initiatives such as the WHO Global Initiative for Childhood Cancer have significantly improved treatment availability, particularly in low- and middle-income countries (22, 27). Strengthened public health infrastructure, enhanced infection control, and nutritional improvements have likely further contributed to increased survival (34). While NHL remains a major global health concern, the sustained decline in mortality and disease burden reflects the success of multidisciplinary treatment strategies and systemic healthcare improvements over the past three decades.

The greater decline in childhood NHL mortality and DALYs rates in high-SDI regions reflects advancements in early diagnosis, treatment accessibility, and supportive care. These countries benefit from well-established pediatric oncology programs, broad access to chemotherapy and targeted therapies, and improved infection management strategies, all contributing to better survival outcomes (35, 36). Additionally, robust healthcare infrastructure, cancer registries, and national cancer control programs have facilitated early detection and timely intervention, further improving survival (36). Socioeconomic stability in high-SDI countries supports better nutrition, post-treatment care, and comprehensive insurance coverage, reducing treatment discontinuation and late-stage diagnoses. However, the persisting disparities in NHL burden reduction between high- and low-SDI regions underscore the urgent need to strengthen pediatric oncology capacity, expand access to essential therapies, and enhance healthcare infrastructure in low-SDI regions. Addressing these challenges through international collaboration, healthcare investment, and policy reforms will be critical in reducing global inequalities in childhood NHL outcomes. In line with our findings, a nationwide study from the Netherlands analyzing childhood NHL between 1990 and 2015 reported that while the incidence rate remained stable over time (37), there was a significant decline in mortality and a corresponding improvement in survival rates during the same period. The authors attributed this progress primarily to advances in treatment protocols, centralized care, and enhanced supportive therapies. This supports our interpretation that the global decline in NHL mortality and DALYs may reflect real gains in clinical management, particularly in high-income countries with accessible and specialized pediatric oncology care.

Among all countries analyzed, Ghana exhibited the most significant decline in childhood NHL incidence, mortality, and DALYs rates, reflecting remarkable progress in pediatric oncology despite its moderate SDI level. Over the past two decades, Ghana has expanded pediatric oncology services, particularly through major referral centers such as Korle Bu Teaching Hospital (38). The inclusion of select pediatric cancer treatments under Ghana's National Health Insurance Scheme (NHIS) has likely improved access to life-saving therapies (39). Global collaborations in pediatric oncology, such as the Wilms Africa Project, have provided critical insights into establishing sustainable childhood cancer care frameworks (40). Efforts to enhance pediatric neuro-oncology services and cross-border research initiatives have further strengthened medical capacity for childhood cancer treatment in Africa (41). While Ghana remains a middle-SDI country facing economic and healthcare challenges, its success in reducing childhood NHL mortality underscores the transformative impact of strategic healthcare investments, global partnerships, and heightened cancer awareness. This highlights the importance of replicating similar models in other low- and middle-SDI countries to reduce global disparities in childhood NHL outcomes.

This study presents a comprehensive analysis of the global burden of childhood NHL from 1990 to 2021 using GBD 2021 data, providing critical insights for policymakers and healthcare professionals in shaping prevention, management, and treatment strategies. However, limitations must be acknowledged. Data quality and completeness vary across countries, particularly in low-SDI regions where underdeveloped cancer registries may lead to underreporting or misclassification of cases. Additionally, GBD estimates rely on statistical modeling and extrapolation, which, despite their robustness, introduce uncertainties, especially in regions with scarce primary data. Furthermore, GBD data do not establish direct causal relationships. Some fluctuations in case numbers between years or regions may reflect not only actual changes in disease burden but also inconsistencies in cancer registry coverage, variation in data quality, or changes in GBD modeling inputs over time. These limitations should be considered when interpreting temporal trends. It is also important to recognize that the burden estimates for childhood NHL are derived from secondary sources and rely heavily on model-based imputation where empirical data are limited or missing. The assumptions used in the GBD modeling framework, including the choice of covariates and smoothing techniques, may affect the comparability of results across countries or time periods. Although GBD provides the most comprehensive global estimates currently available, the findings should be interpreted with caution, especially in countries lacking high-quality population-based cancer surveillance systems. Future studies based on primary data from national registries and hospital-based cohorts are needed to validate and refine these global patterns. In addition, evolving diagnostic criteria and classification systems for NHL—particularly updates to the WHO lymphoma classification—may have influenced case identification and reporting practices over the study period. These changes could partially affect the comparability of temporal trends, especially when interpreting shifts in incidence across decades.

In summary, the burden of childhood NHL has evolved significantly, with mortality and DALYs rates showing a steady decline from 1990 to 2021. While high-SDI regions have seen substantial improvements, disparities persist, as low-SDI regions continue to grapple with limited medical resources, delayed diagnosis, and restricted access to life-saving therapies, exacerbating disease burden. Urgent policy interventions are needed to strengthen prevention, enhance healthcare infrastructure, and improve treatment accessibility, ultimately reducing disparities, supporting affected families, and alleviating economic strain on society.

## Data Availability

Publicly available datasets were analyzed in this study. This data can be found here: GBD.
