# Supplementary material for: Global burden and trends of childhood non-Hodgkin lymphoma from 1990 to 2021
Source: Front Pediatr. 2025 Jun 26;13:1618810. doi: 10.3389/fped.2025.1618810 (PMC12241043; doi:10.3389/fped.2025.1618810)
Supplement: Supplementary file 1 [file Datasheet1.pdf]

Figure S1

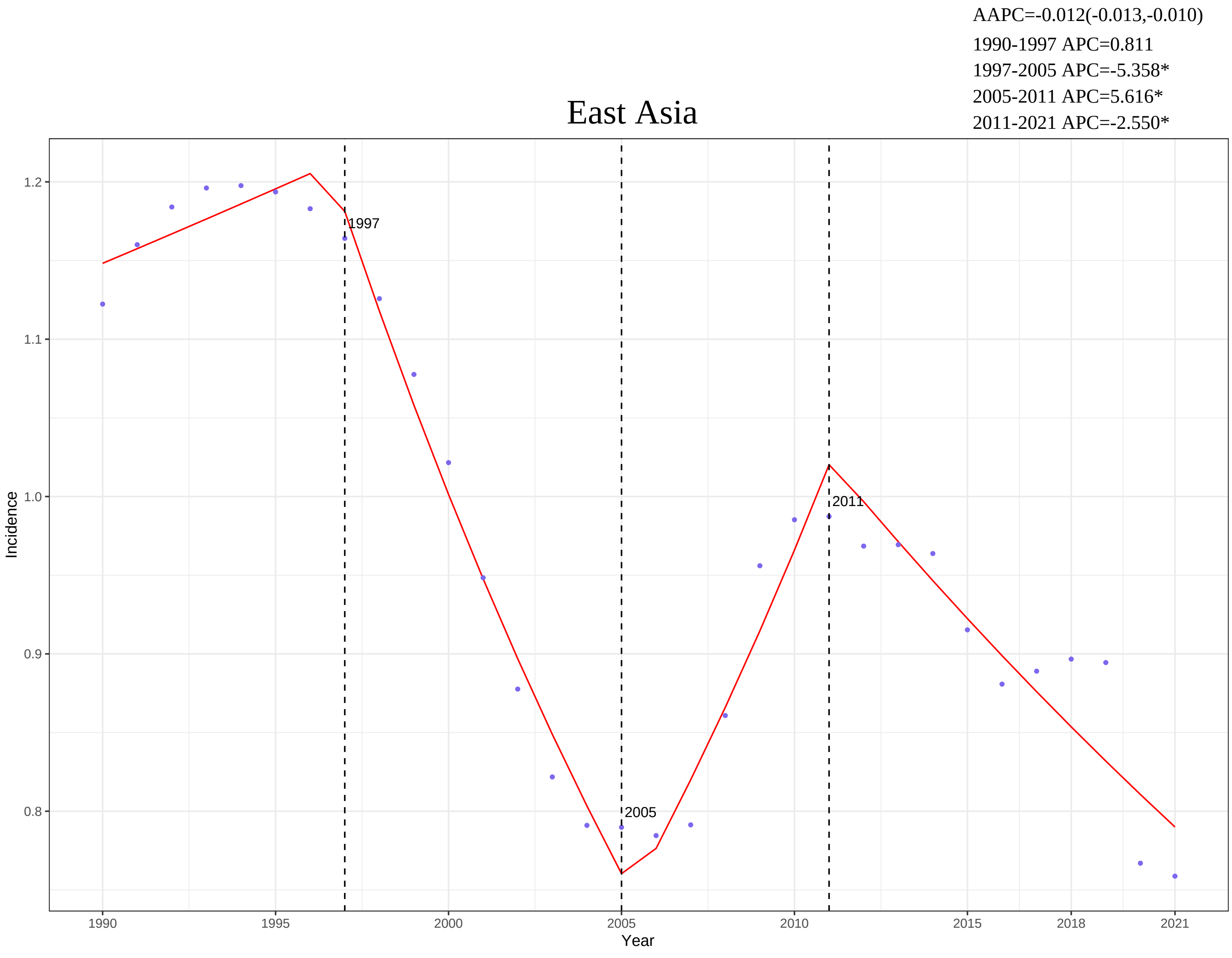
AAPC=-0.012(-0.013,-0.010)
1990-1997 APC=0.811
1997-2005 APC=-5.358*
2005-2011 APC=5.616*
2011-2021 APC=-2.550*
East Asia
1.2
1.1
1.0
0.9
0.8
Incidence
1997
2005
2011
1990
1995
2000
2005
2010
2015
2018
2021
Year

Figure S2

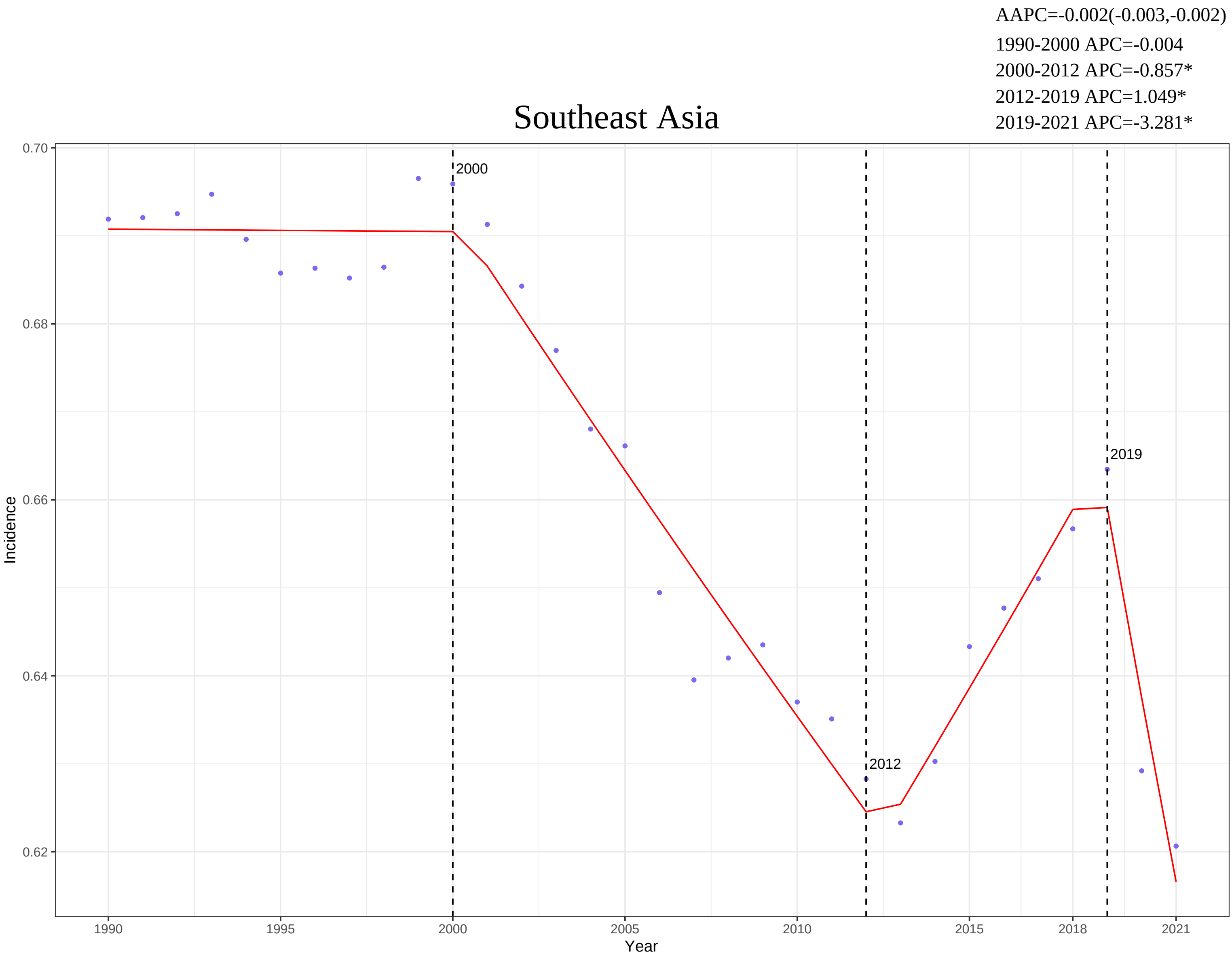
AAPC=-0.002(-0.003,-0.002)
1990-2000 APC=-0.004
2000-2012 APC=-0.857*
2012-2019 APC=1.049*
2019-2021 APC=-3.281*
Southeast Asia
2000
2012
2019
Incidence
0.70
0.68
0.66
0.64
0.62
Year
1990
1995
2000
2005
2010
2015
2018
2021

Figure S3

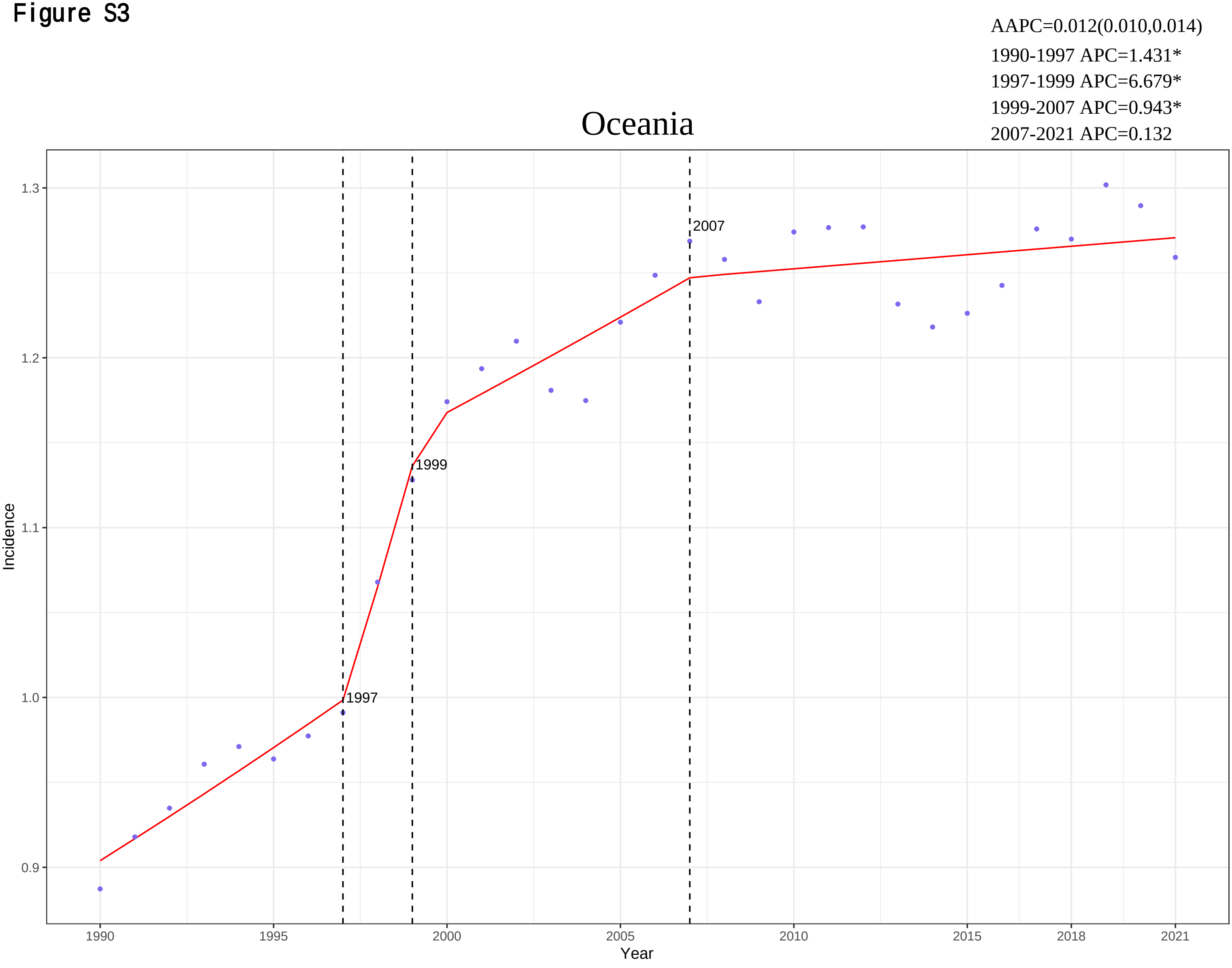

AAPC=0.012(0.010,0.014)
1990-1997 APC=1.431*
1997-1999 APC=6.679*
1999-2007 APC=0.943*
2007-2021 APC=0.132
Oceania
1997
1999
2007
Incidence
1.3
1.2
1.1
1.0
0.9
1990
1995
2000
2005
2010
2015
2018
2021
Year

Figure S4

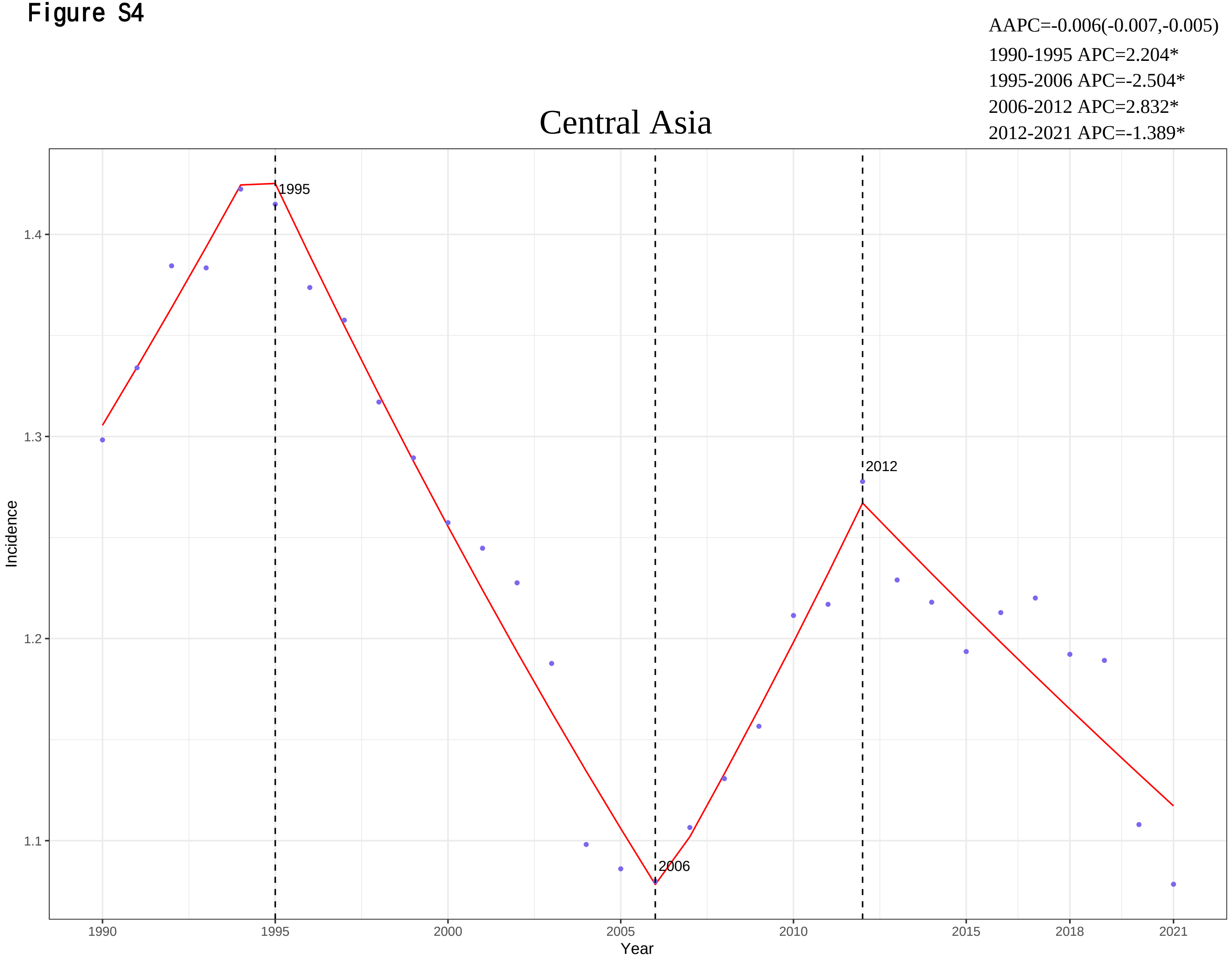

AAPC=-0.006(-0.007,-0.005)
1990-1995 APC=2.204*
1995-2006 APC=-2.504*
2006-2012 APC=2.832*
2012-2021 APC=-1.389*
Central Asia
Incidence
1.4
1.3
1.2
1.1
1995
2006
2012
1990
1995
2000
2005
2010
2015
2018
2021
Year

Figure S5

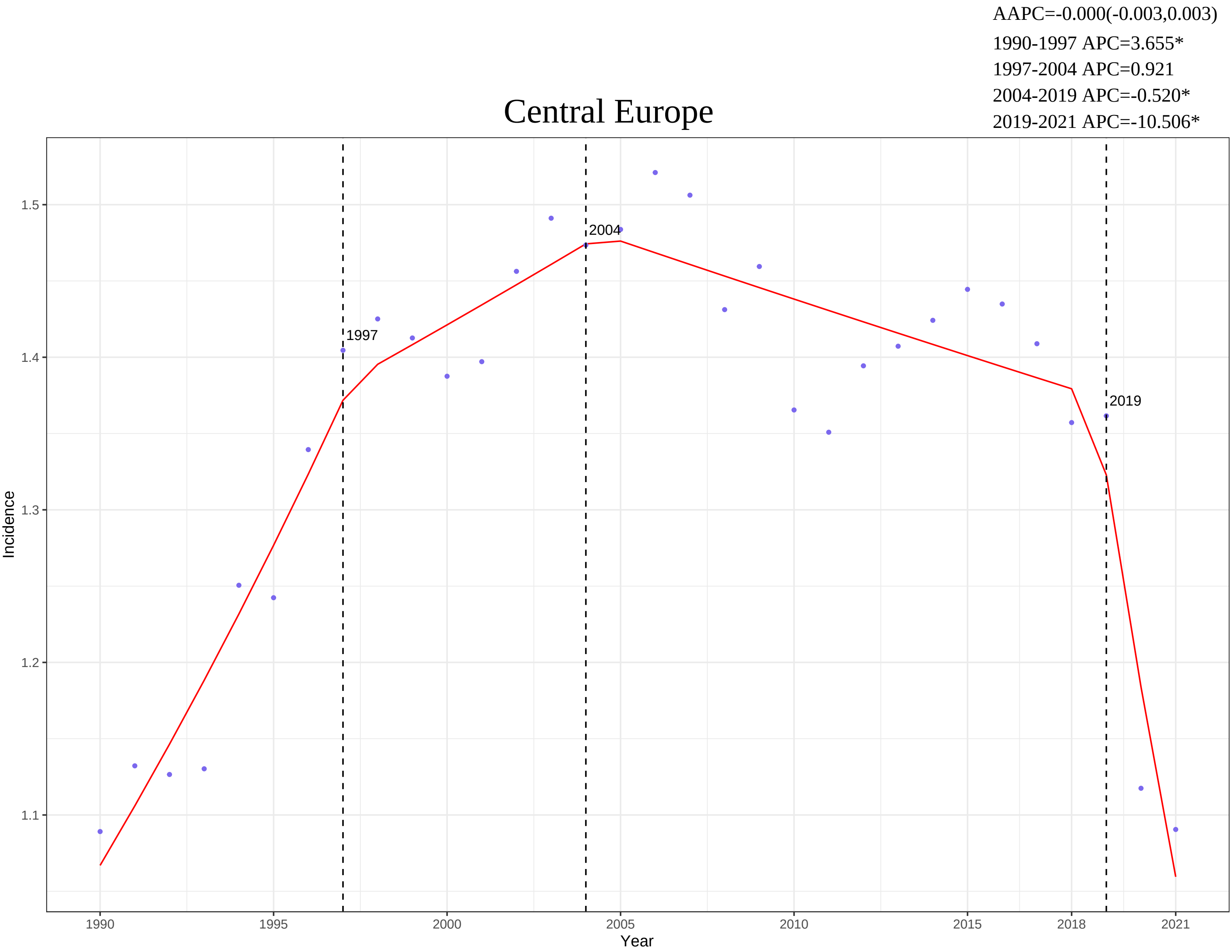
AAPC=-0.000(-0.003,0.003)
1990-1997 APC=3.655*
1997-2004 APC=0.921
2004-2019 APC=-0.520*
2019-2021 APC=-10.506*
Central Europe
1997
2004
2019
Incidence
Year
1.1
1.2
1.3
1.4
1.5
1990
1995
2000
2005
2010
2015
2018
2021

Figure S6

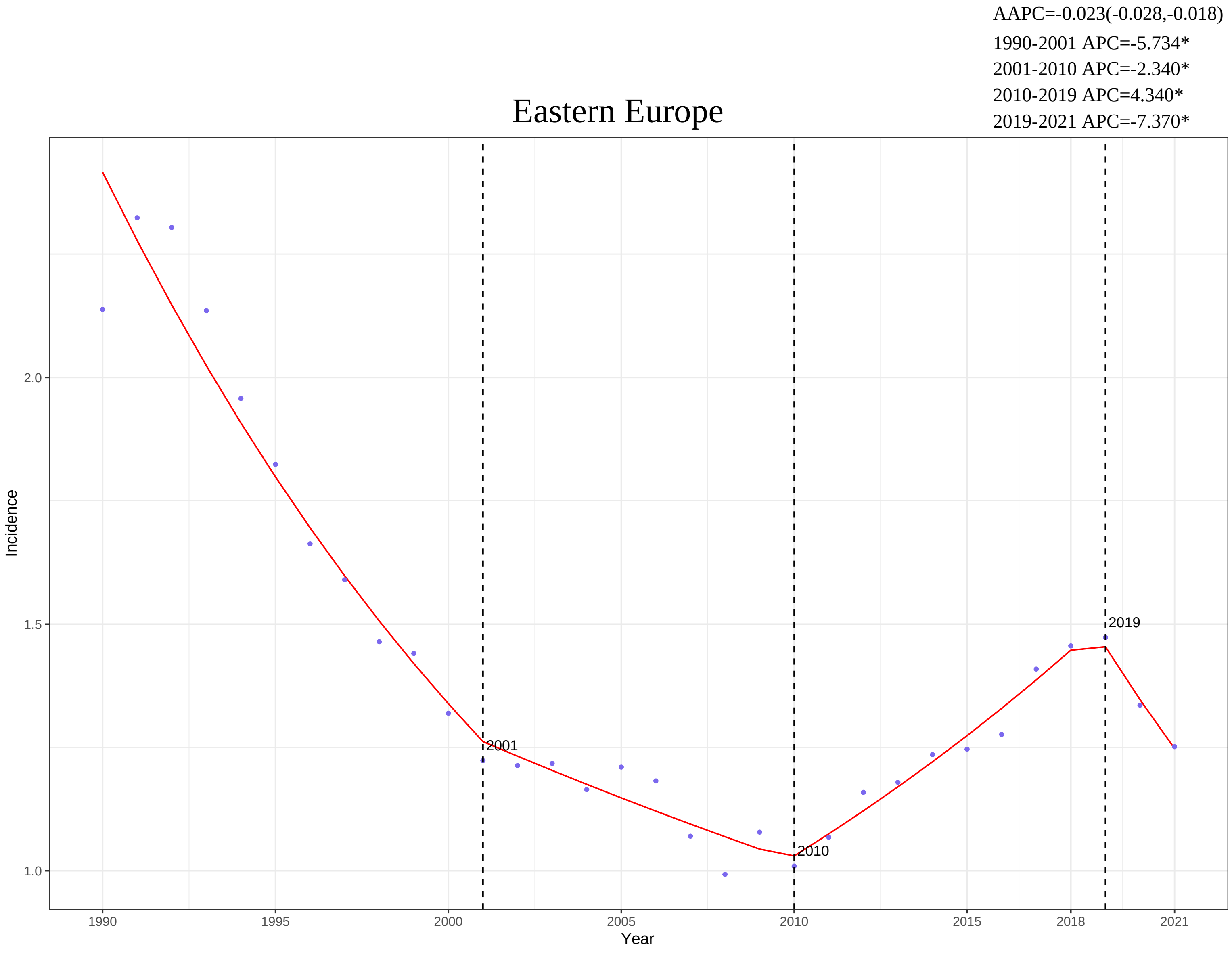
AAPC=-0.023(-0.028,-0.018)
1990-2001 APC=-5.734*
2001-2010 APC=-2.340*
2010-2019 APC=4.340*
2019-2021 APC=-7.370*
Eastern Europe
Incidence
2.0
1.5
1.0
2001
2010
2019
1990
1995
2000
2005
2010
2015
2018
2021
Year

Figure S7

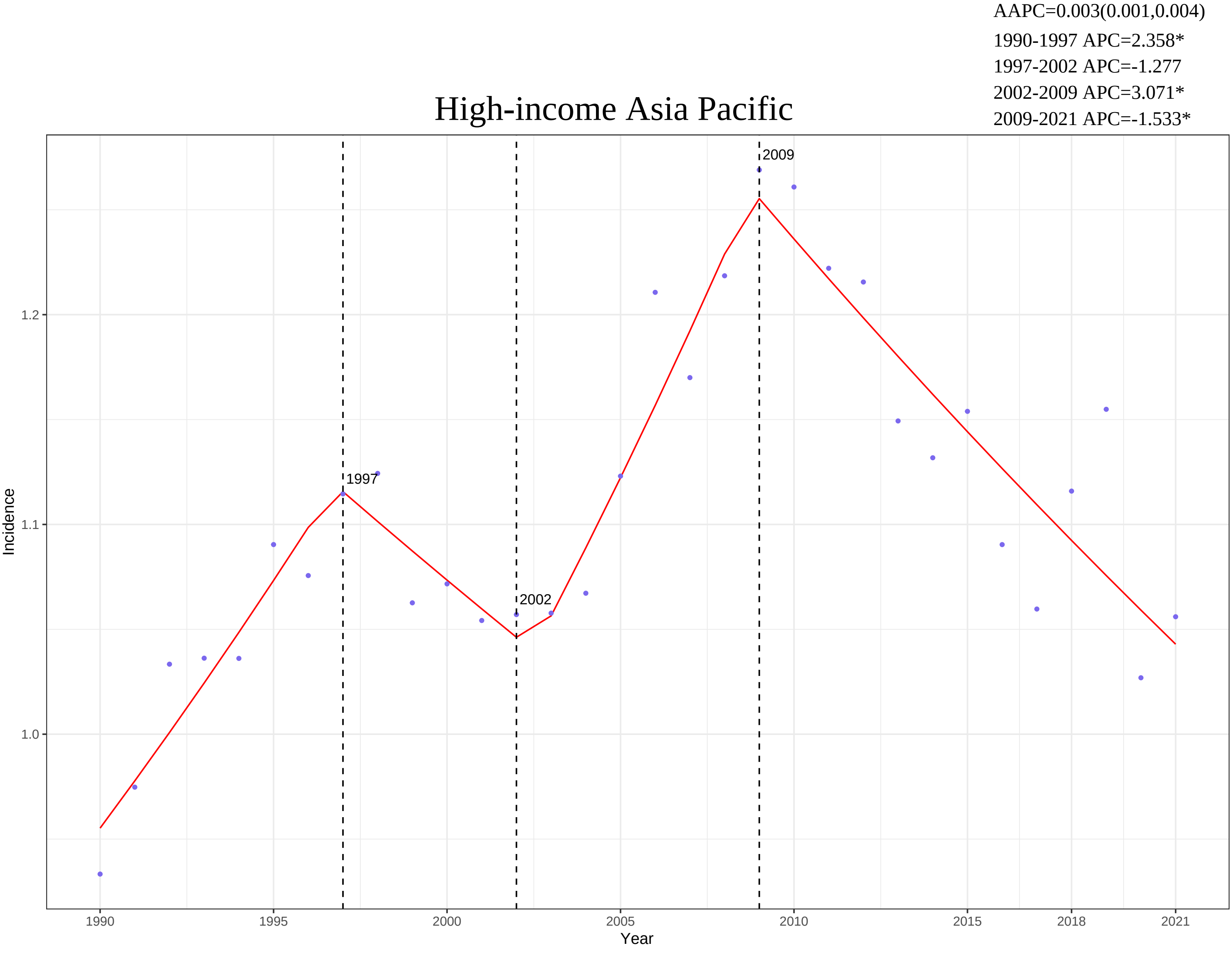
AAPC=0.003(0.001,0.004)
1990-1997 APC=2.358*
1997-2002 APC=-1.277
2002-2009 APC=3.071*
2009-2021 APC=-1.533*
High-income Asia Pacific
1997
2002
2009
1.2
1.1
1.0
Incidence
1990
1995
2000
2005
2010
2015
2018
2021
Year

Figure S8

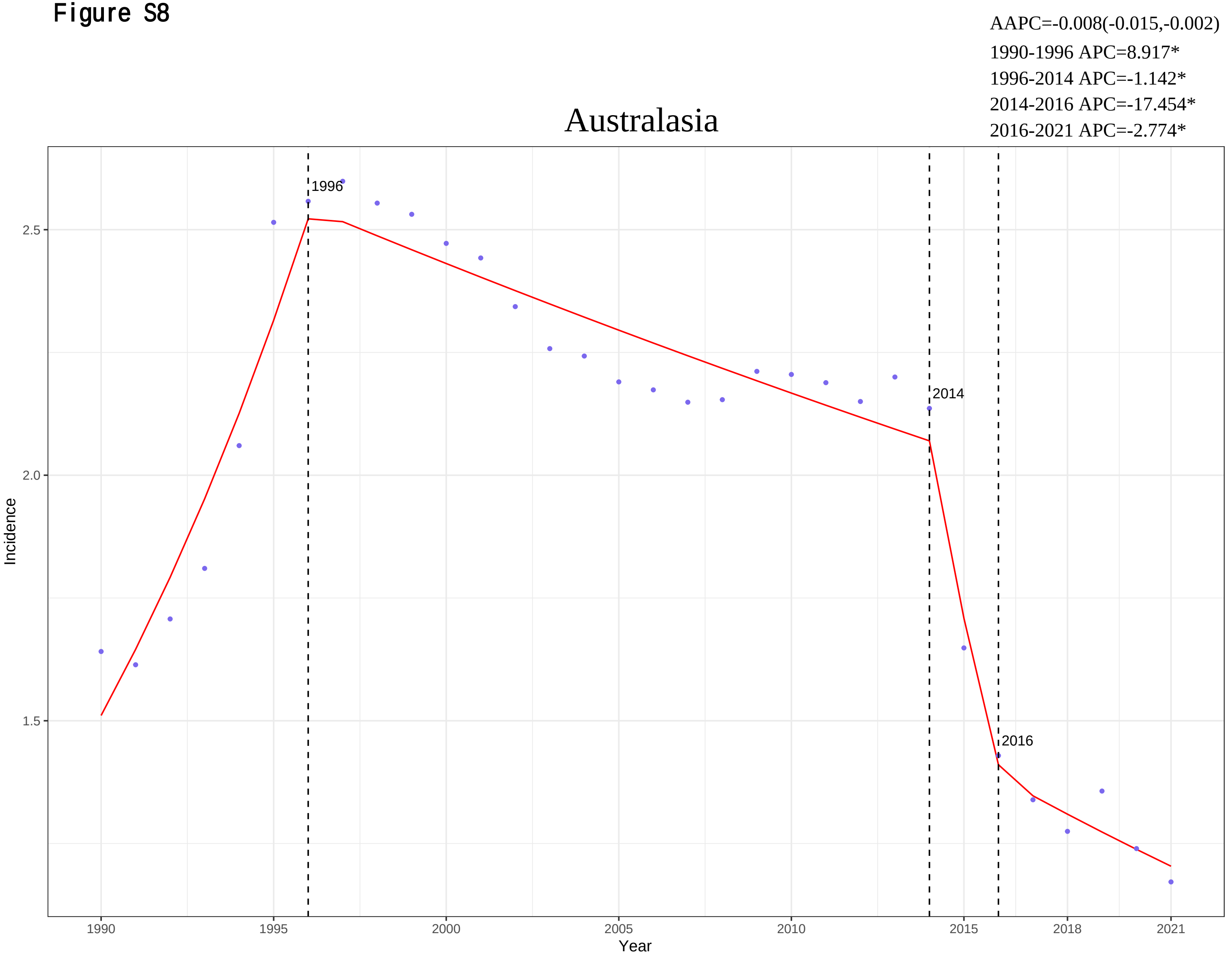

AAPC=-0.008(-0.015,-0.002)
1990-1996 APC=8.917*
1996-2014 APC=-1.142*
2014-2016 APC=-17.454*
2016-2021 APC=-2.774*
Australasia
1996
2014
2016
Incidence
2.5
2.0
1.5
Year
1990
1995
2000
2005
2010
2015
2018
2021

Figure S9

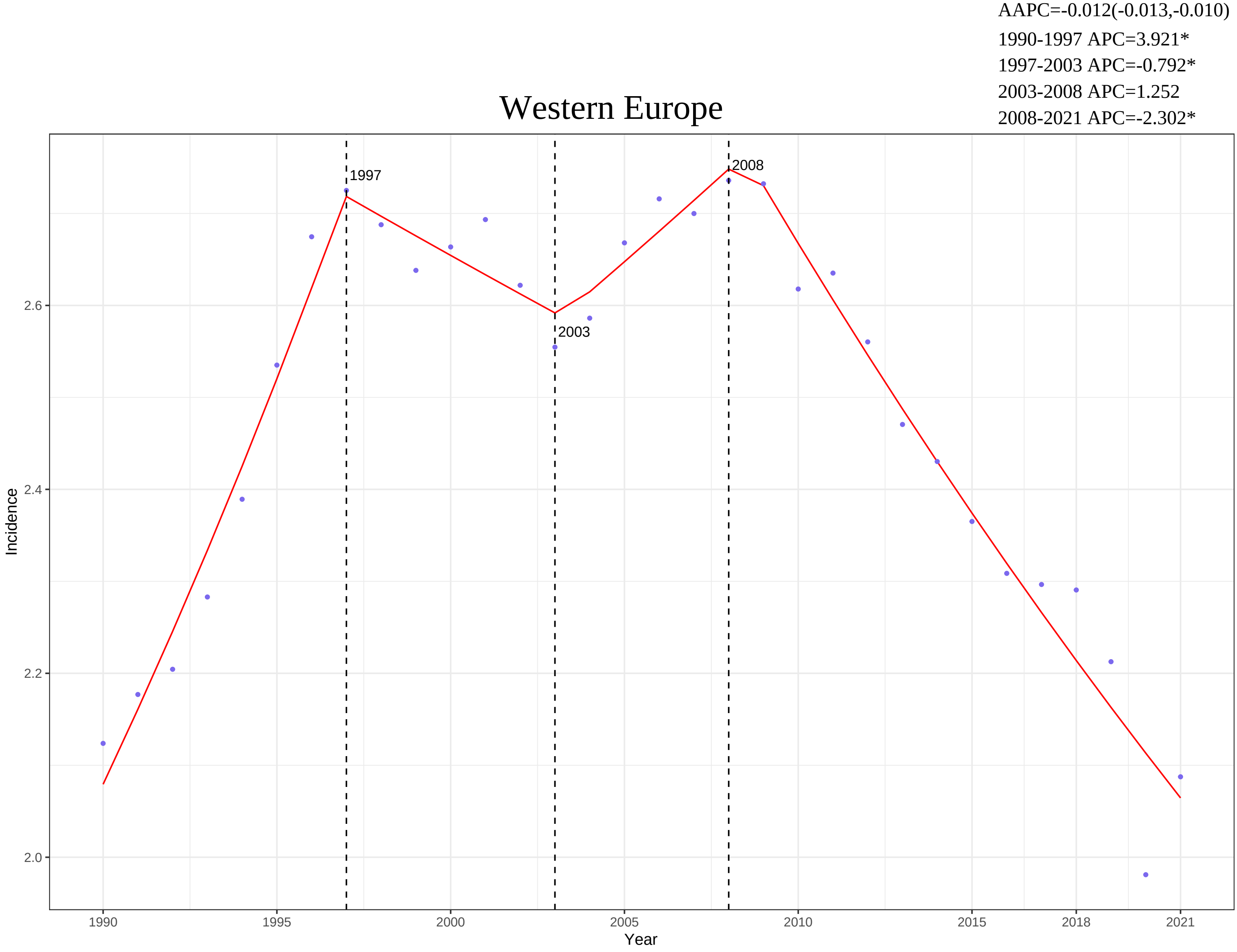
AAPC=-0.012(-0.013,-0.010)
1990-1997 APC=3.921*
1997-2003 APC=-0.792*
2003-2008 APC=1.252
2008-2021 APC=-2.302*
Western Europe
1997
2003
2008
2.6
2.4
2.2
2.0
Incidence
1990
1995
2000
2005
2010
2015
2018
2021
Year

Figure S10

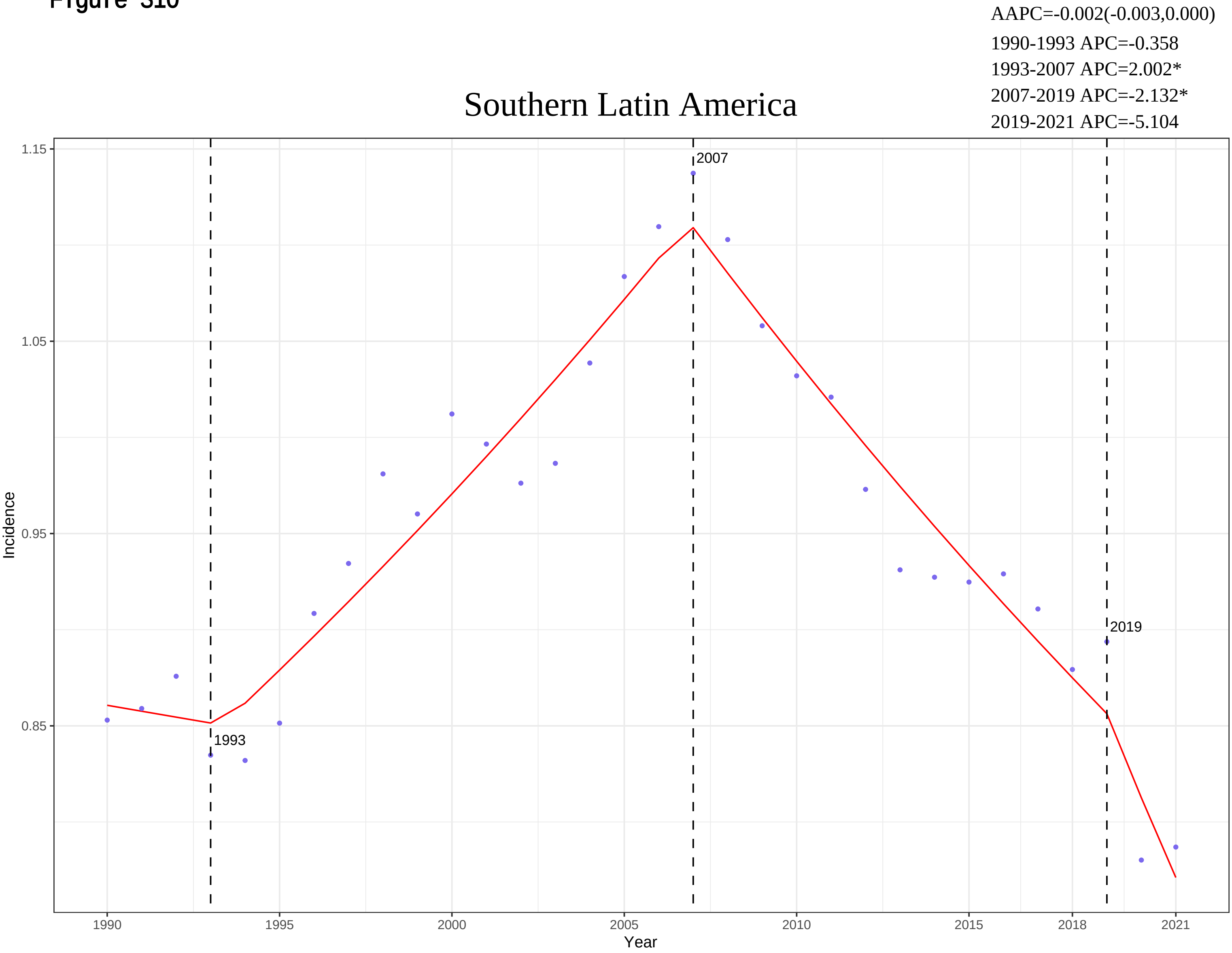
AAPC=-0.002(-0.003,0.000)
1990-1993 APC=-0.358
1993-2007 APC=2.002*
2007-2019 APC=-2.132*
2019-2021 APC=-5.104
Southern Latin America
1.15
1.05
0.95
0.85
Incidence
2007
1993
2019
1990
1995
2000
2005
2010
2015
2018
2021
Year

Figure S11

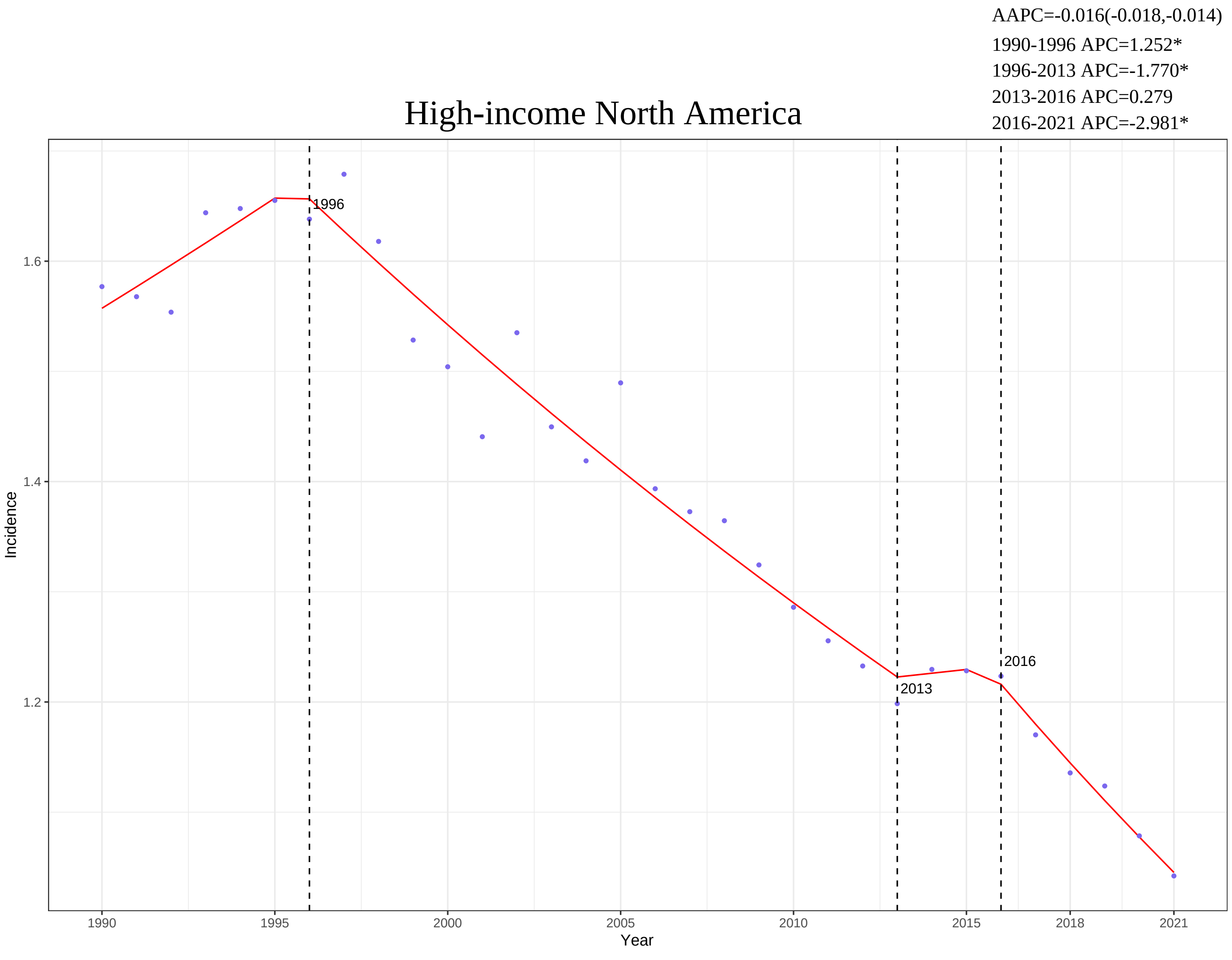
AAPC=-0.016(-0.018,-0.014)
1990-1996 APC=1.252*
1996-2013 APC=-1.770*
2013-2016 APC=0.279
2016-2021 APC=-2.981*
High-income North America
1996
2013
2016
1.6
1.4
1.2
Incidence
1990
1995
2000
2005
2010
2015
2018
2021
Year

Figure S12

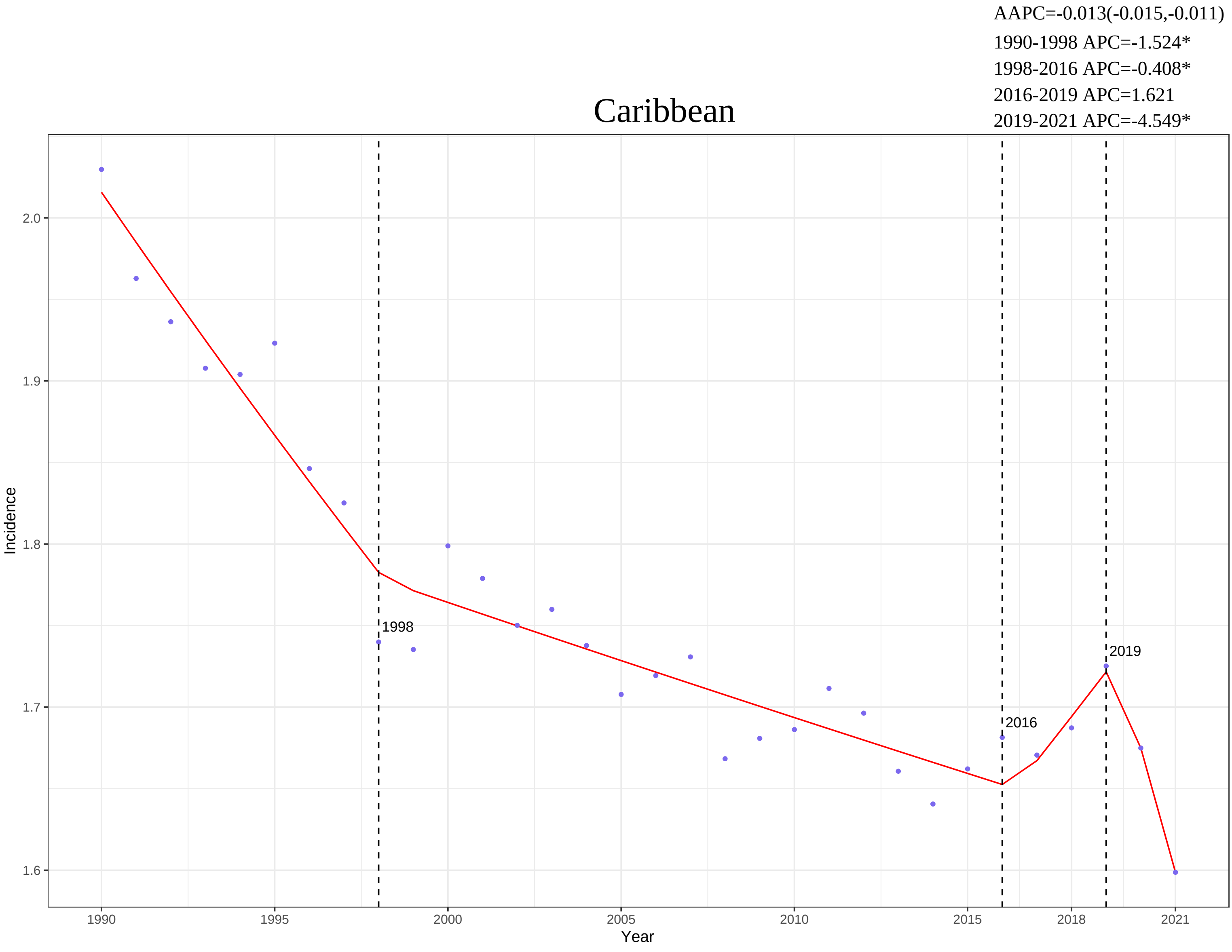
AAPC=-0.013(-0.015,-0.011)
1990-1998 APC=-1.524*
1998-2016 APC=-0.408*
2016-2019 APC=1.621
2019-2021 APC=-4.549*
Caribbean
Incidence
2.0
1.9
1.8
1.7
1.6
1998
2016
2019
1990
1995
2000
2005
2010
2015
2018
2021
Year

Figure S13

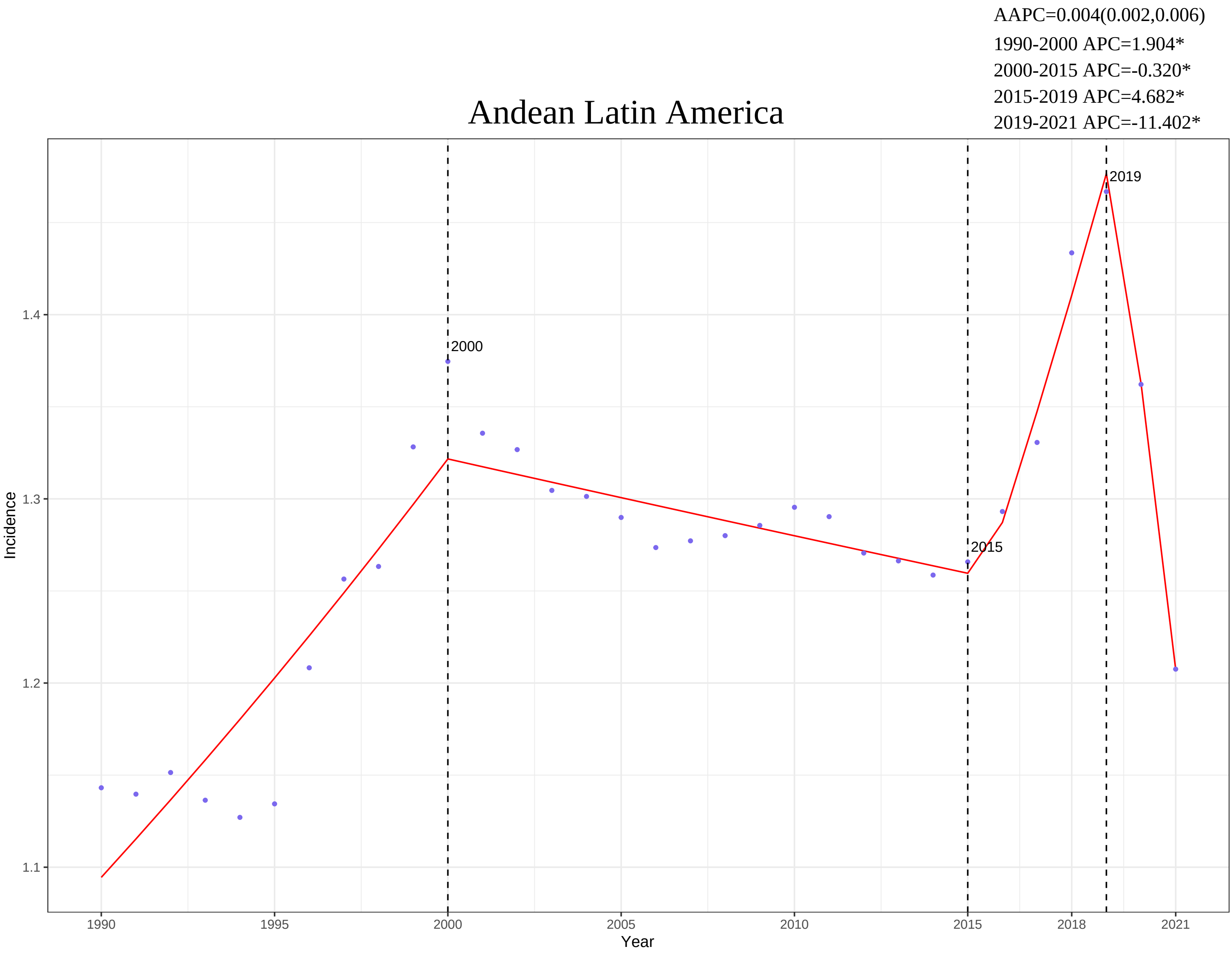
AAPC=0.004(0.002,0.006)
1990-2000 APC=1.904*
2000-2015 APC=-0.320*
2015-2019 APC=4.682*
2019-2021 APC=-11.402*
Andean Latin America
2000
2015
2019
Incidence
1.1
1.2
1.3
1.4
1990
1995
2000
2005
2010
2015
2018
2021
Year

Figure S14

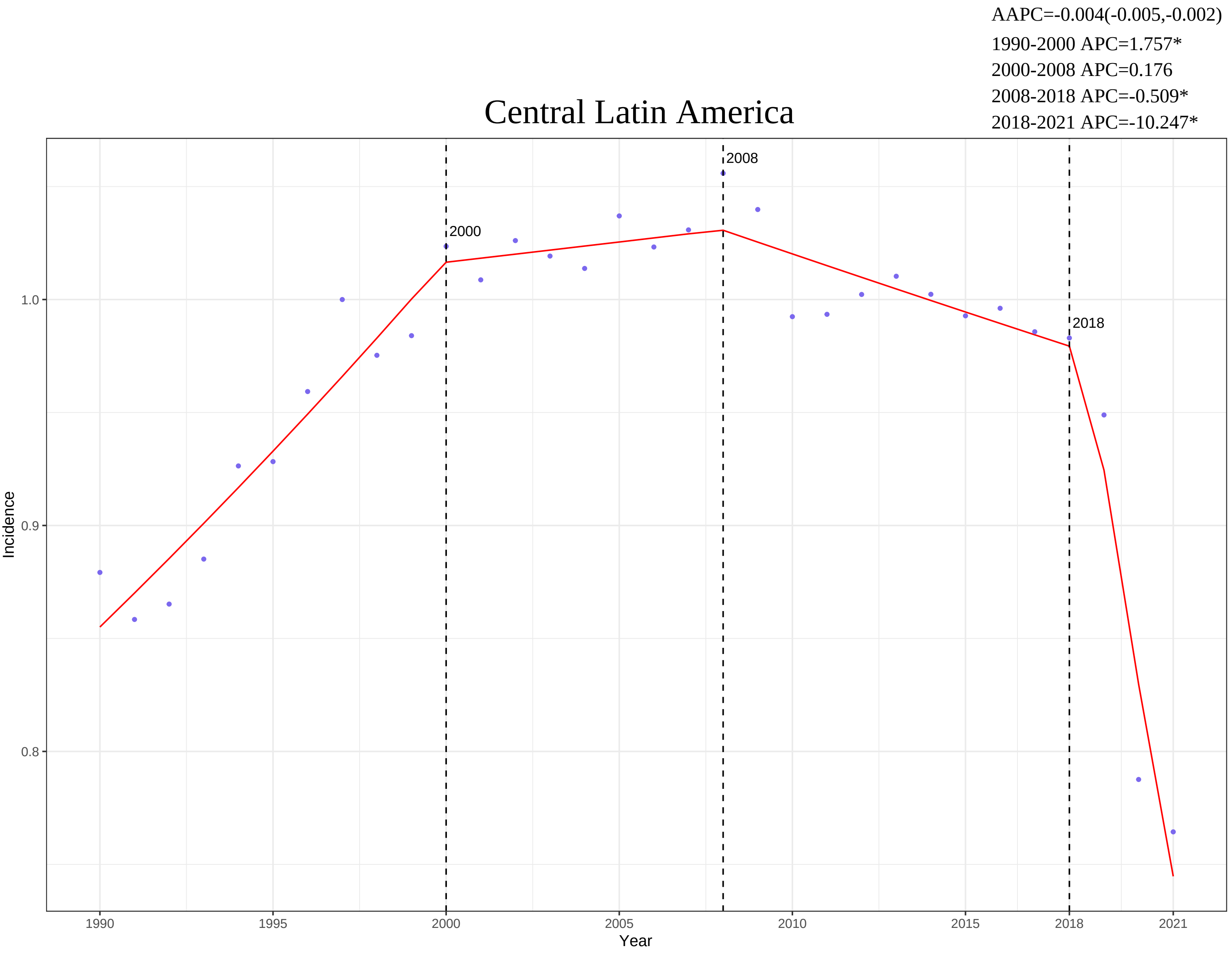
AAPC=-0.004(-0.005,-0.002)
1990-2000 APC=1.757*
2000-2008 APC=0.176
2008-2018 APC=-0.509*
2018-2021 APC=-10.247*
Central Latin America
2000
2008
2018
Incidence
1.0
0.9
0.8
1990
1995
2000
2005
2010
2015
2018
2021
Year

Figure S15

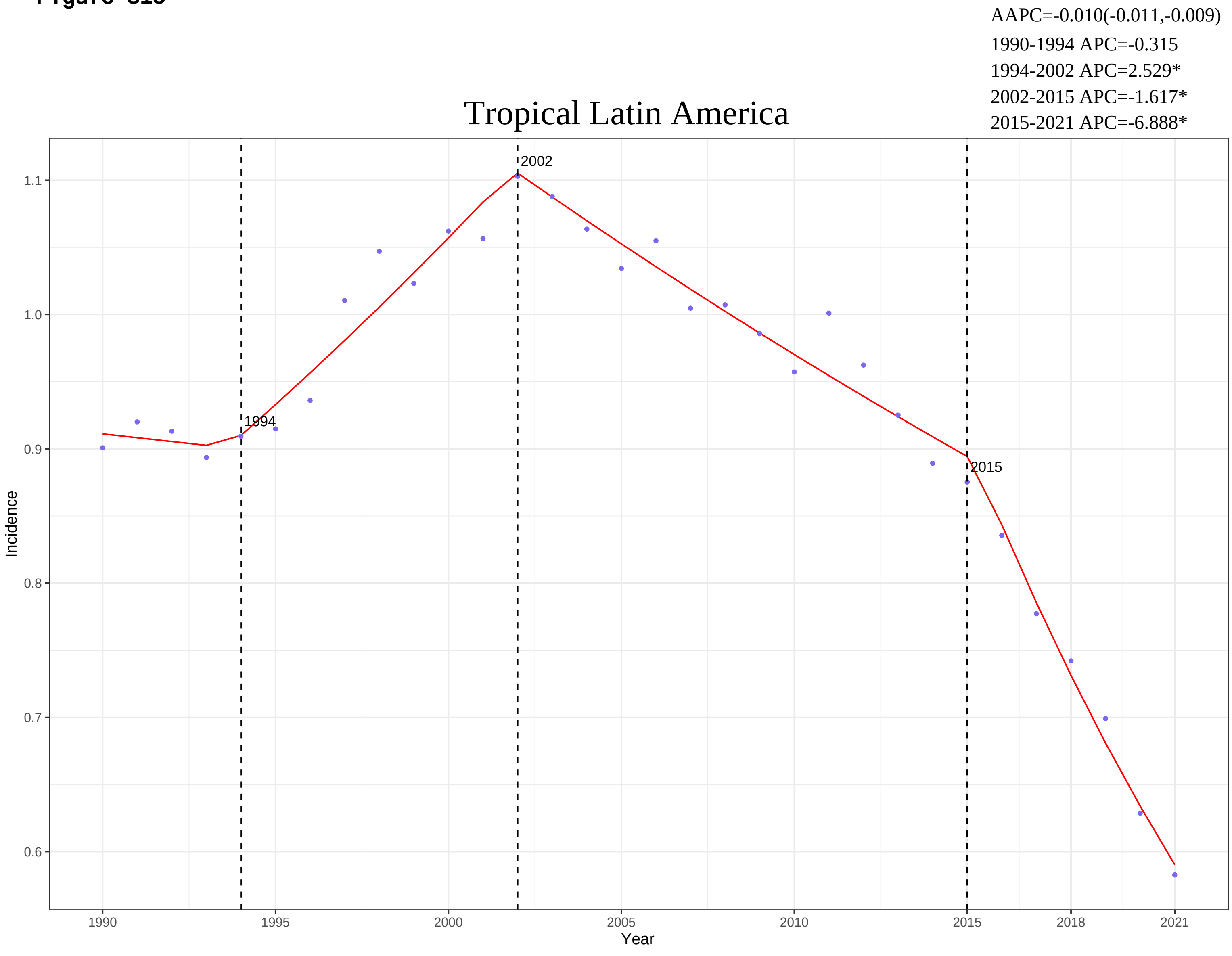
AAPC=-0.010(-0.011,-0.009)
1990-1994 APC=-0.315
1994-2002 APC=2.529*
2002-2015 APC=-1.617*
2015-2021 APC=-6.888*
Tropical Latin America
1994
2002
2015
Incidence
Year
1.1
1.0
0.9
0.8
0.7
0.6
1990
1995
2000
2005
2010
2015
2018
2021

Figure S16

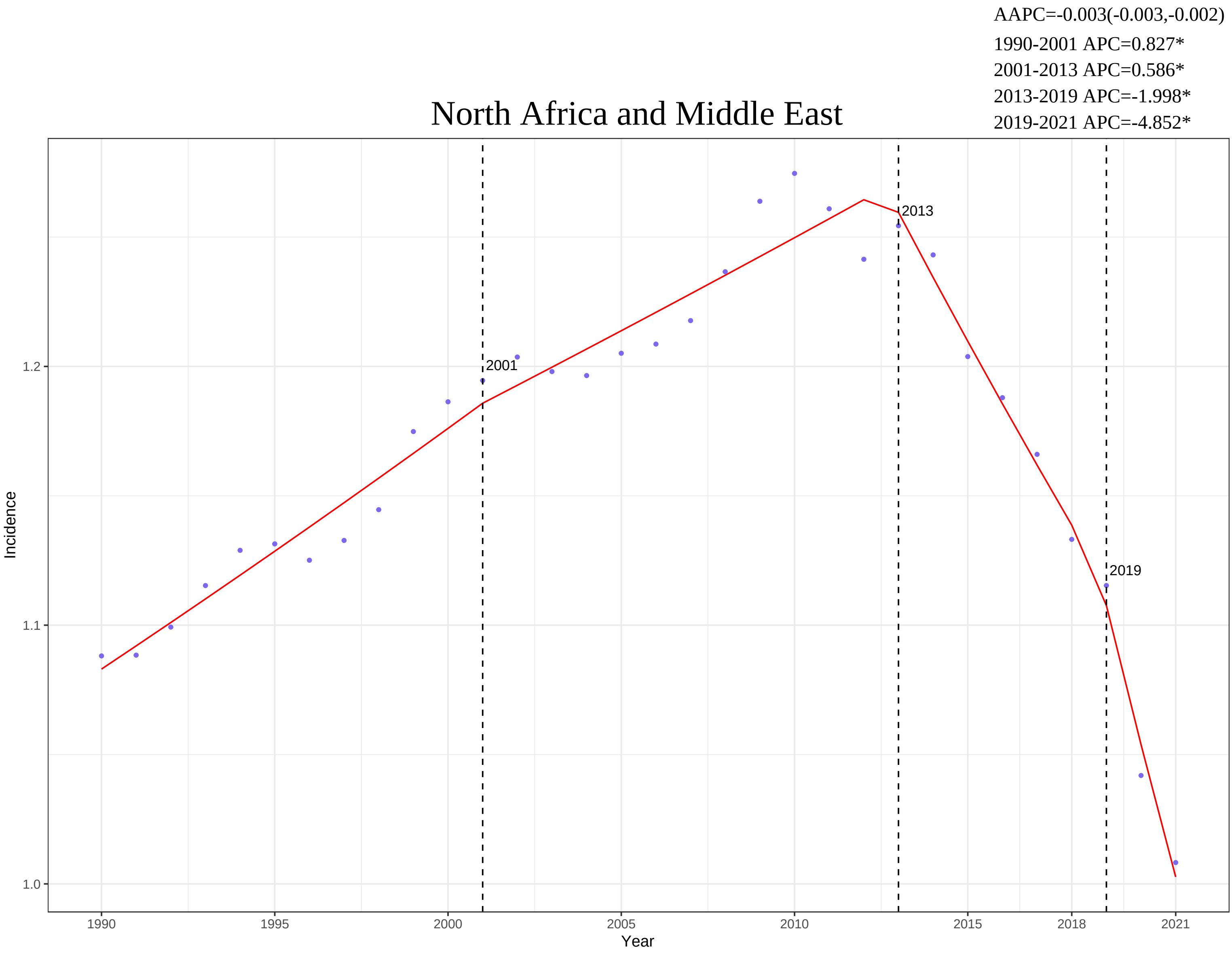
AAPC=-0.003(-0.003,-0.002)
1990-2001 APC=0.827*
2001-2013 APC=0.586*
2013-2019 APC=-1.998*
2019-2021 APC=-4.852*
North Africa and Middle East
2001
2013
2019
Incidence
1.2
1.1
1.0
Year
1990
1995
2000
2005
2010
2015
2018
2021

Figure S17

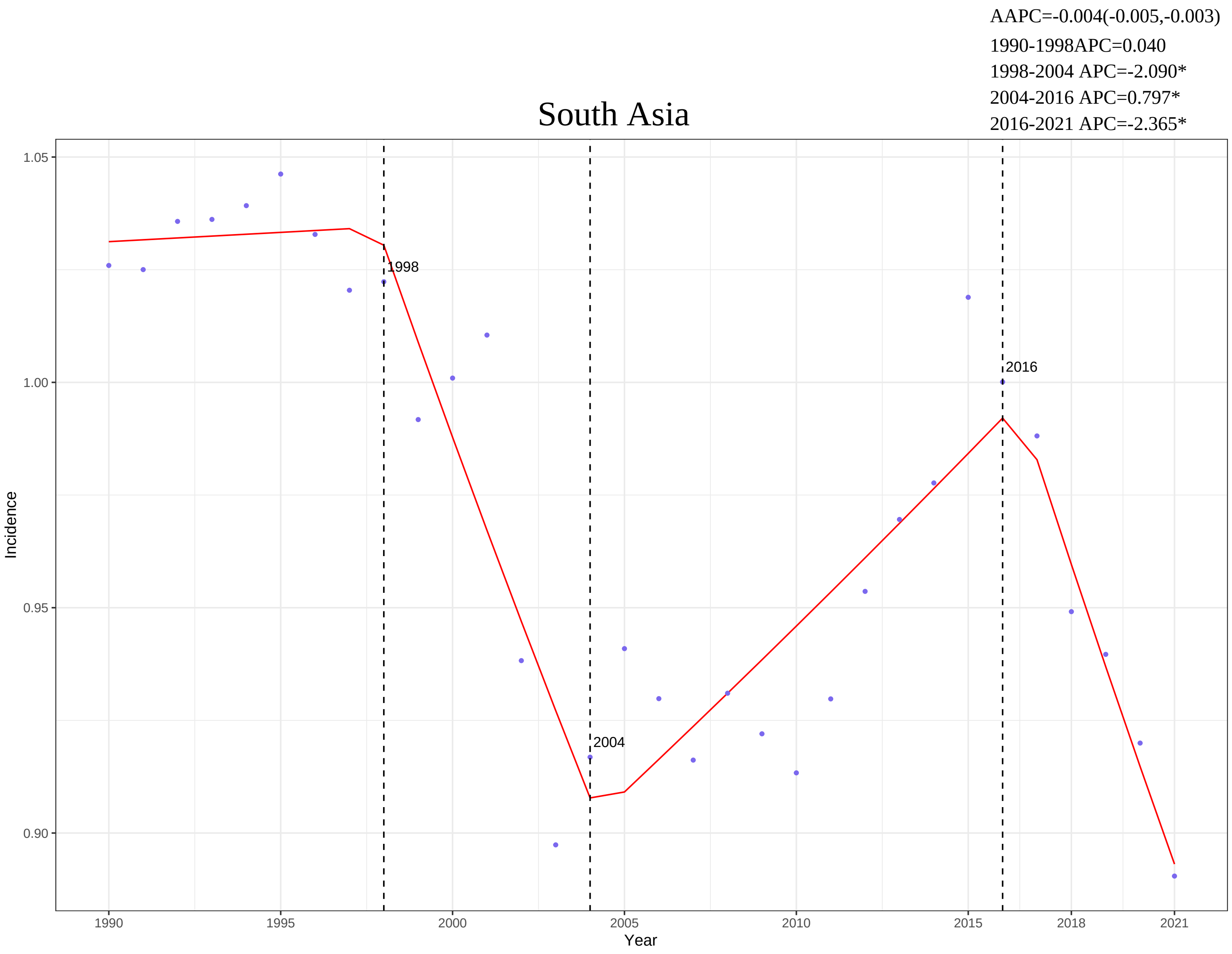
AAPC=-0.004(-0.005,-0.003)
1990-1998APC=0.040
1998-2004 APC=-2.090*
2004-2016 APC=0.797*
2016-2021 APC=-2.365*
South Asia
1.05
1.00
0.95
0.90
Incidence
1998
2004
2016
1990
1995
2000
2005
2010
2015
2018
2021
Year

FigureS18

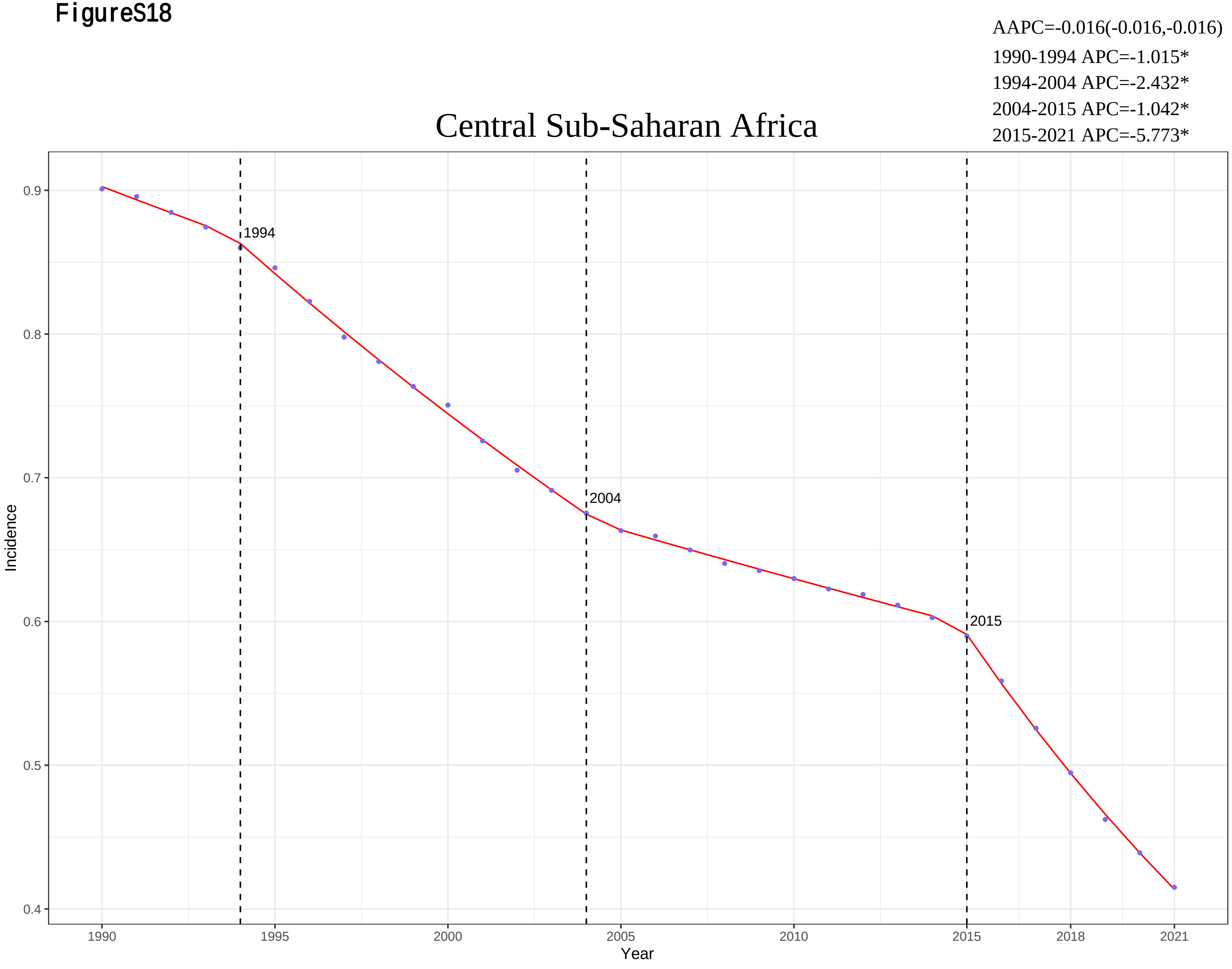
AAPC=-0.016(-0.016,-0.016)
1990-1994 APC=-1.015*
1994-2004 APC=-2.432*
2004-2015 APC=-1.042*
2015-2021 APC=-5.773*
Central Sub-Saharan Africa
Incidence
0.9
0.8
0.7
0.6
0.5
0.4
1994
2004
2015
1990
1995
2000
2005
2010
2015
2018
2021
Year

Figure S19

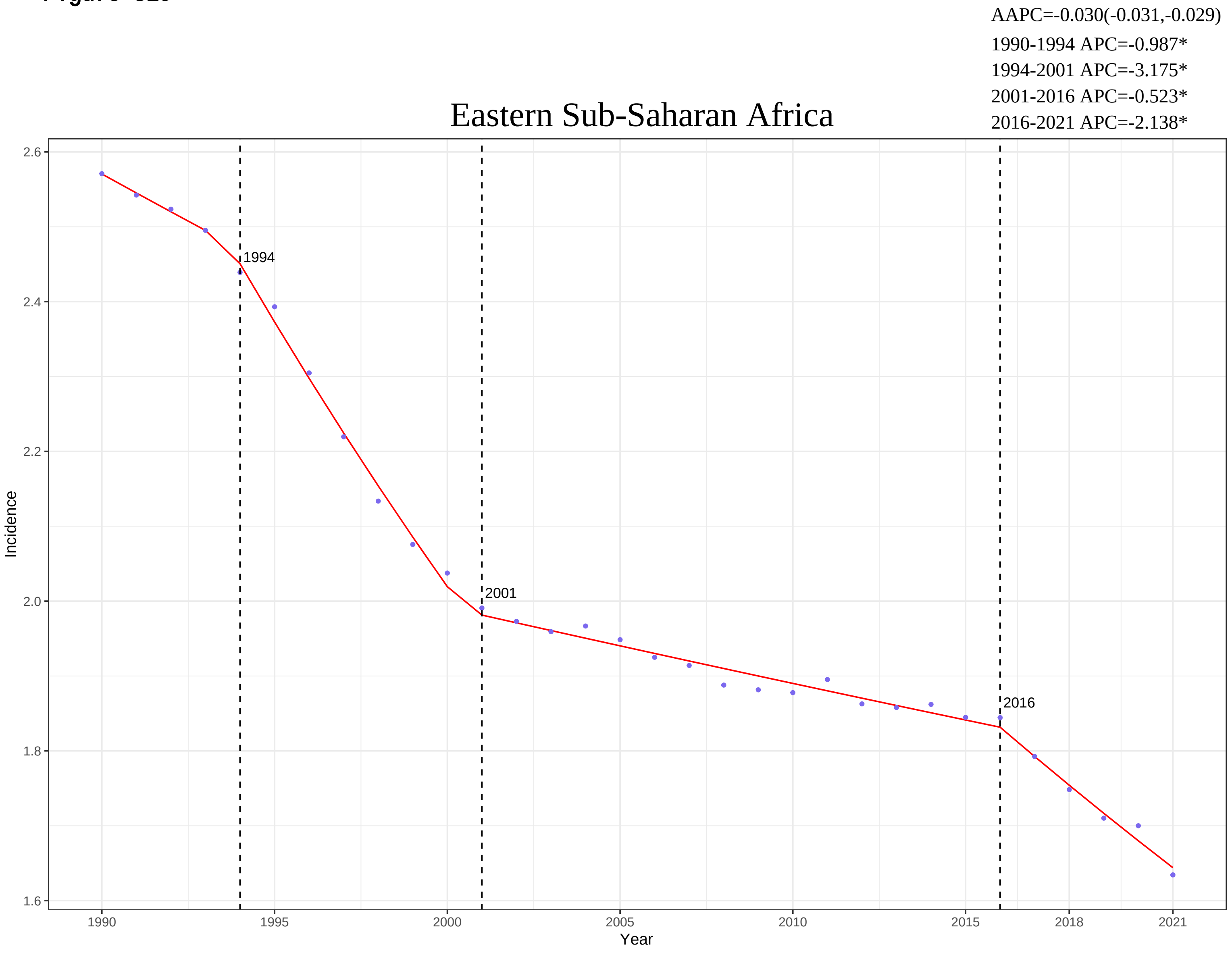
AAPC=-0.030(-0.031,-0.029)
1990-1994 APC=-0.987*
1994-2001 APC=-3.175*
2001-2016 APC=-0.523*
2016-2021 APC=-2.138*
Eastern Sub-Saharan Africa
Incidence
Year
1994
2001
2016
2.6
2.4
2.2
2.0
1.8
1.6
1990
1995
2000
2005
2010
2015
2018
2021

Figure S20

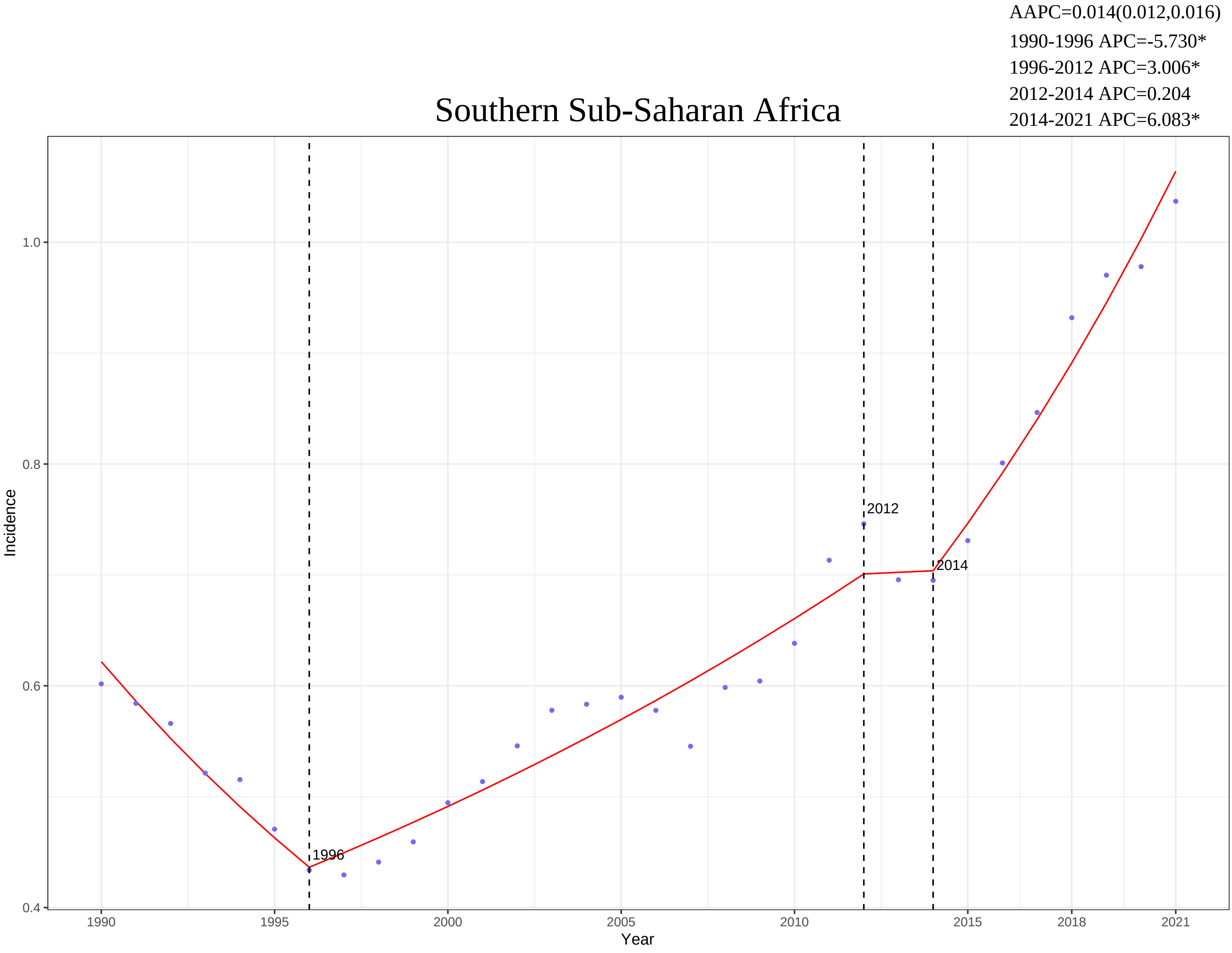
AAPC=0.014(0.012,0.016)
1990-1996 APC=-5.730*
1996-2012 APC=3.006*
2012-2014 APC=0.204
2014-2021 APC=6.083*
Southern Sub-Saharan Africa
Incidence
Year
1996
2012
2014
1.0
0.8
0.6
0.4
1990
1995
2000
2005
2010
2015
2018
2021

Figure S21

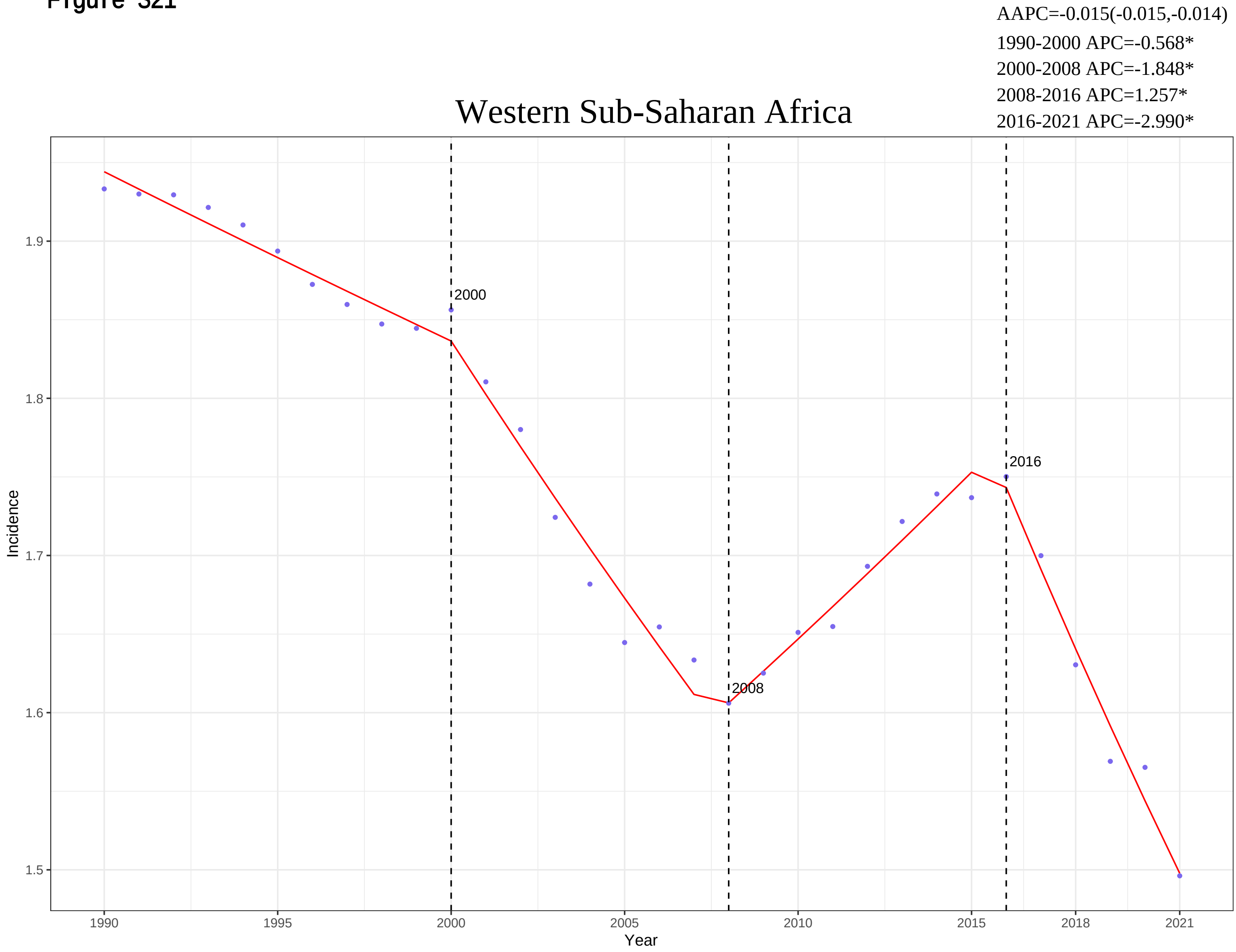
AAPC=-0.015(-0.015,-0.014)
1990-2000 APC=-0.568*
2000-2008 APC=-1.848*
2008-2016 APC=1.257*
2016-2021 APC=-2.990*
Western Sub-Saharan Africa
Incidence
Year
2000
2008
2016
1.9
1.8
1.7
1.6
1.5
1990
1995
2000
2005
2010
2015
2018
2021

Figure S22

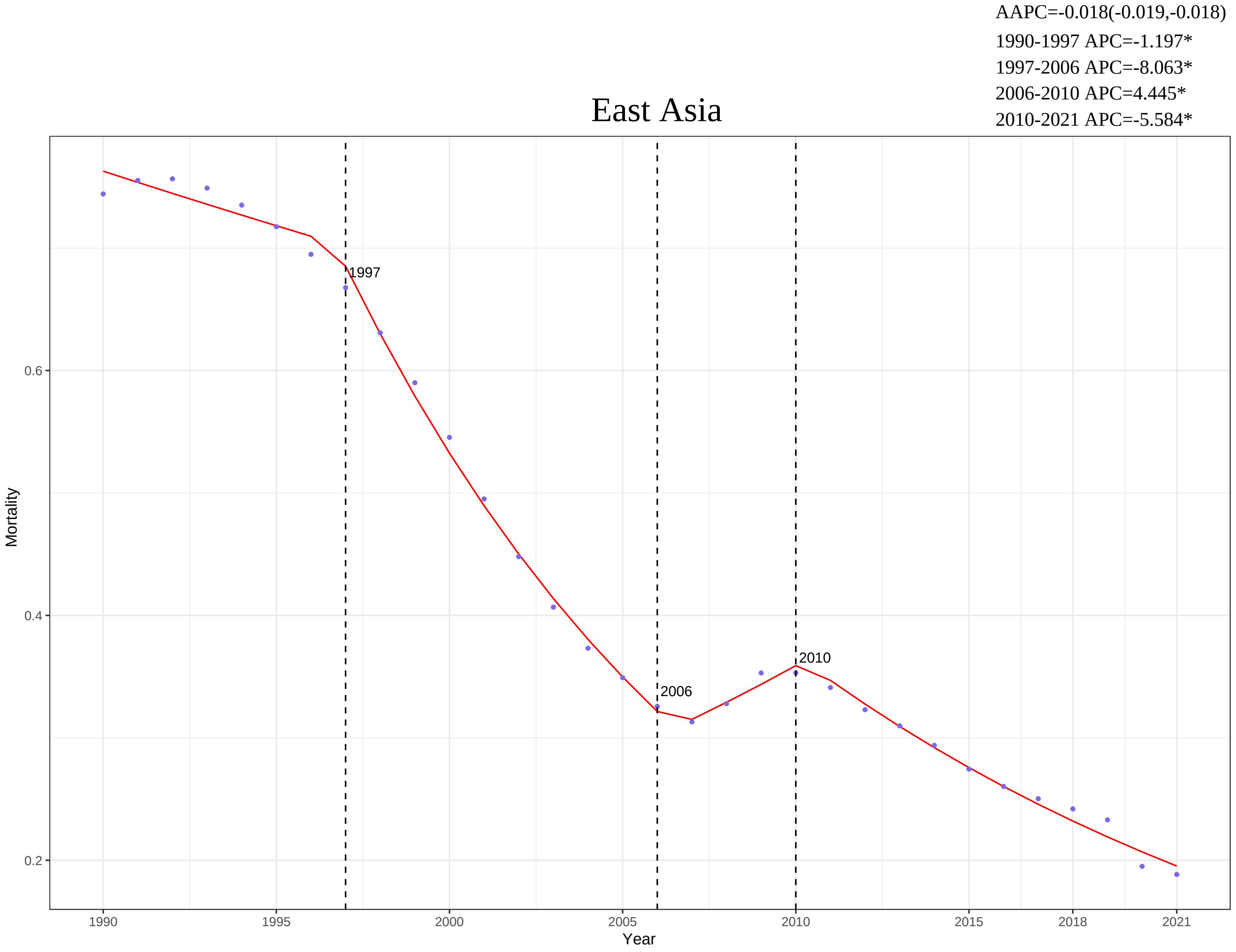
AAPC=-0.018(-0.019,-0.018)
1990-1997 APC=-1.197*
1997-2006 APC=-8.063*
2006-2010 APC=4.445*
2010-2021 APC=-5.584*
East Asia
Mortality
0.6
0.4
0.2
1997
2006
2010
1990
1995
2000
2005
2010
2015
2018
2021
Year

Figure S23

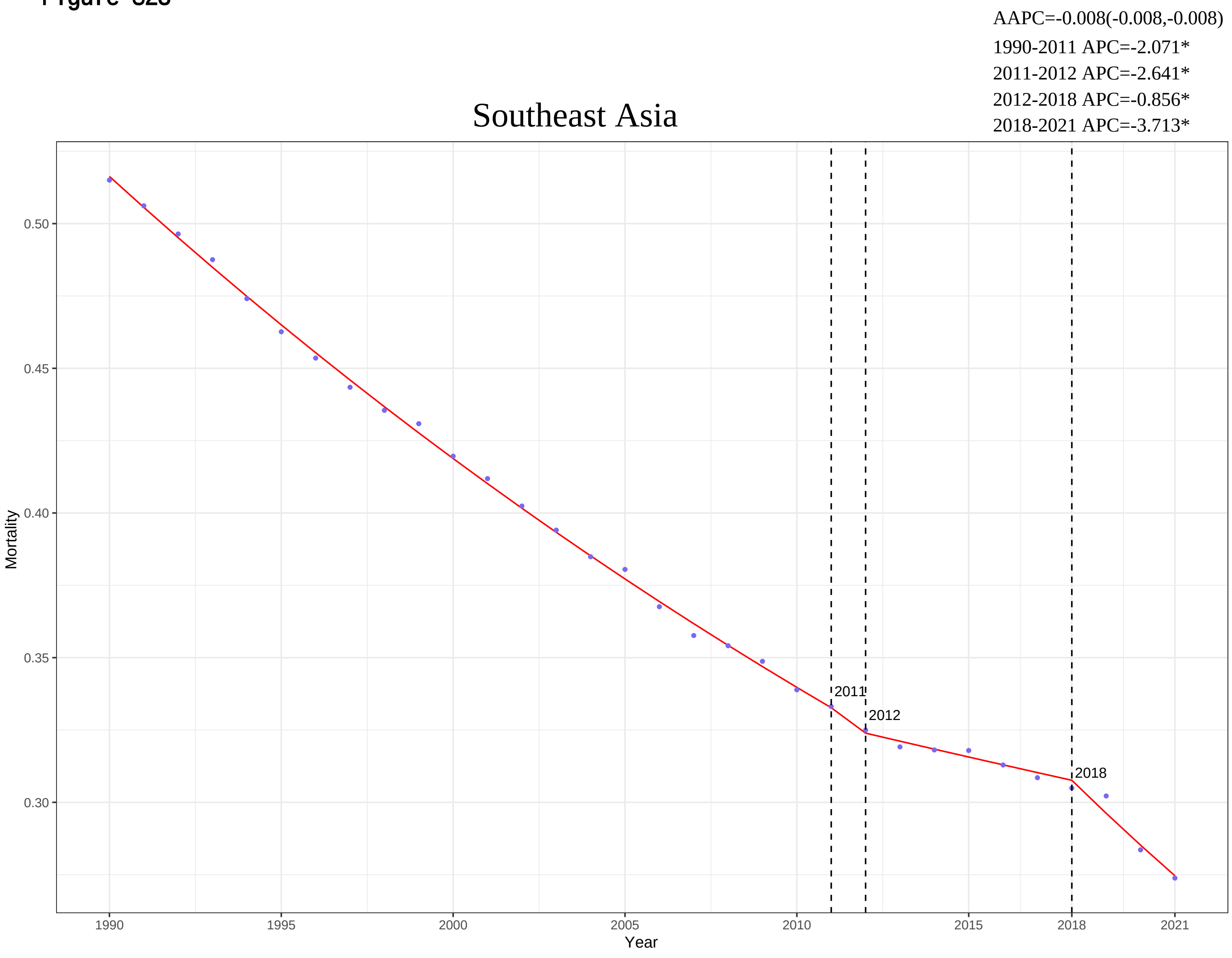
AAPC=-0.008(-0.008,-0.008)
1990-2011 APC=-2.071*
2011-2012 APC=-2.641*
2012-2018 APC=-0.856*
2018-2021 APC=-3.713*
Southeast Asia
Mortality
0.50
0.45
0.40
0.35
0.30
2011
2012
2018
1990
1995
2000
2005
2010
2015
2018
2021
Year

Figure S24

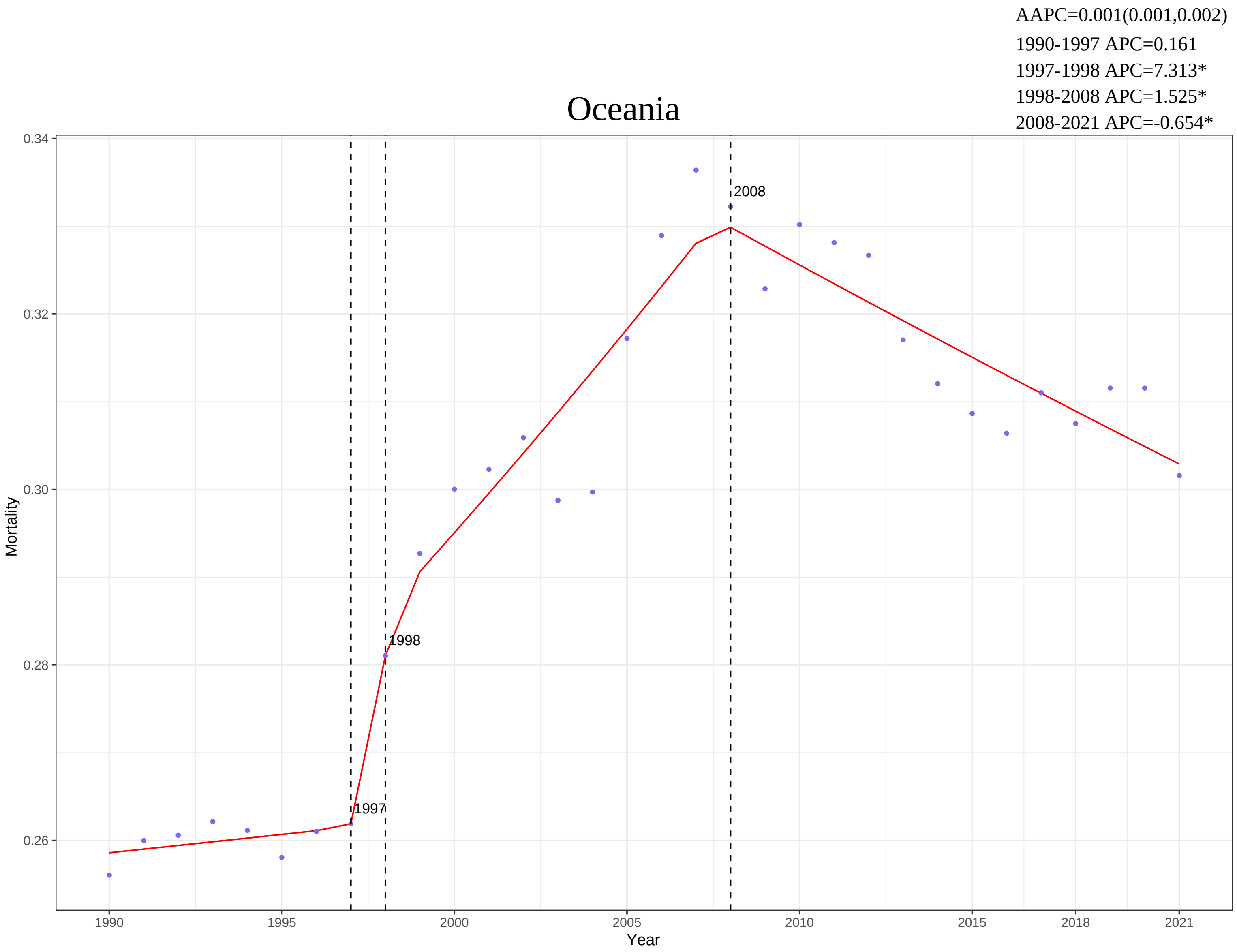
AAPC=0.001(0.001,0.002)
1990-1997 APC=0.161
1997-1998 APC=7.313*
1998-2008 APC=1.525*
2008-2021 APC=-0.654*
Oceania
0.34
0.32
0.30
0.28
0.26
Mortality
1990
1995
2000
2005
2010
2015
2018
2021
Year
1997
1998
2008

Figure S25

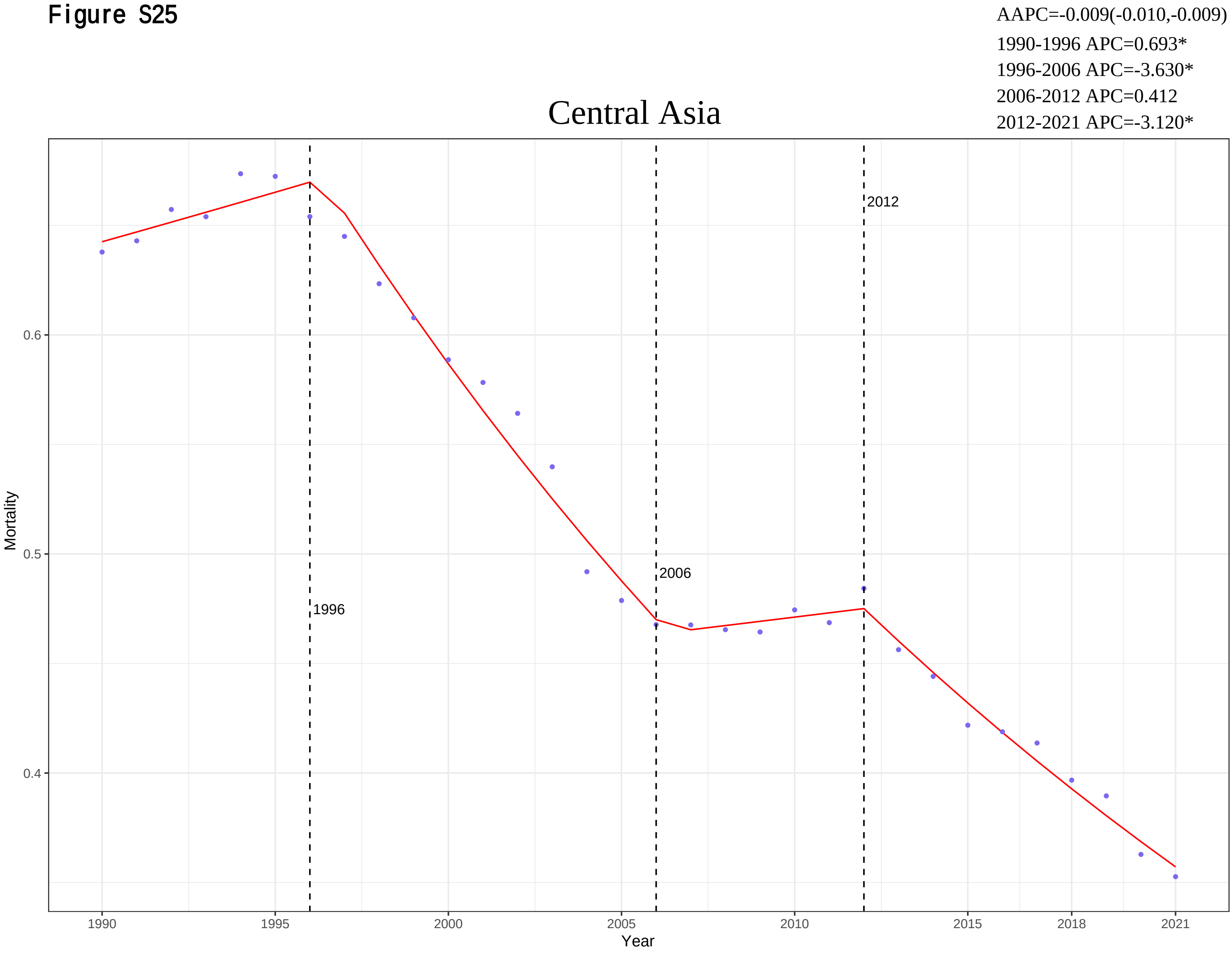
AAPC=-0.009(-0.010,-0.009)
1990-1996 APC=0.693*
1996-2006 APC=-3.630*
2006-2012 APC=0.412
2012-2021 APC=-3.120*
Central Asia
Mortality
0.6
0.5
0.4
1996
2006
2012
1990
1995
2000
2005
2010
2015
2018
2021
Year

Figure S26

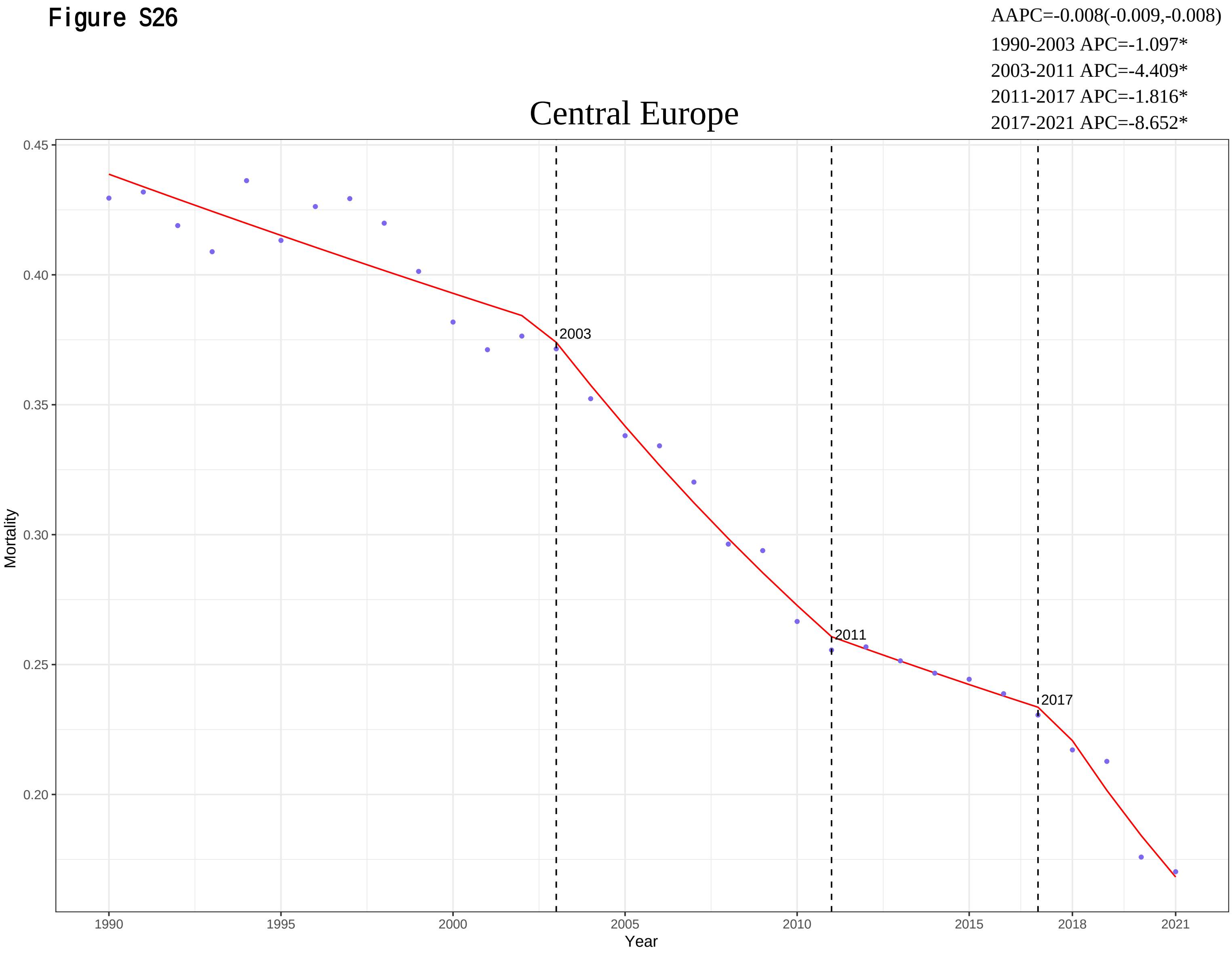

AAPC=-0.008(-0.009,-0.008)
1990-2003 APC=-1.097*
2003-2011 APC=-4.409*
2011-2017 APC=-1.816*
2017-2021 APC=-8.652*
Central Europe
0.45
0.40
0.35
0.30
0.25
0.20
Mortality
2003
2011
2017
1990
1995
2000
2005
2010
2015
2018
2021
Year

Figure S27

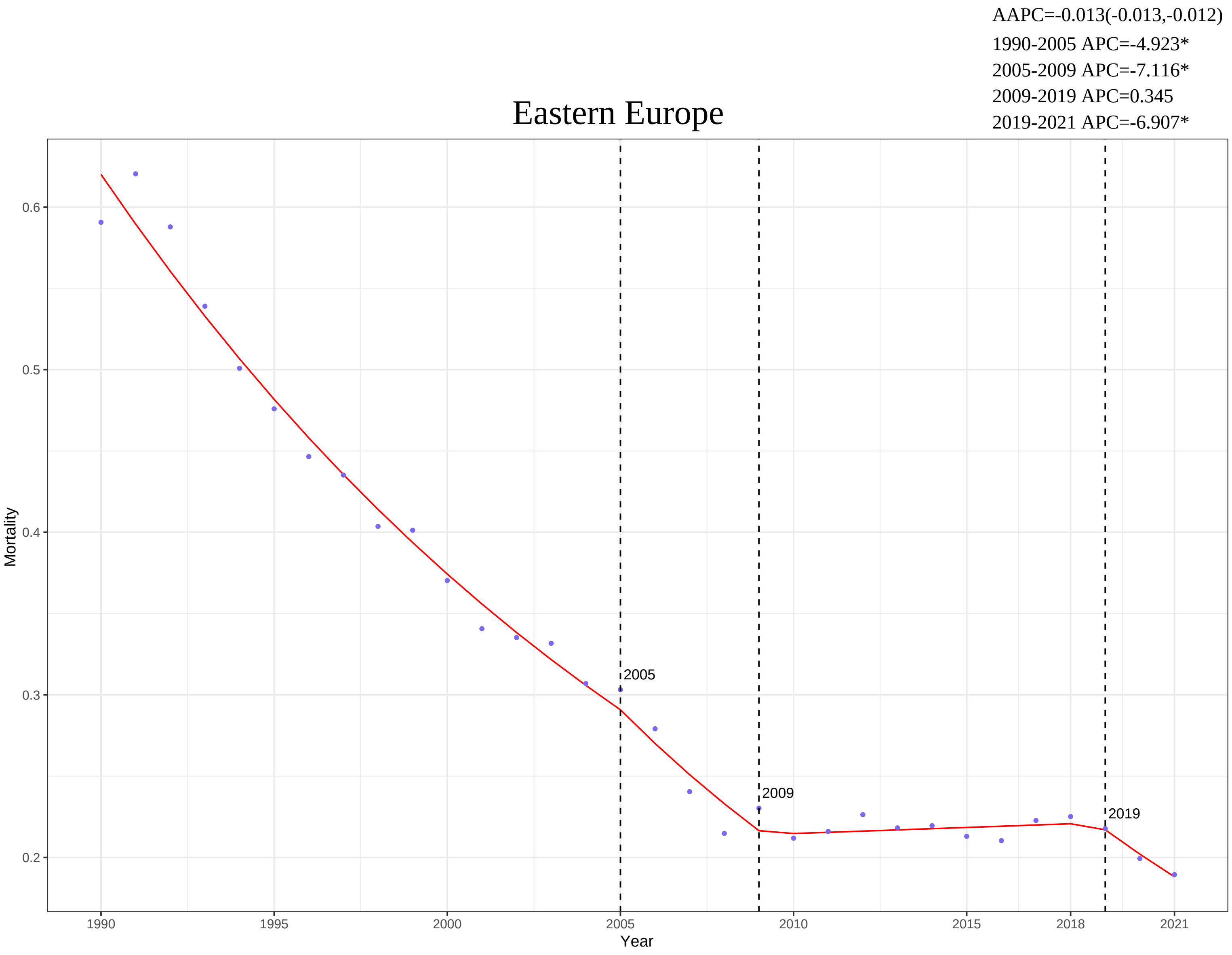
AAPC=-0.013(-0.013,-0.012)
1990-2005 APC=-4.923*
2005-2009 APC=-7.116*
2009-2019 APC=0.345
2019-2021 APC=-6.907*
Eastern Europe
Mortality
0.6
0.5
0.4
0.3
0.2
2005
2009
2019
1990
1995
2000
2005
2010
2015
2018
2021
Year

Figure S28

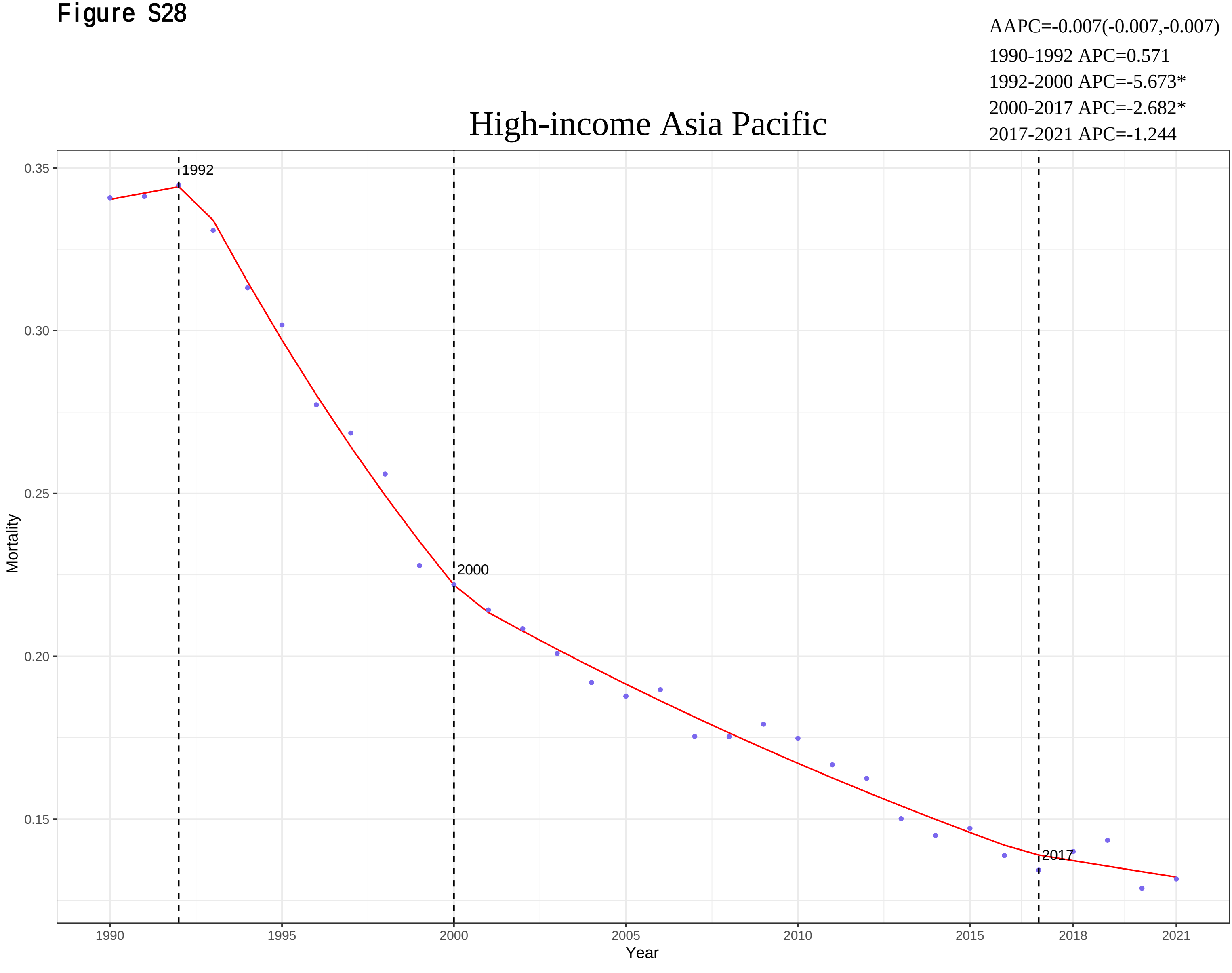
AAPC=-0.007(-0.007,-0.007)
1990-1992 APC=0.571
1992-2000 APC=-5.673*
2000-2017 APC=-2.682*
2017-2021 APC=-1.244
High-income Asia Pacific
Mortality
Year
0.35
0.30
0.25
0.20
0.15
1990
1995
2000
2005
2010
2015
2018
2021
1992
2000
2017

Figure S29

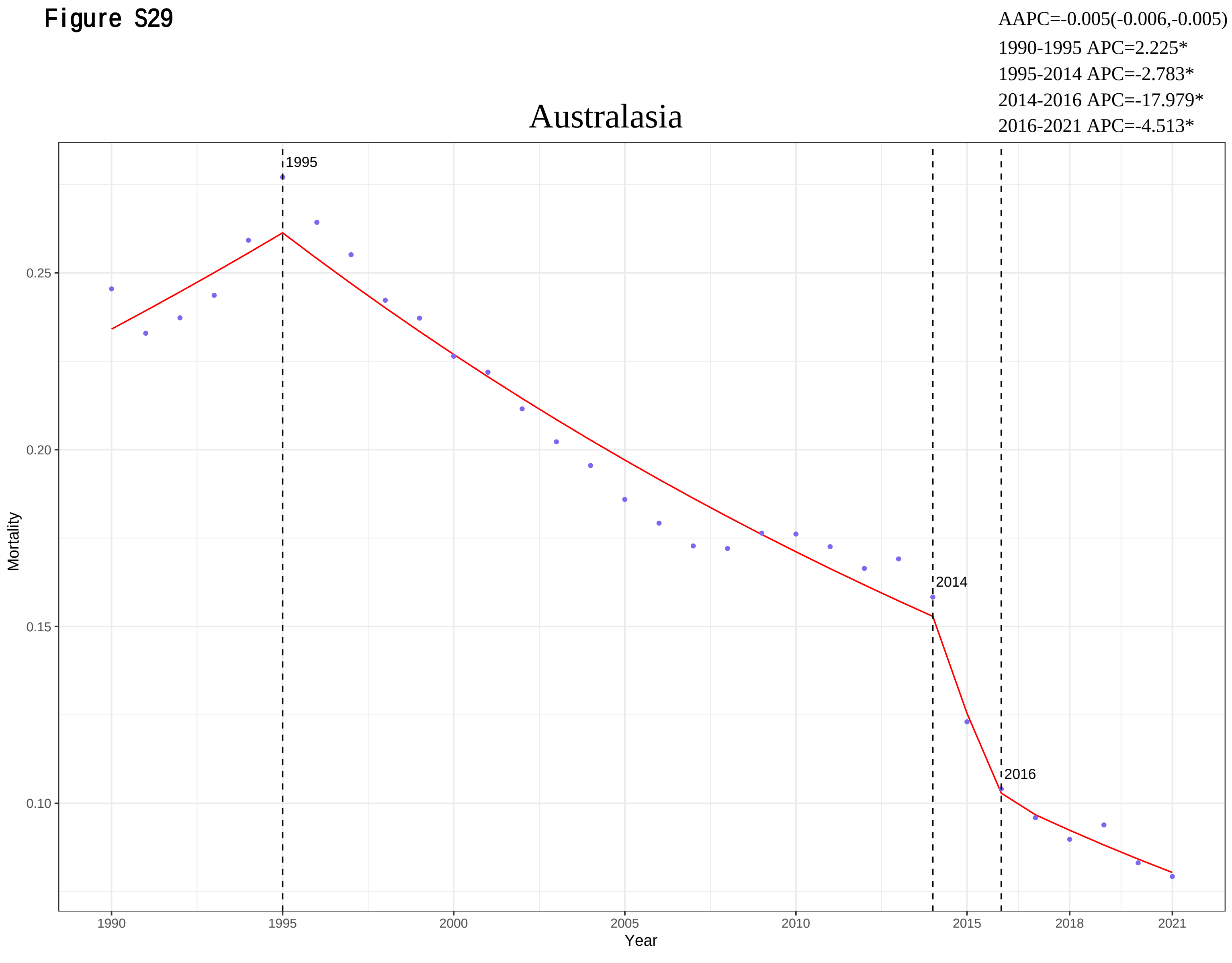
AAPC=-0.005(-0.006,-0.005)
1990-1995 APC=2.225*
1995-2014 APC=-2.783*
2014-2016 APC=-17.979*
2016-2021 APC=-4.513*
Australasia
Mortality
0.25
0.20
0.15
0.10
1995
2014
2016
1990
1995
2000
2005
2010
2015
2018
2021
Year

Figure S30

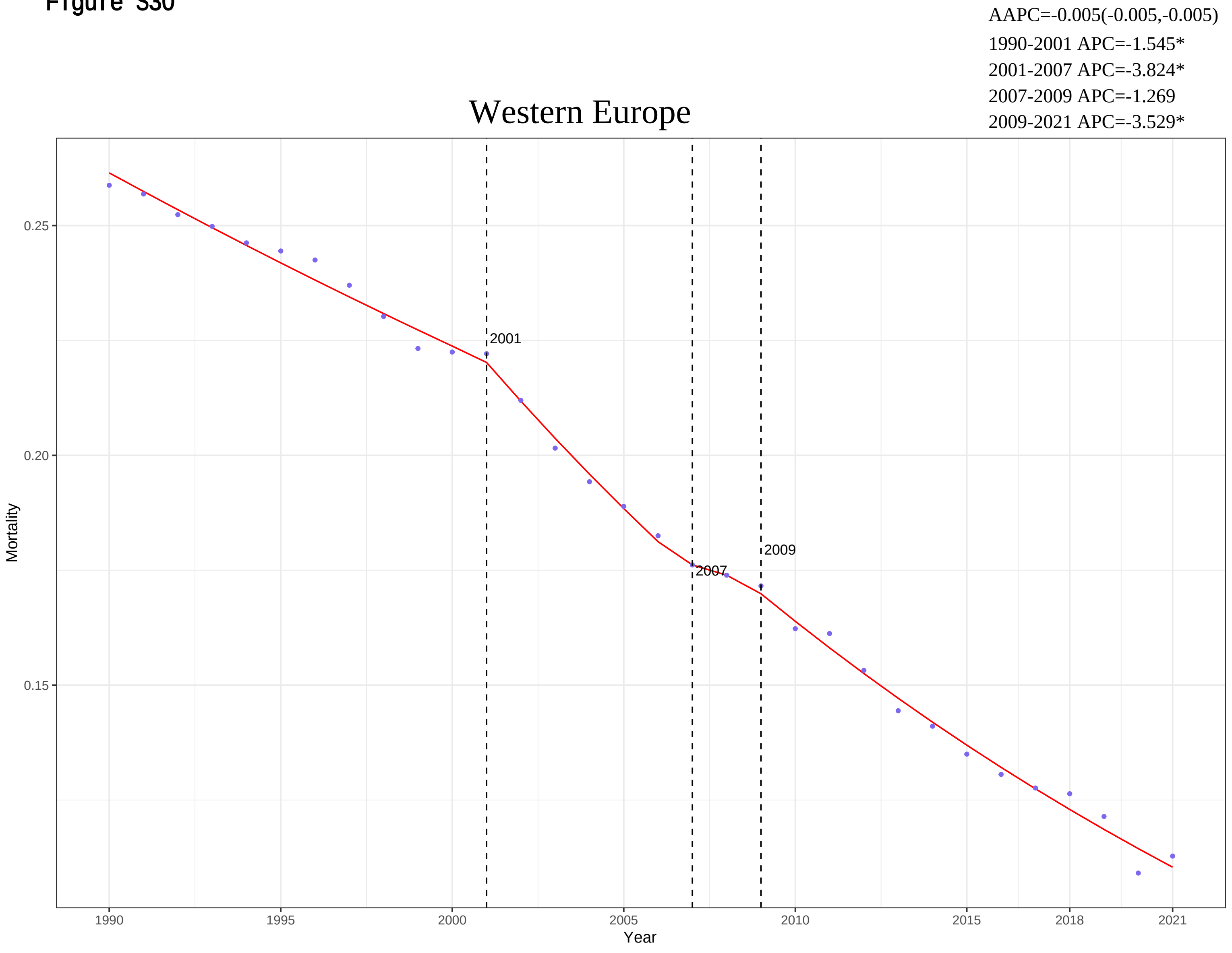
AAPC=-0.005(-0.005,-0.005)
1990-2001 APC=-1.545*
2001-2007 APC=-3.824*
2007-2009 APC=-1.269
2009-2021 APC=-3.529*
Western Europe
2001
2007
2009
0.25
0.20
0.15
Mortality
1990
1995
2000
2005
2010
2015
2018
2021
Year

Figure S31

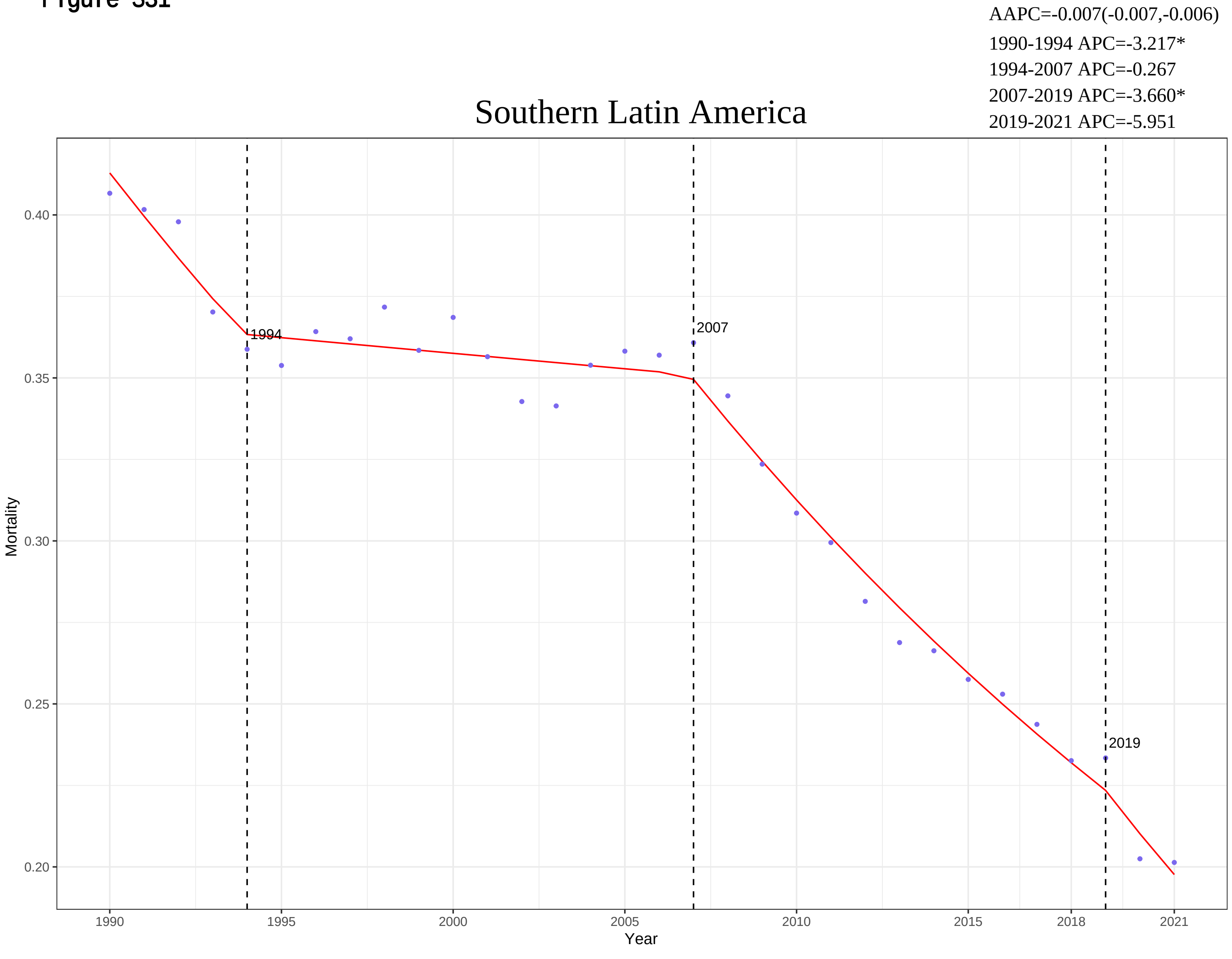
AAPC=-0.007(-0.007,-0.006)
1990-1994 APC=-3.217*
1994-2007 APC=-0.267
2007-2019 APC=-3.660*
2019-2021 APC=-5.951
Southern Latin America
0.40
0.35
0.30
0.25
0.20
Mortality
1994
2007
2019
1990
1995
2000
2005
2010
2015
2018
2021
Year

Figure S32

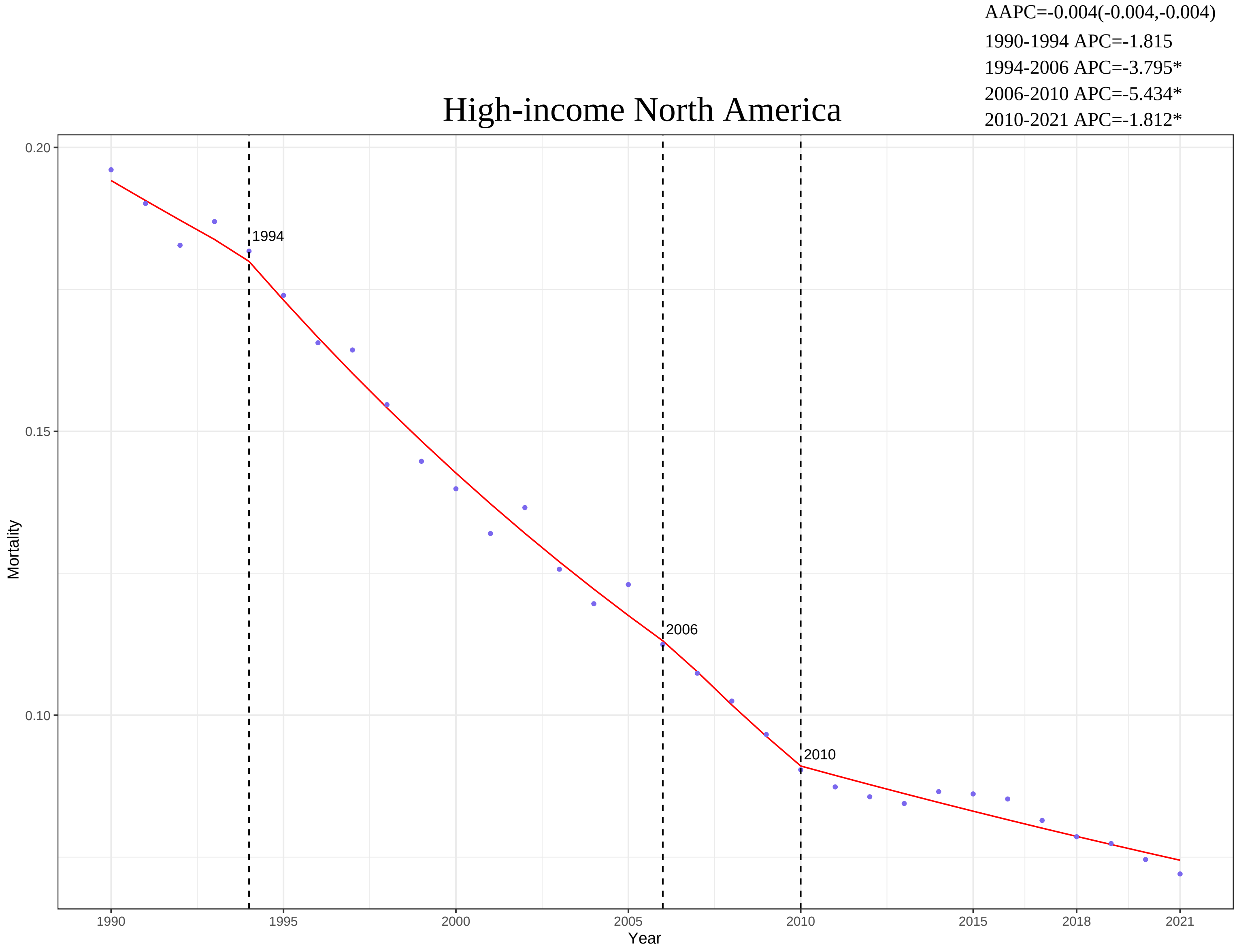

AAPC=-0.004(-0.004,-0.004)
1990-1994 APC=-1.815
1994-2006 APC=-3.795*
2006-2010 APC=-5.434*
2010-2021 APC=-1.812*
High-income North America
Mortality
0.20
0.15
0.10
1994
2006
2010
1990
1995
2000
2005
2010
2015
2018
2021
Year

Figure S33

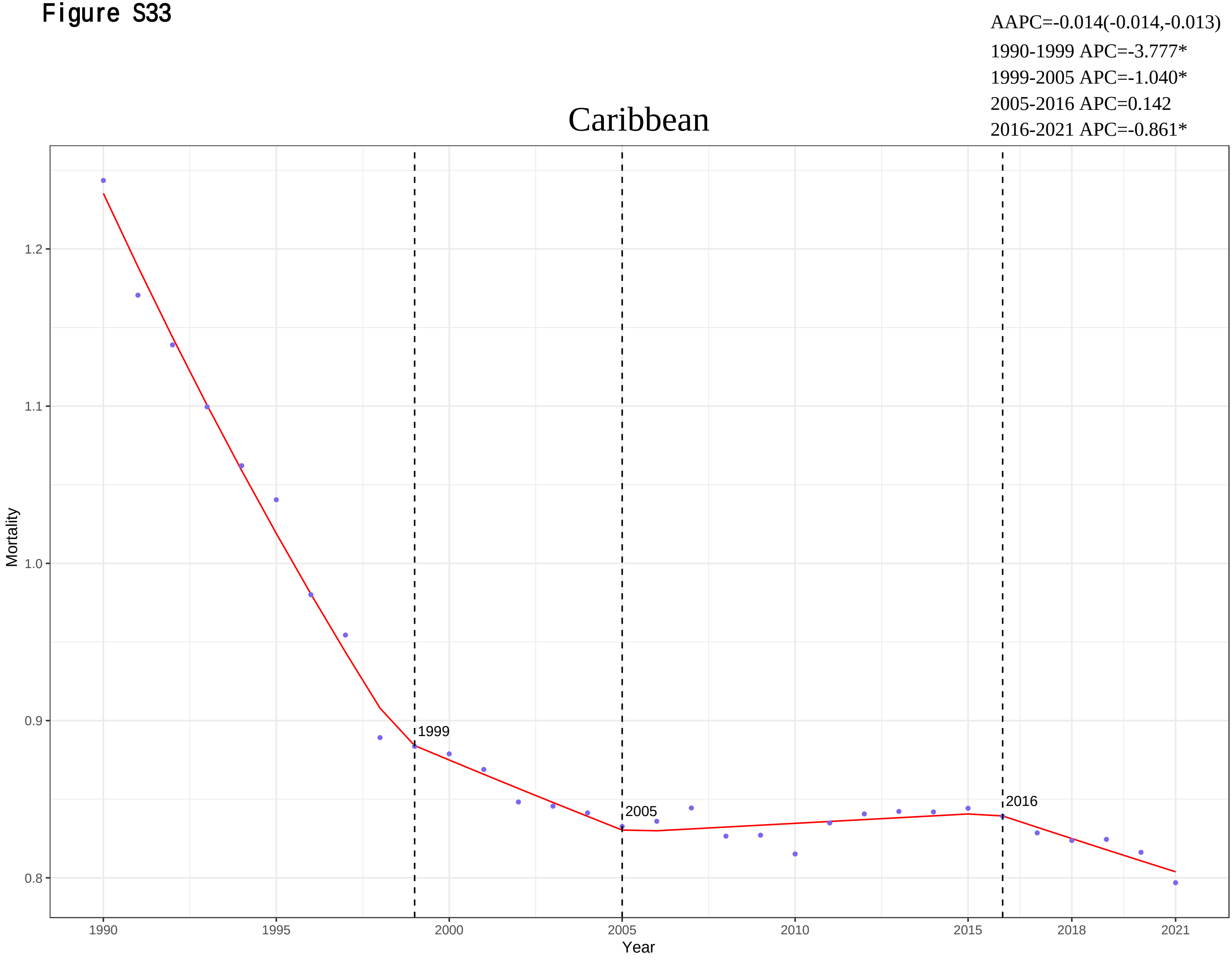
AAPC=-0.014(-0.014,-0.013)
1990-1999 APC=-3.777*
1999-2005 APC=-1.040*
2005-2016 APC=0.142
2016-2021 APC=-0.861*
Caribbean
Mortality
1.2
1.1
1.0
0.9
0.8
1999
2005
2016
1990
1995
2000
2005
2010
2015
2018
2021
Year

Figure S34

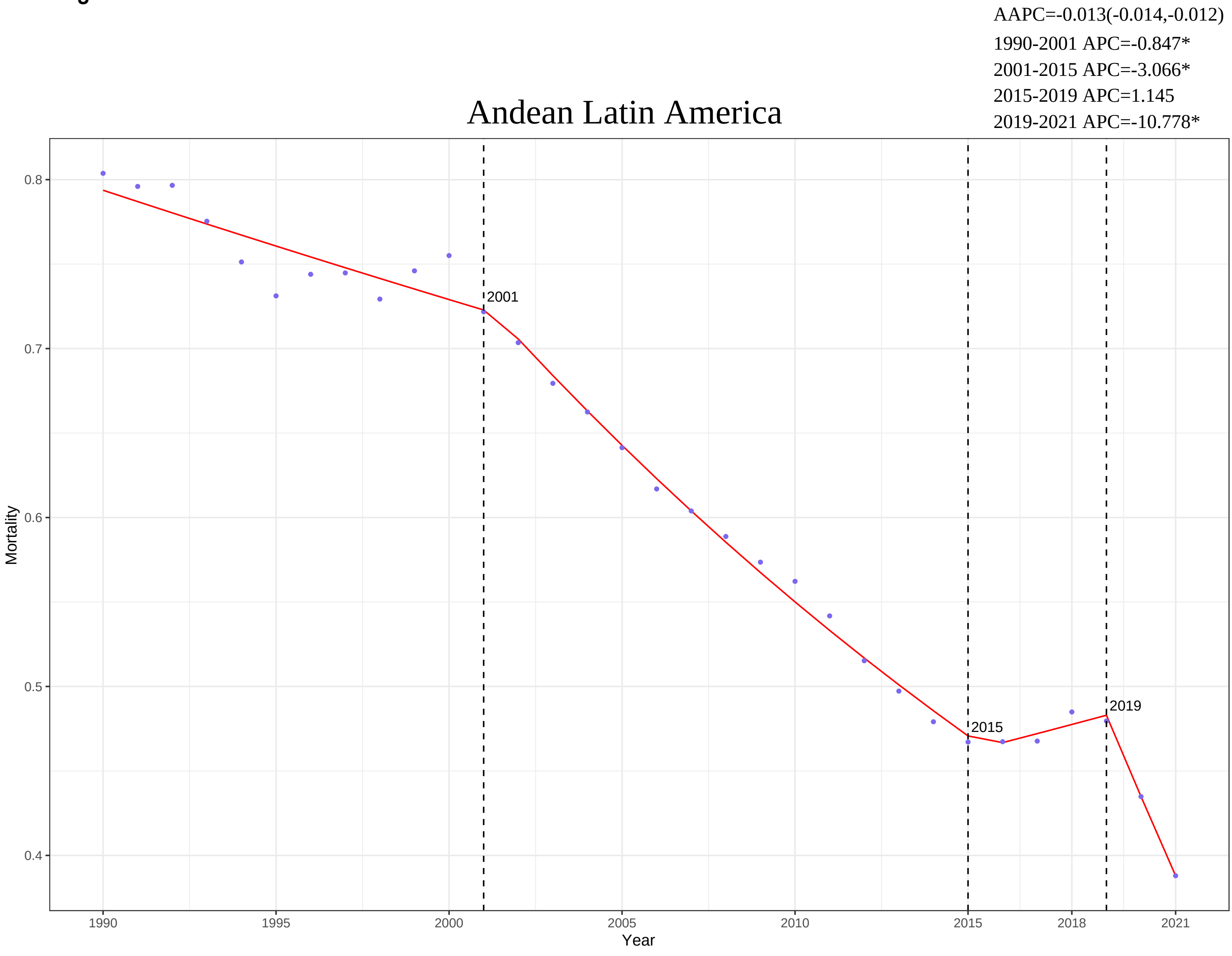
AAPC=-0.013(-0.014,-0.012)
1990-2001 APC=-0.847*
2001-2015 APC=-3.066*
2015-2019 APC=1.145
2019-2021 APC=-10.778*
Andean Latin America
Mortality
0.8
0.7
0.6
0.5
0.4
2001
2015
2019
1990
1995
2000
2005
2010
2015
2018
2021
Year

Figure S35

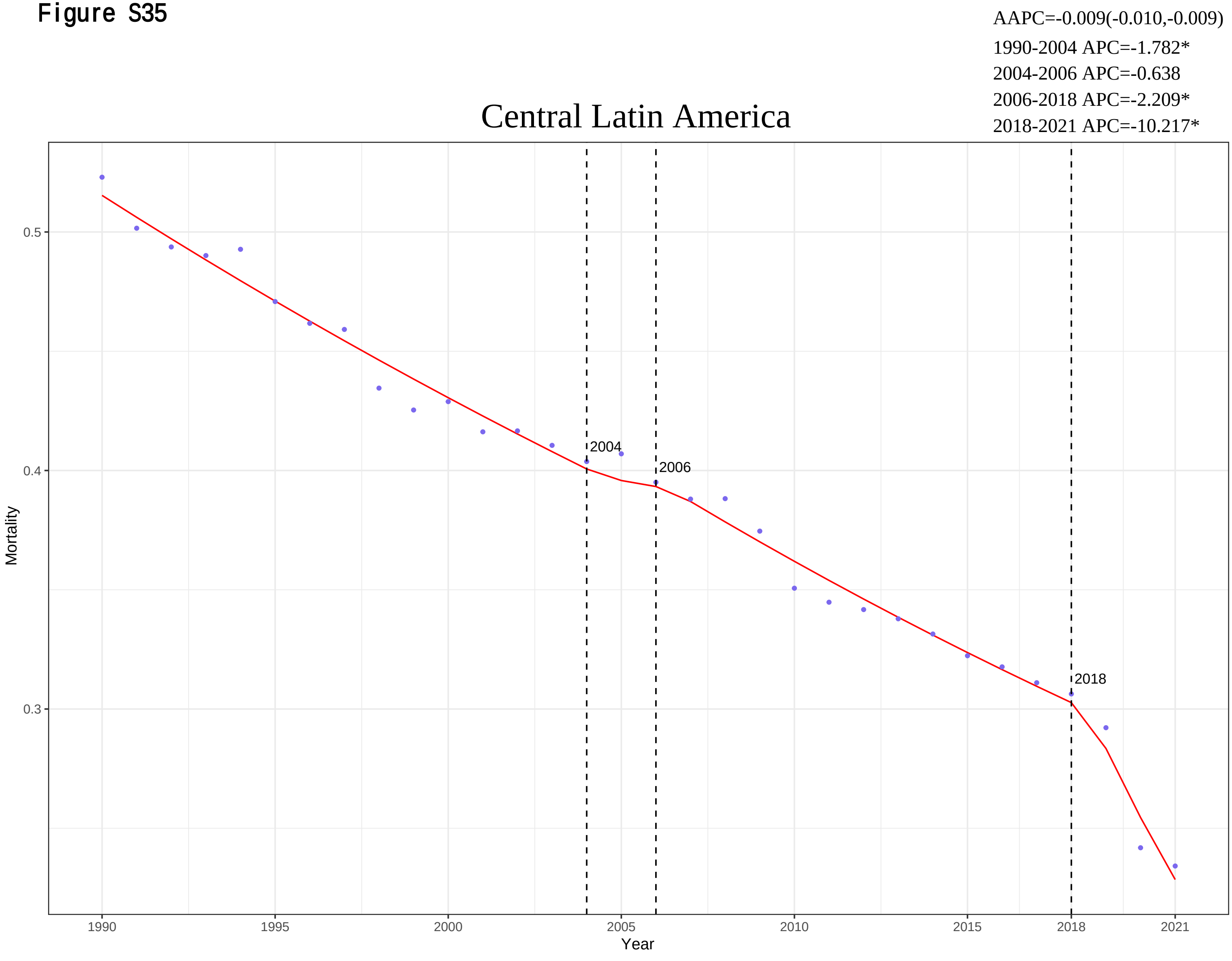
AAPC=-0.009(-0.010,-0.009)
1990-2004 APC=-1.782*
2004-2006 APC=-0.638
2006-2018 APC=-2.209*
2018-2021 APC=-10.217*
Central Latin America
Mortality
0.5
0.4
0.3
2004
2006
2018
1990
1995
2000
2005
2010
2015
2018
2021
Year

Figure S36

AAPC=-0.011(-0.012,-0.011)
1990-1993 APC=-2.175*
1993-2005 APC=-0.501*
2005-2015 APC=-3.775*
2015-2021 APC=-8.251*

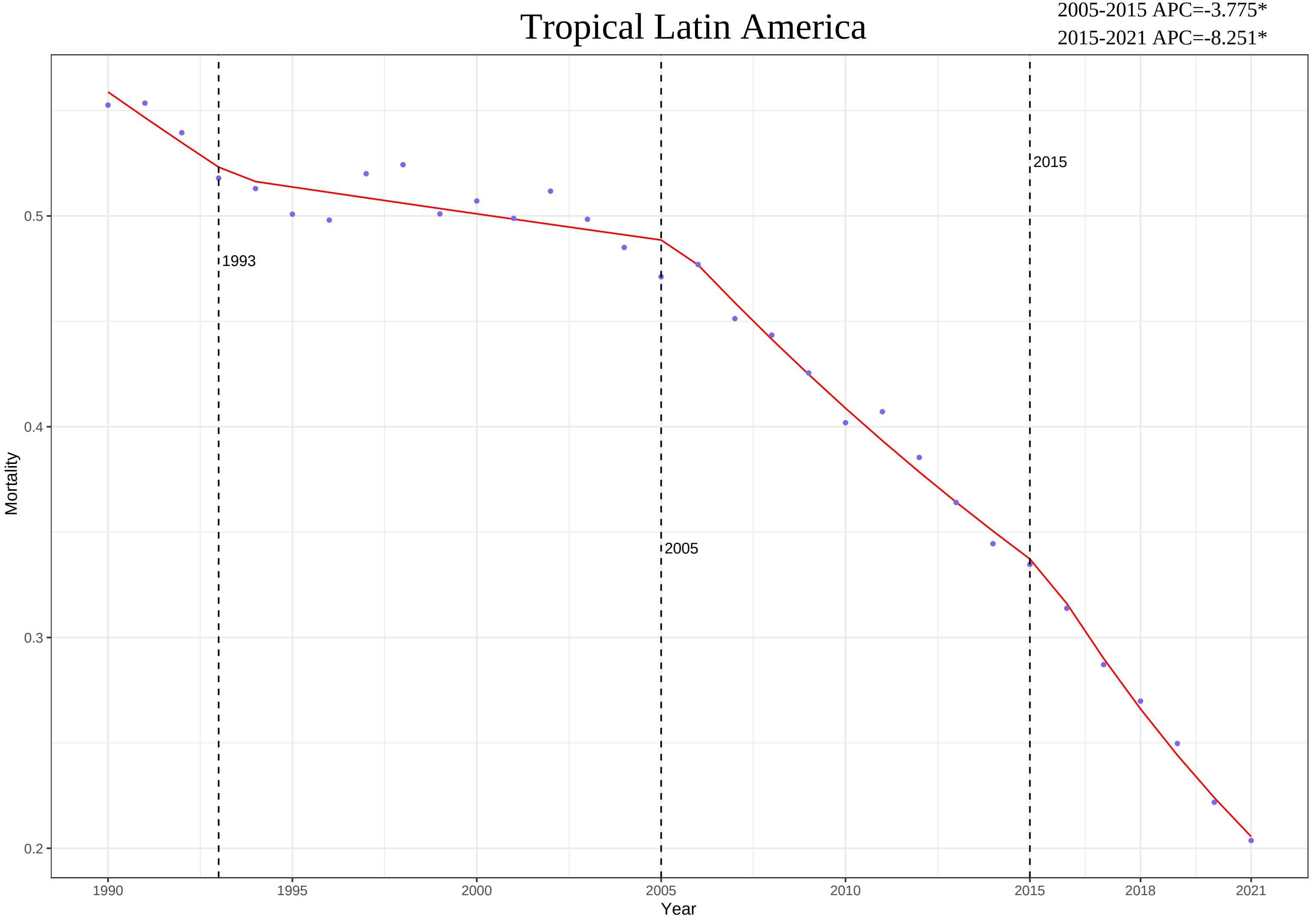

Figure S37

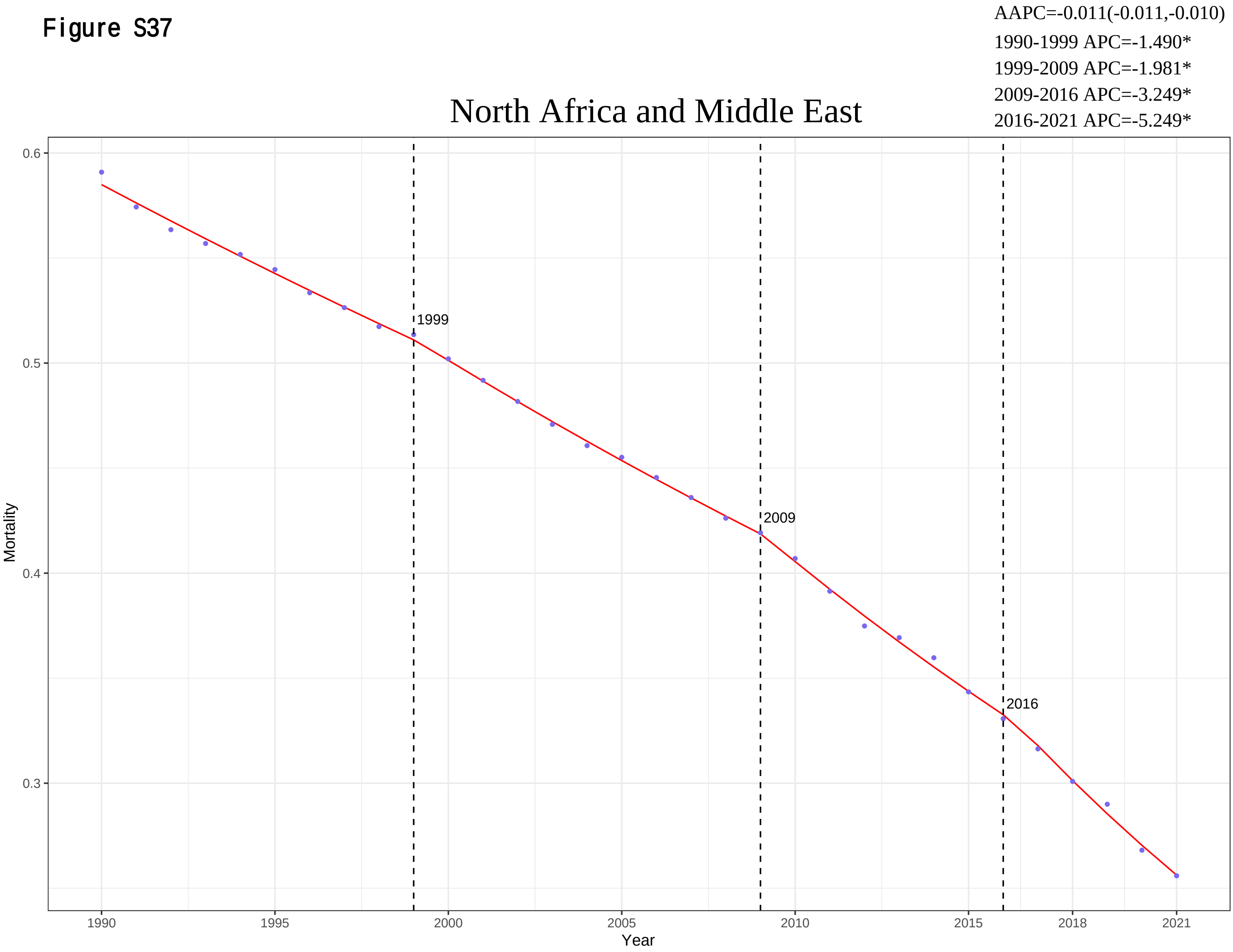
AAPC=-0.011(-0.011,-0.010)
1990-1999 APC=-1.490*
1999-2009 APC=-1.981*
2009-2016 APC=-3.249*
2016-2021 APC=-5.249*
North Africa and Middle East
Mortality
0.6
0.5
0.4
0.3
1999
2009
2016
1990
1995
2000
2005
2010
2015
2018
2021
Year

Figure S38

AAPC=-0.010(-0.011,-0.010)
1990-1999 APC=-0.851*
1999-2003 APC=-3.762*
2003-2017 APC=-1.033*
2017-2021 APC=-3.677*

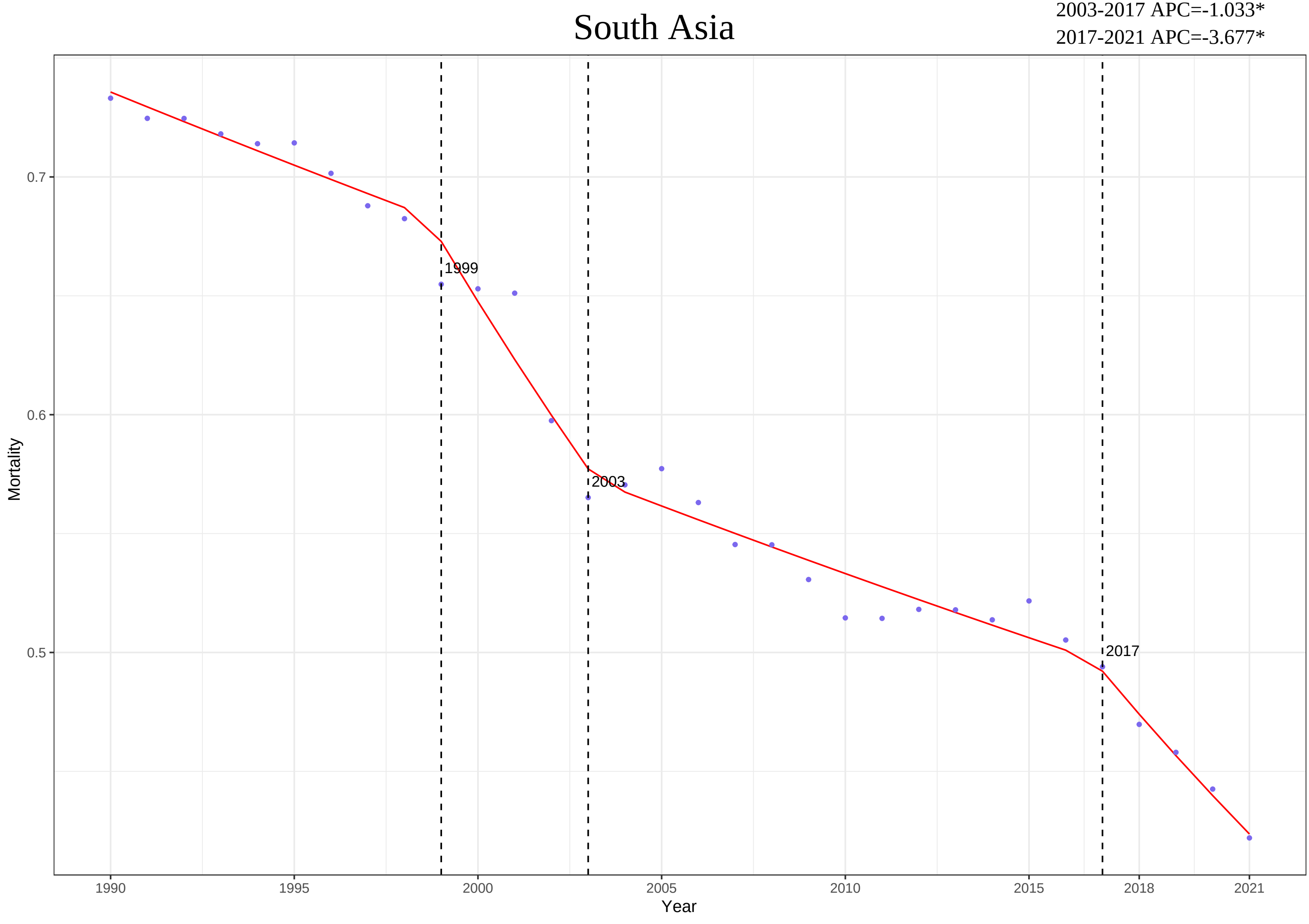

Figure S39

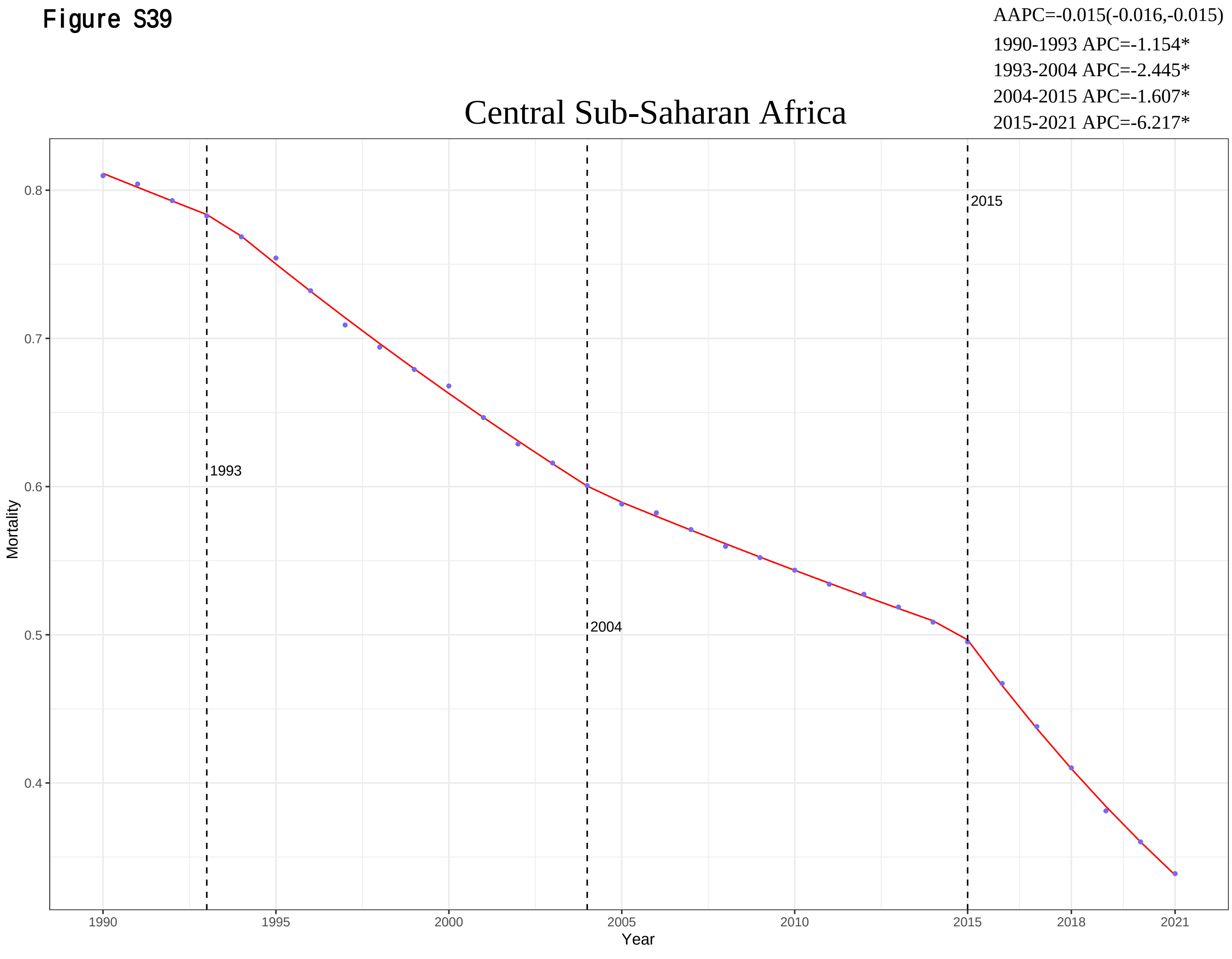
AAPC=-0.015(-0.016,-0.015)
1990-1993 APC=-1.154*
1993-2004 APC=-2.445*
2004-2015 APC=-1.607*
2015-2021 APC=-6.217*
Central Sub-Saharan Africa
Mortality
0.8
0.7
0.6
0.5
0.4
1993
2004
2015
1990
1995
2000
2005
2010
2015
2018
2021
Year

Figure S40

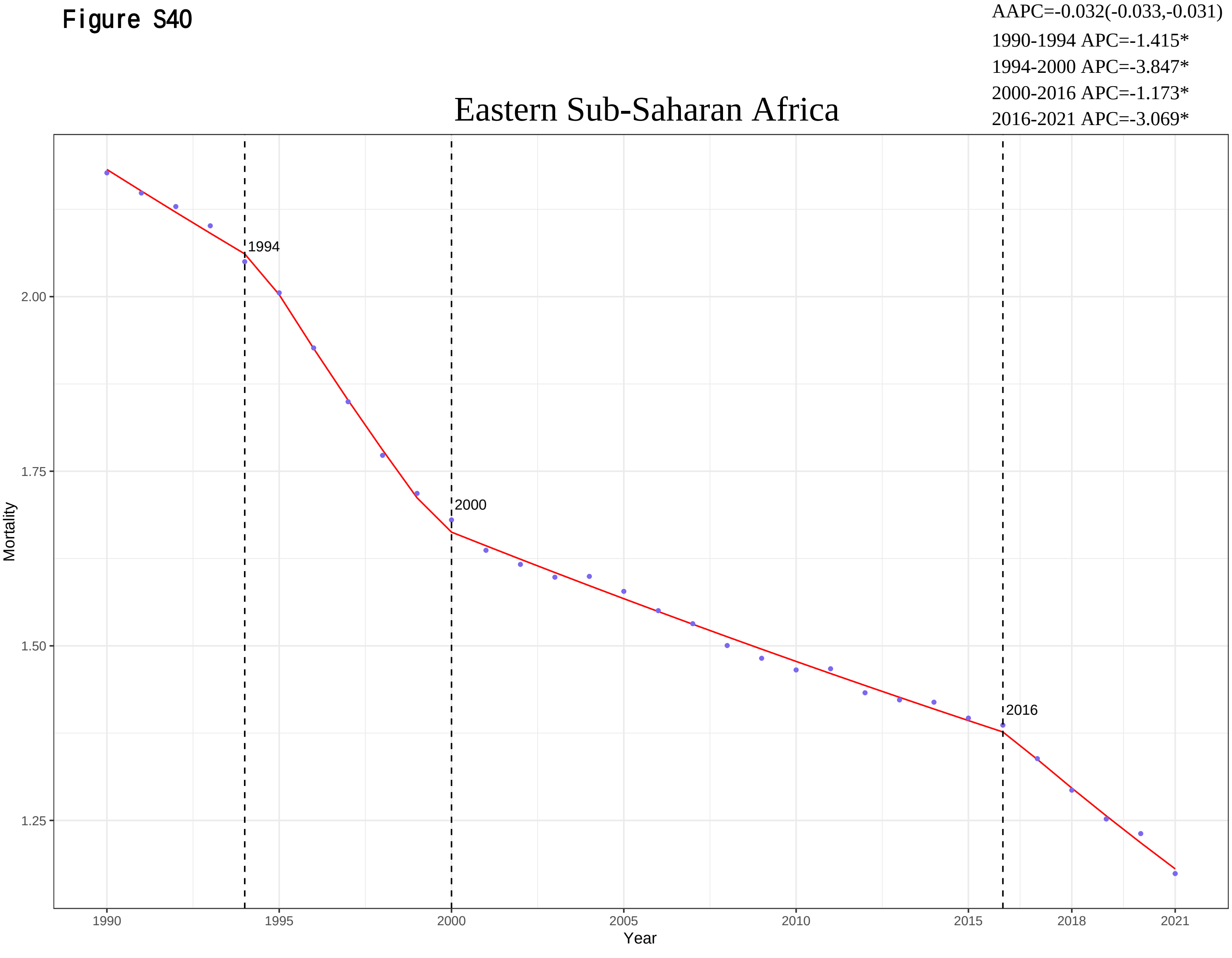
AAPC=-0.032(-0.033,-0.031)
1990-1994 APC=-1.415*
1994-2000 APC=-3.847*
2000-2016 APC=-1.173*
2016-2021 APC=-3.069*
Eastern Sub-Saharan Africa
1994
2000
2016
2.00
1.75
1.50
1.25
Mortality
1990
1995
2000
2005
2010
2015
2018
2021
Year

Figure S41

AAPC=0.006(0.005,0.007)
1990-1997 APC=-5.358*
1997-2002 APC=4.587*
2002-2015 APC=2.219*
2015-2021 APC=4.144*

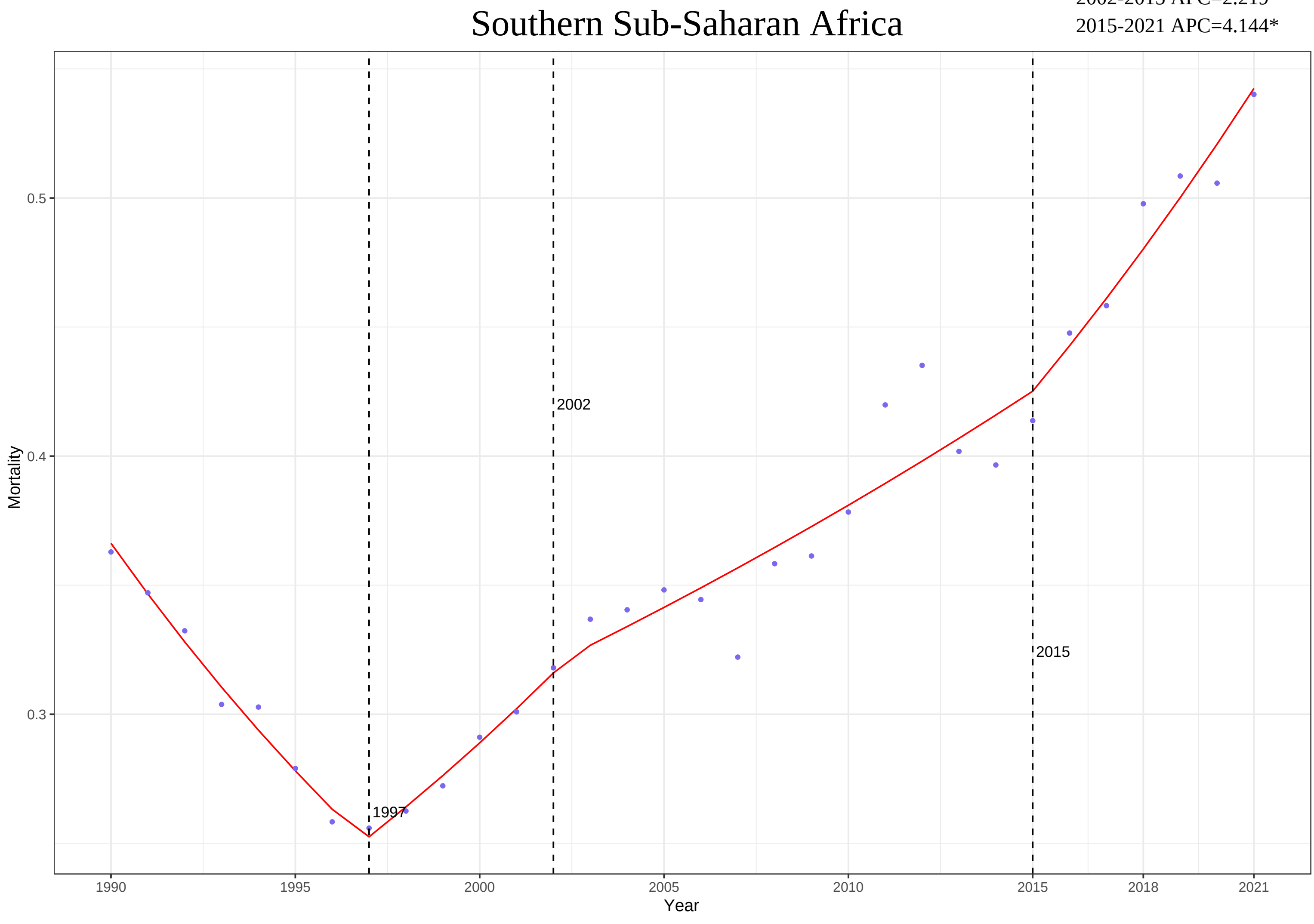

Figure S42

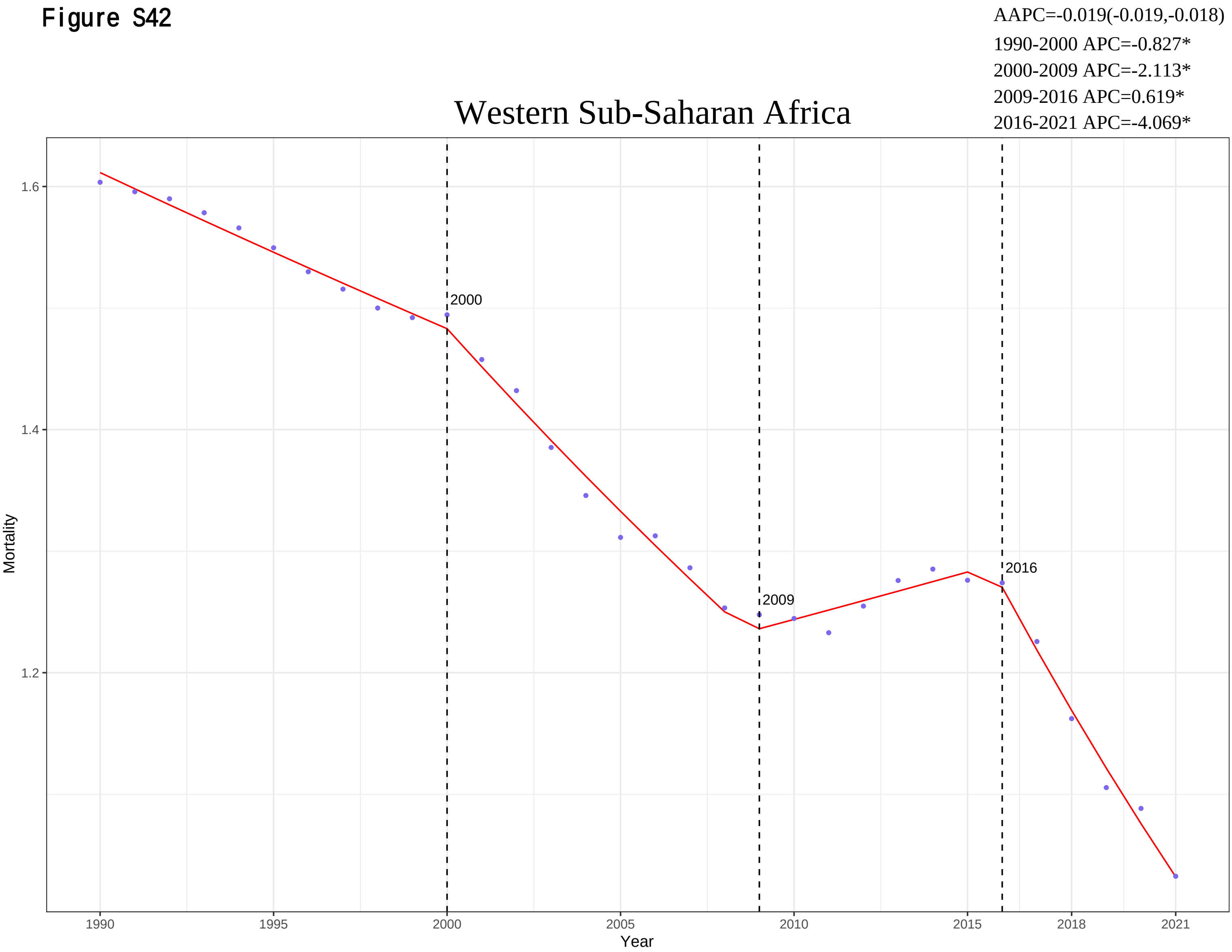
AAPC=-0.019(-0.019,-0.018)
1990-2000 APC=-0.827*
2000-2009 APC=-2.113*
2009-2016 APC=0.619*
2016-2021 APC=-4.069*
Western Sub-Saharan Africa
1.6
1.4
1.2
Mortality
2000
2009
2016
1990
1995
2000
2005
2010
2015
2018
2021
Year

Figure S43

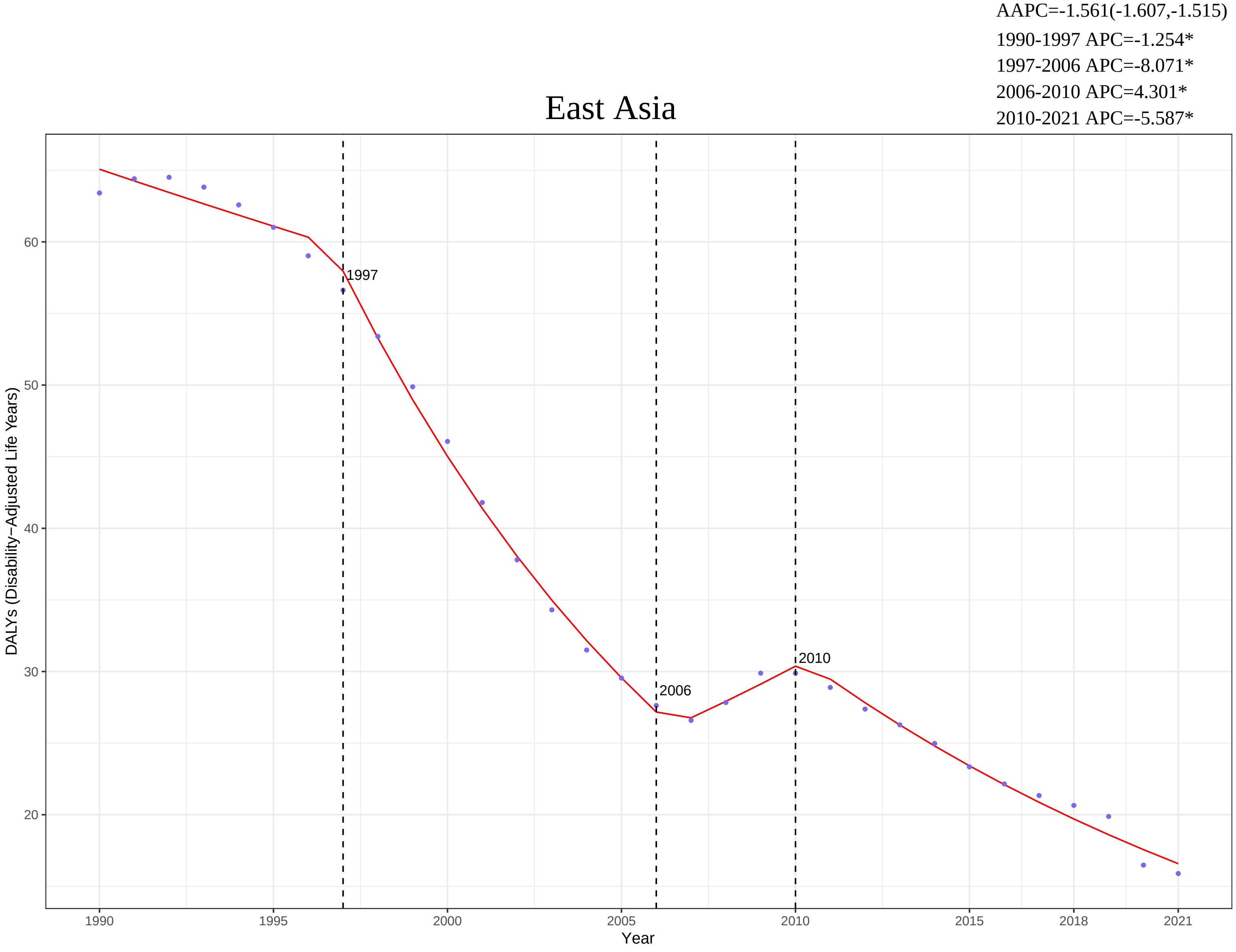
AAPC=-1.561(-1.607,-1.515)
1990-1997 APC=-1.254*
1997-2006 APC=-8.071*
2006-2010 APC=4.301*
2010-2021 APC=-5.587*
East Asia
DALYs (Disability−Adjusted Life Years)
60
50
40
30
20
1997
2006
2010
1990
1995
2000
2005
2010
2015
2018
2021
Year

Figure S44

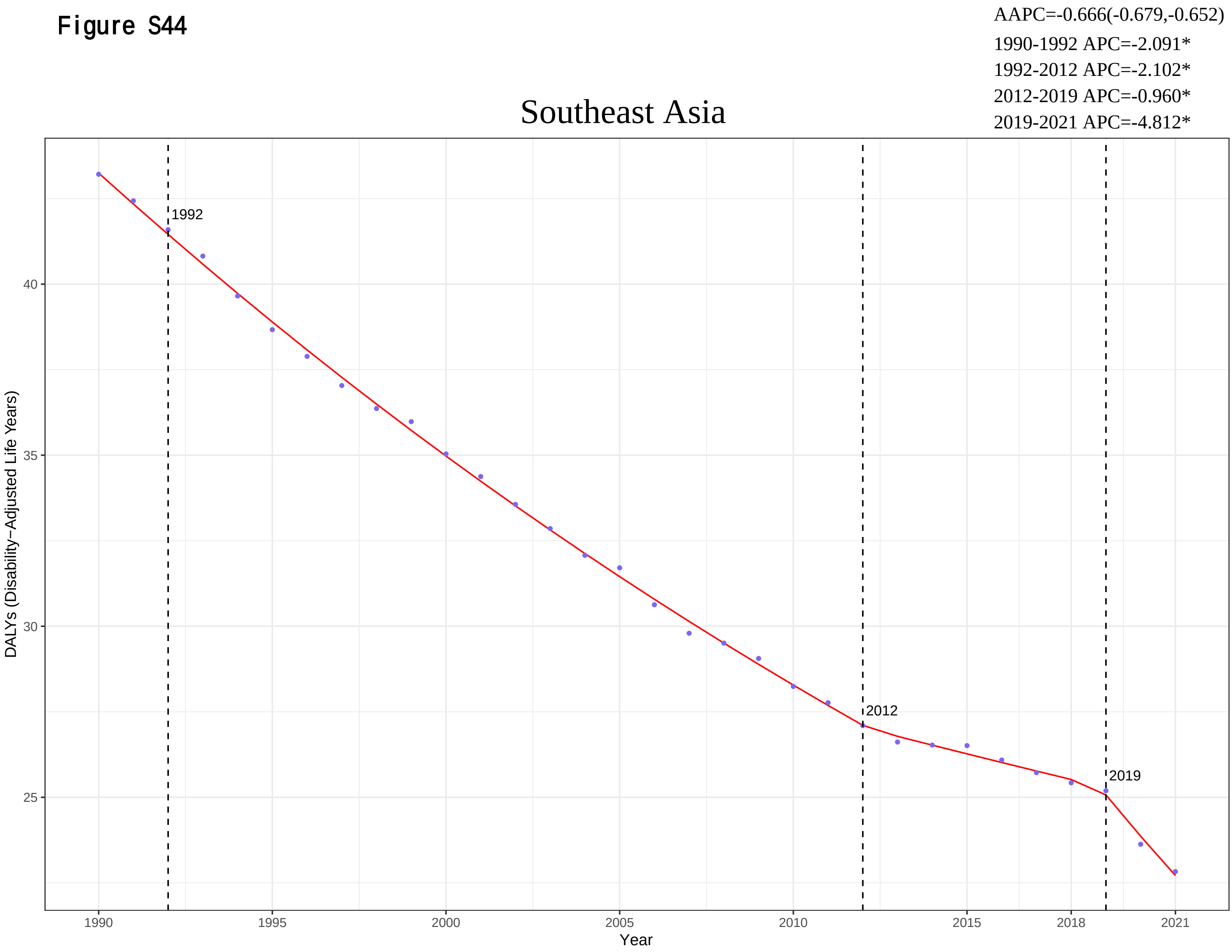
AAPC=-0.666(-0.679,-0.652)
1990-1992 APC=-2.091*
1992-2012 APC=-2.102*
2012-2019 APC=-0.960*
2019-2021 APC=-4.812*
Southeast Asia
1992
2012
2019
40
35
30
25
DALYs (Disability−Adjusted Life Years)
1990
1995
2000
2005
2010
2015
2018
2021
Year

Figure S45

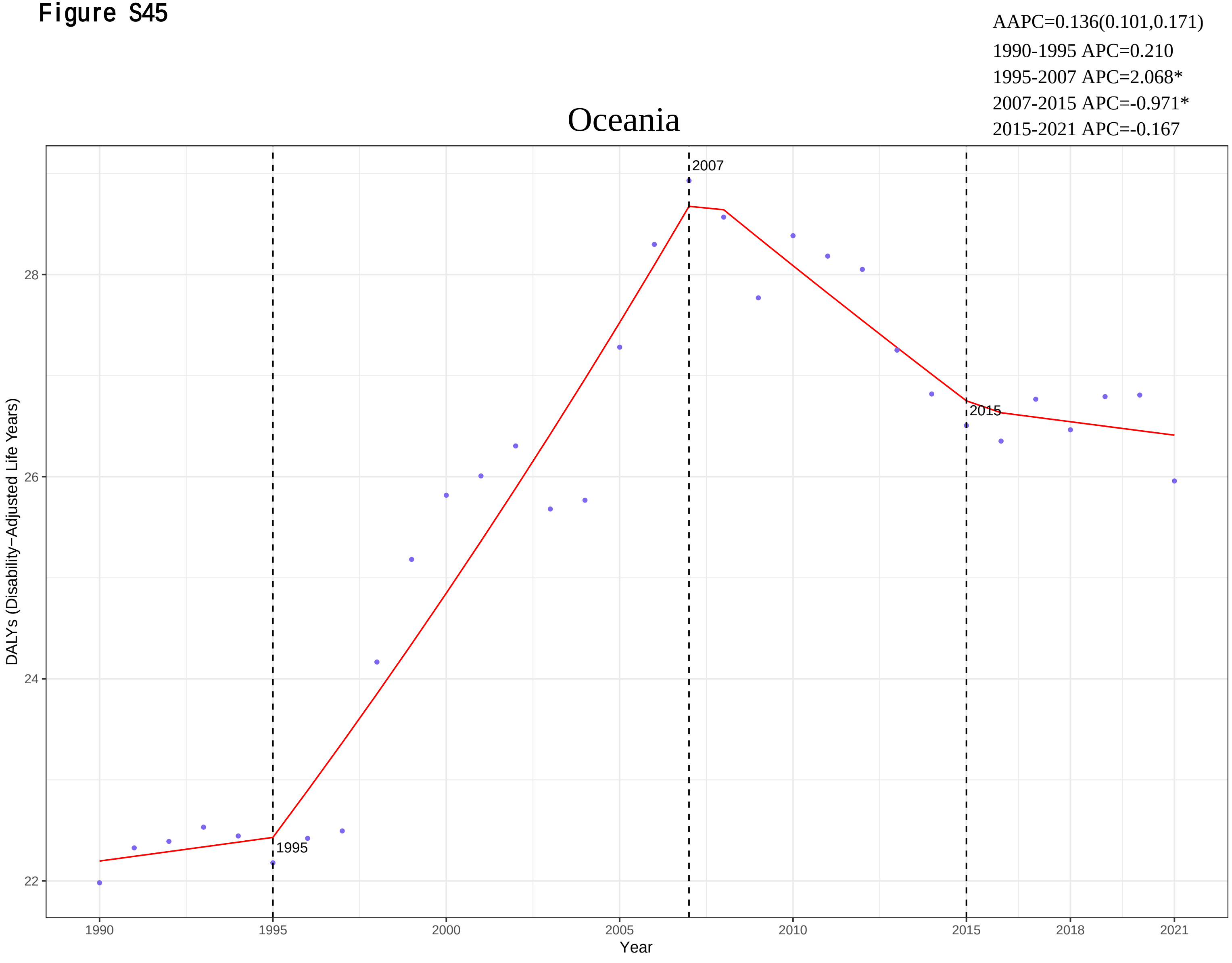

AAPC=0.136(0.101,0.171)
1990-1995 APC=0.210
1995-2007 APC=2.068*
2007-2015 APC=-0.971*
2015-2021 APC=-0.167
Oceania
DALYs (Disability−Adjusted Life Years)
Year
1995
2007
2015
22
24
26
28
1990
1995
2000
2005
2010
2015
2018
2021

Figure S46

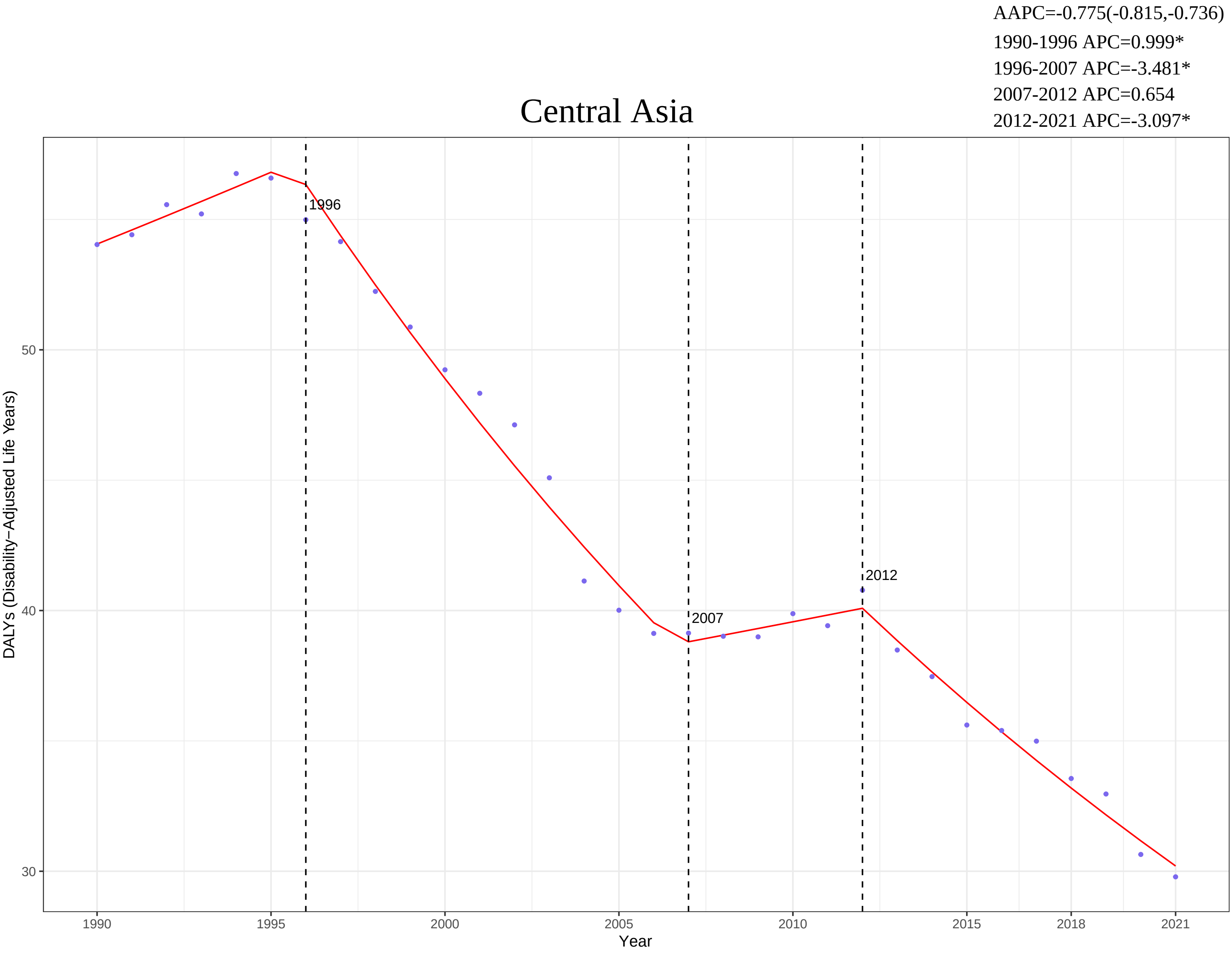
AAPC=-0.775(-0.815,-0.736)
1990-1996 APC=0.999*
1996-2007 APC=-3.481*
2007-2012 APC=0.654
2012-2021 APC=-3.097*
Central Asia
1996
2007
2012
DALYs (Disability−Adjusted Life Years)
50
40
30
1990
1995
2000
2005
2010
2015
2018
2021
Year

Figure S47

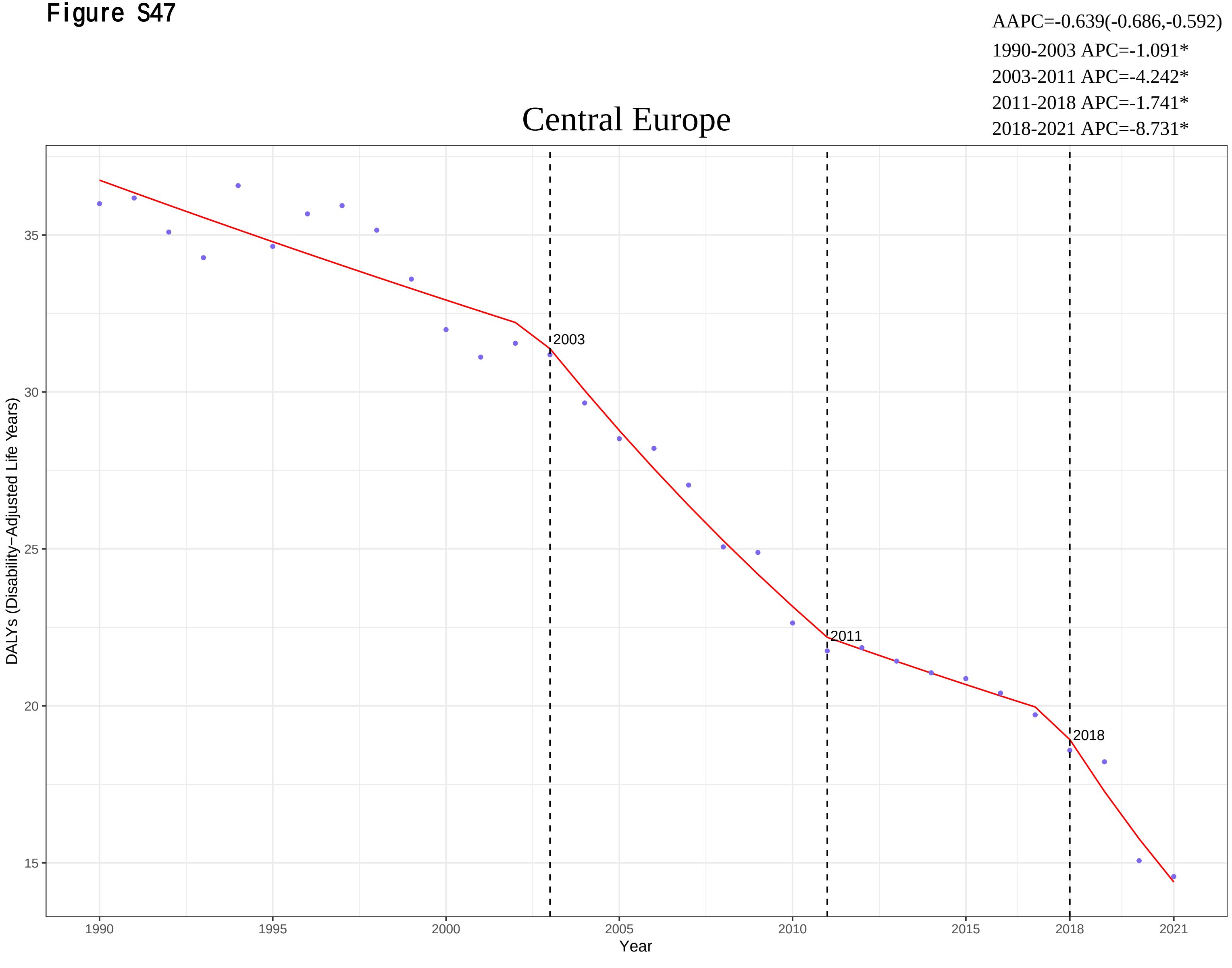
AAPC=-0.639(-0.686,-0.592)
1990-2003 APC=-1.091*
2003-2011 APC=-4.242*
2011-2018 APC=-1.741*
2018-2021 APC=-8.731*
Central Europe
DALYs (Disability−Adjusted Life Years)
35
30
25
20
15
2003
2011
2018
1990
1995
2000
2005
2010
2015
2018
2021
Year

Figure S48

AAPC=-1.060(-1.128,-0.991)
1990-1992 APC=-1.986
1992-2010 APC=-5.312*
2010-2019 APC=0.425
2019-2021 APC=-7.055*

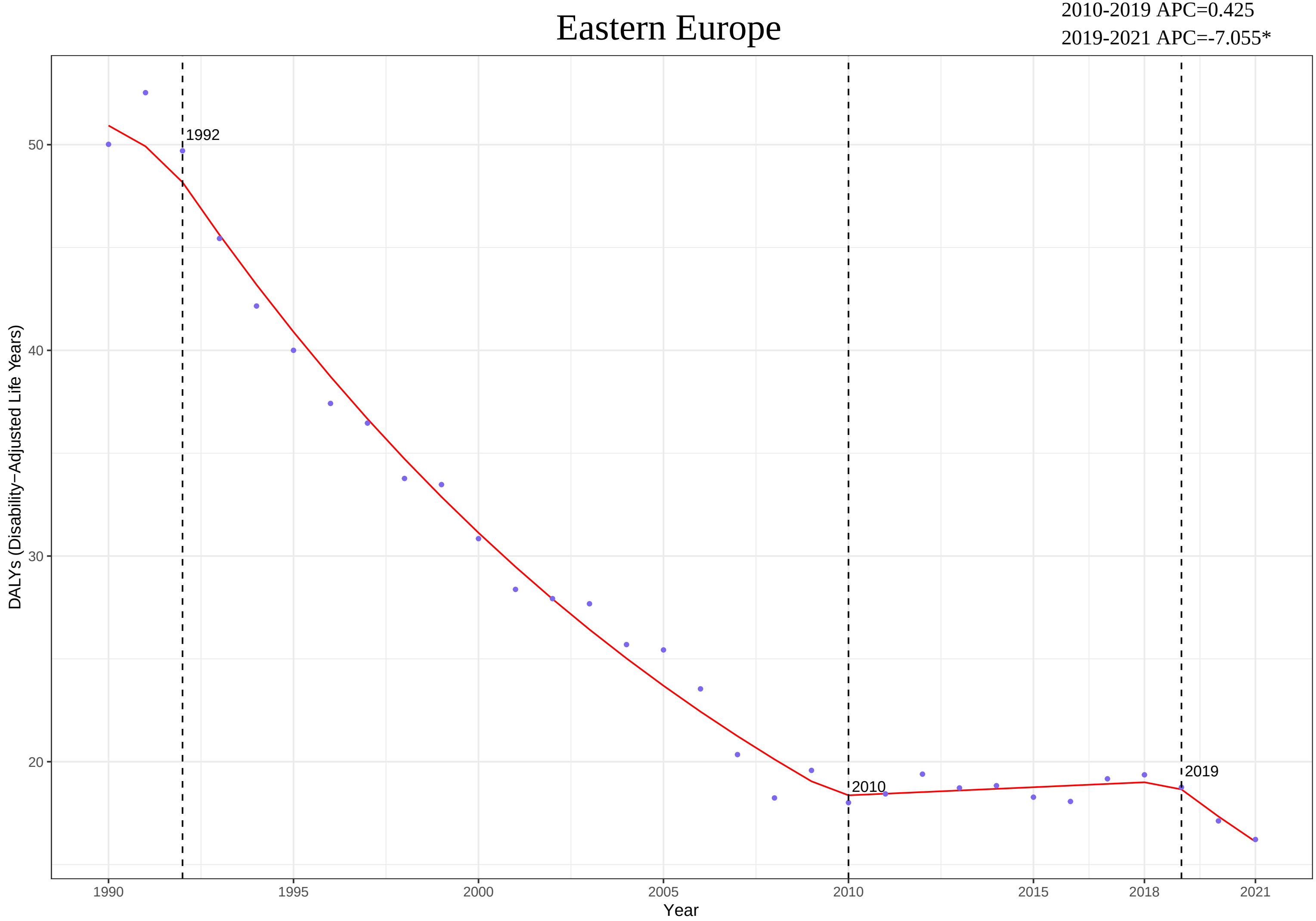

Figure S49

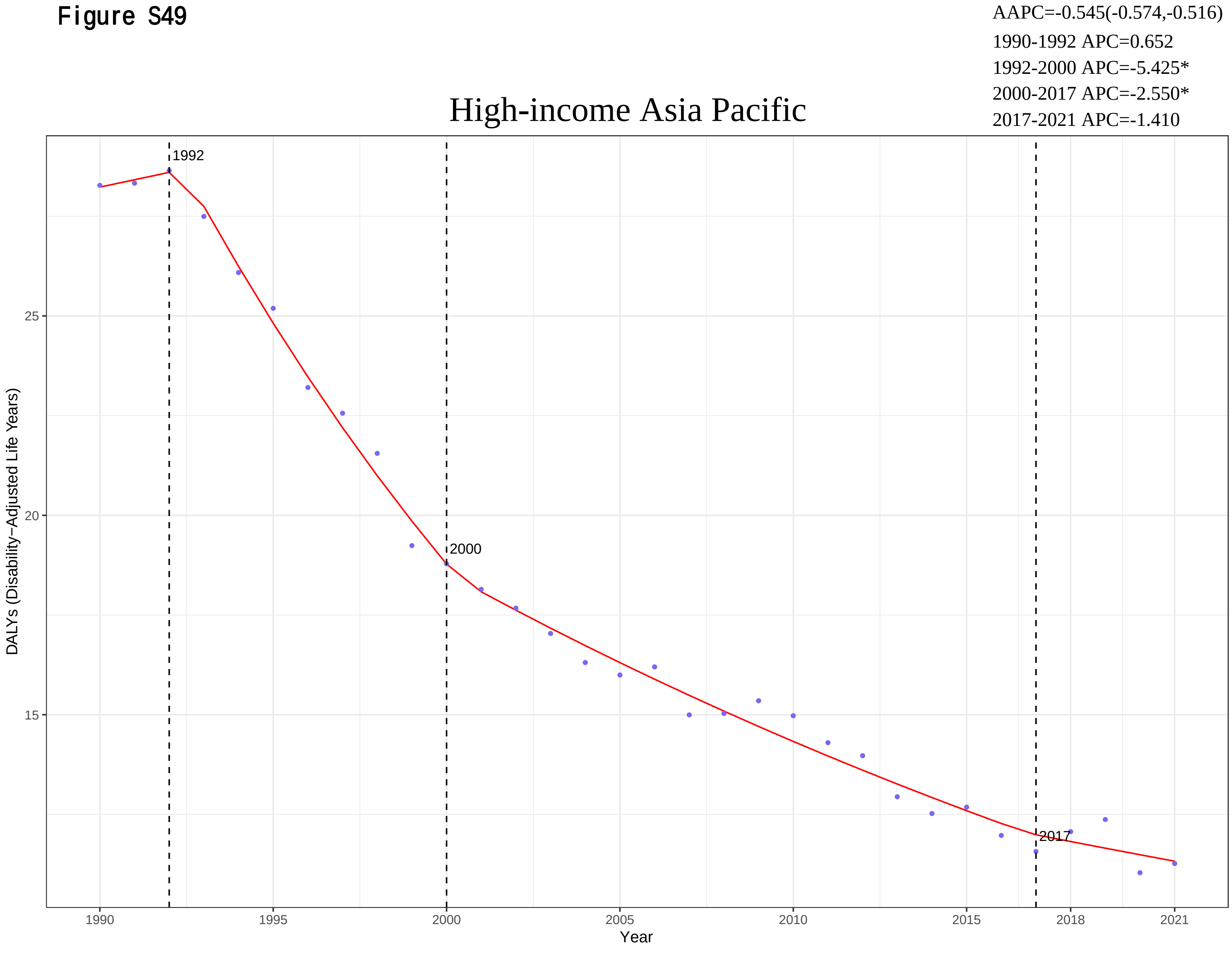
AAPC=-0.545(-0.574,-0.516)
1990-1992 APC=0.652
1992-2000 APC=-5.425*
2000-2017 APC=-2.550*
2017-2021 APC=-1.410
High-income Asia Pacific
1992
2000
2017
25
20
15
DALYs (Disability−Adjusted Life Years)
1990
1995
2000
2005
2010
2015
2018
2021
Year

Figure S50

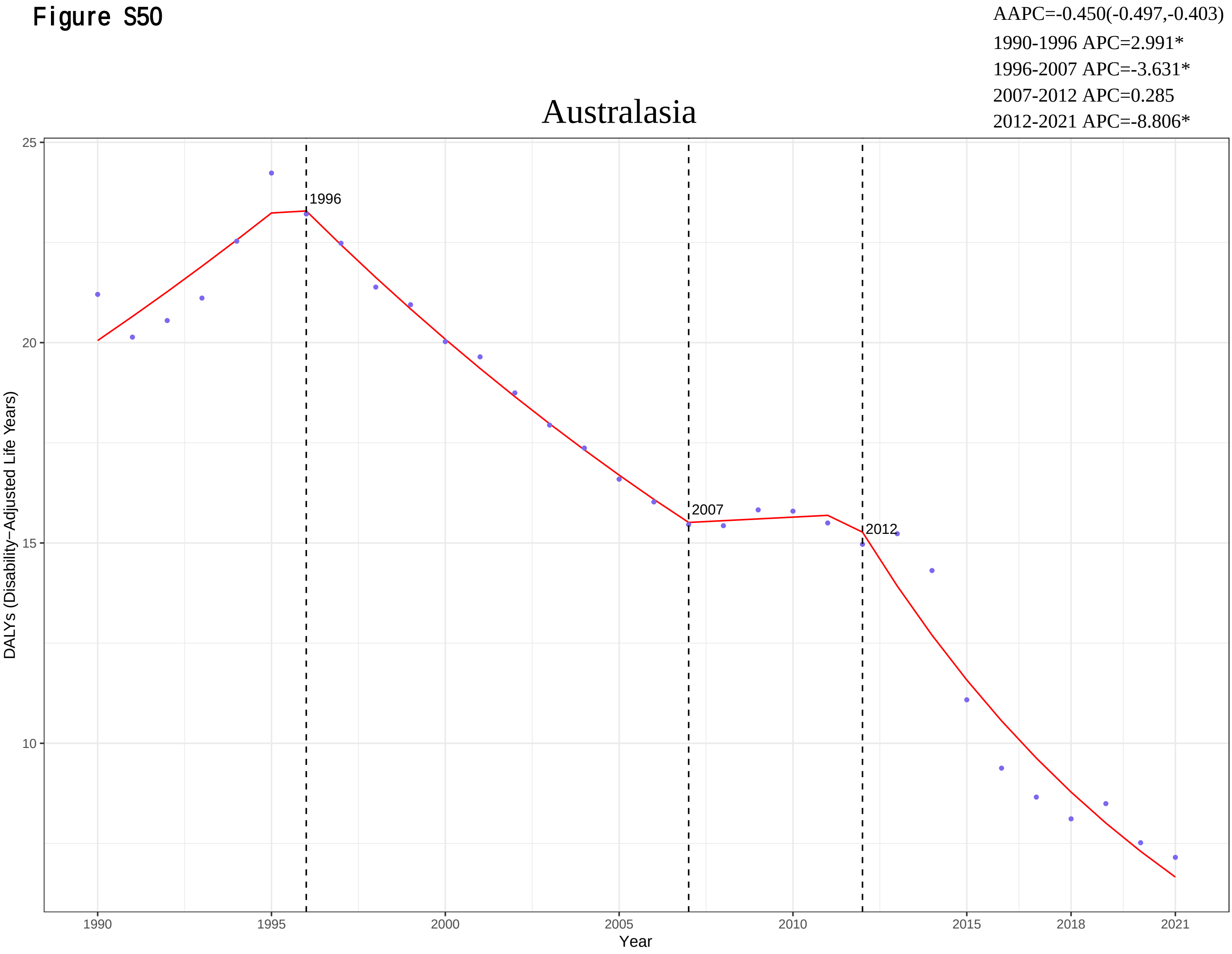
AAPC=-0.450(-0.497,-0.403)
1990-1996 APC=2.991*
1996-2007 APC=-3.631*
2007-2012 APC=0.285
2012-2021 APC=-8.806*
Australasia
25
20
15
10
1996
2007
2012
DALYs (Disability−Adjusted Life Years)
1990
1995
2000
2005
2010
2015
2018
2021
Year

Figure S51

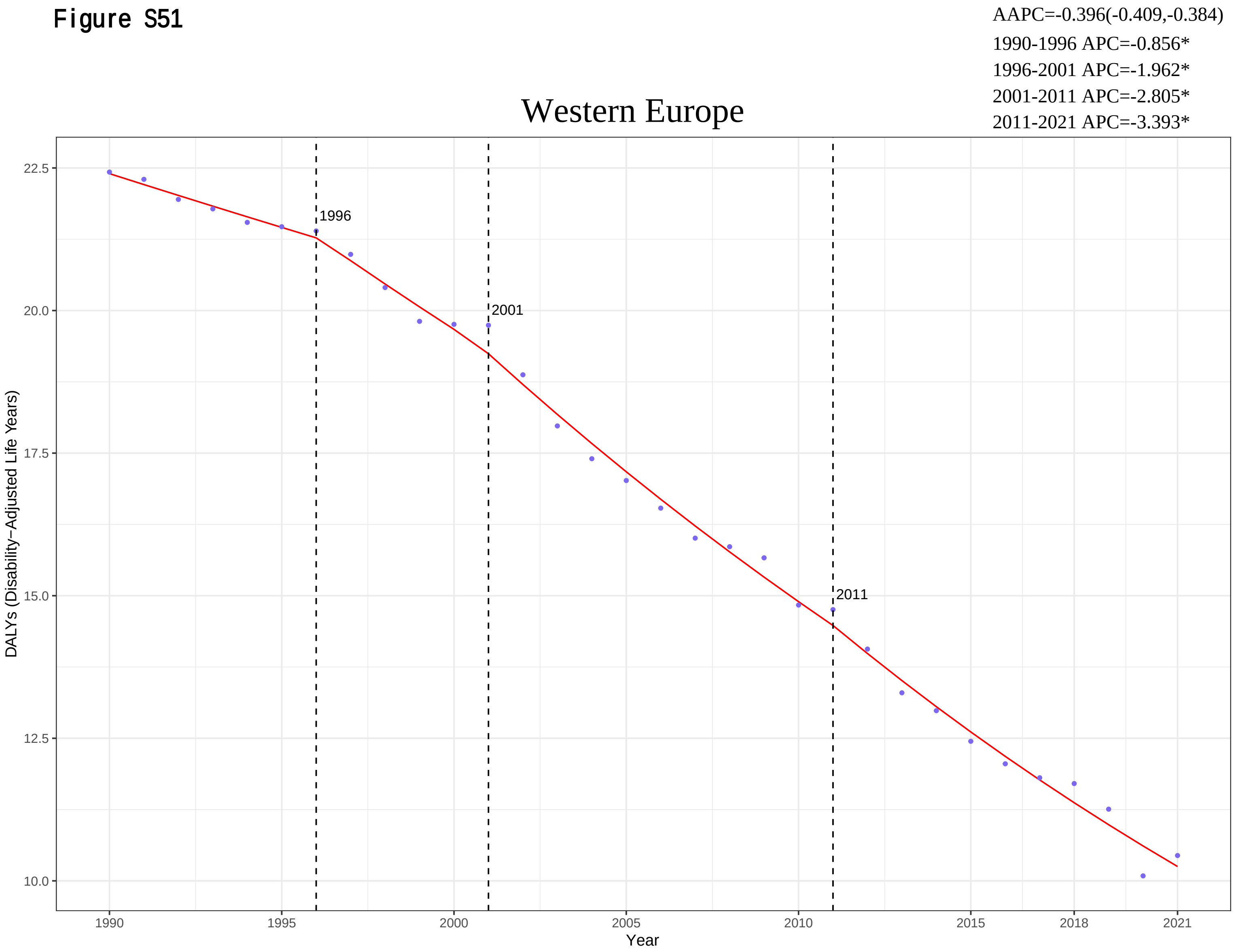

AAPC=-0.396(-0.409,-0.384)
1990-1996 APC=-0.856*
1996-2001 APC=-1.962*
2001-2011 APC=-2.805*
2011-2021 APC=-3.393*
Western Europe
DALYs (Disability−Adjusted Life Years)
22.5
20.0
17.5
15.0
12.5
10.0
1996
2001
2011
1990
1995
2000
2005
2010
2015
2018
2021
Year

Figure S52

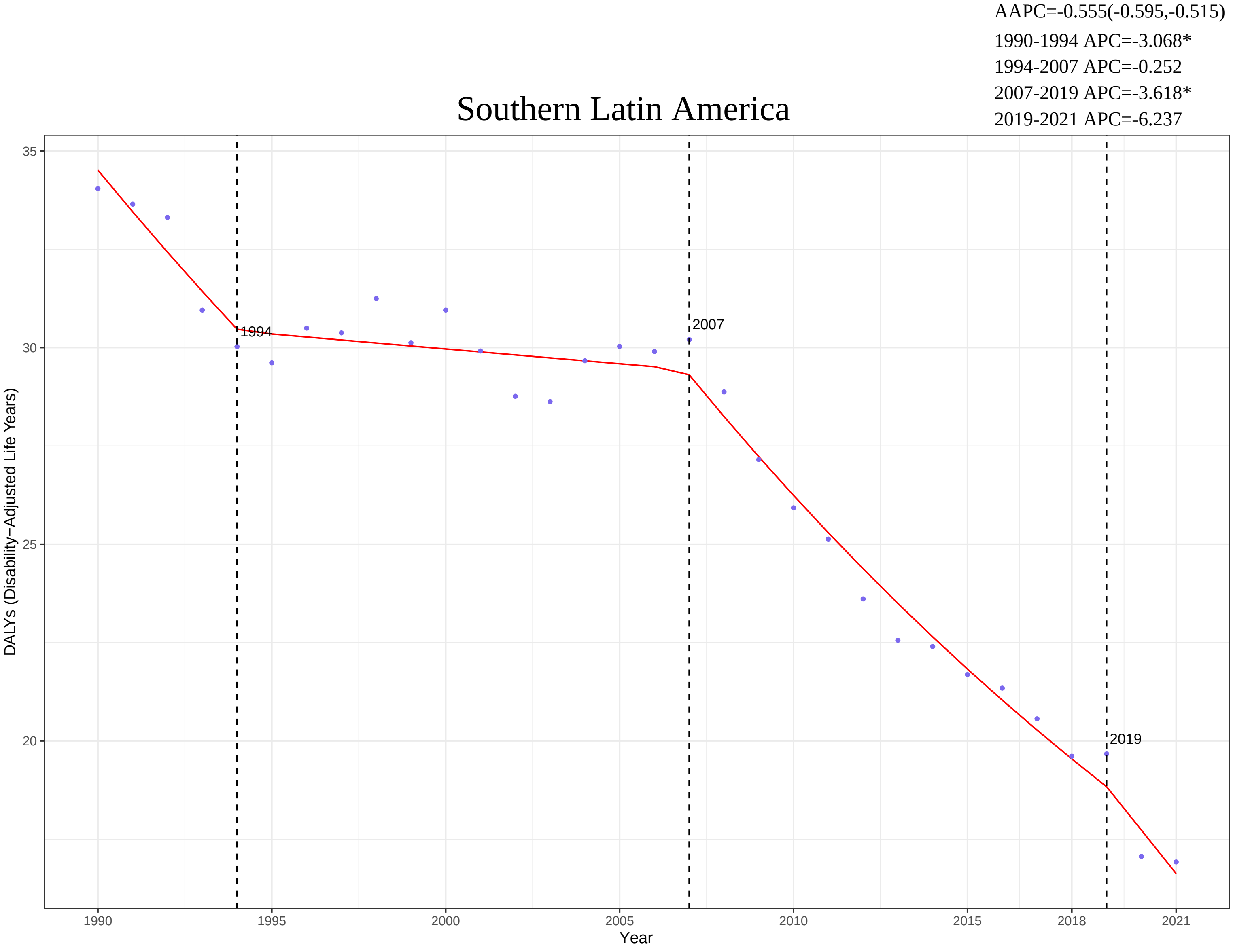
AAPC=-0.555(-0.595,-0.515)
1990-1994 APC=-3.068*
1994-2007 APC=-0.252
2007-2019 APC=-3.618*
2019-2021 APC=-6.237
Southern Latin America
DALYs (Disability−Adjusted Life Years)
35
30
25
20
1994
2007
2019
1990
1995
2000
2005
2010
2015
2018
2021
Year

Figure S53

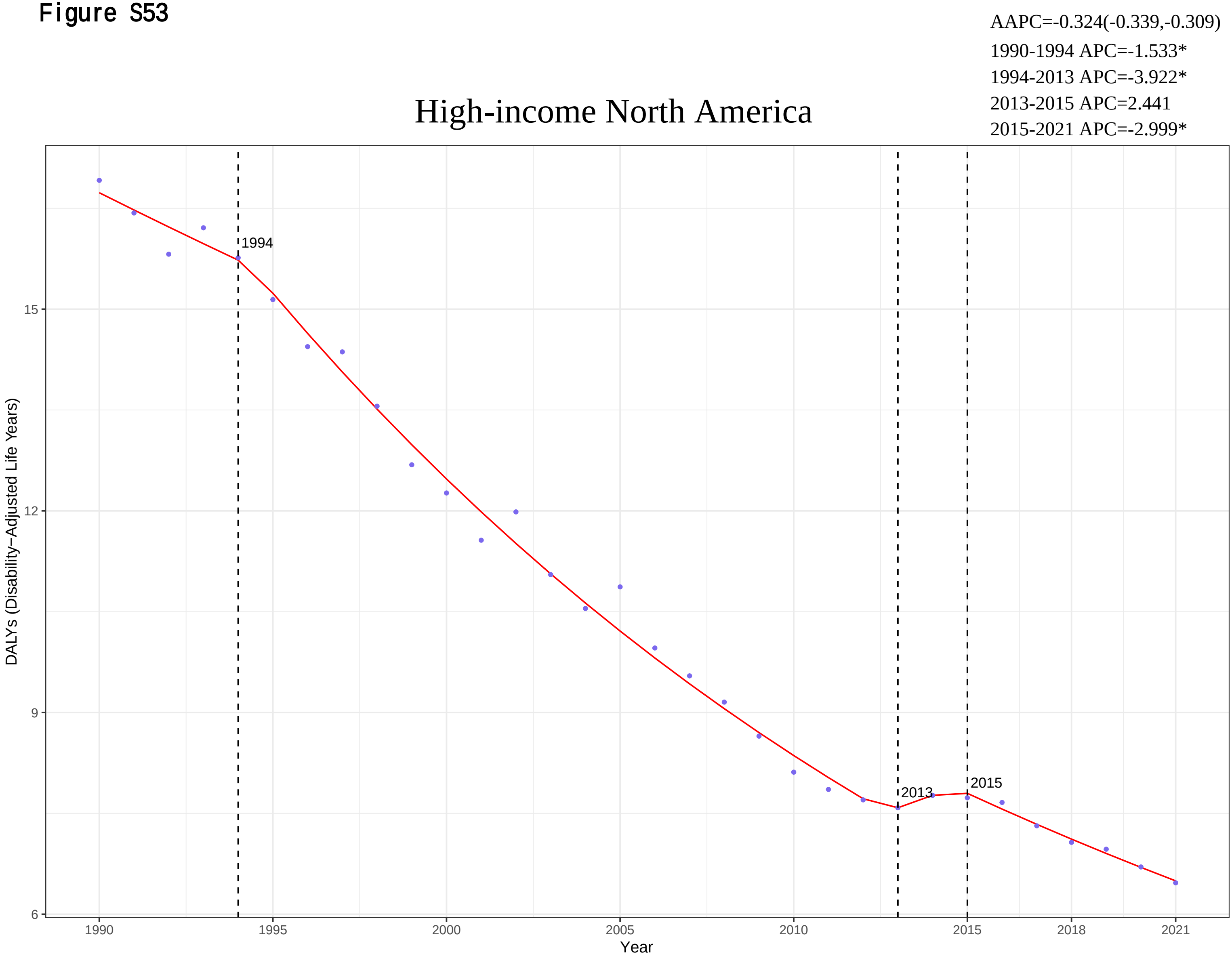

AAPC=-0.324(-0.339,-0.309)
1990-1994 APC=-1.533*
1994-2013 APC=-3.922*
2013-2015 APC=2.441
2015-2021 APC=-2.999*
High-income North America
1994
2013
2015
15
12
9
6
DALYs (Disability-Adjusted Life Years)
1990
1995
2000
2005
2010
2015
2018
2021
Year

Figure S54

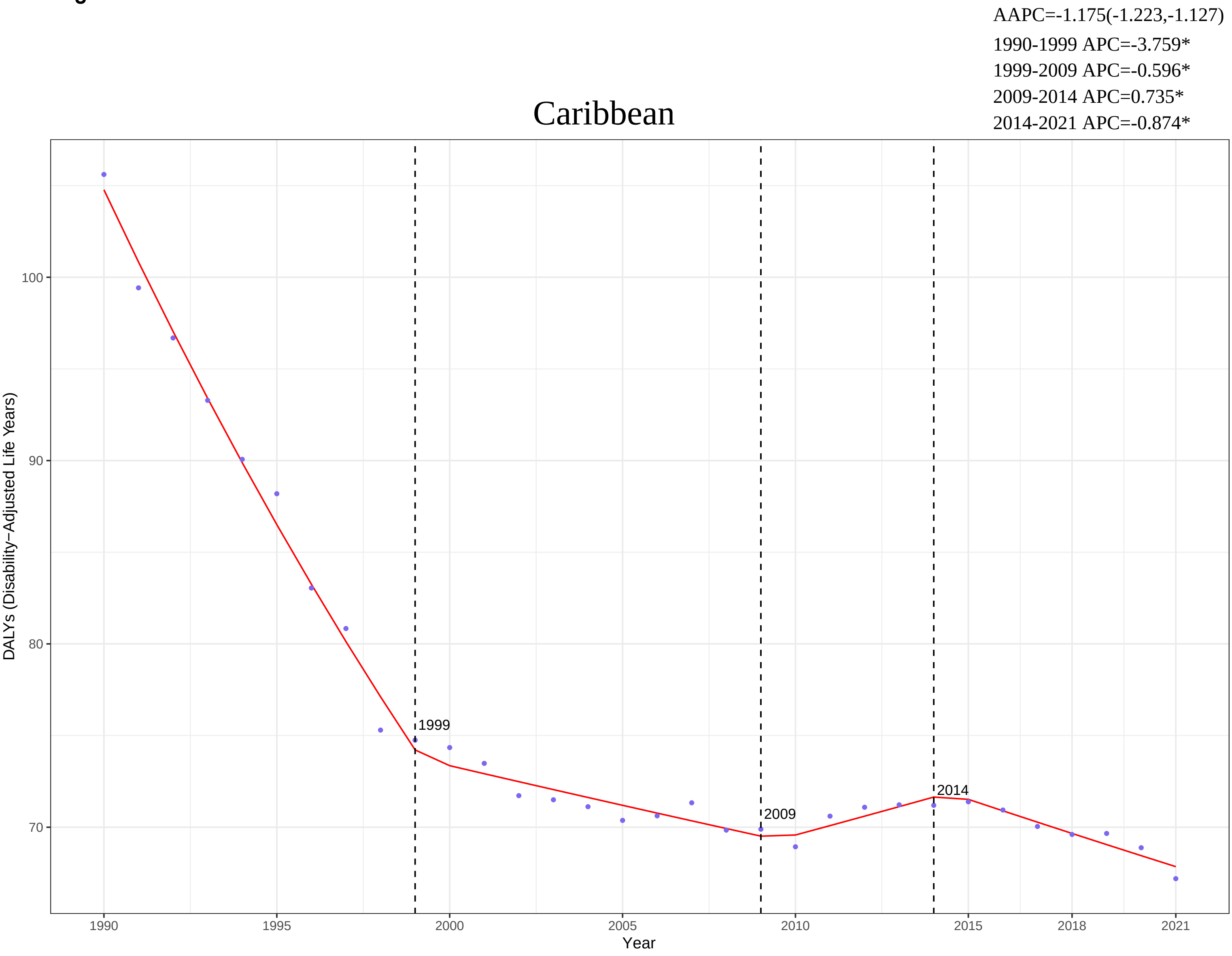
AAPC=-1.175(-1.223,-1.127)
1990-1999 APC=-3.759*
1999-2009 APC=-0.596*
2009-2014 APC=0.735*
2014-2021 APC=-0.874*
Caribbean
DALYs (Disability−Adjusted Life Years)
100
90
80
70
1999
2009
2014
1990
1995
2000
2005
2010
2015
2018
2021
Year

Figure S55

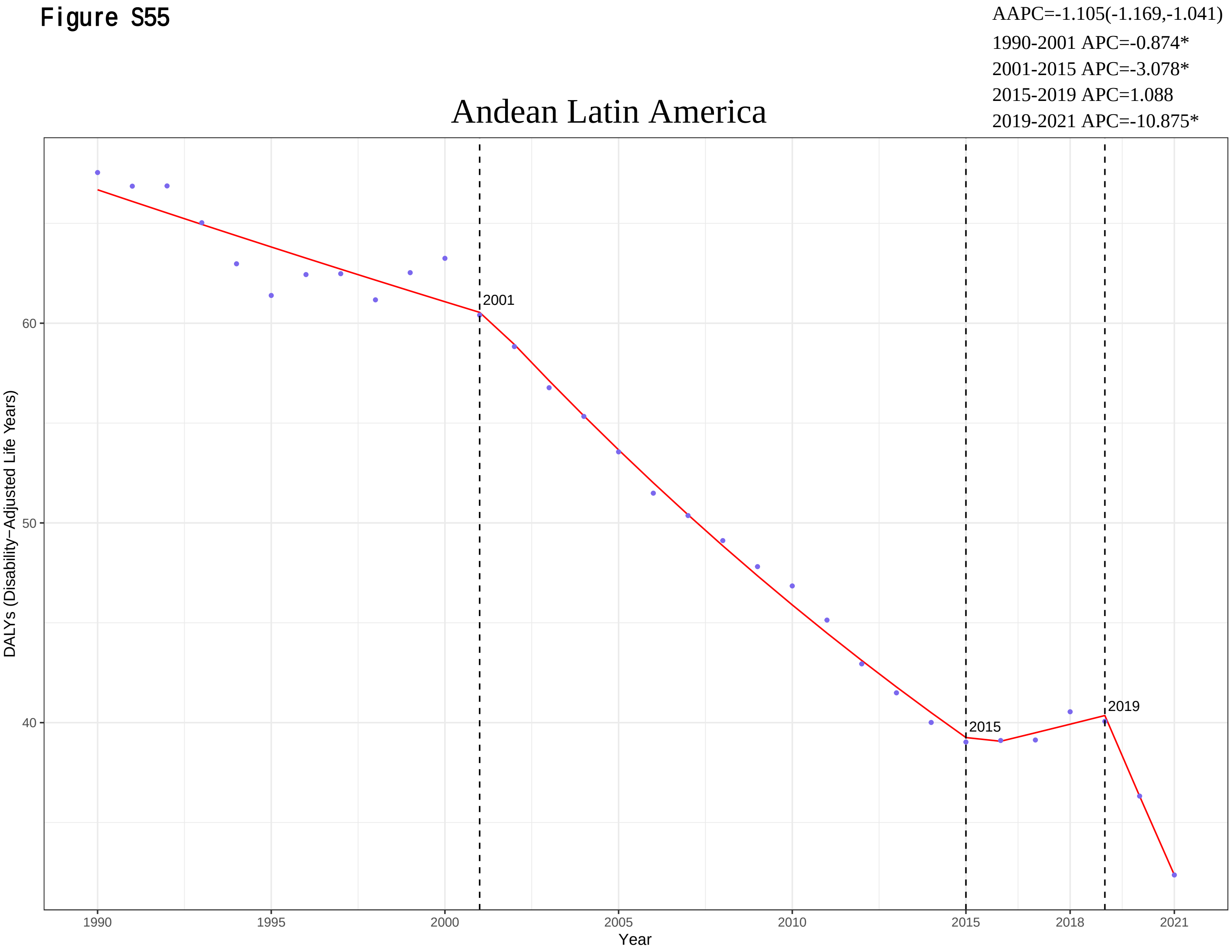
AAPC=-1.105(-1.169,-1.041)
1990-2001 APC=-0.874*
2001-2015 APC=-3.078*
2015-2019 APC=1.088
2019-2021 APC=-10.875*
Andean Latin America
2001
2015
2019
60
50
40
DALYs (Disability−Adjusted Life Years)
1990
1995
2000
2005
2010
2015
2018
2021
Year

Figure S56

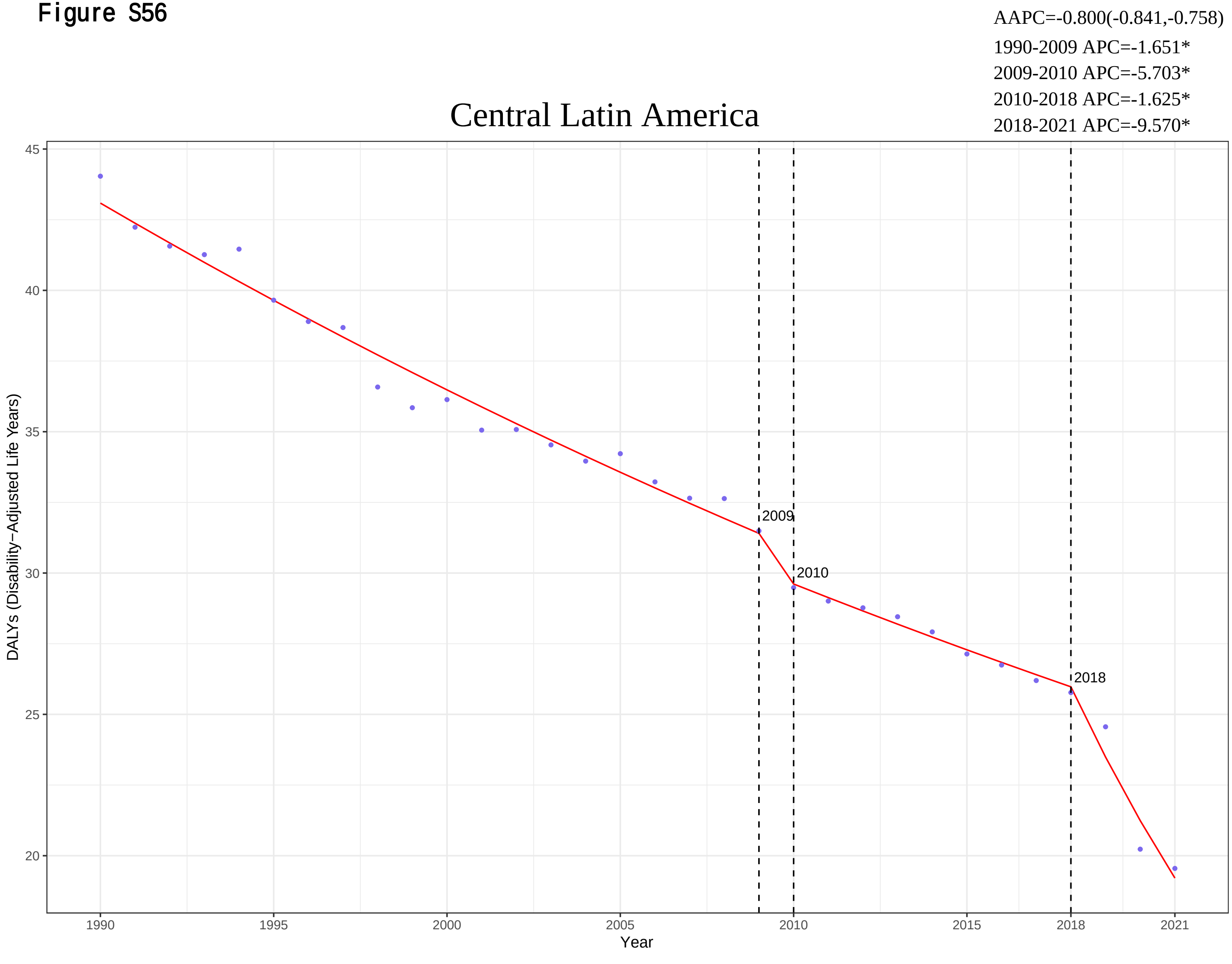

AAPC=-0.800(-0.841,-0.758)
1990-2009 APC=-1.651*
2009-2010 APC=-5.703*
2010-2018 APC=-1.625*
2018-2021 APC=-9.570*
Central Latin America
DALYs (Disability−Adjusted Life Years)
45
40
35
30
25
20
2009
2010
2018
1990
1995
2000
2005
2010
2015
2018
2021
Year

Figure S57

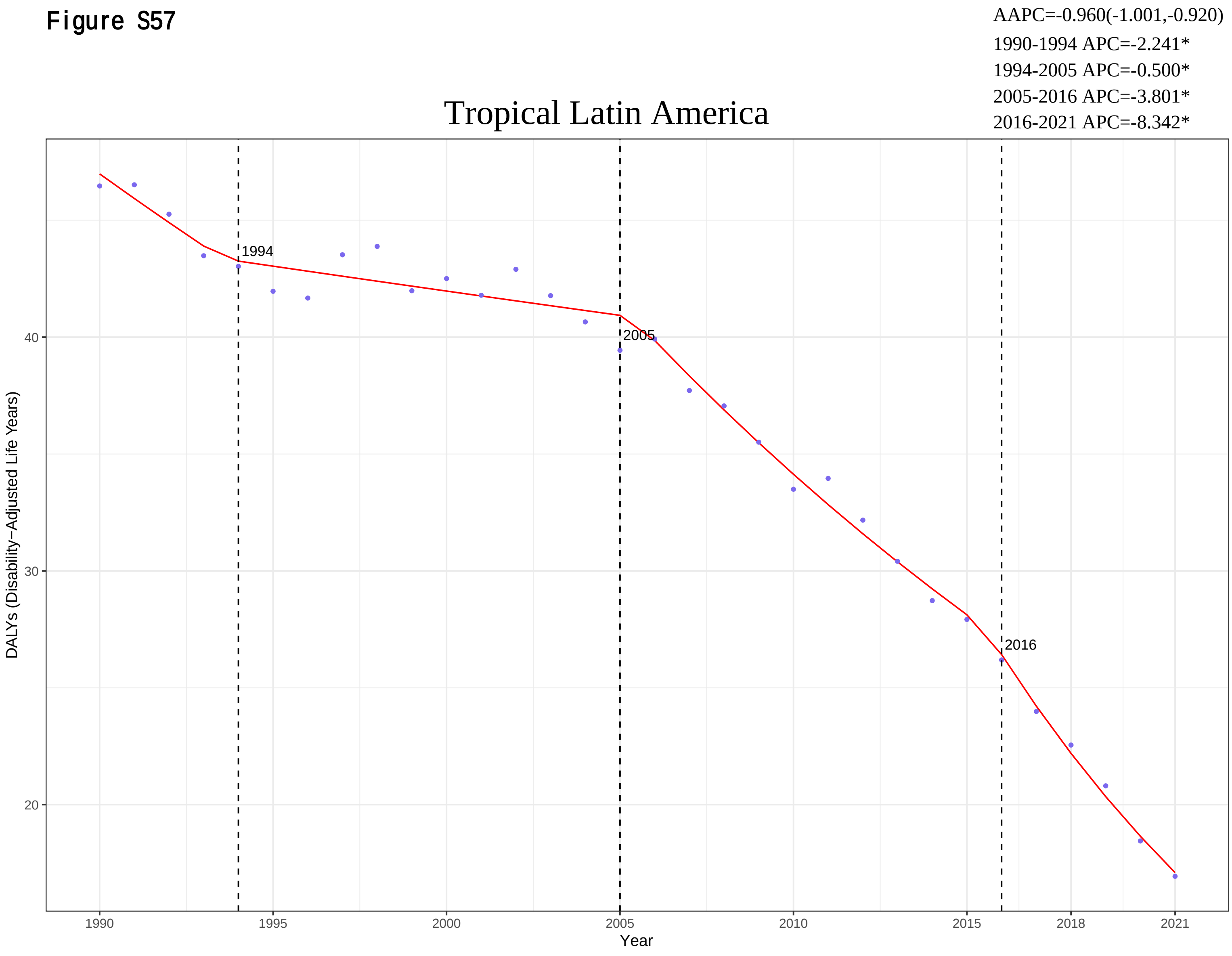
AAPC=-0.960(-1.001,-0.920)
1990-1994 APC=-2.241*
1994-2005 APC=-0.500*
2005-2016 APC=-3.801*
2016-2021 APC=-8.342*
Tropical Latin America
1994
2005
2016
40
30
20
DALYs (Disability−Adjusted Life Years)
1990
1995
2000
2005
2010
2015
2018
2021
Year

Figure S58

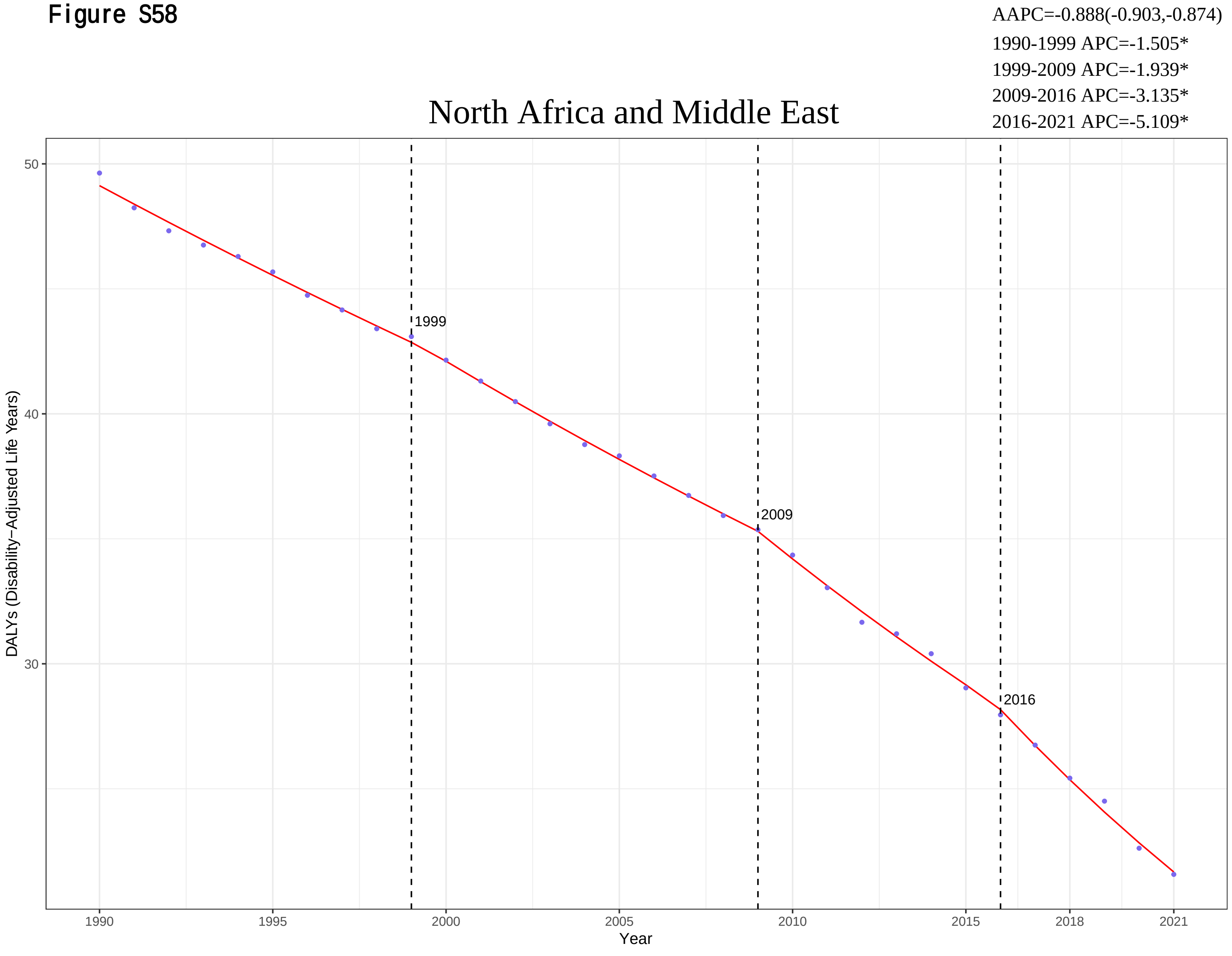

AAPC=-0.888(-0.903,-0.874)
1990-1999 APC=-1.505*
1999-2009 APC=-1.939*
2009-2016 APC=-3.135*
2016-2021 APC=-5.109*
North Africa and Middle East
DALYs (Disability−Adjusted Life Years)
50
40
30
1999
2009
2016
1990
1995
2000
2005
2010
2015
2018
2021
Year

Figure S59

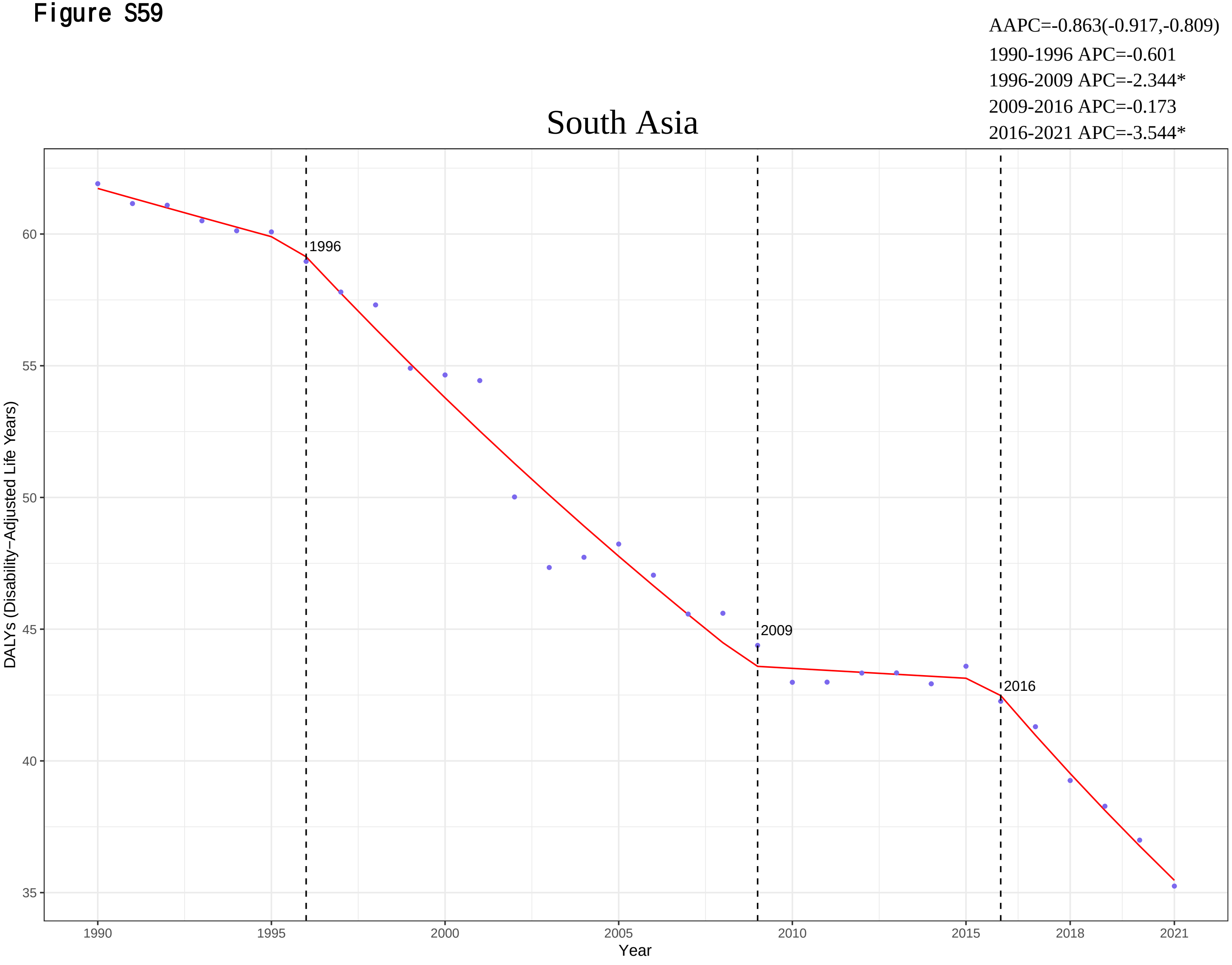
AAPC=-0.863(-0.917,-0.809)
1990-1996 APC=-0.601
1996-2009 APC=-2.344*
2009-2016 APC=-0.173
2016-2021 APC=-3.544*
South Asia
DALYs (Disability-Adjusted Life Years)
60
55
50
45
40
35
1996
2009
2016
1990
1995
2000
2005
2010
2015
2018
2021
Year

Figure S60

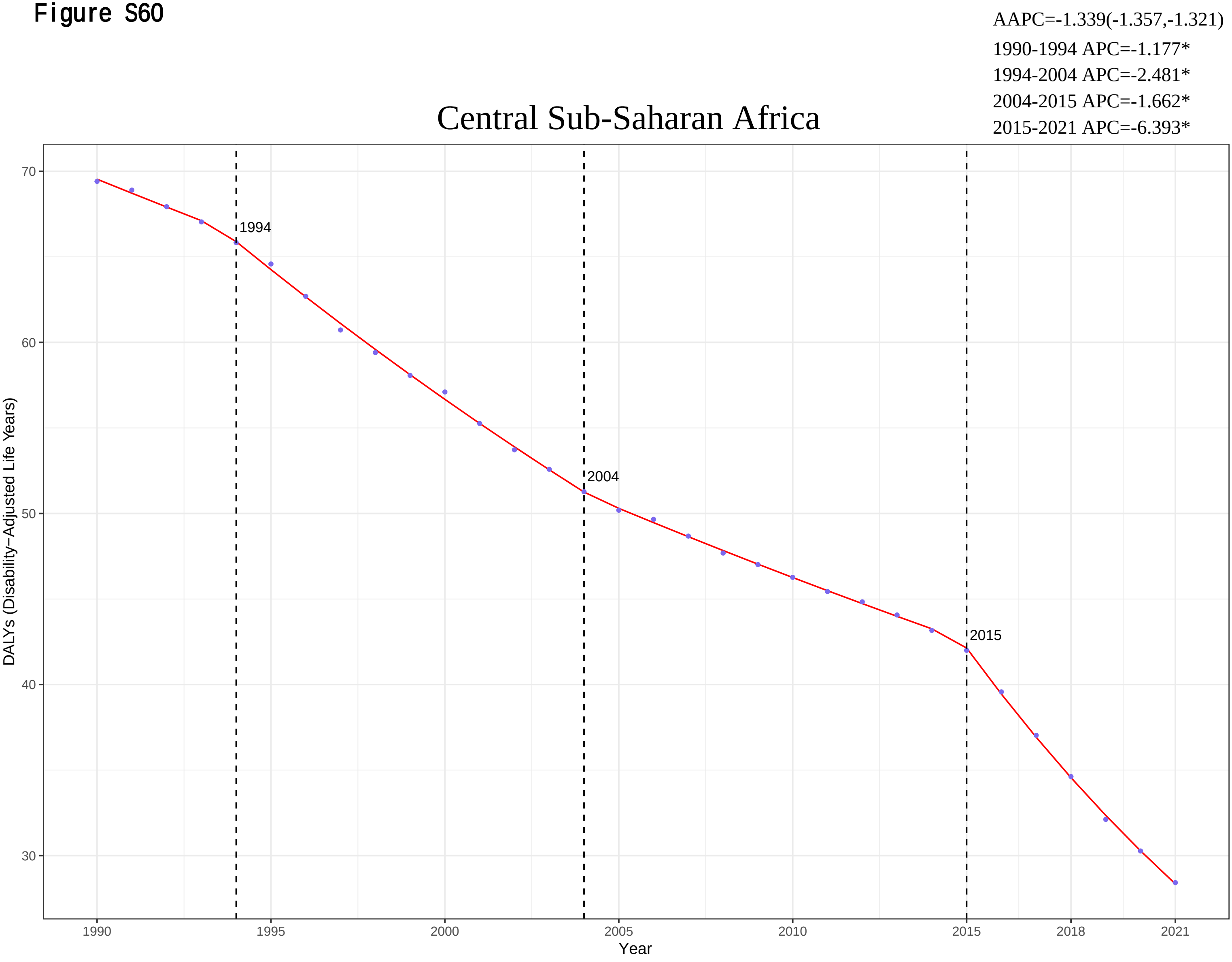
AAPC=-1.339(-1.357,-1.321)
1990-1994 APC=-1.177*
1994-2004 APC=-2.481*
2004-2015 APC=-1.662*
2015-2021 APC=-6.393*
Central Sub-Saharan Africa
70
60
50
40
30
DALYs (Disability−Adjusted Life Years)
1994
2004
2015
1990
1995
2000
2005
2010
2015
2018
2021
Year

Figure S61

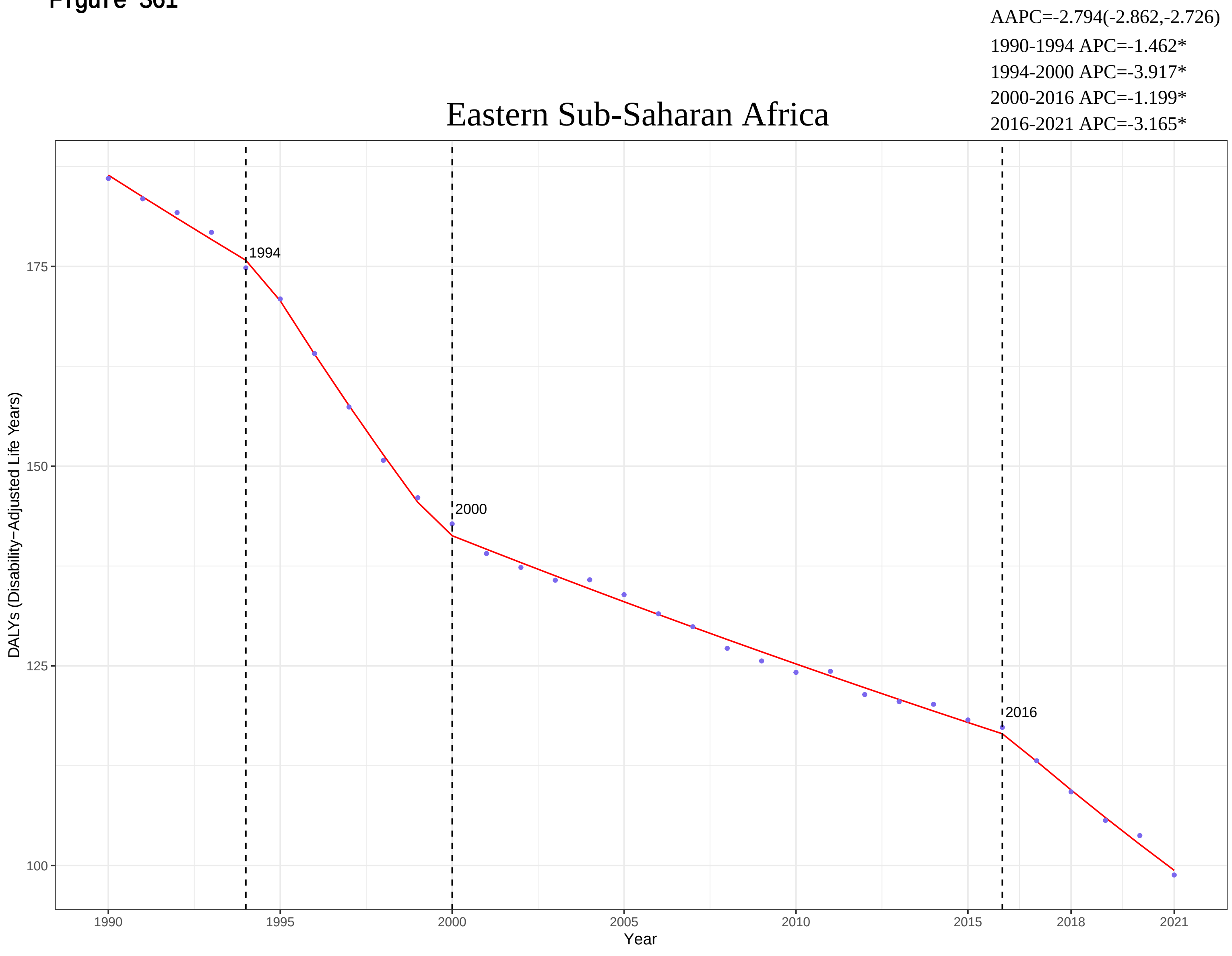
AAPC=-2.794(-2.862,-2.726)
1990-1994 APC=-1.462*
1994-2000 APC=-3.917*
2000-2016 APC=-1.199*
2016-2021 APC=-3.165*
Eastern Sub-Saharan Africa
DALYs (Disability−Adjusted Life Years)
175
150
125
100
1994
2000
2016
1990
1995
2000
2005
2010
2015
2018
2021
Year

Figure S62

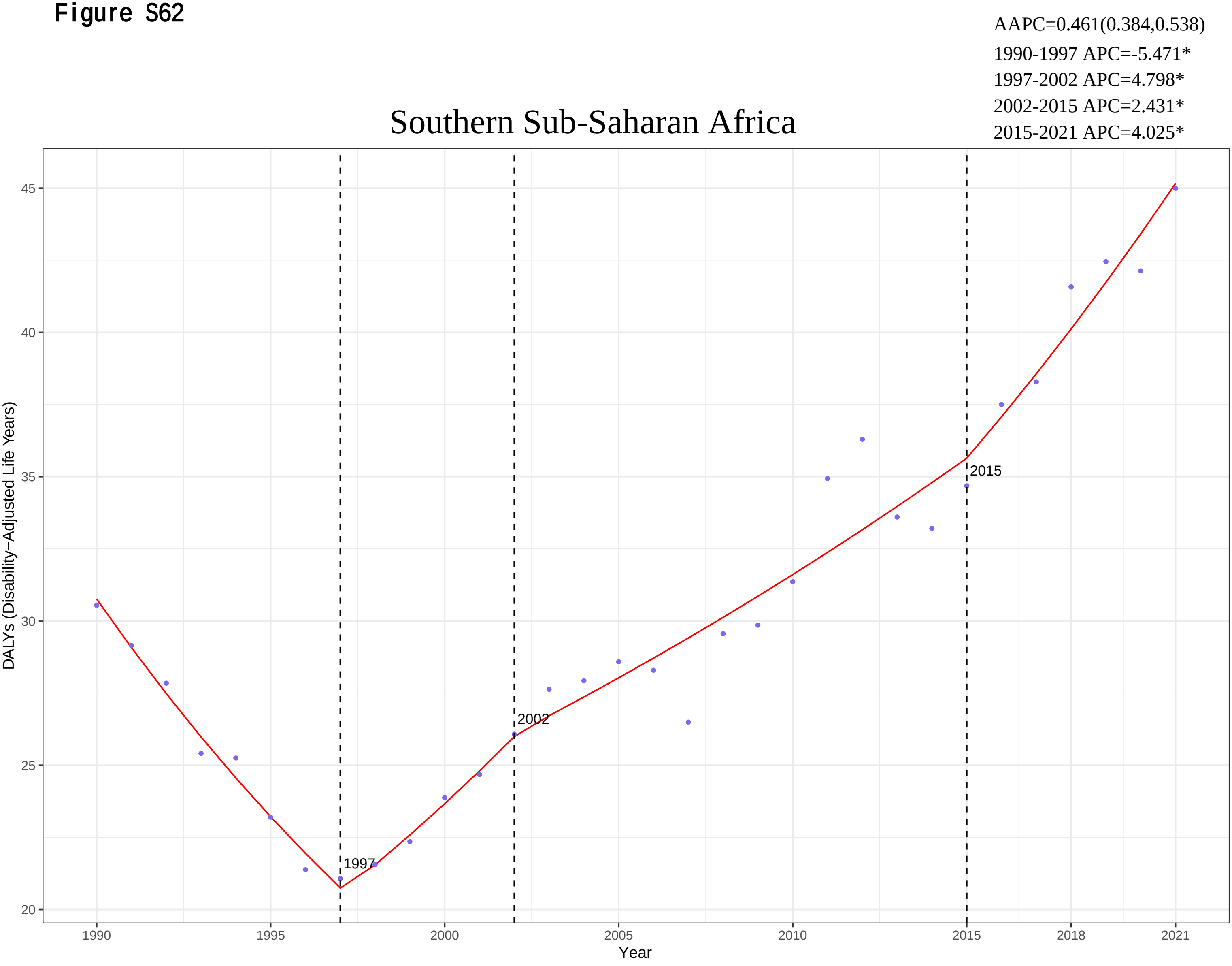
AAPC=0.461(0.384,0.538)
1990-1997 APC=-5.471*
1997-2002 APC=4.798*
2002-2015 APC=2.431*
2015-2021 APC=4.025*
Southern Sub-Saharan Africa
DALYs (Disability−Adjusted Life Years)
45
40
35
30
25
20
1997
2002
2015
1990
1995
2000
2005
2010
2015
2018
2021
Year

Figure S63

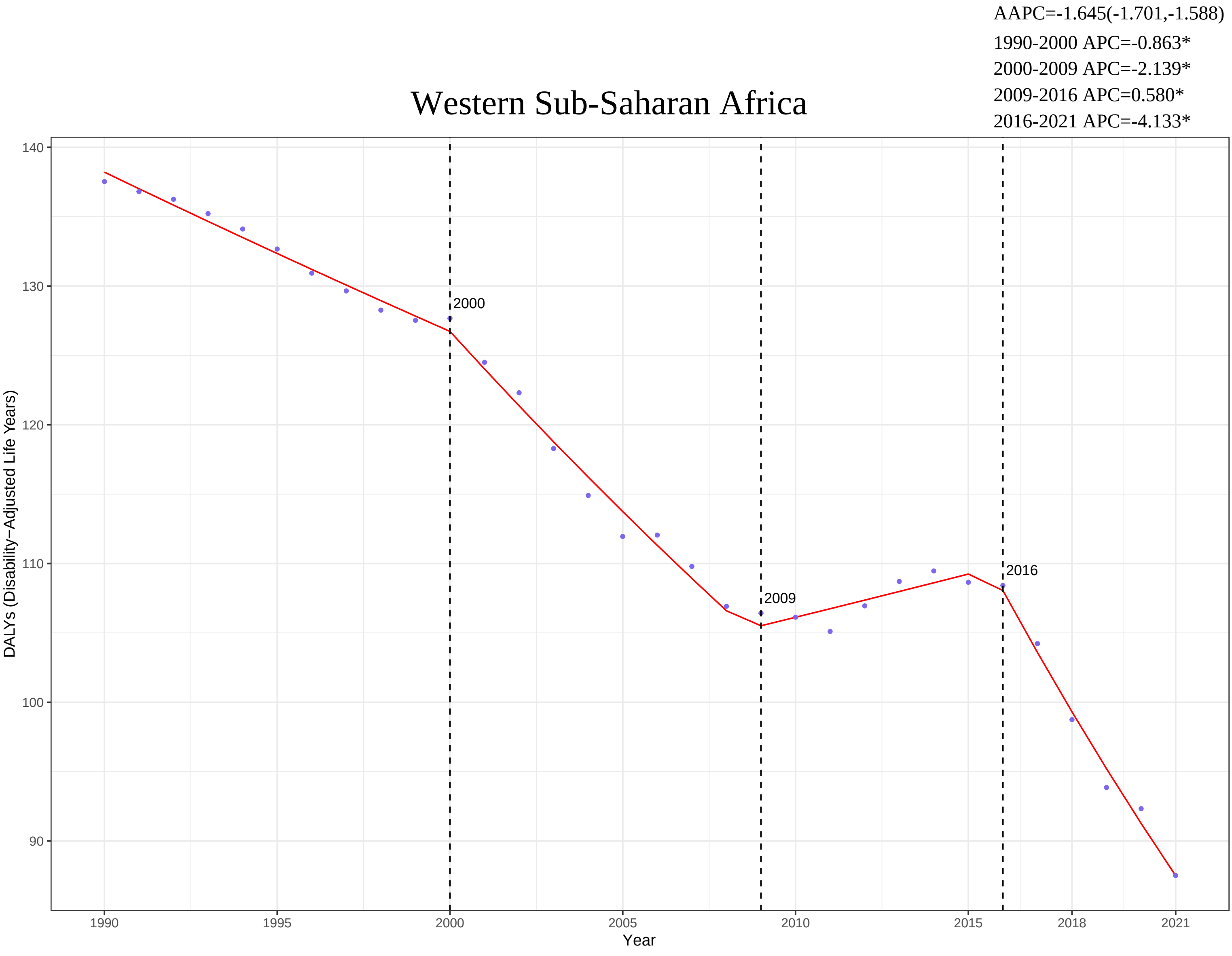
AAPC=-1.645(-1.701,-1.588)
1990-2000 APC=-0.863*
2000-2009 APC=-2.139*
2009-2016 APC=0.580*
2016-2021 APC=-4.133*
Western Sub-Saharan Africa
DALYs (Disability−Adjusted Life Years)
140
130
120
110
100
90
2000
2009
2016
1990
1995
2000
2005
2010
2015
2018
2021
Year

Figure S64

A

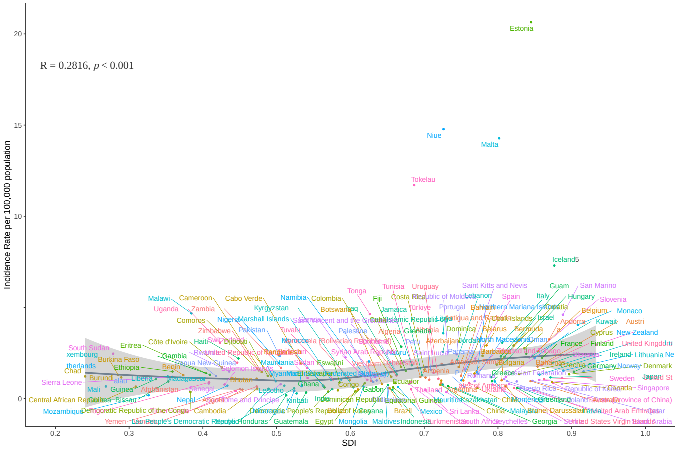

B

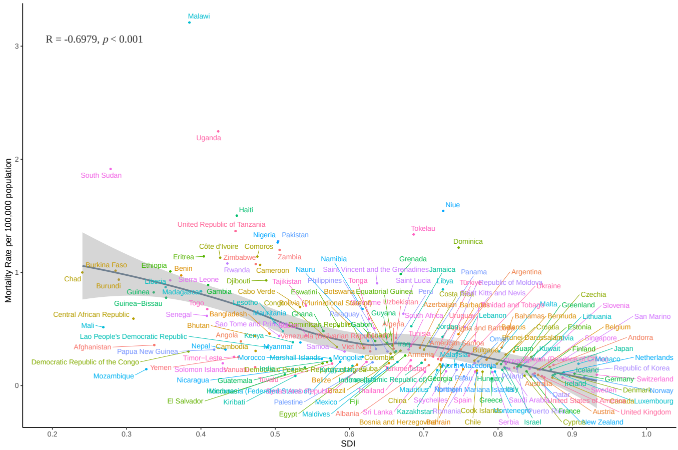

C

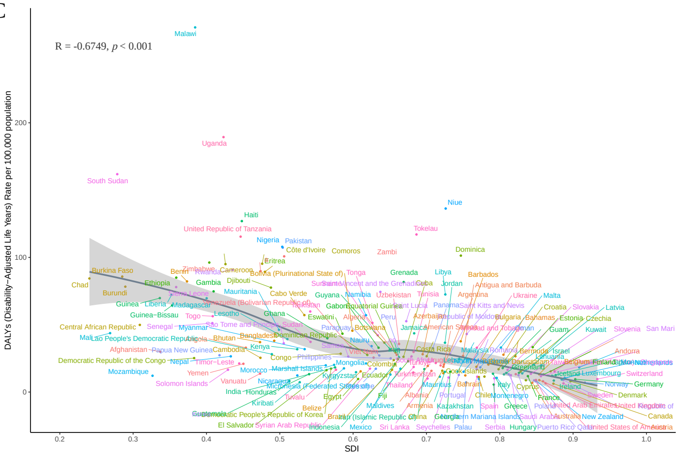

Figure S65

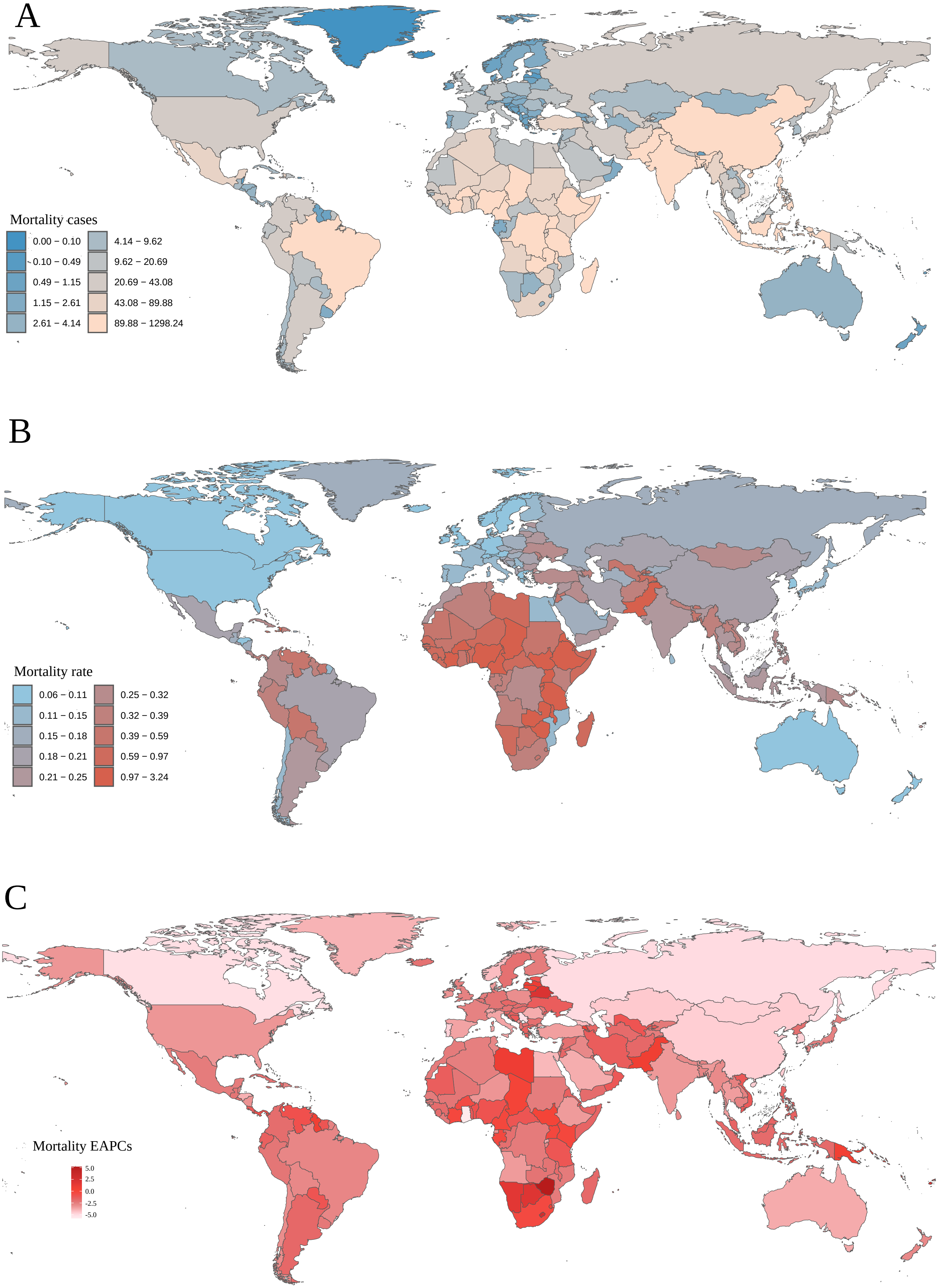
A
Mortality cases
0.00 − 0.10
0.10 − 0.49
0.49 − 1.15
1.15 − 2.61
2.61 − 4.14
4.14 − 9.62
9.62 − 20.69
20.69 − 43.08
43.08 − 89.88
89.88 − 1298.24
B
Mortality rate
0.06 − 0.11
0.11 − 0.15
0.15 − 0.18
0.18 − 0.21
0.21 − 0.25
0.25 − 0.32
0.32 − 0.39
0.39 − 0.59
0.59 − 0.97
0.97 − 3.24
C
Mortality EAPCs
5.0
2.5
0.0
-2.5
-5.0

Figure S66

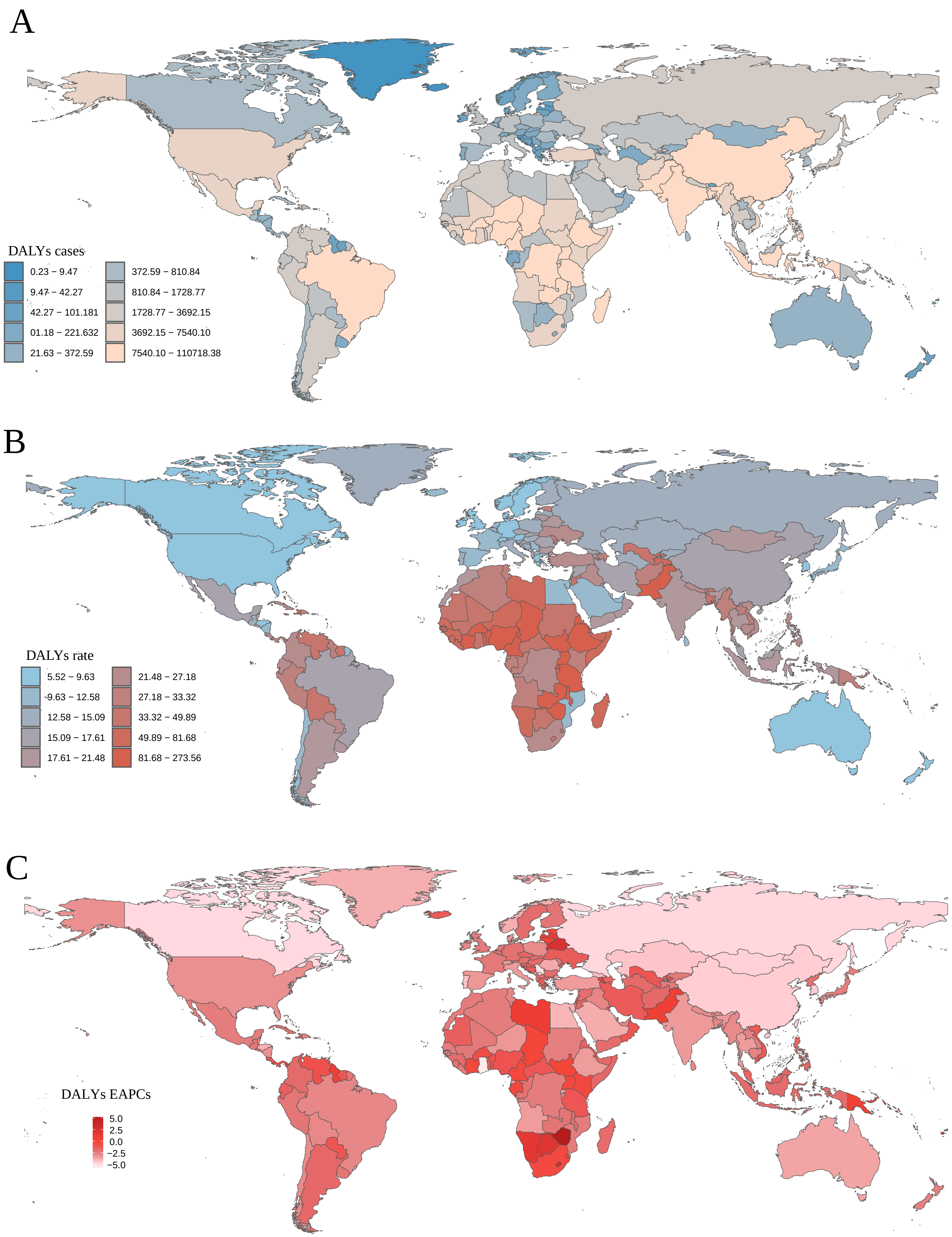
A
DALYs cases
0.23 − 9.47
9.47 − 42.27
42.27 − 101.181
01.18 − 221.632
21.63 − 372.59
372.59 − 810.84
810.84 − 1728.77
1728.77 − 3692.15
3692.15 − 7540.10
7540.10 − 110718.38
B
DALYs rate
5.52 − 9.63
9.63 − 12.58
12.58 − 15.09
15.09 − 17.61
17.61 − 21.48
21.48 − 27.18
27.18 − 33.32
33.32 − 49.89
49.89 − 81.68
81.68 − 273.56
C
DALYs EAPCs
5.0
2.5
0.0
−2.5
−5.0
