# Supplementary material for: Global burden and trends of childhood non-Hodgkin lymphoma from 1990 to 2021
Source: Front Pediatr. 2025 Jun 26;13:1618810. doi: 10.3389/fped.2025.1618810 (PMC12241043; doi:10.3389/fped.2025.1618810)
Supplement: Supplementary file 2 [file Table2.docx]

**Table S1: Mortality of Non-Hodgkin Lymphoma in children between 1990 and 2021 at the global and regional level.**

| Location | Number (95% UI) | | Percentage change (95% UI) | Rate (95% UI) | | EAPC (95% CI) |
| --- | --- | --- | --- | --- | --- | --- |
|  | 1990 | 2021 |  | 1990 | 2021 |  |
| Global | 12990.00(9904.43,15509.02) | 9036.49(7197.45,11251.98) | -30.43(-43.66,6.80) | 0.75(0.57,0.89) | 0.45(0.36,0.56) | -1.49(-1.60,-1.39) |
| Low SDI | 3622.77(2370.30,4792.28) | 4157.48(2868.69,5570.47) | 14.76(-12.81,108.52) | 1.58(1.04,2.09) | 0.90(0.62,1.21) | -1.68(-1.78,-1.57) |
| Low-middle SDI | 3626.63(2446.64,4617.89) | 2750.39(2217.99,3493.93) | -24.16(-42.56,27.72) | 0.77(0.52,0.98) | 0.47(0.38,0.60) | -1.41(-1.50,-1.32) |
| Middle SDI | 3591.85(3029.39,4179.98) | 1499.26(1233.04,1855.05) | -58.26(-65.61,-43.72) | 0.62(0.52,0.72) | 0.26(0.22,0.33) | -2.59(-2.76,-2.41) |
| High-middle SDI | 1633.11(1422.84,1867.51) | 439.50(380.19,519.97) | -73.09(-77.21,-66.26) | 0.60(0.52,0.68) | 0.19(0.16,0.23) | -3.69(-3.88,-3.51) |
| High SDI | 505.04(481.15,530.35) | 182.62(169.57,196.35) | -63.84(-66.22,-61.23) | 0.27(0.26,0.29) | 0.11(0.10,0.11) | -3.12(-3.21,-3.03) |
| East Asia | 2454.63(2046.08,2989.85) | 503.68(406.26,645.39) | -79.48(-84.12,-71.76) | 0.74(0.62,0.91) | 0.19(0.15,0.24) | -4.47(-4.82,-4.12) |
| Southeast Asia | 879.41(560.12,1168.28) | 472.77(383.69,638.70) | -46.24(-57.31,-17.87) | 0.52(0.33,0.68) | 0.27(0.22,0.37) | -1.93(-1.99,-1.87) |
| Oceania | 6.86(4.41,9.96) | 15.32(10.16,22.75) | 123.32(58.92,207.92) | 0.26(0.16,0.37) | 0.30(0.20,0.45) | 0.72(0.49,0.95) |
| Central Asia | 159.40(130.76,185.09) | 97.61(80.56,120.99) | -38.76(-51.83,-22.07) | 0.64(0.52,0.74) | 0.35(0.29,0.44) | -2.02(-2.21,-1.84) |
| Central Europe | 126.64(119.22,134.40) | 30.14(27.25,33.40) | -76.20(-78.62,-73.19) | 0.43(0.40,0.46) | 0.17(0.15,0.19) | -2.87(-3.15,-2.60) |
| Eastern Europe | 303.94(288.01,316.97) | 67.14(62.54,72.41) | -77.91(-79.82,-75.27) | 0.59(0.56,0.62) | 0.19(0.18,0.20) | -3.79(-4.20,-3.37) |
| High-income Asia Pacific | 119.96(109.41,135.53) | 29.51(27.51,31.79) | -75.40(-78.47,-72.11) | 0.34(0.31,0.39) | 0.13(0.12,0.14) | -3.26(-3.49,-3.02) |
| Australasia | 11.26(10.06,12.64) | 4.54(3.78,5.33) | -59.63(-67.52,-51.22) | 0.25(0.22,0.28) | 0.08(0.07,0.09) | -3.57(-4.12,-3.02) |
| Western Europe | 183.77(177.22,190.86) | 76.83(70.61,83.16) | -58.19(-61.93,-54.47) | 0.26(0.25,0.27) | 0.11(0.10,0.12) | -2.83(-2.98,-2.67) |
| Southern Latin America | 60.70(55.33,66.99) | 29.19(24.82,34.24) | -51.91(-60.49,-41.89) | 0.41(0.37,0.45) | 0.20(0.17,0.24) | -1.95(-2.25,-1.64) |
| High-income North America | 120.93(118.02,124.24) | 47.27(43.95,50.38) | -60.91(-63.74,-58.28) | 0.20(0.19,0.20) | 0.07(0.07,0.08) | -3.39(-3.57,-3.21) |
| Caribbean | 141.92(84.98,192.00) | 91.68(55.17,131.85) | -35.40(-52.69,-14.08) | 1.24(0.74,1.68) | 0.80(0.48,1.15) | -1.06(-1.33,-0.79) |
| Andean Latin America | 119.37(99.87,148.00) | 70.20(56.06,90.20) | -41.19(-55.57,-20.93) | 0.80(0.67,1.00) | 0.39(0.31,0.50) | -2.20(-2.38,-2.01) |
| Central Latin America | 336.69(315.01,360.91) | 148.67(127.00,175.72) | -55.84(-62.84,-48.06) | 0.52(0.49,0.56) | 0.23(0.20,0.28) | -2.08(-2.28,-1.89) |
| Tropical Latin America | 296.27(264.35,328.36) | 102.23(82.96,120.27) | -65.50(-71.53,-58.58) | 0.55(0.49,0.61) | 0.20(0.17,0.24) | -2.67(-3.12,-2.21) |
| North Africa and Middle East | 830.13(618.27,1104.17) | 469.17(389.87,610.37) | -43.48(-58.02,-20.21) | 0.59(0.44,0.79) | 0.26(0.21,0.33) | -2.44(-2.64,-2.24) |
| South Asia | 3177.06(1996.32,4138.23) | 2139.59(1680.09,2795.58) | -32.66(-51.99,27.34) | 0.73(0.46,0.95) | 0.42(0.33,0.55) | -1.71(-1.83,-1.58) |
| Central Sub-Saharan Africa | 204.86(63.28,302.80) | 198.83(134.58,274.79) | -2.94(-32.26,151.86) | 0.81(0.25,1.20) | 0.34(0.23,0.47) | -2.45(-2.65,-2.25) |
| Eastern Sub-Saharan Africa | 1971.94(1397.50,2650.84) | 2094.62(1418.15,2964.12) | 6.22(-23.14,90.58) | 2.18(1.54,2.93) | 1.17(0.79,1.66) | -1.80(-1.93,-1.67) |
| Southern Sub-Saharan Africa | 75.08(59.79,93.97) | 129.99(92.28,166.96) | 73.14(24.58,131.95) | 0.36(0.29,0.45) | 0.54(0.38,0.69) | 1.90(1.44,2.36) |
| Western Sub-Saharan Africa | 1409.17(993.63,1865.93) | 2217.51(1423.04,2946.83) | 57.36(18.93,139.11) | 1.60(1.13,2.12) | 1.03(0.66,1.37) | -1.24(-1.37,-1.11) |

Abbreviations: EAPC, estimated annual percentage change; SDI, Sociodemographic Index; UI, uncertainty interval; CI, confidence interval

**Table S2: Disability-adjusted life years of Non-Hodgkin Lymphoma in children between 1990 and 2021 at the global and regional level.**

| Location | Number (95% UI) | | Percentage change (95% UI) | Rate(95% UI) | | EAPC (95% CI) |
| --- | --- | --- | --- | --- | --- | --- |
|  | 1990 | 2021 |  | 1990 | 2021 |  |
| Global | 1102346.32(836303.95,1316917.95) | 761085.36(602606.50,948912.73) | -30.96(-44.26,6.73) | 63.38(48.09,75.72) | 37.83(29.95,47.17) | -1.51(-1.61,-1.40) |
| Low SDI | 309591.61(201695.81,409472.51) | 350984.47(240530.35,471925.94) | 13.37(-14.21,107.39) | 135.24(88.11,178.88) | 76.26(52.26,102.54) | -1.71(-1.81,-1.60) |
| Low-middle SDI | 306898.12(205627.98,391484.01) | 230248.63(185145.73,293375.42) | -24.98(-43.32,27.79) | 65.01(43.55,82.92) | 39.71(31.93,50.60) | -1.44(-1.53,-1.35) |
| Middle SDI | 303674.44(255665.33,353037.50) | 125629.47(103248.82,155971.70) | -58.63(-66.03,-43.73) | 52.61(44.29,61.16) | 22.16(18.21,27.51) | -2.60(-2.78,-2.43) |
| High-middle SDI | 138345.40(119944.22,158628.66) | 37408.44(32186.93,44297.50) | -72.96(-77.16,-66.08) | 50.56(43.84,57.97) | 16.20(13.94,19.19) | -3.65(-3.84,-3.47) |
| High SDI | 42938.50(40791.48,45092.95) | 16201.97(14995.59,17393.34) | -62.27(-64.82,-59.58) | 23.11(21.95,24.27) | 9.39(8.69,10.08) | -2.96(-3.05,-2.87) |
| East Asia | 209159.50(173974.04,255126.65) | 42491.44(34061.03,54539.66) | -79.68(-84.31,-71.97) | 63.41(52.75,77.35) | 15.89(12.74,20.40) | -4.48(-4.83,-4.12) |
| Southeast Asia | 73783.18(46430.36,98374.35) | 39411.06(31914.86,53265.23) | -46.59(-57.61,-17.69) | 43.21(27.19,57.61) | 22.83(18.48,30.85) | -1.95(-2.01,-1.88) |
| Oceania | 589.08(378.09,854.00) | 1318.87(870.08,1963.71) | 123.89(59.16,206.95) | 21.98(14.11,31.87) | 25.96(17.12,38.65) | 0.73(0.50,0.96) |
| Central Asia | 13504.65(11053.23,15688.07) | 8243.72(6808.41,10244.58) | -38.96(-52.44,-22.13) | 54.04(44.23,62.77) | 29.79(24.60,37.02) | -2.01(-2.20,-1.83) |
| Central Europe | 10612.93(9989.53,11244.68) | 2578.10(2333.22,2855.72) | -75.71(-78.29,-72.55) | 36.00(33.88,38.14) | 14.56(13.18,16.13) | -2.79(-3.06,-2.52) |
| Eastern Europe | 25740.17(24460.27,26923.32) | 5748.47(5342.40,6223.71) | -77.67(-79.60,-75.00) | 50.02(47.53,52.32) | 16.22(15.07,17.56) | -3.70(-4.13,-3.27) |
| High-income Asia Pacific | 9952.21(9087.41,11209.30) | 2527.38(2347.43,2728.34) | -74.60(-77.59,-71.32) | 28.27(25.82,31.85) | 11.27(10.47,12.17) | -3.13(-3.35,-2.90) |
| Australasia | 972.43(862.91,1094.07) | 409.97(336.55,484.22) | -57.84(-66.45,-48.63) | 21.20(18.82,23.86) | 7.15(5.87,8.45) | -3.43(-3.99,-2.86) |
| Western Europe | 15928.12(15288.53,16594.16) | 7114.41(6543.20,7744.55) | -55.33(-59.23,-51.27) | 22.43(21.53,23.37) | 10.44(9.61,11.37) | -2.60(-2.76,-2.43) |
| Southern Latin America | 5080.94(4633.35,5597.39) | 2452.92(2085.22,2873.45) | -51.72(-60.42,-41.56) | 34.04(31.04,37.50) | 16.92(14.39,19.82) | -1.93(-2.23,-1.62) |
| High-income North America | 10433.52(10115.12,10806.48) | 4243.12(3922.81,4581.94) | -59.33(-62.45,-56.35) | 16.92(16.40,17.52) | 6.47(5.98,6.98) | -3.24(-3.41,-3.08) |
| Caribbean | 12052.53(7142.76,16393.66) | 7730.79(4566.73,11149.79) | -35.86(-53.33,-14.33) | 105.61(62.59,143.65) | 67.19(39.69,96.91) | -1.08(-1.35,-0.81) |
| Andean Latin America | 10031.91(8381.31,12474.55) | 5857.70(4682.62,7553.76) | -41.61(-55.91,-21.15) | 67.55(56.43,83.99) | 32.37(25.88,41.75) | -2.22(-2.40,-2.03) |
| Central Latin America | 28353.04(26468.32,30385.17) | 12411.28(10568.73,14725.47) | -56.23(-63.19,-48.39) | 44.04(41.11,47.20) | 19.55(16.65,23.20) | -2.09(-2.30,-1.89) |
| Tropical Latin America | 24912.55(22230.82,27647.58) | 8500.52(6874.04,10030.48) | -65.88(-71.97,-59.04) | 46.47(41.46,51.57) | 16.94(13.70,19.98) | -2.70(-3.15,-2.24) |
| North Africa and Middle East | 69726.00(51695.85,93026.52) | 39550.74(32870.68,51394.60) | -43.28(-58.02,-19.33) | 49.63(36.80,66.22) | 21.57(17.93,28.04) | -2.41(-2.61,-2.21) |
| South Asia | 268297.54(167728.63,350213.37) | 178714.33(140209.04,233215.82) | -33.39(-52.76,27.75) | 61.91(38.70,80.81) | 35.25(27.65,46.00) | -1.74(-1.86,-1.61) |
| Central Sub-Saharan Africa | 17560.51(5257.12,25953.29) | 16677.96(11189.67,23091.16) | -5.03(-34.13,151.98) | 69.41(20.78,102.59) | 28.42(19.07,39.35) | -2.51(-2.72,-2.30) |
| Eastern Sub-Saharan Africa | 168477.30(119364.46,226748.53) | 176326.64(118574.95,250699.21) | 4.66(-24.59,89.59) | 186.02(131.79,250.36) | 98.82(66.45,140.50) | -1.84(-1.97,-1.71) |
| Southern Sub-Saharan Africa | 6319.04(4995.97,7927.51) | 10827.95(7669.36,13967.35) | 71.35(22.80,129.62) | 30.54(24.15,38.32) | 44.99(31.87,58.04) | 1.91(1.43,2.40) |
| Western Sub-Saharan Africa | 120859.18(84772.51,160450.53) | 187947.98(119464.74,250299.69) | 55.51(16.98,138.12) | 137.53(96.46,182.58) | 87.51(55.63,116.55) | -1.27(-1.40,-1.14) |

Abbreviations: EAPC, estimated annual percentage change; SDI, Sociodemographic Index; UI, uncertainty interval; CI, confidence interval

**Table S3: Incidence of Non-Hodgkin Lymphoma in children between 1990 and 2021 in 204 countries.**

| location | Number (95% UI) | | Percentage change (95% UI) | Rate (95% UI) | | EAPC (95% CI) |
| --- | --- | --- | --- | --- | --- | --- |
|  | 1990 | 2021 |  | 1990 | 2021 |  |
| People's Republic of China | 3625.27(2990.85,4465.12) | 1985.21(1562.60,2570.22) | -45.24(-58.17,-22.02) | 1.14(0.94,1.40) | 0.76(0.60,0.99) | -1.17(-1.59,-0.75) |
| Democratic People's Republic of Korea | 32.63(20.45,47.92) | 18.24(11.85,29.76) | -44.10(-64.70,-8.44) | 0.55(0.34,0.81) | 0.38(0.25,0.62) | -0.83(-1.10,-0.56) |
| Taiwan (Province of China) | 43.89(37.67,51.51) | 25.00(19.01,31.84) | -43.05(-57.89,-24.75) | 0.80(0.68,0.94) | 0.85(0.65,1.08) | 0.15(-0.32,0.62) |
| Kingdom of Cambodia | 33.63(14.65,49.44) | 28.10(19.01,45.92) | -16.44(-48.97,88.54) | 0.72(0.31,1.06) | 0.55(0.37,0.90) | -1.23(-1.49,-0.97) |
| Republic of Indonesia | 394.24(217.08,597.31) | 321.73(226.23,503.68) | -18.39(-40.97,29.10) | 0.58(0.32,0.88) | 0.48(0.34,0.75) | -0.60(-0.66,-0.54) |
| Lao People's Democratic Republic | 15.58(5.54,24.51) | 12.34(7.83,20.14) | -20.82(-50.64,87.64) | 0.85(0.30,1.33) | 0.54(0.34,0.88) | -1.52(-1.59,-1.45) |
| Malaysia | 33.82(23.67,45.77) | 42.65(28.47,60.77) | 26.13(-25.77,104.98) | 0.51(0.36,0.70) | 0.56(0.37,0.80) | 0.75(0.42,1.09) |
| Republic of Maldives | 0.59(0.28,0.87) | 0.57(0.36,0.86) | -4.55(-48.63,159.70) | 0.56(0.27,0.83) | 0.57(0.36,0.86) | 0.72(0.42,1.01) |
| Republic of the Union of Myanmar | 148.31(51.56,230.62) | 105.97(66.91,163.94) | -28.55(-53.18,38.41) | 1.00(0.35,1.56) | 0.68(0.43,1.05) | -1.54(-1.70,-1.38) |
| Republic of the Philippines | 199.85(133.81,256.85) | 201.13(153.09,271.56) | 0.64(-27.24,48.18) | 0.79(0.53,1.02) | 0.59(0.45,0.80) | -0.61(-0.76,-0.46) |
| Democratic Socialist Republic of Sri Lanka | 24.69(17.69,32.61) | 23.47(14.76,34.15) | -4.95(-39.68,54.66) | 0.45(0.32,0.59) | 0.46(0.29,0.67) | 0.20(-0.42,0.83) |
| Kingdom of Thailand | 147.48(109.46,201.05) | 89.22(59.38,139.15) | -39.51(-61.92,-7.76) | 0.87(0.65,1.19) | 0.91(0.61,1.42) | -0.53(-0.94,-0.11) |
| Democratic Republic of Timor-Leste | 2.41(0.91,3.86) | 2.17(1.46,3.45) | -10.11(-42.35,105.31) | 0.73(0.27,1.16) | 0.42(0.28,0.66) | -1.86(-2.24,-1.47) |
| Socialist Republic of Viet Nam | 178.56(121.26,246.25) | 241.76(155.21,352.55) | 35.40(-17.79,130.79) | 0.67(0.46,0.93) | 0.98(0.63,1.42) | 1.50(1.39,1.60) |
| Republic of Fiji | 1.86(1.16,2.92) | 4.15(2.41,6.75) | 123.47(14.07,350.86) | 0.66(0.41,1.04) | 1.52(0.89,2.48) | 2.81(2.60,3.01) |
| Republic of Kiribati | 0.08(0.04,0.11) | 0.12(0.06,0.20) | 51.49(-19.75,173.23) | 0.26(0.14,0.38) | 0.28(0.13,0.48) | -0.03(-0.31,0.24) |
| Republic of the Marshall Islands | 0.09(0.06,0.14) | 0.18(0.10,0.31) | 96.50(-0.51,311.60) | 0.41(0.26,0.65) | 1.01(0.58,1.79) | 2.82(2.45,3.18) |
| Federated States of Micronesia | 0.31(0.19,0.50) | 0.32(0.18,0.54) | 1.67(-47.15,115.53) | 0.68(0.40,1.10) | 1.04(0.58,1.77) | 1.50(1.28,1.72) |
| Independent State of Papua New Guinea | 14.89(7.62,23.80) | 48.35(26.49,78.30) | 224.60(85.53,434.56) | 0.88(0.45,1.40) | 1.23(0.68,2.00) | 1.06(0.83,1.28) |
| Independent State of Samoa | 1.43(0.89,2.13) | 1.80(1.00,3.21) | 26.39(-28.79,126.56) | 2.00(1.25,2.99) | 2.26(1.25,4.01) | 0.18(-0.03,0.38) |
| Solomon Islands | 0.65(0.34,1.05) | 2.00(1.25,3.17) | 207.65(66.57,523.15) | 0.42(0.22,0.67) | 0.77(0.48,1.22) | 1.96(1.63,2.30) |
| Kingdom of Tonga | 1.17(0.71,1.79) | 1.81(1.02,3.20) | 54.80(-20.82,228.78) | 2.79(1.70,4.28) | 4.63(2.63,8.21) | 1.02(0.55,1.50) |
| Republic of Vanuatu | 0.27(0.16,0.43) | 0.77(0.48,1.20) | 183.11(50.12,431.71) | 0.40(0.23,0.64) | 0.66(0.41,1.03) | 1.78(1.36,2.20) |
| Republic of Armenia | 7.92(5.42,10.24) | 6.82(4.79,9.58) | -13.89(-43.84,47.85) | 0.76(0.52,0.98) | 1.15(0.81,1.62) | 3.12(2.46,3.78) |
| Republic of Azerbaijan | 22.42(15.19,31.84) | 28.07(16.60,42.85) | 25.22(-31.43,134.75) | 0.92(0.63,1.31) | 1.19(0.70,1.82) | 0.43(0.19,0.67) |
| Georgia | 26.31(18.03,36.49) | 5.01(3.37,7.15) | -80.98(-88.84,-66.88) | 1.92(1.32,2.67) | 0.68(0.46,0.97) | -3.44(-4.17,-2.71) |
| Republic of Kazakhstan | 78.81(63.83,97.87) | 41.73(30.43,56.78) | -47.05(-64.48,-21.47) | 1.52(1.23,1.88) | 0.77(0.56,1.05) | -1.93(-2.58,-1.28) |
| Kyrgyz Republic | 11.88(8.85,16.26) | 13.93(9.80,19.55) | 17.31(-29.52,89.81) | 0.71(0.53,0.97) | 0.61(0.43,0.86) | -0.38(-0.96,0.19) |
| Mongolia | 12.93(8.31,18.07) | 7.18(4.82,10.35) | -44.42(-66.59,-1.42) | 1.44(0.92,2.01) | 0.66(0.44,0.95) | -2.51(-3.14,-1.89) |
| Republic of Tajikistan | 51.72(23.79,75.88) | 60.09(38.72,89.36) | 16.19(-35.82,144.22) | 2.23(1.02,3.27) | 1.68(1.08,2.49) | -1.66(-2.14,-1.18) |
| Turkmenistan | 7.14(5.79,8.69) | 7.80(5.87,10.15) | 9.20(-23.12,55.45) | 0.48(0.39,0.58) | 0.51(0.39,0.67) | -0.31(-0.83,0.22) |
| Republic of Uzbekistan | 105.34(71.85,145.07) | 127.83(92.69,173.90) | 21.35(-26.14,104.76) | 1.23(0.84,1.70) | 1.27(0.92,1.72) | 0.42(0.18,0.66) |
| Republic of Albania | 9.27(6.54,12.45) | 4.58(2.91,7.01) | -50.62(-69.80,-17.09) | 0.83(0.59,1.11) | 1.03(0.66,1.58) | 1.44(0.34,2.56) |
| Bosnia and Herzegovina | 7.18(5.21,9.55) | 4.82(2.96,7.00) | -32.87(-61.68,10.94) | 0.66(0.48,0.87) | 0.98(0.60,1.43) | 2.10(1.56,2.65) |
| Republic of Bulgaria | 21.89(18.11,26.89) | 13.38(9.90,18.00) | -38.88(-56.99,-10.62) | 1.26(1.04,1.55) | 1.37(1.01,1.84) | 0.41(-0.06,0.88) |
| Republic of Croatia | 15.80(11.49,21.08) | 11.10(7.56,15.86) | -29.71(-57.13,16.77) | 1.60(1.16,2.14) | 1.86(1.27,2.66) | 1.24(0.60,1.88) |
| Czech Republic | 23.35(17.84,29.90) | 23.17(15.68,32.42) | -0.77(-37.19,55.43) | 1.06(0.81,1.36) | 1.35(0.91,1.89) | 1.12(0.77,1.46) |
| Hungary | 23.54(18.33,29.98) | 16.23(12.04,22.41) | -31.07(-53.31,4.22) | 1.10(0.86,1.41) | 1.17(0.87,1.61) | 0.91(0.48,1.35) |
| North Macedonia | 5.49(4.13,7.31) | 3.29(2.06,4.77) | -39.98(-66.15,-3.18) | 1.04(0.78,1.39) | 1.01(0.63,1.46) | 0.96(0.42,1.50) |
| Montenegro | 2.81(2.05,3.65) | 1.04(0.68,1.59) | -62.83(-77.56,-33.19) | 1.74(1.27,2.26) | 0.94(0.61,1.43) | -1.35(-1.96,-0.74) |
| Republic of Poland | 81.22(71.74,92.21) | 56.49(44.51,72.78) | -30.44(-48.15,-8.78) | 0.85(0.75,0.96) | 0.96(0.76,1.24) | 0.46(-0.20,1.14) |
| Romania | 69.44(57.70,85.92) | 28.66(22.16,36.88) | -58.73(-69.33,-42.10) | 1.25(1.04,1.54) | 0.95(0.74,1.23) | -0.77(-1.18,-0.35) |
| Republic of Serbia | 37.46(24.00,52.50) | 10.27(6.61,15.45) | -72.59(-84.22,-44.38) | 1.73(1.11,2.42) | 0.77(0.50,1.16) | -3.00(-3.49,-2.50) |
| Slovak Republic | 12.59(9.13,16.99) | 10.15(6.90,14.81) | -19.38(-50.45,30.43) | 0.95(0.69,1.28) | 1.18(0.81,1.73) | 0.76(0.35,1.19) |
| Republic of Slovenia | 5.95(4.42,7.60) | 7.04(4.84,9.70) | 18.35(-22.88,85.64) | 1.44(1.07,1.84) | 2.25(1.55,3.11) | 2.66(2.15,3.18) |
| Republic of Belarus | 22.80(16.85,31.26) | 45.99(31.36,66.62) | 101.72(21.24,229.96) | 0.95(0.70,1.30) | 2.91(1.99,4.22) | 5.19(4.46,5.93) |
| Republic of Estonia | 31.04(20.93,44.07) | 44.62(28.91,66.19) | 43.76(-12.80,136.81) | 8.89(5.99,12.62) | 20.64(13.38,30.62) | 2.94(2.11,3.78) |
| Republic of Latvia | 2.59(2.12,3.13) | 1.69(1.25,2.21) | -34.55(-54.45,-7.32) | 0.45(0.37,0.55) | 0.57(0.42,0.75) | 2.46(1.76,3.17) |
| Republic of Lithuania | 5.08(3.80,6.78) | 5.37(3.83,7.56) | 5.63(-31.54,58.88) | 0.61(0.46,0.82) | 1.32(0.94,1.85) | 1.95(1.34,2.56) |
| Republic of Moldova | 50.79(37.57,68.57) | 13.42(9.77,18.10) | -73.57(-82.62,-59.02) | 4.11(3.04,5.55) | 2.57(1.87,3.46) | -0.64(-1.33,0.06) |
| Russian Federation | 868.37(827.23,911.97) | 277.92(254.93,301.10) | -67.99(-71.26,-64.73) | 2.50(2.38,2.63) | 1.07(0.98,1.15) | -2.88(-3.65,-2.11) |
| Ukraine | 119.62(78.00,164.20) | 54.57(41.65,71.98) | -54.38(-70.64,-19.99) | 1.05(0.69,1.44) | 0.86(0.66,1.13) | -0.94(-1.45,-0.43) |
| Brunei Darussalam | 0.78(0.56,1.05) | 0.65(0.47,0.85) | -17.10(-44.53,33.18) | 0.86(0.62,1.16) | 0.68(0.50,0.89) | 0.39(-0.14,0.93) |
| Japan | 241.22(203.13,281.50) | 179.31(141.66,225.05) | -25.67(-44.56,0.21) | 1.04(0.88,1.22) | 1.16(0.92,1.46) | 0.17(-0.05,0.39) |
| Republic of Korea | 81.54(62.56,112.23) | 48.57(31.47,72.73) | -40.43(-63.04,-2.82) | 0.72(0.55,0.99) | 0.80(0.52,1.20) | 0.58(0.16,1.01) |
| Republic of Singapore | 4.98(4.02,6.20) | 8.28(6.04,11.15) | 66.28(17.73,145.06) | 0.77(0.62,0.96) | 1.02(0.74,1.37) | 1.70(1.13,2.28) |
| Australia | 59.24(48.67,73.04) | 45.41(33.27,60.76) | -23.36(-46.78,7.67) | 1.56(1.29,1.93) | 0.96(0.70,1.28) | -1.64(-2.32,-0.94) |
| New Zealand | 16.01(12.17,20.56) | 21.75(16.41,28.78) | 35.83(-4.16,91.47) | 2.00(1.52,2.57) | 2.22(1.67,2.93) | -0.47(-1.63,0.71) |
| Principality of Andorra | 0.66(0.38,1.02) | 0.33(0.21,0.51) | -49.99(-69.93,-3.76) | 6.94(4.00,10.73) | 3.24(2.09,5.02) | -2.22(-2.60,-1.84) |
| Republic of Austria | 26.35(20.72,33.84) | 22.76(17.31,29.63) | -13.61(-39.40,28.54) | 1.95(1.54,2.51) | 1.75(1.33,2.28) | -0.31(-0.69,0.08) |
| Kingdom of Belgium | 35.45(26.17,46.93) | 49.60(34.79,70.28) | 39.90(-8.34,120.15) | 1.96(1.45,2.60) | 2.59(1.82,3.68) | 0.10(-0.27,0.47) |
| Republic of Cyprus | 5.35(3.61,7.93) | 6.02(3.97,8.78) | 12.38(-33.85,100.65) | 2.70(1.83,4.01) | 2.75(1.82,4.01) | 1.44(0.29,2.60) |
| Kingdom of Denmark | 11.05(8.85,14.06) | 12.71(9.08,17.87) | 15.03(-24.36,73.88) | 1.25(1.00,1.59) | 1.33(0.95,1.87) | 0.04(-0.29,0.37) |
| Republic of Finland | 13.80(10.51,17.64) | 19.00(12.91,26.59) | 37.73(-14.13,116.98) | 1.43(1.09,1.83) | 2.24(1.52,3.14) | 0.90(0.26,1.54) |
| French Republic | 222.89(172.13,291.07) | 285.87(203.89,388.93) | 28.25(-12.80,90.75) | 1.90(1.47,2.48) | 2.46(1.76,3.35) | 0.57(0.29,0.84) |
| Federal Republic of Germany | 220.97(170.43,276.26) | 176.14(133.96,233.32) | -20.29(-44.61,17.73) | 1.71(1.32,2.13) | 1.47(1.12,1.95) | -0.50(-0.84,-0.16) |
| Hellenic Republic | 21.78(16.35,29.52) | 13.24(10.16,17.36) | -39.21(-59.18,-11.67) | 1.08(0.81,1.46) | 0.95(0.73,1.24) | -0.15(-0.67,0.36) |
| Republic of Iceland | 3.50(2.51,4.75) | 4.93(3.22,7.20) | 40.62(-16.37,140.40) | 5.52(3.96,7.48) | 7.29(4.77,10.66) | 1.31(0.38,2.25) |
| Ireland | 19.48(14.01,26.86) | 19.45(13.30,28.26) | -0.13(-37.84,65.15) | 1.98(1.43,2.73) | 1.95(1.33,2.83) | 0.43(-0.08,0.94) |
| State of Israel | 35.61(27.60,46.42) | 60.09(44.73,81.77) | 68.74(16.18,141.62) | 2.32(1.80,3.03) | 2.29(1.70,3.11) | 0.29(-0.32,0.91) |
| Republic of Italy | 328.58(236.57,441.71) | 233.12(173.69,313.21) | -29.05(-54.62,9.70) | 3.56(2.56,4.79) | 3.07(2.29,4.12) | -1.14(-1.63,-0.65) |
| Grand Duchy of Luxembourg | 1.14(0.85,1.53) | 1.87(1.39,2.53) | 63.76(6.28,149.25) | 1.73(1.29,2.32) | 1.85(1.38,2.50) | -1.31(-1.84,-0.78) |
| Republic of Malta | 3.65(2.31,5.28) | 9.14(5.75,13.44) | 150.25(43.28,323.38) | 4.17(2.64,6.03) | 14.27(8.98,20.99) | 2.02(1.17,2.88) |
| Kingdom of the Netherlands | 39.28(31.33,48.58) | 41.11(31.48,54.45) | 4.66(-26.57,46.82) | 1.44(1.15,1.78) | 1.53(1.17,2.03) | 0.42(0.01,0.84) |
| Kingdom of Norway | 15.76(12.20,20.19) | 13.78(10.24,18.71) | -12.53(-41.51,33.20) | 1.97(1.53,2.53) | 1.49(1.11,2.03) | -0.58(-1.40,0.24) |
| Portuguese Republic | 57.55(43.12,76.38) | 33.12(22.74,46.83) | -42.45(-64.30,-8.78) | 2.72(2.04,3.61) | 2.43(1.67,3.44) | -1.32(-1.74,-0.89) |
| Kingdom of Spain | 140.51(108.87,173.81) | 142.17(101.80,195.47) | 1.18(-32.72,49.11) | 1.79(1.39,2.22) | 2.19(1.57,3.02) | 0.33(-0.12,0.78) |
| Kingdom of Sweden | 20.79(16.49,26.05) | 20.65(15.87,26.76) | -0.69(-31.46,38.88) | 1.35(1.07,1.69) | 1.13(0.87,1.47) | 0.42(-0.46,1.31) |
| Swiss Confederation | 39.94(30.01,52.97) | 39.37(27.84,54.03) | -1.42(-34.93,56.19) | 3.46(2.60,4.58) | 2.95(2.09,4.05) | -1.10(-1.78,-0.42) |
| United Kingdom of Great Britain and Northern Ireland | 242.48(216.23,272.24) | 215.84(185.70,250.17) | -10.99(-26.11,6.82) | 2.22(1.98,2.49) | 1.83(1.58,2.12) | -0.41(-0.80,-0.01) |
| Argentine Republic | 93.66(75.38,115.10) | 85.57(66.01,111.74) | -8.63(-34.41,28.91) | 0.92(0.74,1.14) | 0.84(0.65,1.10) | 0.02(-0.36,0.40) |
| Republic of Chile | 24.82(20.97,29.44) | 21.37(16.92,26.90) | -13.91(-34.79,10.81) | 0.62(0.53,0.74) | 0.59(0.46,0.74) | 0.08(-0.32,0.49) |
| Eastern Republic of Uruguay | 8.84(7.22,11.08) | 7.13(5.35,9.37) | -19.34(-41.48,10.74) | 1.08(0.88,1.35) | 1.08(0.81,1.42) | -0.44(-1.08,0.21) |
| Canada | 87.55(67.82,113.80) | 55.94(39.85,76.37) | -36.10(-58.41,-3.37) | 1.52(1.18,1.98) | 0.91(0.65,1.24) | -1.98(-2.26,-1.70) |
| United States of America | 884.86(835.25,940.29) | 627.82(555.57,701.47) | -29.05(-37.35,-21.05) | 1.58(1.49,1.68) | 1.06(0.93,1.18) | -1.38(-1.54,-1.22) |
| Antigua and Barbuda | 0.16(0.12,0.19) | 0.21(0.16,0.27) | 36.19(-4.30,89.93) | 0.85(0.66,1.05) | 1.25(0.97,1.60) | 1.35(0.76,1.94) |
| Commonwealth of the Bahamas | 0.84(0.69,1.04) | 0.81(0.60,1.11) | -3.52(-34.87,39.22) | 1.05(0.86,1.29) | 1.00(0.73,1.37) | -0.38(-1.01,0.26) |
| Barbados | 1.26(1.04,1.56) | 0.82(0.57,1.15) | -35.55(-55.91,-6.59) | 2.03(1.66,2.51) | 1.73(1.20,2.44) | 0.45(0.00,0.90) |
| Belize | 0.80(0.66,0.95) | 0.61(0.48,0.77) | -23.30(-43.19,4.22) | 0.97(0.81,1.16) | 0.50(0.39,0.63) | -1.51(-1.82,-1.19) |
| Republic of Cuba | 47.67(39.20,58.22) | 30.75(22.20,41.83) | -35.48(-55.03,-7.80) | 1.90(1.57,2.33) | 1.73(1.25,2.35) | -0.11(-0.41,0.21) |
| Commonwealth of Dominica | 0.35(0.26,0.47) | 0.43(0.30,0.61) | 23.65(-22.42,81.77) | 1.40(1.04,1.88) | 3.13(2.18,4.43) | 2.78(2.56,3.00) |
| Dominican Republic | 31.29(22.67,40.82) | 24.61(15.26,37.43) | -21.35(-52.27,24.32) | 1.16(0.84,1.51) | 0.84(0.52,1.27) | -1.46(-1.85,-1.08) |
| Grenada | 0.83(0.65,1.08) | 0.62(0.47,0.84) | -25.24(-50.03,4.92) | 2.48(1.93,3.24) | 2.84(2.16,3.86) | 0.75(0.28,1.23) |
| Republic of Guyana | 1.98(1.52,2.46) | 1.60(1.14,2.14) | -19.42(-44.43,17.29) | 0.67(0.52,0.84) | 0.75(0.54,1.00) | 1.68(1.15,2.22) |
| Republic of Haiti | 92.30(25.56,146.31) | 95.68(39.14,157.11) | 3.66(-34.76,70.67) | 3.40(0.94,5.39) | 2.20(0.90,3.61) | -0.99(-1.21,-0.76) |
| Jamaica | 21.09(16.82,26.55) | 10.68(7.73,15.10) | -49.38(-66.11,-22.49) | 2.52(2.01,3.18) | 1.83(1.32,2.59) | -1.15(-1.35,-0.94) |
| Saint Lucia | 0.79(0.64,0.98) | 0.60(0.44,0.81) | -24.46(-47.64,12.70) | 1.53(1.25,1.91) | 2.01(1.47,2.74) | 0.32(-0.11,0.74) |
| Saint Vincent and the Grenadines | 0.97(0.79,1.20) | 0.60(0.45,0.77) | -38.34(-56.91,-13.38) | 2.37(1.92,2.93) | 2.41(1.81,3.10) | 0.18(-0.22,0.58) |
| Republic of Suriname | 1.57(0.94,2.08) | 1.69(1.14,2.47) | 7.74(-34.92,106.15) | 1.20(0.72,1.60) | 1.18(0.79,1.72) | 0.21(-0.10,0.52) |
| Republic of Trinidad and Tobago | 6.08(4.98,7.38) | 3.36(2.49,4.43) | -44.69(-61.59,-20.55) | 1.50(1.23,1.82) | 1.23(0.91,1.63) | 0.01(-0.40,0.41) |
| Plurinational State of Bolivia | 42.39(29.87,57.77) | 34.94(24.95,49.66) | -17.58(-48.02,32.43) | 1.58(1.11,2.15) | 1.00(0.72,1.42) | -1.51(-1.61,-1.42) |
| Republic of Ecuador | 27.78(23.46,32.86) | 42.93(31.63,56.72) | 54.52(10.44,107.10) | 0.72(0.61,0.85) | 0.85(0.62,1.12) | 0.61(-0.07,1.30) |
| Republic of Peru | 99.61(78.71,133.54) | 140.63(95.43,202.62) | 41.19(-8.31,113.87) | 1.20(0.95,1.61) | 1.47(1.00,2.12) | 1.13(0.90,1.35) |
| Republic of Colombia | 138.13(113.29,165.11) | 126.35(92.62,170.44) | -8.53(-35.41,30.89) | 1.18(0.97,1.42) | 1.19(0.87,1.61) | 0.97(0.53,1.40) |
| Republic of Costa Rica | 13.66(10.75,16.94) | 16.09(11.74,21.98) | 17.77(-20.37,71.46) | 1.22(0.96,1.51) | 1.58(1.15,2.16) | 0.94(0.77,1.11) |
| Republic of El Salvador | 12.68(9.80,16.17) | 12.17(8.79,16.44) | -4.04(-34.58,42.27) | 0.59(0.45,0.75) | 0.67(0.48,0.90) | 0.72(0.50,0.93) |
| Republic of Guatemala | 21.68(19.14,24.89) | 16.44(12.92,21.49) | -24.16(-42.06,0.20) | 0.53(0.47,0.61) | 0.33(0.26,0.44) | -0.59(-0.86,-0.32) |
| Republic of Honduras | 9.47(7.11,12.21) | 5.97(3.11,10.34) | -36.97(-67.61,10.09) | 0.43(0.32,0.55) | 0.18(0.09,0.32) | -2.97(-3.29,-2.65) |
| United Mexican States | 283.66(256.75,314.24) | 203.61(171.45,239.91) | -28.22(-41.46,-13.61) | 0.85(0.77,0.94) | 0.63(0.53,0.75) | -0.56(-1.04,-0.08) |
| Republic of Nicaragua | 15.05(11.08,19.12) | 8.44(6.10,11.71) | -43.90(-63.71,-12.72) | 0.83(0.61,1.05) | 0.43(0.31,0.59) | -1.02(-1.39,-0.66) |
| Republic of Panama | 8.10(6.58,9.98) | 18.22(13.91,23.55) | 124.85(62.90,212.40) | 0.97(0.79,1.20) | 1.58(1.21,2.04) | 1.36(1.11,1.62) |
| Bolivarian Republic of Venezuela | 63.61(54.07,75.49) | 77.99(57.87,104.13) | 22.60(-12.99,69.09) | 0.90(0.76,1.06) | 1.18(0.87,1.57) | 1.09(0.68,1.50) |
| Federative Republic of Brazil | 468.22(413.47,522.67) | 272.57(217.18,322.50) | -41.79(-53.20,-29.73) | 0.90(0.80,1.01) | 0.57(0.45,0.67) | -1.01(-1.54,-0.48) |
| Republic of Paraguay | 14.70(10.47,19.95) | 19.90(13.43,29.55) | 35.32(-14.79,112.07) | 0.88(0.63,1.19) | 0.99(0.67,1.47) | 0.63(0.18,1.08) |
| People's Democratic Republic of Algeria | 157.21(109.22,215.26) | 192.98(131.84,276.98) | 22.75(-23.52,90.36) | 1.47(1.02,2.01) | 1.45(0.99,2.08) | 0.14(-0.01,0.29) |
| Kingdom of Bahrain | 1.17(0.86,1.61) | 2.95(1.96,4.29) | 151.14(55.32,299.58) | 0.72(0.53,0.98) | 0.99(0.66,1.45) | 1.23(0.84,1.62) |
| Arab Republic of Egypt | 160.11(116.11,262.30) | 146.69(86.40,330.09) | -8.38(-44.27,53.67) | 0.72(0.52,1.18) | 0.40(0.23,0.90) | -1.91(-2.49,-1.32) |
| Islamic Republic of Iran | 214.75(150.86,292.88) | 260.70(176.88,368.55) | 21.40(-29.81,98.19) | 0.85(0.59,1.15) | 1.29(0.88,1.83) | 1.89(1.38,2.41) |
| Republic of Iraq | 132.20(79.67,197.54) | 179.03(117.62,255.38) | 35.42(-23.69,151.33) | 1.61(0.97,2.40) | 1.33(0.87,1.90) | 0.11(-0.19,0.42) |
| Hashemite Kingdom of Jordan | 24.39(17.05,33.82) | 92.96(63.05,133.87) | 281.17(123.14,533.25) | 1.49(1.04,2.07) | 2.56(1.74,3.68) | 1.88(1.49,2.27) |
| State of Kuwait | 17.80(13.61,22.87) | 19.88(13.57,27.38) | 11.70(-27.53,72.56) | 3.21(2.45,4.12) | 2.35(1.60,3.24) | 1.13(0.12,2.16) |
| Lebanese Republic | 16.07(10.83,22.39) | 26.85(16.57,41.69) | 67.09(-3.66,206.32) | 1.54(1.04,2.14) | 2.10(1.30,3.26) | 1.09(0.65,1.54) |
| State of Libya | 30.75(20.56,42.93) | 53.40(34.83,76.46) | 73.65(8.22,199.34) | 1.70(1.14,2.37) | 3.58(2.33,5.13) | 3.24(2.81,3.66) |
| Kingdom of Morocco | 90.40(57.19,131.32) | 70.00(44.09,103.69) | -22.57(-55.15,44.32) | 0.92(0.58,1.34) | 0.71(0.45,1.06) | -0.46(-0.63,-0.29) |
| Palestine | 7.01(4.67,10.17) | 16.97(11.60,23.88) | 142.30(41.78,308.91) | 0.72(0.48,1.05) | 0.91(0.62,1.28) | 1.44(0.94,1.94) |
| Sultanate of Oman | 8.39(5.06,12.49) | 16.62(11.29,23.92) | 98.12(7.30,239.43) | 1.00(0.60,1.49) | 1.36(0.92,1.96) | 2.01(1.53,2.50) |
| State of Qatar | 0.66(0.44,0.98) | 4.78(3.06,7.38) | 625.28(304.39,1190.44) | 0.53(0.35,0.79) | 0.97(0.62,1.49) | 3.01(2.51,3.51) |
| Kingdom of Saudi Arabia | 57.81(33.09,82.95) | 61.19(35.33,96.49) | 5.84(-44.43,100.66) | 0.88(0.50,1.27) | 0.81(0.47,1.28) | -0.07(-0.25,0.10) |
| Syrian Arab Republic | 47.43(32.26,62.42) | 37.56(24.55,56.61) | -20.81(-50.07,38.59) | 0.80(0.54,1.05) | 1.03(0.67,1.55) | 0.98(0.45,1.52) |
| Republic of Tunisia | 69.44(46.52,96.78) | 55.26(35.45,82.55) | -20.42(-55.65,37.83) | 2.24(1.50,3.12) | 2.00(1.28,2.98) | -0.04(-0.20,0.11) |
| Republic of Turkey | 267.91(156.91,374.04) | 261.01(185.20,366.13) | -2.57(-39.98,72.14) | 1.31(0.77,1.83) | 1.41(1.00,1.98) | 0.64(0.25,1.04) |
| United Arab Emirates | 3.34(2.02,5.39) | 6.08(4.02,8.93) | 82.04(-0.29,259.87) | 0.57(0.34,0.91) | 0.45(0.30,0.67) | -0.36(-0.78,0.06) |
| Republic of Yemen | 48.12(25.84,91.06) | 72.68(44.49,114.50) | 51.03(-8.51,206.60) | 0.68(0.36,1.28) | 0.53(0.32,0.83) | -0.57(-0.73,-0.41) |
| Islamic Republic of Afghanistan | 36.96(13.05,63.56) | 82.39(50.72,131.86) | 122.91(46.33,398.94) | 0.86(0.30,1.48) | 0.58(0.36,0.93) | -0.90(-1.15,-0.64) |
| People's Republic of Bangladesh | 771.00(330.27,1140.61) | 561.48(355.08,958.16) | -27.17(-56.04,46.02) | 1.58(0.68,2.33) | 1.23(0.78,2.09) | -0.55(-0.76,-0.34) |
| Kingdom of Bhutan | 3.20(1.36,5.20) | 2.04(1.15,3.78) | -36.35(-72.15,152.67) | 1.22(0.52,1.98) | 1.09(0.61,2.02) | -0.76(-1.05,-0.47) |
| Republic of India | 2788.41(1697.94,3755.23) | 1895.15(1460.53,2495.08) | -32.03(-50.05,35.14) | 0.85(0.52,1.15) | 0.52(0.40,0.68) | -1.59(-1.79,-1.39) |
| Federal Democratic Republic of Nepal | 98.87(44.42,154.17) | 65.13(40.88,107.43) | -34.13(-59.71,48.85) | 1.17(0.53,1.83) | 0.71(0.44,1.16) | -1.35(-1.57,-1.13) |
| Islamic Republic of Pakistan | 784.56(559.80,1049.17) | 1990.97(1340.98,2880.86) | 153.77(63.40,312.79) | 1.59(1.14,2.13) | 2.33(1.57,3.37) | 1.70(1.51,1.88) |
| Republic of Angola | 61.13(12.78,106.29) | 74.34(44.69,113.28) | 21.60(-25.64,339.33) | 1.30(0.27,2.25) | 0.49(0.29,0.74) | -2.95(-3.23,-2.67) |
| Central African Republic | 11.61(3.30,19.86) | 14.95(7.16,23.55) | 28.76(-16.30,141.40) | 0.95(0.27,1.62) | 0.65(0.31,1.03) | -0.93(-1.09,-0.78) |
| Republic of the Congo | 7.68(3.15,11.86) | 8.66(5.80,12.44) | 12.85(-27.10,125.30) | 0.73(0.30,1.13) | 0.45(0.30,0.64) | -1.49(-1.68,-1.30) |
| Democratic Republic of the Congo | 143.46(46.44,215.19) | 138.01(83.42,197.12) | -3.80(-36.44,139.18) | 0.81(0.26,1.22) | 0.36(0.22,0.52) | -2.13(-2.35,-1.90) |
| Republic of Equatorial Guinea | 1.65(0.55,2.66) | 3.56(1.67,6.85) | 115.62(-1.21,460.50) | 0.84(0.28,1.35) | 0.61(0.29,1.17) | -1.65(-2.07,-1.22) |
| Gabonese Republic | 2.40(1.36,3.57) | 4.02(2.48,6.33) | 67.90(-8.14,191.64) | 0.59(0.33,0.88) | 0.63(0.39,0.99) | 0.73(0.48,0.99) |
| Republic of Burundi | 77.43(48.24,119.73) | 67.80(35.07,124.76) | -12.43(-47.85,91.05) | 2.95(1.84,4.57) | 1.16(0.60,2.13) | -2.45(-2.70,-2.20) |
| Union of the Comoros | 4.01(2.09,5.69) | 3.72(2.31,5.71) | -7.32(-46.84,82.24) | 1.89(0.98,2.68) | 1.55(0.96,2.38) | -0.69(-1.08,-0.29) |
| Republic of Djibouti | 3.13(2.09,4.51) | 5.14(2.94,8.88) | 64.09(-1.93,166.68) | 1.80(1.20,2.59) | 1.24(0.71,2.15) | -1.11(-1.47,-0.74) |
| State of Eritrea | 32.74(21.29,45.86) | 35.33(20.93,63.18) | 7.93(-35.54,116.11) | 2.06(1.34,2.88) | 1.40(0.83,2.50) | -1.18(-1.35,-1.01) |
| Federal Democratic Republic of Ethiopia | 704.39(185.39,1084.71) | 656.80(434.34,950.60) | -6.76(-37.72,198.78) | 2.89(0.76,4.45) | 1.48(0.98,2.14) | -2.53(-2.87,-2.19) |
| Republic of Kenya | 82.79(53.52,118.46) | 104.02(72.91,137.60) | 25.65(-12.58,96.13) | 0.74(0.48,1.06) | 0.56(0.39,0.74) | 0.08(-0.44,0.61) |
| Republic of Madagascar | 107.69(76.19,144.87) | 125.16(79.42,187.59) | 16.23(-24.09,101.97) | 1.97(1.40,2.66) | 1.07(0.68,1.60) | -1.56(-1.77,-1.36) |
| Republic of Malawi | 349.37(249.76,466.12) | 379.63(180.31,782.00) | 8.66(-46.58,112.75) | 7.68(5.49,10.25) | 4.67(2.22,9.63) | -1.26(-1.52,-1.00) |
| Republic of Mauritius | 0.45(0.39,0.52) | 0.88(0.68,1.13) | 95.93(45.93,159.97) | 0.14(0.12,0.16) | 0.43(0.33,0.54) | 2.22(1.48,2.96) |
| Republic of Mozambique | 25.01(13.42,63.71) | 25.96(12.48,59.62) | 3.80(-43.75,165.16) | 0.40(0.22,1.03) | 0.18(0.09,0.42) | -2.14(-2.44,-1.84) |
| Republic of Rwanda | 111.06(74.57,156.08) | 73.81(47.61,126.92) | -33.54(-59.99,32.69) | 3.27(2.20,4.60) | 1.48(0.96,2.55) | -3.07(-3.36,-2.78) |
| Republic of Seychelles | 0.08(0.06,0.11) | 0.08(0.05,0.12) | 5.83(-34.52,69.63) | 0.33(0.24,0.47) | 0.35(0.23,0.52) | 1.91(0.83,3.00) |
| Federal Republic of Somalia | 73.19(38.98,109.21) | 102.81(50.31,160.81) | 40.47(-12.20,138.68) | 1.88(1.00,2.80) | 1.00(0.49,1.56) | -1.59(-1.98,-1.21) |
| United Republic of Tanzania | 331.92(225.91,448.40) | 480.01(300.14,788.12) | 44.62(-13.09,140.90) | 2.75(1.87,3.71) | 1.97(1.23,3.23) | -0.56(-0.85,-0.28) |
| Republic of Uganda | 249.14(164.03,349.15) | 608.41(344.59,1027.66) | 144.20(50.57,305.74) | 2.96(1.95,4.15) | 3.07(1.74,5.18) | 0.30(0.07,0.53) |
| Republic of Zambia | 108.66(73.45,146.60) | 139.38(80.67,236.09) | 28.28(-22.87,166.04) | 2.89(1.96,3.90) | 1.69(0.98,2.85) | -1.58(-1.75,-1.42) |
| Republic of Botswana | 2.50(1.68,3.79) | 5.56(3.44,8.46) | 122.85(37.58,256.73) | 0.42(0.28,0.64) | 0.80(0.49,1.21) | 2.83(2.37,3.30) |
| Kingdom of Lesotho | 2.51(1.67,4.04) | 4.44(2.71,6.72) | 77.05(14.29,186.37) | 0.37(0.24,0.59) | 0.70(0.43,1.07) | 3.02(2.64,3.40) |
| Republic of Namibia | 4.60(2.88,6.82) | 11.17(6.73,17.28) | 142.58(40.02,323.34) | 0.77(0.48,1.13) | 1.35(0.82,2.09) | 2.86(2.17,3.55) |
| Republic of South Africa | 78.61(52.87,119.32) | 121.51(98.29,154.62) | 54.58(13.82,116.63) | 0.58(0.39,0.88) | 0.80(0.65,1.02) | 0.83(0.34,1.32) |
| Kingdom of Eswatini | 2.66(1.55,4.11) | 3.64(2.23,5.55) | 36.88(-8.71,122.13) | 0.69(0.40,1.07) | 0.88(0.54,1.35) | 1.05(0.69,1.42) |
| Republic of Zimbabwe | 33.63(15.91,49.04) | 103.23(47.74,150.59) | 206.97(87.55,379.00) | 0.70(0.33,1.02) | 1.64(0.76,2.39) | 4.95(3.83,6.08) |
| Republic of Benin | 38.55(25.12,56.51) | 78.26(48.54,114.59) | 103.00(18.66,257.70) | 1.59(1.04,2.33) | 1.29(0.80,1.88) | -0.49(-0.63,-0.35) |
| Burkina Faso | 73.82(45.28,107.87) | 134.62(90.96,185.50) | 82.36(19.35,186.73) | 1.56(0.96,2.29) | 1.30(0.88,1.79) | -0.25(-0.45,-0.05) |
| Republic of Cameroon | 74.90(49.07,103.81) | 195.74(128.80,283.49) | 161.34(72.35,312.25) | 1.53(1.01,2.13) | 1.45(0.96,2.11) | 0.15(-0.03,0.33) |
| Republic of Cabo Verde | 0.43(0.24,0.74) | 2.74(1.09,4.03) | 536.47(73.85,1204.29) | 0.27(0.15,0.47) | 1.91(0.76,2.81) | 5.43(4.55,6.32) |
| Republic of Chad | 36.58(22.05,55.54) | 109.33(65.38,159.15) | 198.84(89.68,403.62) | 1.25(0.75,1.90) | 1.21(0.73,1.77) | 0.25(0.13,0.37) |
| Republic of Côte d'Ivoire | 93.05(64.80,130.08) | 196.86(123.69,351.71) | 111.57(28.86,246.10) | 1.63(1.14,2.28) | 1.70(1.07,3.04) | 0.41(0.17,0.65) |
| Republic of the Gambia | 8.60(5.64,12.06) | 12.95(7.68,23.16) | 50.58(-13.83,162.13) | 1.86(1.22,2.61) | 1.30(0.77,2.33) | -1.35(-1.64,-1.07) |
| Republic of Ghana | 153.90(74.86,231.85) | 78.53(48.70,135.01) | -48.98(-73.16,37.80) | 2.29(1.11,3.45) | 0.61(0.38,1.05) | -5.84(-6.91,-4.76) |
| Republic of Guinea | 58.01(38.43,82.15) | 64.37(37.92,131.99) | 10.96(-33.24,116.94) | 2.11(1.40,2.99) | 1.06(0.63,2.18) | -1.69(-1.85,-1.53) |
| Republic of Guinea-Bissau | 9.28(5.66,13.62) | 8.39(5.16,13.16) | -9.58(-42.39,72.08) | 1.92(1.17,2.82) | 0.93(0.57,1.47) | -2.09(-2.42,-1.75) |
| Republic of Liberia | 24.77(13.63,34.36) | 26.50(16.55,38.76) | 6.98(-37.11,92.38) | 2.19(1.21,3.04) | 1.21(0.76,1.77) | -1.72(-2.47,-0.97) |
| Republic of Mali | 56.29(35.72,82.05) | 76.77(44.87,152.13) | 36.38(-16.94,169.19) | 1.36(0.86,1.99) | 0.66(0.39,1.31) | -2.03(-2.20,-1.85) |
| Islamic Republic of Mauritania | 8.76(5.80,12.78) | 17.54(10.90,25.58) | 100.07(15.83,228.60) | 0.95(0.63,1.38) | 0.95(0.59,1.38) | -0.29(-0.50,-0.07) |
| Republic of the Niger | 89.23(50.21,134.30) | 108.05(60.63,166.79) | 21.09(-30.30,133.59) | 2.20(1.24,3.31) | 0.85(0.48,1.31) | -3.09(-3.34,-2.84) |
| Federal Republic of Nigeria | 854.62(549.17,1208.59) | 1977.44(977.58,3011.15) | 131.38(50.48,332.39) | 2.18(1.40,3.09) | 1.95(0.96,2.96) | -0.07(-0.22,0.08) |
| Democratic Republic of Sao Tome and Principe | 0.95(0.66,1.39) | 0.61(0.33,1.14) | -35.41(-65.07,29.91) | 1.67(1.16,2.46) | 0.79(0.43,1.46) | -2.09(-2.61,-1.57) |
| Republic of Senegal | 57.84(39.87,82.58) | 52.56(34.41,83.90) | -9.13(-42.39,46.91) | 1.58(1.09,2.26) | 0.83(0.54,1.32) | -2.15(-2.39,-1.90) |
| Republic of Sierra Leone | 37.60(21.75,54.11) | 42.61(28.01,62.09) | 13.31(-31.07,118.37) | 2.07(1.20,2.98) | 1.19(0.78,1.74) | -1.75(-1.92,-1.58) |
| Togolese Republic | 21.66(14.50,31.09) | 29.37(17.93,44.54) | 35.56(-15.71,129.91) | 1.23(0.82,1.76) | 0.89(0.54,1.35) | -0.98(-1.11,-0.86) |
| American Samoa | 0.15(0.10,0.24) | 0.27(0.15,0.45) | 78.50(-8.87,244.96) | 0.79(0.51,1.26) | 1.90(1.08,3.17) | 3.24(2.97,3.51) |
| Bermuda | 0.21(0.16,0.28) | 0.25(0.18,0.35) | 15.10(-25.06,77.18) | 1.79(1.36,2.37) | 2.91(2.09,4.11) | 2.01(1.49,2.53) |
| Cook Islands | 0.06(0.03,0.09) | 0.09(0.04,0.18) | 51.82(-40.52,291.56) | 0.88(0.50,1.40) | 2.34(1.06,4.78) | 1.48(0.80,2.17) |
| Greenland | 0.18(0.08,0.25) | 0.07(0.04,0.10) | -60.82(-78.35,-24.21) | 1.24(0.58,1.76) | 0.59(0.38,0.87) | -2.13(-2.63,-1.64) |
| Guam | 1.11(0.78,1.54) | 0.84(0.50,1.37) | -24.04(-58.32,37.16) | 2.67(1.86,3.69) | 2.31(1.37,3.73) | 2.38(1.31,3.47) |
| Principality of Monaco | 0.10(0.06,0.16) | 0.20(0.13,0.32) | 102.86(19.93,264.95) | 2.81(1.69,4.43) | 4.04(2.57,6.39) | -0.22(-0.75,0.32) |
| Republic of Nauru | 0.05(0.03,0.07) | 0.08(0.05,0.13) | 79.50(-5.51,254.83) | 1.07(0.66,1.62) | 2.03(1.17,3.24) | 1.80(1.59,2.02) |
| Republic of Niue | 0.01(0.01,0.01) | 0.06(0.04,0.09) | 521.27(251.01,983.06) | 1.14(0.70,1.77) | 14.78(9.19,24.50) | 4.53(3.16,5.91) |
| Northern Mariana Islands | 0.07(0.04,0.14) | 0.16(0.09,0.28) | 121.84(3.48,392.61) | 0.60(0.33,1.14) | 1.43(0.80,2.51) | 3.56(2.72,4.40) |
| Republic of Palau | 0.03(0.02,0.04) | 0.02(0.01,0.03) | -20.67(-55.29,44.21) | 0.58(0.34,0.91) | 0.64(0.36,1.05) | 0.27(0.01,0.53) |
| Puerto Rico | 15.00(12.08,18.51) | 4.17(3.19,5.51) | -72.20(-80.56,-61.30) | 1.51(1.21,1.86) | 0.94(0.72,1.24) | -0.77(-1.13,-0.42) |
| Saint Kitts and Nevis | 0.17(0.14,0.20) | 0.16(0.12,0.21) | -8.39(-35.39,26.24) | 1.21(1.02,1.44) | 1.59(1.22,2.09) | 1.17(0.84,1.50) |
| Republic of San Marino | 0.37(0.24,0.54) | 0.20(0.13,0.31) | -46.04(-68.08,1.42) | 9.14(5.93,13.17) | 4.60(2.94,7.07) | -1.97(-2.17,-1.77) |
| Tokelau | 0.01(0.00,0.01) | 0.05(0.02,0.08) | 762.01(320.61,1681.25) | 0.88(0.52,1.39) | 11.71(6.37,20.53) | 4.06(2.24,5.90) |
| Tuvalu | 0.03(0.02,0.05) | 0.04(0.02,0.06) | 27.79(-34.98,158.48) | 0.88(0.47,1.37) | 1.05(0.66,1.54) | 0.72(0.60,0.83) |
| United States Virgin Islands | 0.44(0.31,0.62) | 0.08(0.04,0.13) | -82.41(-90.83,-66.53) | 1.37(0.98,1.93) | 0.58(0.33,0.98) | -1.76(-2.33,-1.19) |
| Republic of South Sudan | 66.29(37.63,94.24) | 105.67(63.15,154.01) | 59.40(6.40,142.11) | 2.53(1.43,3.59) | 2.46(1.47,3.59) | 0.41(-0.30,1.13) |
| Republic of Sudan | 135.90(55.23,275.55) | 186.65(120.81,274.39) | 37.34(-32.87,210.70) | 1.53(0.62,3.10) | 1.13(0.73,1.65) | -0.76(-0.87,-0.65) |

**Table S4: Mortality of Non-Hodgkin Lymphoma in children between 1990 and 2021 in 204 countries.**

| location | Number (95% UI) | | Percentage change (95% UI) | Rate(95% UI) | | EAPC (95% CI) |
| --- | --- | --- | --- | --- | --- | --- |
|  | 1990 | 2021 |  | 1990 | 2021 |  |
| People's Republic of China | 2407.27(2002.99,2938.59) | 488.34(392.42,627.62) | -79.71(-84.37,-72.04) | 0.76(0.63,0.92) | 0.19(0.15,0.24) | -4.53(-4.89,-4.18) |
| Democratic People's Republic of Korea | 25.12(15.97,36.56) | 9.94(6.54,15.67) | -60.43(-74.92,-37.04) | 0.42(0.27,0.61) | 0.21(0.14,0.33) | -1.99(-2.25,-1.73) |
| Taiwan (Province of China) | 22.24(19.84,24.65) | 5.40(4.60,6.39) | -75.74(-79.62,-70.61) | 0.40(0.36,0.45) | 0.18(0.16,0.22) | -2.53(-2.90,-2.16) |
| Kingdom of Cambodia | 28.76(12.35,42.47) | 15.67(10.97,25.08) | -45.50(-65.65,20.01) | 0.62(0.26,0.91) | 0.31(0.21,0.49) | -2.61(-2.85,-2.37) |
| Republic of Indonesia | 310.76(170.79,467.93) | 167.67(119.25,259.67) | -46.05(-60.83,-13.93) | 0.46(0.25,0.69) | 0.25(0.18,0.39) | -1.84(-1.89,-1.79) |
| Lao People's Democratic Republic | 13.71(4.75,21.55) | 7.79(5.04,12.40) | -43.14(-64.71,35.58) | 0.74(0.26,1.17) | 0.34(0.22,0.54) | -2.60(-2.67,-2.53) |
| Malaysia | 22.73(16.05,30.12) | 14.02(9.82,18.51) | -38.32(-62.80,-3.64) | 0.35(0.24,0.46) | 0.18(0.13,0.24) | -1.61(-1.94,-1.27) |
| Republic of Maldives | 0.46(0.22,0.67) | 0.15(0.11,0.23) | -66.44(-80.61,-8.95) | 0.44(0.21,0.64) | 0.15(0.11,0.23) | -2.97(-3.17,-2.78) |
| Republic of the Union of Myanmar | 126.37(44.06,197.53) | 59.54(38.63,92.68) | -52.89(-68.04,1.31) | 0.86(0.30,1.34) | 0.38(0.25,0.59) | -2.85(-3.00,-2.70) |
| Republic of the Philippines | 143.14(96.32,189.70) | 102.55(83.29,137.04) | -28.35(-43.72,4.36) | 0.57(0.38,0.75) | 0.30(0.24,0.40) | -1.50(-1.68,-1.32) |
| Democratic Socialist Republic of Sri Lanka | 16.09(11.77,20.90) | 6.21(4.09,8.83) | -61.39(-74.30,-37.00) | 0.29(0.21,0.38) | 0.12(0.08,0.17) | -2.79(-3.36,-2.21) |
| Kingdom of Thailand | 88.25(67.85,117.32) | 21.85(16.18,34.35) | -75.24(-82.80,-64.88) | 0.52(0.40,0.70) | 0.22(0.17,0.35) | -3.39(-3.79,-2.98) |
| Democratic Republic of Timor-Leste | 2.03(0.76,3.20) | 1.32(0.93,2.03) | -34.88(-57.95,50.39) | 0.61(0.23,0.96) | 0.25(0.18,0.39) | -2.98(-3.40,-2.56) |
| Socialist Republic of Viet Nam | 125.53(83.90,171.37) | 75.02(49.52,106.91) | -40.24(-62.30,6.18) | 0.47(0.32,0.65) | 0.30(0.20,0.43) | -1.14(-1.29,-0.99) |
| Republic of Fiji | 0.42(0.28,0.64) | 0.72(0.47,1.09) | 73.21(-1.94,188.25) | 0.15(0.10,0.23) | 0.27(0.17,0.40) | 2.19(1.94,2.44) |
| Republic of Kiribati | 0.03(0.02,0.05) | 0.04(0.02,0.06) | 6.94(-40.09,92.74) | 0.11(0.06,0.16) | 0.09(0.04,0.14) | -1.01(-1.21,-0.82) |
| Republic of the Marshall Islands | 0.03(0.02,0.05) | 0.04(0.03,0.07) | 38.82(-24.47,157.88) | 0.14(0.09,0.21) | 0.24(0.15,0.38) | 1.70(1.26,2.15) |
| Federated States of Micronesia | 0.11(0.07,0.16) | 0.06(0.04,0.10) | -39.21(-64.14,15.85) | 0.23(0.15,0.35) | 0.21(0.14,0.33) | -0.18(-0.35,-0.01) |
| Independent State of Papua New Guinea | 4.77(2.65,7.43) | 12.44(7.61,19.38) | 160.97(71.92,293.78) | 0.28(0.16,0.44) | 0.32(0.19,0.49) | 0.59(0.34,0.84) |
| Independent State of Samoa | 0.32(0.22,0.45) | 0.24(0.15,0.41) | -24.93(-53.16,27.23) | 0.45(0.30,0.64) | 0.30(0.19,0.51) | -1.28(-1.39,-1.17) |
| Solomon Islands | 0.24(0.13,0.38) | 0.51(0.34,0.78) | 112.22(26.27,306.96) | 0.16(0.08,0.25) | 0.20(0.13,0.30) | 0.91(0.62,1.21) |
| Kingdom of Tonga | 0.23(0.15,0.33) | 0.23(0.14,0.38) | -1.50(-42.28,81.18) | 0.56(0.37,0.79) | 0.59(0.37,0.97) | -0.20(-0.63,0.24) |
| Republic of Vanuatu | 0.09(0.05,0.14) | 0.19(0.12,0.28) | 122.75(38.51,279.18) | 0.13(0.07,0.20) | 0.16(0.11,0.24) | 1.15(0.72,1.58) |
| Republic of Armenia | 3.11(2.23,3.80) | 1.36(1.08,1.73) | -56.47(-69.36,-30.23) | 0.30(0.21,0.36) | 0.23(0.18,0.29) | 0.69(0.09,1.30) |
| Republic of Azerbaijan | 11.40(7.78,15.89) | 7.90(5.09,12.26) | -30.74(-58.44,19.07) | 0.47(0.32,0.65) | 0.33(0.22,0.52) | -1.63(-1.98,-1.27) |
| Georgia | 9.58(6.85,12.57) | 1.33(0.94,1.77) | -86.16(-91.09,-76.40) | 0.70(0.50,0.92) | 0.18(0.13,0.24) | -4.11(-4.82,-3.39) |
| Republic of Kazakhstan | 35.32(30.88,39.95) | 9.62(7.79,11.80) | -72.75(-79.16,-64.75) | 0.68(0.59,0.77) | 0.18(0.14,0.22) | -4.40(-4.84,-3.96) |
| Kyrgyz Republic | 6.06(4.79,7.95) | 3.97(2.98,5.03) | -34.46(-56.12,-7.18) | 0.36(0.29,0.47) | 0.17(0.13,0.22) | -2.30(-2.82,-1.78) |
| Mongolia | 8.86(5.73,12.37) | 2.77(1.94,3.92) | -68.77(-81.03,-45.81) | 0.98(0.64,1.37) | 0.25(0.18,0.36) | -4.57(-5.09,-4.05) |
| Republic of Tajikistan | 29.20(13.23,42.83) | 25.36(17.09,36.89) | -13.17(-46.72,78.79) | 1.26(0.57,1.84) | 0.71(0.48,1.03) | -2.45(-2.84,-2.05) |
| Turkmenistan | 3.86(3.33,4.47) | 2.64(2.17,3.22) | -31.69(-46.95,-13.02) | 0.26(0.22,0.30) | 0.17(0.14,0.21) | -1.81(-2.30,-1.31) |
| Republic of Uzbekistan | 52.00(36.35,67.64) | 42.68(33.53,54.55) | -17.93(-44.08,28.05) | 0.61(0.42,0.79) | 0.42(0.33,0.54) | -0.96(-1.25,-0.68) |
| Republic of Albania | 4.23(3.08,5.52) | 0.80(0.53,1.13) | -81.03(-87.54,-71.58) | 0.38(0.28,0.49) | 0.18(0.12,0.26) | -2.05(-2.81,-1.29) |
| Bosnia and Herzegovina | 2.83(2.19,3.56) | 0.81(0.55,1.10) | -71.20(-81.13,-59.50) | 0.26(0.20,0.32) | 0.17(0.11,0.22) | -1.13(-1.44,-0.82) |
| Republic of Bulgaria | 7.04(6.23,7.73) | 2.15(1.86,2.49) | -69.49(-74.49,-63.09) | 0.41(0.36,0.45) | 0.22(0.19,0.26) | -1.96(-2.43,-1.49) |
| Republic of Croatia | 3.49(2.88,4.17) | 1.15(0.88,1.48) | -66.91(-76.35,-53.20) | 0.35(0.29,0.42) | 0.19(0.15,0.25) | -1.41(-1.98,-0.83) |
| Czech Republic | 7.44(6.35,8.71) | 3.03(2.32,3.81) | -59.21(-70.04,-45.42) | 0.34(0.29,0.40) | 0.18(0.13,0.22) | -1.67(-2.01,-1.34) |
| Hungary | 7.86(6.62,9.30) | 2.18(1.80,2.68) | -72.22(-78.98,-62.91) | 0.37(0.31,0.44) | 0.16(0.13,0.19) | -1.89(-2.31,-1.48) |
| North Macedonia | 2.25(1.83,2.82) | 0.59(0.42,0.78) | -73.74(-82.66,-59.90) | 0.43(0.35,0.53) | 0.18(0.13,0.24) | -2.02(-2.58,-1.47) |
| Montenegro | 0.61(0.48,0.75) | 0.14(0.10,0.21) | -77.24(-84.35,-61.47) | 0.38(0.30,0.46) | 0.12(0.09,0.19) | -3.29(-3.91,-2.66) |
| Republic of Poland | 37.46(35.87,39.01) | 9.13(8.09,10.00) | -75.64(-78.41,-73.02) | 0.39(0.37,0.41) | 0.16(0.14,0.17) | -2.99(-3.40,-2.57) |
| Romania | 33.95(29.95,38.16) | 6.00(5.13,7.07) | -82.32(-85.29,-78.26) | 0.61(0.54,0.69) | 0.20(0.17,0.23) | -3.73(-4.09,-3.38) |
| Republic of Serbia | 11.52(7.70,14.86) | 1.59(1.16,2.28) | -86.16(-90.89,-71.66) | 0.53(0.36,0.69) | 0.12(0.09,0.17) | -5.13(-5.64,-4.62) |
| Slovak Republic | 4.62(3.66,5.96) | 1.65(1.24,2.25) | -64.39(-74.21,-48.36) | 0.35(0.28,0.45) | 0.19(0.14,0.26) | -1.87(-2.12,-1.61) |
| Republic of Slovenia | 1.31(1.13,1.48) | 0.46(0.37,0.58) | -64.52(-73.04,-55.43) | 0.32(0.27,0.36) | 0.15(0.12,0.18) | -1.67(-2.12,-1.23) |
| Republic of Belarus | 4.03(3.38,4.89) | 3.61(2.92,4.56) | -10.33(-31.91,18.58) | 0.17(0.14,0.20) | 0.23(0.18,0.29) | 2.19(1.52,2.87) |
| Republic of Estonia | 1.03(0.86,1.24) | 0.55(0.44,0.67) | -46.72(-59.82,-28.96) | 0.29(0.25,0.35) | 0.25(0.20,0.31) | -0.93(-1.71,-0.14) |
| Republic of Latvia | 1.19(1.02,1.37) | 0.49(0.40,0.60) | -58.86(-68.15,-47.10) | 0.21(0.18,0.24) | 0.16(0.13,0.20) | 0.96(0.23,1.70) |
| Republic of Lithuania | 1.49(1.23,1.81) | 0.95(0.75,1.21) | -36.15(-53.04,-14.80) | 0.18(0.15,0.22) | 0.23(0.18,0.30) | 0.49(-0.12,1.10) |
| Republic of Moldova | 11.67(10.00,13.47) | 1.70(1.41,2.06) | -85.44(-88.44,-81.47) | 0.94(0.81,1.09) | 0.33(0.27,0.39) | -2.38(-2.88,-1.88) |
| Russian Federation | 242.75(234.85,250.45) | 43.08(39.97,46.00) | -82.25(-83.65,-81.04) | 0.70(0.68,0.72) | 0.17(0.15,0.18) | -4.91(-5.37,-4.44) |
| Ukraine | 41.78(29.13,51.48) | 16.75(13.91,20.22) | -59.90(-70.47,-35.99) | 0.37(0.26,0.45) | 0.26(0.22,0.32) | -1.31(-1.73,-0.89) |
| Brunei Darussalam | 0.54(0.39,0.72) | 0.26(0.19,0.33) | -52.13(-66.79,-26.37) | 0.60(0.44,0.80) | 0.27(0.20,0.35) | -1.38(-1.87,-0.88) |
| Japan | 68.97(67.07,70.69) | 21.72(20.47,22.75) | -68.51(-70.42,-66.76) | 0.30(0.29,0.31) | 0.14(0.13,0.15) | -2.61(-2.85,-2.37) |
| Republic of Korea | 48.18(38.10,63.41) | 6.40(4.68,8.68) | -86.72(-91.16,-79.23) | 0.42(0.34,0.56) | 0.11(0.08,0.14) | -4.67(-4.93,-4.41) |
| Republic of Singapore | 2.27(1.93,2.73) | 1.13(0.93,1.36) | -50.26(-60.70,-36.49) | 0.35(0.30,0.42) | 0.14(0.12,0.17) | -2.47(-2.81,-2.13) |
| Australia | 9.88(8.74,11.24) | 3.70(2.99,4.42) | -62.57(-70.93,-53.04) | 0.26(0.23,0.30) | 0.08(0.06,0.09) | -3.70(-4.19,-3.20) |
| New Zealand | 1.38(1.21,1.57) | 0.85(0.72,1.01) | -38.67(-50.14,-23.63) | 0.17(0.15,0.20) | 0.09(0.07,0.10) | -2.84(-3.84,-1.82) |
| Principality of Andorra | 0.06(0.04,0.09) | 0.02(0.01,0.02) | -72.58(-82.58,-55.38) | 0.64(0.41,0.90) | 0.16(0.12,0.23) | -4.05(-4.29,-3.80) |
| Republic of Austria | 2.81(2.58,3.05) | 1.24(1.09,1.40) | -55.92(-61.52,-49.49) | 0.21(0.19,0.23) | 0.10(0.08,0.11) | -2.12(-2.39,-1.84) |
| Kingdom of Belgium | 4.03(3.51,4.64) | 2.74(2.24,3.40) | -31.93(-46.87,-13.02) | 0.22(0.19,0.26) | 0.14(0.12,0.18) | -2.17(-2.43,-1.91) |
| Republic of Cyprus | 0.51(0.38,0.65) | 0.19(0.14,0.26) | -62.27(-74.10,-42.83) | 0.26(0.19,0.33) | 0.09(0.06,0.12) | -2.91(-3.83,-1.98) |
| Kingdom of Denmark | 1.97(1.71,2.27) | 0.84(0.69,1.02) | -57.08(-66.91,-45.56) | 0.22(0.19,0.26) | 0.09(0.07,0.11) | -3.38(-3.65,-3.12) |
| Republic of Finland | 2.31(1.94,2.76) | 1.22(0.97,1.53) | -47.18(-60.12,-29.26) | 0.24(0.20,0.29) | 0.14(0.11,0.18) | -2.41(-2.82,-1.99) |
| French Republic | 28.91(25.86,31.69) | 14.63(12.35,17.10) | -49.40(-58.21,-39.45) | 0.25(0.22,0.27) | 0.13(0.11,0.15) | -2.59(-2.81,-2.37) |
| Federal Republic of Germany | 28.24(25.44,31.81) | 12.63(10.85,14.56) | -55.28(-62.48,-46.48) | 0.22(0.20,0.25) | 0.11(0.09,0.12) | -2.15(-2.39,-1.91) |
| Hellenic Republic | 2.13(1.94,2.34) | 0.86(0.74,0.98) | -59.77(-65.56,-52.63) | 0.11(0.10,0.12) | 0.06(0.05,0.07) | -1.42(-1.76,-1.08) |
| Republic of Iceland | 0.14(0.12,0.16) | 0.07(0.06,0.08) | -51.02(-61.88,-34.95) | 0.22(0.19,0.25) | 0.10(0.08,0.12) | -2.07(-2.75,-1.38) |
| Ireland | 2.40(2.01,2.81) | 0.90(0.71,1.15) | -62.38(-71.84,-49.60) | 0.24(0.20,0.29) | 0.09(0.07,0.12) | -3.18(-3.57,-2.79) |
| State of Israel | 5.60(4.97,6.35) | 4.14(3.43,5.06) | -26.16(-41.18,-7.69) | 0.37(0.32,0.41) | 0.16(0.13,0.19) | -2.56(-2.97,-2.15) |
| Republic of Italy | 34.03(32.22,35.97) | 10.60(9.45,11.97) | -68.84(-72.38,-65.02) | 0.37(0.35,0.39) | 0.14(0.12,0.16) | -3.58(-3.82,-3.35) |
| Grand Duchy of Luxembourg | 0.15(0.14,0.17) | 0.10(0.08,0.12) | -34.33(-49.93,-17.32) | 0.23(0.21,0.26) | 0.10(0.08,0.12) | -4.15(-4.66,-3.65) |
| Republic of Malta | 0.18(0.13,0.21) | 0.19(0.15,0.25) | 8.68(-21.91,54.92) | 0.20(0.15,0.24) | 0.30(0.23,0.39) | -0.84(-1.63,-0.03) |
| Kingdom of the Netherlands | 7.01(6.18,7.89) | 3.17(2.61,3.73) | -54.82(-63.82,-44.49) | 0.26(0.23,0.29) | 0.12(0.10,0.14) | -2.50(-2.77,-2.24) |
| Kingdom of Norway | 2.41(2.25,2.60) | 0.69(0.60,0.79) | -71.63(-75.35,-67.19) | 0.30(0.28,0.33) | 0.07(0.06,0.09) | -4.13(-4.55,-3.70) |
| Portuguese Republic | 9.08(7.86,10.43) | 1.89(1.53,2.27) | -79.19(-83.98,-73.26) | 0.43(0.37,0.49) | 0.14(0.11,0.17) | -4.59(-4.99,-4.18) |
| Kingdom of Spain | 25.05(21.26,28.93) | 8.08(6.45,10.09) | -67.75(-74.99,-58.28) | 0.32(0.27,0.37) | 0.12(0.10,0.16) | -3.53(-3.75,-3.30) |
| Kingdom of Sweden | 2.74(2.45,3.09) | 1.30(1.11,1.52) | -52.63(-61.54,-41.75) | 0.18(0.16,0.20) | 0.07(0.06,0.08) | -2.03(-2.68,-1.36) |
| Swiss Confederation | 3.06(2.69,3.52) | 1.28(1.09,1.51) | -58.17(-65.92,-48.25) | 0.26(0.23,0.30) | 0.10(0.08,0.11) | -3.65(-3.97,-3.33) |
| United Kingdom of Great Britain and Northern Ireland | 20.75(20.13,21.37) | 9.97(9.30,10.54) | -51.96(-55.22,-48.87) | 0.19(0.18,0.20) | 0.08(0.08,0.09) | -2.60(-2.90,-2.31) |
| Argentine Republic | 43.52(38.20,49.52) | 22.79(18.75,27.40) | -47.63(-58.68,-33.60) | 0.43(0.38,0.49) | 0.22(0.18,0.27) | -1.73(-2.04,-1.42) |
| Republic of Chile | 13.76(12.21,15.52) | 4.85(4.09,5.80) | -64.75(-71.63,-56.86) | 0.35(0.31,0.39) | 0.13(0.11,0.16) | -2.93(-3.25,-2.61) |
| Eastern Republic of Uruguay | 3.42(2.93,3.98) | 1.55(1.26,1.92) | -54.69(-65.22,-41.23) | 0.42(0.36,0.49) | 0.24(0.19,0.29) | -2.25(-2.79,-1.71) |
| Canada | 15.07(13.25,17.10) | 4.38(3.61,5.29) | -70.93(-77.41,-63.36) | 0.26(0.23,0.30) | 0.07(0.06,0.09) | -5.01(-5.45,-4.57) |
| United States of America | 105.77(103.53,108.28) | 42.87(39.86,45.77) | -59.47(-62.33,-56.59) | 0.19(0.19,0.19) | 0.07(0.07,0.08) | -3.19(-3.36,-3.03) |
| Antigua and Barbuda | 0.07(0.06,0.08) | 0.05(0.05,0.06) | -23.44(-37.35,-5.16) | 0.39(0.32,0.45) | 0.32(0.28,0.36) | -0.60(-1.13,-0.07) |
| Commonwealth of the Bahamas | 0.43(0.37,0.51) | 0.27(0.22,0.34) | -37.19(-51.83,-17.66) | 0.54(0.46,0.63) | 0.34(0.27,0.42) | -1.79(-2.21,-1.37) |
| Barbados | 0.58(0.50,0.66) | 0.21(0.16,0.28) | -62.88(-72.20,-50.25) | 0.92(0.81,1.06) | 0.45(0.33,0.60) | -1.12(-1.63,-0.61) |
| Belize | 0.43(0.38,0.48) | 0.23(0.19,0.27) | -47.36(-56.71,-36.00) | 0.53(0.47,0.59) | 0.18(0.16,0.22) | -2.69(-3.00,-2.38) |
| Republic of Cuba | 17.41(15.54,19.53) | 5.48(4.37,6.70) | -68.52(-75.23,-60.26) | 0.70(0.62,0.78) | 0.31(0.25,0.38) | -2.07(-2.42,-1.72) |
| Commonwealth of Dominica | 0.19(0.15,0.24) | 0.17(0.12,0.23) | -12.60(-42.99,21.60) | 0.77(0.60,0.98) | 1.22(0.89,1.66) | 1.81(1.56,2.06) |
| Dominican Republic | 19.44(14.24,24.51) | 9.70(6.49,14.00) | -50.10(-67.28,-25.64) | 0.72(0.53,0.91) | 0.33(0.22,0.48) | -2.64(-2.95,-2.33) |
| Grenada | 0.49(0.39,0.60) | 0.22(0.17,0.27) | -55.78(-66.93,-41.79) | 1.46(1.17,1.80) | 0.99(0.79,1.22) | -0.67(-0.99,-0.34) |
| Republic of Guyana | 1.44(1.16,1.73) | 0.81(0.62,1.04) | -43.96(-59.51,-24.70) | 0.49(0.39,0.59) | 0.38(0.29,0.49) | 0.77(0.24,1.29) |
| Republic of Haiti | 76.38(21.40,122.88) | 65.39(28.43,105.51) | -14.39(-44.27,39.50) | 2.82(0.79,4.53) | 1.50(0.65,2.42) | -1.57(-1.84,-1.30) |
| Jamaica | 9.30(7.80,10.80) | 3.06(2.40,3.88) | -67.03(-74.98,-57.37) | 1.11(0.93,1.29) | 0.52(0.41,0.66) | -2.31(-2.66,-1.96) |
| Saint Lucia | 0.44(0.38,0.53) | 0.19(0.15,0.24) | -57.53(-68.25,-44.18) | 0.86(0.73,1.02) | 0.64(0.51,0.79) | -1.37(-1.62,-1.12) |
| Saint Vincent and the Grenadines | 0.54(0.45,0.63) | 0.23(0.19,0.27) | -58.02(-66.49,-47.79) | 1.31(1.10,1.54) | 0.90(0.77,1.08) | -0.96(-1.33,-0.59) |
| Republic of Suriname | 0.98(0.58,1.26) | 0.73(0.50,0.99) | -25.64(-54.51,34.01) | 0.75(0.45,0.97) | 0.51(0.35,0.69) | -1.00(-1.33,-0.67) |
| Republic of Trinidad and Tobago | 3.33(2.91,3.79) | 1.08(0.86,1.37) | -67.60(-74.93,-57.41) | 0.82(0.72,0.93) | 0.40(0.31,0.50) | -1.91(-2.32,-1.50) |
| Plurinational State of Bolivia | 33.77(23.90,45.46) | 18.99(13.89,25.59) | -43.76(-64.13,-7.28) | 1.26(0.89,1.69) | 0.54(0.40,0.73) | -2.79(-2.90,-2.68) |
| Republic of Ecuador | 18.16(16.13,20.74) | 14.85(11.90,18.46) | -18.20(-35.91,3.20) | 0.47(0.42,0.54) | 0.29(0.23,0.36) | -1.41(-1.98,-0.83) |
| Republic of Peru | 67.44(55.01,88.54) | 36.35(26.49,48.95) | -46.09(-62.45,-21.79) | 0.81(0.66,1.07) | 0.38(0.28,0.51) | -2.19(-2.38,-2.00) |
| Republic of Colombia | 78.41(67.78,89.64) | 29.99(23.79,37.72) | -61.75(-70.58,-51.29) | 0.67(0.58,0.77) | 0.28(0.22,0.36) | -1.87(-2.25,-1.48) |
| Republic of Costa Rica | 5.13(4.51,5.96) | 3.23(2.60,4.08) | -37.06(-51.32,-17.07) | 0.46(0.40,0.53) | 0.32(0.26,0.40) | -1.20(-1.38,-1.02) |
| Republic of El Salvador | 8.27(6.52,10.31) | 3.57(2.74,4.62) | -56.80(-68.36,-39.31) | 0.38(0.30,0.48) | 0.20(0.15,0.25) | -1.90(-2.15,-1.66) |
| Republic of Guatemala | 16.75(15.20,18.50) | 7.87(6.46,9.68) | -53.03(-62.10,-41.36) | 0.41(0.37,0.46) | 0.16(0.13,0.20) | -2.16(-2.46,-1.85) |
| Republic of Honduras | 6.87(5.21,8.87) | 3.23(1.72,5.46) | -52.94(-74.30,-18.15) | 0.31(0.24,0.40) | 0.10(0.05,0.17) | -3.85(-4.10,-3.59) |
| United Mexican States | 171.56(156.59,189.57) | 66.86(57.50,77.77) | -61.03(-67.70,-53.37) | 0.51(0.47,0.57) | 0.21(0.18,0.24) | -2.41(-2.72,-2.10) |
| Republic of Nicaragua | 9.30(7.05,12.04) | 2.92(2.21,3.89) | -68.61(-78.17,-55.42) | 0.51(0.39,0.66) | 0.15(0.11,0.20) | -3.11(-3.39,-2.83) |
| Republic of Panama | 3.99(3.45,4.62) | 4.44(3.62,5.33) | 11.39(-11.77,39.84) | 0.48(0.41,0.55) | 0.38(0.31,0.46) | -0.64(-0.84,-0.44) |
| Bolivarian Republic of Venezuela | 36.42(33.96,39.08) | 26.56(20.83,33.08) | -27.06(-42.67,-7.74) | 0.51(0.48,0.55) | 0.40(0.31,0.50) | -0.76(-1.23,-0.29) |
| Federative Republic of Brazil | 288.19(256.27,319.50) | 95.73(77.79,112.72) | -66.78(-72.56,-60.22) | 0.55(0.49,0.62) | 0.20(0.16,0.23) | -2.75(-3.22,-2.27) |
| Republic of Paraguay | 8.08(6.11,10.51) | 6.50(4.60,9.20) | -19.65(-47.28,21.69) | 0.48(0.37,0.63) | 0.32(0.23,0.46) | -0.97(-1.40,-0.54) |
| People's Democratic Republic of Algeria | 80.81(58.42,104.42) | 43.43(31.43,58.75) | -46.26(-63.13,-23.97) | 0.75(0.54,0.97) | 0.33(0.24,0.44) | -2.51(-2.64,-2.37) |
| Kingdom of Bahrain | 0.49(0.37,0.63) | 0.43(0.31,0.59) | -12.36(-40.42,25.47) | 0.30(0.23,0.39) | 0.15(0.11,0.20) | -2.41(-2.80,-2.03) |
| Arab Republic of Egypt | 96.36(72.10,162.04) | 42.82(26.69,95.40) | -55.56(-71.29,-28.38) | 0.43(0.33,0.73) | 0.12(0.07,0.26) | -3.99(-4.58,-3.39) |
| Islamic Republic of Iran | 93.31(72.54,120.68) | 40.67(31.86,53.15) | -56.42(-70.41,-36.29) | 0.37(0.29,0.48) | 0.20(0.16,0.26) | -1.30(-1.83,-0.76) |
| Republic of Iraq | 68.65(40.42,97.35) | 40.83(28.44,57.27) | -40.52(-63.94,12.70) | 0.83(0.49,1.18) | 0.30(0.21,0.43) | -2.79(-3.14,-2.44) |
| Hashemite Kingdom of Jordan | 10.64(7.93,14.32) | 15.65(11.59,21.23) | 47.07(-4.06,118.86) | 0.65(0.49,0.88) | 0.43(0.32,0.58) | -1.68(-2.06,-1.29) |
| State of Kuwait | 4.11(3.54,4.80) | 1.97(1.53,2.43) | -51.99(-62.75,-37.43) | 0.74(0.64,0.87) | 0.23(0.18,0.29) | -1.85(-2.78,-0.92) |
| Lebanese Republic | 6.46(4.49,8.71) | 3.67(2.56,5.19) | -43.15(-63.42,-6.19) | 0.62(0.43,0.83) | 0.29(0.20,0.41) | -2.58(-2.91,-2.25) |
| State of Libya | 13.43(9.08,17.79) | 12.68(8.89,17.41) | -5.56(-38.44,50.03) | 0.74(0.50,0.98) | 0.85(0.60,1.17) | 0.95(0.64,1.26) |
| Kingdom of Morocco | 54.91(35.39,77.41) | 23.15(15.69,34.23) | -57.83(-74.69,-23.04) | 0.56(0.36,0.79) | 0.24(0.16,0.35) | -2.37(-2.59,-2.15) |
| Palestine | 3.36(2.36,4.59) | 3.84(2.87,5.17) | 14.46(-26.89,85.01) | 0.35(0.24,0.47) | 0.21(0.15,0.28) | -0.96(-1.40,-0.51) |
| Sultanate of Oman | 3.63(2.36,5.21) | 2.61(1.91,3.38) | -28.05(-56.26,14.33) | 0.43(0.28,0.62) | 0.21(0.16,0.28) | -1.13(-1.57,-0.69) |
| State of Qatar | 0.23(0.16,0.33) | 0.49(0.35,0.71) | 113.51(33.45,261.47) | 0.18(0.13,0.26) | 0.10(0.07,0.14) | -1.25(-1.76,-0.74) |
| Kingdom of Saudi Arabia | 31.17(17.80,44.93) | 11.24(6.76,16.13) | -63.93(-78.91,-38.30) | 0.48(0.27,0.69) | 0.15(0.09,0.21) | -3.74(-3.83,-3.64) |
| Syrian Arab Republic | 23.30(17.08,29.78) | 7.67(5.74,10.37) | -67.10(-77.78,-51.43) | 0.39(0.29,0.50) | 0.21(0.16,0.28) | -1.97(-2.60,-1.33) |
| Republic of Tunisia | 28.35(19.95,36.89) | 9.67(6.90,13.23) | -65.90(-78.93,-46.09) | 0.91(0.64,1.19) | 0.35(0.25,0.48) | -2.77(-2.88,-2.66) |
| Republic of Turkey | 154.72(94.18,210.62) | 47.50(35.76,62.42) | -69.30(-79.68,-48.26) | 0.76(0.46,1.03) | 0.26(0.19,0.34) | -3.43(-3.71,-3.14) |
| United Arab Emirates | 1.69(1.07,2.57) | 1.45(1.06,2.00) | -14.43(-48.79,48.55) | 0.29(0.18,0.44) | 0.11(0.08,0.15) | -2.74(-3.09,-2.39) |
| Republic of Yemen | 32.70(18.28,61.87) | 34.70(22.84,54.89) | 6.14(-32.43,105.87) | 0.46(0.26,0.87) | 0.25(0.17,0.40) | -1.85(-1.98,-1.72) |
| Islamic Republic of Afghanistan | 29.34(10.60,48.88) | 50.89(32.22,83.55) | 73.48(14.58,281.33) | 0.68(0.25,1.13) | 0.36(0.23,0.59) | -1.71(-1.99,-1.44) |
| People's Republic of Bangladesh | 555.86(242.73,824.00) | 210.34(138.55,351.22) | -62.16(-75.30,-24.24) | 1.14(0.50,1.68) | 0.46(0.30,0.77) | -2.70(-2.84,-2.55) |
| Kingdom of Bhutan | 2.31(1.04,3.68) | 0.82(0.47,1.55) | -64.47(-84.12,29.04) | 0.88(0.40,1.40) | 0.44(0.25,0.83) | -2.68(-2.99,-2.37) |
| Republic of India | 1979.21(1182.95,2650.46) | 807.21(631.39,1031.71) | -59.22(-69.98,-20.91) | 0.61(0.36,0.81) | 0.22(0.17,0.28) | -3.27(-3.50,-3.04) |
| Federal Democratic Republic of Nepal | 72.53(32.95,109.73) | 29.41(18.63,50.36) | -59.44(-74.20,-9.80) | 0.86(0.39,1.30) | 0.32(0.20,0.55) | -2.86(-3.10,-2.63) |
| Islamic Republic of Pakistan | 567.15(409.30,751.55) | 1091.79(754.34,1552.66) | 92.51(29.80,206.88) | 1.15(0.83,1.53) | 1.28(0.88,1.82) | 0.78(0.60,0.95) |
| Republic of Angola | 54.67(12.01,90.56) | 59.57(36.46,88.40) | 8.97(-31.78,298.55) | 1.16(0.25,1.92) | 0.39(0.24,0.58) | -3.31(-3.62,-3.00) |
| Central African Republic | 10.68(3.09,17.69) | 13.52(6.85,21.15) | 26.51(-18.85,135.89) | 0.87(0.25,1.45) | 0.59(0.30,0.93) | -1.00(-1.15,-0.85) |
| Republic of the Congo | 6.76(2.94,10.33) | 6.60(4.67,8.99) | -2.50(-35.61,99.32) | 0.64(0.28,0.98) | 0.34(0.24,0.47) | -1.97(-2.18,-1.75) |
| Democratic Republic of the Congo | 129.18(43.83,189.18) | 114.56(73.17,164.84) | -11.32(-41.36,117.88) | 0.73(0.25,1.07) | 0.30(0.19,0.43) | -2.38(-2.60,-2.16) |
| Republic of Equatorial Guinea | 1.52(0.52,2.38) | 2.07(1.02,3.98) | 36.27(-35.82,266.47) | 0.77(0.27,1.21) | 0.35(0.18,0.68) | -3.21(-3.55,-2.86) |
| Gabonese Republic | 2.04(1.25,2.92) | 2.52(1.62,3.73) | 23.48(-24.44,104.16) | 0.50(0.31,0.72) | 0.39(0.25,0.58) | -0.21(-0.43,0.01) |
| Republic of Burundi | 67.86(42.04,104.56) | 54.85(29.97,100.40) | -19.17(-49.13,70.61) | 2.59(1.60,3.99) | 0.94(0.51,1.71) | -2.76(-2.99,-2.53) |
| Union of the Comoros | 3.43(1.83,4.96) | 2.74(1.79,4.10) | -20.13(-52.22,51.02) | 1.61(0.86,2.33) | 1.14(0.75,1.71) | -1.22(-1.59,-0.85) |
| Republic of Djibouti | 2.55(1.68,3.67) | 3.84(2.30,6.37) | 50.96(-6.59,133.85) | 1.46(0.96,2.11) | 0.93(0.56,1.54) | -1.37(-1.76,-0.98) |
| State of Eritrea | 28.65(18.82,39.49) | 28.84(17.36,49.37) | 0.63(-37.64,94.82) | 1.80(1.18,2.48) | 1.14(0.69,1.96) | -1.40(-1.56,-1.23) |
| Federal Democratic Republic of Ethiopia | 620.68(166.21,965.12) | 447.56(311.31,618.16) | -27.89(-50.13,130.37) | 2.55(0.68,3.96) | 1.01(0.70,1.39) | -3.31(-3.59,-3.04) |
| Republic of Kenya | 63.79(42.33,91.47) | 71.50(54.02,90.97) | 12.08(-20.97,68.57) | 0.57(0.38,0.82) | 0.38(0.29,0.49) | -0.15(-0.65,0.36) |
| Republic of Madagascar | 90.32(64.41,118.13) | 97.76(66.38,139.10) | 8.24(-26.10,76.76) | 1.66(1.18,2.17) | 0.83(0.57,1.19) | -1.75(-1.96,-1.55) |
| Republic of Malawi | 283.60(205.69,374.14) | 260.70(131.72,542.44) | -8.08(-51.79,78.16) | 6.23(4.52,8.22) | 3.21(1.62,6.68) | -1.79(-2.06,-1.51) |
| Republic of Mauritius | 0.27(0.24,0.29) | 0.28(0.25,0.32) | 6.59(-9.54,24.68) | 0.08(0.07,0.09) | 0.14(0.12,0.15) | 0.53(-0.11,1.17) |
| Republic of Mozambique | 21.67(11.84,53.57) | 20.69(10.12,49.83) | -4.51(-46.03,135.48) | 0.35(0.19,0.86) | 0.15(0.07,0.35) | -2.36(-2.67,-2.05) |
| Republic of Rwanda | 97.62(65.41,135.98) | 53.65(36.53,88.32) | -45.04(-65.15,0.40) | 2.88(1.93,4.01) | 1.08(0.73,1.78) | -3.72(-4.03,-3.42) |
| Republic of Seychelles | 0.05(0.04,0.07) | 0.03(0.02,0.04) | -41.31(-61.87,-11.29) | 0.21(0.16,0.30) | 0.13(0.09,0.17) | 0.05(-0.92,1.04) |
| Federal Republic of Somalia | 64.28(34.92,94.42) | 90.18(45.84,143.14) | 40.29(-11.62,136.49) | 1.65(0.90,2.42) | 0.87(0.44,1.39) | -1.63(-2.01,-1.25) |
| United Republic of Tanzania | 269.97(186.17,359.59) | 333.32(220.07,512.39) | 23.46(-19.92,93.04) | 2.24(1.54,2.98) | 1.37(0.90,2.10) | -1.03(-1.31,-0.76) |
| Republic of Uganda | 210.08(144.27,292.45) | 445.76(270.18,722.69) | 112.19(36.53,228.96) | 2.50(1.71,3.47) | 2.25(1.36,3.64) | -0.18(-0.43,0.06) |
| Republic of Zambia | 91.12(62.94,120.94) | 99.18(59.60,165.15) | 8.85(-33.26,120.57) | 2.43(1.68,3.22) | 1.20(0.72,2.00) | -2.13(-2.28,-1.98) |
| Republic of Botswana | 1.77(1.18,2.65) | 3.04(1.97,4.52) | 72.10(10.12,166.41) | 0.30(0.20,0.45) | 0.44(0.28,0.65) | 1.94(1.55,2.32) |
| Kingdom of Lesotho | 1.83(1.23,2.77) | 3.03(1.94,4.40) | 65.05(6.43,160.69) | 0.27(0.18,0.41) | 0.48(0.31,0.70) | 2.86(2.49,3.23) |
| Republic of Namibia | 3.27(2.08,4.71) | 5.59(3.40,8.46) | 70.65(5.75,202.76) | 0.55(0.35,0.78) | 0.68(0.41,1.02) | 1.68(1.10,2.25) |
| Republic of South Africa | 44.08(30.15,65.90) | 48.57(39.72,60.58) | 10.19(-17.56,49.16) | 0.32(0.22,0.48) | 0.32(0.26,0.40) | -0.31(-0.67,0.05) |
| Kingdom of Eswatini | 1.84(1.07,2.83) | 2.16(1.37,3.36) | 17.29(-19.61,84.07) | 0.48(0.28,0.73) | 0.52(0.33,0.81) | 0.65(0.39,0.91) |
| Republic of Zimbabwe | 22.28(10.81,32.19) | 67.60(30.88,97.37) | 203.42(90.60,355.63) | 0.46(0.22,0.67) | 1.07(0.49,1.55) | 5.16(4.00,6.34) |
| Republic of Benin | 32.14(21.06,46.68) | 59.24(37.44,84.64) | 84.30(9.93,209.20) | 1.33(0.87,1.93) | 0.97(0.62,1.39) | -0.76(-0.91,-0.62) |
| Burkina Faso | 61.87(40.09,88.88) | 105.36(72.71,145.54) | 70.29(13.39,166.31) | 1.31(0.85,1.88) | 1.02(0.70,1.40) | -0.46(-0.66,-0.27) |
| Republic of Cameroon | 61.31(40.76,84.70) | 143.66(95.96,198.47) | 134.34(52.81,262.46) | 1.26(0.83,1.73) | 1.07(0.71,1.47) | -0.14(-0.35,0.07) |
| Republic of Cabo Verde | 0.31(0.18,0.52) | 0.99(0.42,1.41) | 220.06(-5.51,539.59) | 0.20(0.11,0.33) | 0.69(0.29,0.98) | 2.75(1.78,3.74) |
| Republic of Chad | 31.27(19.10,47.09) | 90.17(56.81,130.48) | 188.40(81.31,367.91) | 1.07(0.65,1.61) | 1.00(0.63,1.45) | 0.15(0.01,0.28) |
| Republic of Côte d'Ivoire | 75.48(53.49,103.92) | 130.80(87.43,214.47) | 73.29(12.82,168.90) | 1.32(0.94,1.82) | 1.13(0.76,1.85) | -0.17(-0.40,0.05) |
| Republic of the Gambia | 6.58(4.41,9.09) | 8.83(5.75,14.67) | 34.12(-15.21,110.81) | 1.43(0.96,1.97) | 0.89(0.58,1.48) | -1.69(-1.98,-1.40) |
| Republic of Ghana | 126.85(59.36,193.56) | 62.11(40.45,103.52) | -51.03(-73.12,28.90) | 1.89(0.88,2.88) | 0.48(0.31,0.80) | -5.70(-6.63,-4.76) |
| Republic of Guinea | 48.16(32.12,66.84) | 49.81(30.90,98.71) | 3.44(-35.35,96.17) | 1.75(1.17,2.43) | 0.82(0.51,1.63) | -1.86(-2.04,-1.69) |
| Republic of Guinea-Bissau | 8.13(5.11,12.08) | 6.98(4.32,10.97) | -14.15(-44.14,58.67) | 1.69(1.06,2.50) | 0.78(0.48,1.22) | -2.23(-2.57,-1.89) |
| Republic of Liberia | 20.78(11.78,28.67) | 19.02(12.44,26.91) | -8.44(-44.18,67.15) | 1.84(1.04,2.54) | 0.87(0.57,1.23) | -2.29(-2.98,-1.58) |
| Republic of Mali | 46.90(31.31,68.65) | 59.75(36.03,109.76) | 27.39(-19.18,128.48) | 1.14(0.76,1.66) | 0.52(0.31,0.95) | -2.22(-2.39,-2.04) |
| Islamic Republic of Mauritania | 7.40(5.00,10.48) | 10.27(6.69,14.34) | 38.84(-12.22,117.45) | 0.80(0.54,1.13) | 0.55(0.36,0.77) | -1.35(-1.63,-1.06) |
| Republic of the Niger | 76.46(43.07,111.70) | 89.88(52.79,141.17) | 17.55(-28.31,125.49) | 1.88(1.06,2.75) | 0.70(0.41,1.11) | -3.18(-3.43,-2.93) |
| Federal Republic of Nigeria | 708.11(444.85,987.26) | 1285.39(732.11,1831.87) | 81.52(28.36,230.73) | 1.81(1.14,2.52) | 1.27(0.72,1.80) | -0.89(-1.02,-0.76) |
| Democratic Republic of Sao Tome and Principe | 0.75(0.53,1.07) | 0.34(0.20,0.57) | -54.44(-73.84,-10.12) | 1.32(0.94,1.88) | 0.44(0.26,0.73) | -3.07(-3.48,-2.65) |
| Republic of Senegal | 47.70(32.48,66.64) | 39.22(26.76,58.64) | -17.78(-46.50,27.02) | 1.31(0.89,1.83) | 0.62(0.42,0.92) | -2.36(-2.61,-2.12) |
| Republic of Sierra Leone | 31.09(18.74,44.05) | 33.29(23.04,48.27) | 7.07(-32.08,100.42) | 1.72(1.03,2.43) | 0.93(0.64,1.35) | -1.89(-2.05,-1.73) |
| Togolese Republic | 17.85(12.43,25.31) | 22.37(14.78,34.16) | 25.35(-19.33,111.58) | 1.01(0.71,1.44) | 0.68(0.45,1.03) | -1.17(-1.31,-1.02) |
| American Samoa | 0.03(0.02,0.04) | 0.03(0.02,0.05) | 23.42(-29.94,117.85) | 0.14(0.10,0.21) | 0.24(0.15,0.38) | 2.22(1.95,2.49) |
| Bermuda | 0.07(0.06,0.08) | 0.03(0.02,0.03) | -63.30(-72.74,-51.63) | 0.58(0.48,0.70) | 0.30(0.24,0.37) | -1.72(-2.09,-1.34) |
| Cook Islands | 0.01(0.01,0.01) | 0.00(0.00,0.01) | -41.86(-73.76,35.54) | 0.12(0.08,0.17) | 0.12(0.07,0.23) | -1.46(-2.04,-0.87) |
| Greenland | 0.09(0.04,0.12) | 0.02(0.01,0.03) | -77.15(-86.07,-60.29) | 0.60(0.28,0.85) | 0.17(0.11,0.24) | -3.90(-4.40,-3.40) |
| Guam | 0.11(0.09,0.14) | 0.07(0.05,0.09) | -38.22(-56.14,-13.91) | 0.27(0.22,0.33) | 0.19(0.14,0.26) | 1.52(0.57,2.49) |
| Principality of Monaco | 0.01(0.01,0.01) | 0.01(0.01,0.01) | 14.69(-21.36,85.08) | 0.24(0.16,0.35) | 0.19(0.14,0.27) | -1.99(-2.50,-1.48) |
| Republic of Nauru | 0.01(0.01,0.02) | 0.02(0.01,0.02) | 19.70(-26.63,101.30) | 0.31(0.21,0.44) | 0.40(0.25,0.59) | 0.70(0.22,1.17) |
| Republic of Niue | 0.00(0.00,0.00) | 0.01(0.00,0.01) | 223.90(103.13,441.23) | 0.23(0.15,0.33) | 1.54(1.04,2.30) | 2.59(1.31,3.90) |
| Northern Mariana Islands | 0.01(0.01,0.02) | 0.01(0.01,0.02) | 47.23(-24.01,187.68) | 0.07(0.04,0.14) | 0.11(0.07,0.17) | 2.76(1.93,3.60) |
| Republic of Palau | 0.01(0.00,0.01) | 0.00(0.00,0.00) | -48.92(-66.34,-17.61) | 0.11(0.08,0.16) | 0.08(0.05,0.13) | -0.90(-1.08,-0.72) |
| Puerto Rico | 5.28(4.64,6.03) | 0.66(0.55,0.79) | -87.42(-90.09,-83.98) | 0.53(0.47,0.61) | 0.15(0.12,0.18) | -3.57(-3.83,-3.32) |
| Saint Kitts and Nevis | 0.11(0.10,0.12) | 0.05(0.04,0.06) | -53.68(-61.91,-43.50) | 0.78(0.69,0.88) | 0.52(0.44,0.61) | -1.02(-1.23,-0.82) |
| Republic of San Marino | 0.03(0.02,0.04) | 0.01(0.01,0.01) | -65.66(-77.09,-42.95) | 0.72(0.52,0.96) | 0.23(0.16,0.32) | -3.36(-3.49,-3.23) |
| Tokelau | 0.00(0.00,0.00) | 0.01(0.00,0.01) | 271.14(112.34,570.25) | 0.23(0.15,0.35) | 1.34(0.84,2.09) | 1.48(-0.22,3.22) |
| Tuvalu | 0.01(0.01,0.02) | 0.01(0.01,0.01) | -34.23(-60.50,22.97) | 0.32(0.18,0.47) | 0.19(0.14,0.28) | -1.23(-1.39,-1.06) |
| United States Virgin Islands | 0.20(0.15,0.27) | 0.02(0.01,0.03) | -89.44(-94.07,-82.19) | 0.63(0.47,0.85) | 0.16(0.10,0.25) | -3.46(-3.92,-3.00) |
| Republic of South Sudan | 54.92(31.92,78.53) | 82.23(50.74,116.56) | 49.73(3.96,122.68) | 2.09(1.22,2.99) | 1.91(1.18,2.71) | 0.14(-0.57,0.86) |
| Republic of Sudan | 92.01(36.99,182.62) | 73.35(48.25,103.49) | -20.28(-60.63,77.56) | 1.03(0.42,2.05) | 0.44(0.29,0.62) | -2.43(-2.55,-2.31) |

**Table S5: Disability-adjusted life years of Non-Hodgkin Lymphoma in children between 1990 and 2021 in 204 countries.**

| location | Number (95% UI) | | Percentage change (95% UI) | Rate (95% UI) | | EAPC (95% CI) |
| --- | --- | --- | --- | --- | --- | --- |
|  | 1990 | 2021 |  | 1990 | 2021 |  |
| People's Republic of China | 205190.83(170360.96,250898.13) | 41207.39(32904.56,52922.11) | -79.92(-84.52,-72.19) | 64.45(53.51,78.81) | 15.87(12.67,20.38) | -4.54(-4.90,-4.17) |
| Democratic People's Republic of Korea | 2124.14(1345.88,3095.05) | 829.87(543.64,1313.31) | -60.93(-75.11,-37.90) | 35.70(22.62,52.02) | 17.38(11.39,27.51) | -2.03(-2.29,-1.77) |
| Taiwan (Province of China) | 1844.53(1647.89,2042.67) | 454.18(386.92,535.98) | -75.38(-79.28,-70.28) | 33.49(29.92,37.08) | 15.41(13.13,18.19) | -2.49(-2.85,-2.12) |
| Kingdom of Cambodia | 2426.64(1012.02,3600.11) | 1302.09(911.00,2090.62) | -46.34(-66.17,19.56) | 52.06(21.71,77.24) | 25.45(17.80,40.86) | -2.66(-2.91,-2.41) |
| Republic of Indonesia | 26032.64(14063.48,39380.34) | 13940.58(9909.76,21671.44) | -46.45(-61.14,-14.25) | 38.43(20.76,58.14) | 20.72(14.73,32.21) | -1.86(-1.91,-1.81) |
| Lao People's Democratic Republic | 1154.45(391.52,1820.40) | 645.70(415.24,1030.18) | -44.07(-65.32,35.30) | 62.64(21.24,98.77) | 28.12(18.08,44.86) | -2.65(-2.72,-2.59) |
| Malaysia | 1879.71(1321.95,2501.76) | 1157.82(809.61,1537.51) | -38.40(-62.88,-3.81) | 28.60(20.11,38.06) | 15.21(10.63,20.19) | -1.60(-1.94,-1.26) |
| Republic of Maldives | 38.74(17.96,56.72) | 13.06(9.09,19.25) | -66.30(-80.56,-7.40) | 36.89(17.10,53.99) | 13.03(9.08,19.22) | -2.94(-3.14,-2.73) |
| Republic of the Union of Myanmar | 10669.60(3643.74,16736.69) | 4977.54(3219.87,7727.28) | -53.35(-68.46,2.05) | 72.21(24.66,113.27) | 31.88(20.62,49.49) | -2.88(-3.03,-2.73) |
| Republic of the Philippines | 12014.40(7978.29,15961.46) | 8519.00(6917.10,11427.10) | -29.09(-44.56,3.89) | 47.65(31.64,63.30) | 25.06(20.35,33.61) | -1.53(-1.71,-1.34) |
| Democratic Socialist Republic of Sri Lanka | 1326.16(972.51,1719.08) | 514.69(340.67,733.61) | -61.19(-74.13,-36.96) | 23.97(17.58,31.07) | 10.08(6.67,14.37) | -2.75(-3.32,-2.18) |
| Kingdom of Thailand | 7295.17(5625.03,9743.55) | 1820.32(1341.63,2862.39) | -75.05(-82.69,-64.51) | 43.27(33.37,57.80) | 18.64(13.74,29.31) | -3.37(-3.78,-2.96) |
| Democratic Republic of Timor-Leste | 173.19(63.32,273.43) | 110.62(77.38,170.67) | -36.13(-58.65,51.10) | 52.07(19.04,82.20) | 21.25(14.86,32.78) | -3.05(-3.48,-2.63) |
| Socialist Republic of Viet Nam | 10639.43(7062.76,14546.50) | 6328.50(4163.61,9040.53) | -40.52(-62.74,6.11) | 40.13(26.64,54.87) | 25.56(16.81,36.51) | -1.14(-1.30,-0.98) |
| Republic of Fiji | 35.26(24.19,54.22) | 62.52(40.12,93.38) | 77.33(1.38,199.00) | 12.53(8.60,19.26) | 22.94(14.72,34.26) | 2.28(2.03,2.53) |
| Republic of Kiribati | 2.91(1.65,4.06) | 3.06(1.49,5.14) | 5.05(-41.36,89.39) | 9.86(5.57,13.76) | 7.28(3.53,12.23) | -1.07(-1.26,-0.87) |
| Republic of the Marshall Islands | 2.54(1.69,3.84) | 3.52(2.22,5.56) | 38.84(-23.14,159.51) | 11.57(7.72,17.48) | 20.19(12.71,31.87) | 1.71(1.25,2.16) |
| Federated States of Micronesia | 8.92(5.57,13.38) | 5.39(3.47,8.33) | -39.58(-64.18,12.37) | 19.42(12.13,29.13) | 17.61(11.32,27.21) | -0.20(-0.36,-0.03) |
| Independent State of Papua New Guinea | 410.05(228.26,642.58) | 1070.85(646.55,1672.96) | 161.15(71.49,293.76) | 24.12(13.43,37.80) | 27.34(16.51,42.71) | 0.58(0.33,0.83) |
| Independent State of Samoa | 27.71(18.48,39.19) | 21.08(13.00,35.43) | -23.93(-53.33,28.75) | 38.89(25.94,55.00) | 26.37(16.26,44.31) | -1.25(-1.36,-1.13) |
| Solomon Islands | 20.41(11.11,32.23) | 43.07(28.86,64.78) | 111.03(27.04,303.28) | 13.11(7.13,20.70) | 16.56(11.10,24.91) | 0.89(0.59,1.20) |
| Kingdom of Tonga | 19.97(13.48,28.58) | 20.05(12.57,32.48) | 0.38(-40.96,84.99) | 47.77(32.24,68.35) | 51.37(32.22,83.24) | -0.14(-0.57,0.29) |
| Republic of Vanuatu | 7.23(4.29,11.55) | 15.97(10.41,23.55) | 121.01(40.48,269.80) | 10.61(6.31,16.96) | 13.70(8.93,20.21) | 1.13(0.69,1.57) |
| Republic of Armenia | 262.50(188.53,319.41) | 114.97(91.47,147.01) | -56.20(-69.37,-29.93) | 25.16(18.07,30.62) | 19.41(15.44,24.82) | 0.74(0.13,1.36) |
| Republic of Azerbaijan | 961.78(656.68,1340.44) | 663.48(428.70,1033.46) | -31.02(-58.88,17.69) | 39.63(27.06,55.23) | 28.11(18.16,43.78) | -1.60(-1.94,-1.27) |
| Georgia | 803.69(577.28,1051.92) | 111.48(78.98,149.40) | -86.13(-91.09,-76.42) | 58.72(42.18,76.85) | 15.15(10.73,20.30) | -4.09(-4.80,-3.38) |
| Republic of Kazakhstan | 2966.93(2592.46,3355.41) | 814.86(658.68,1002.07) | -72.54(-79.00,-64.61) | 57.10(49.89,64.57) | 15.02(12.14,18.47) | -4.33(-4.78,-3.89) |
| Kyrgyz Republic | 513.22(404.43,673.66) | 335.06(253.97,426.31) | -34.71(-56.14,-8.01) | 30.59(24.11,40.16) | 14.73(11.17,18.74) | -2.29(-2.81,-1.76) |
| Mongolia | 757.68(490.31,1057.93) | 231.06(161.26,324.86) | -69.50(-81.49,-46.83) | 84.19(54.48,117.55) | 21.26(14.84,29.90) | -4.61(-5.13,-4.10) |
| Republic of Tajikistan | 2499.28(1115.38,3667.62) | 2145.82(1439.26,3115.06) | -14.14(-47.34,77.26) | 107.63(48.03,157.95) | 59.87(40.15,86.91) | -2.46(-2.86,-2.06) |
| Turkmenistan | 329.10(283.40,378.70) | 221.63(182.44,270.25) | -32.66(-47.67,-14.12) | 21.93(18.88,25.23) | 14.54(11.97,17.73) | -1.83(-2.33,-1.33) |
| Republic of Uzbekistan | 4410.47(3057.57,5750.09) | 3605.36(2819.76,4619.85) | -18.25(-44.17,29.23) | 51.55(35.74,67.21) | 35.73(27.94,45.78) | -0.97(-1.24,-0.69) |
| Republic of Albania | 358.32(259.55,468.40) | 69.87(46.19,98.04) | -80.50(-87.24,-70.64) | 32.07(23.23,41.92) | 15.75(10.41,22.10) | -1.98(-2.71,-1.24) |
| Bosnia and Herzegovina | 235.81(182.51,297.22) | 68.66(45.88,92.28) | -70.88(-80.99,-58.88) | 21.52(16.66,27.13) | 14.00(9.36,18.82) | -1.08(-1.39,-0.77) |
| Republic of Bulgaria | 590.62(521.17,650.62) | 182.95(157.72,213.21) | -69.02(-74.08,-62.54) | 34.02(30.02,37.47) | 18.74(16.16,21.84) | -1.90(-2.36,-1.43) |
| Republic of Croatia | 295.36(243.32,352.74) | 101.18(76.53,130.10) | -65.74(-75.65,-51.70) | 29.92(24.65,35.74) | 16.94(12.82,21.79) | -1.28(-1.86,-0.70) |
| Czech Republic | 619.90(528.24,724.05) | 260.66(199.33,328.57) | -57.95(-69.21,-43.90) | 28.13(23.97,32.86) | 15.19(11.61,19.14) | -1.55(-1.89,-1.21) |
| Hungary | 656.37(552.85,775.63) | 189.48(155.11,235.24) | -71.13(-78.01,-61.47) | 30.80(25.94,36.40) | 13.65(11.17,16.94) | -1.79(-2.21,-1.38) |
| North Macedonia | 189.38(154.06,237.58) | 49.43(34.79,65.67) | -73.90(-82.83,-59.80) | 35.95(29.25,45.10) | 15.09(10.62,20.05) | -1.99(-2.55,-1.43) |
| Montenegro | 52.22(40.75,63.70) | 11.78(8.58,17.45) | -77.45(-84.52,-61.58) | 32.32(25.22,39.42) | 10.57(7.70,15.66) | -3.27(-3.88,-2.66) |
| Republic of Poland | 3130.63(2995.15,3260.82) | 780.43(691.07,857.82) | -75.07(-77.88,-72.36) | 32.69(31.27,34.05) | 13.26(11.74,14.57) | -2.89(-3.30,-2.48) |
| Romania | 2841.48(2501.56,3194.04) | 507.08(432.86,593.24) | -82.15(-85.19,-78.10) | 51.03(44.93,57.36) | 16.85(14.38,19.71) | -3.68(-4.02,-3.33) |
| Republic of Serbia | 975.19(648.29,1257.51) | 135.34(98.07,193.42) | -86.12(-90.79,-71.36) | 44.96(29.89,57.98) | 10.19(7.39,14.57) | -5.13(-5.64,-4.61) |
| Slovak Republic | 387.33(305.56,498.50) | 141.46(106.10,194.69) | -63.48(-73.84,-46.62) | 29.22(23.05,37.60) | 16.51(12.39,22.73) | -1.77(-2.02,-1.52) |
| Republic of Slovenia | 110.59(95.75,125.50) | 42.27(33.88,52.49) | -61.78(-71.11,-51.60) | 26.74(23.15,30.35) | 13.53(10.85,16.81) | -1.38(-1.83,-0.94) |
| Republic of Belarus | 346.06(288.60,420.66) | 322.85(258.21,416.33) | -6.71(-30.66,23.75) | 14.40(12.01,17.50) | 20.46(16.36,26.38) | 2.39(1.71,3.07) |
| Republic of Estonia | 103.71(83.94,127.53) | 72.01(54.94,94.60) | -30.56(-49.82,-6.69) | 29.71(24.04,36.53) | 33.32(25.42,43.77) | 0.15(-0.65,0.96) |
| Republic of Latvia | 99.43(85.02,114.73) | 41.19(33.42,50.42) | -58.58(-67.79,-46.89) | 17.48(14.94,20.17) | 13.87(11.25,16.97) | 0.99(0.26,1.73) |
| Republic of Lithuania | 126.29(103.16,152.69) | 80.86(63.66,103.90) | -35.97(-52.51,-14.33) | 15.20(12.42,18.38) | 19.83(15.61,25.48) | 0.50(-0.11,1.11) |
| Republic of Moldova | 1003.49(857.66,1163.26) | 147.83(122.39,179.49) | -85.27(-88.42,-81.14) | 81.19(69.39,94.12) | 28.30(23.43,34.36) | -2.33(-2.85,-1.81) |
| Russian Federation | 20582.13(19888.68,21253.38) | 3692.15(3429.06,3967.13) | -82.06(-83.51,-80.77) | 59.32(57.32,61.25) | 14.16(13.15,15.21) | -4.82(-5.30,-4.35) |
| Ukraine | 3479.05(2426.27,4306.37) | 1391.58(1155.07,1688.29) | -60.00(-70.36,-36.59) | 30.59(21.33,37.86) | 21.93(18.20,26.61) | -1.26(-1.67,-0.85) |
| Brunei Darussalam | 44.46(32.67,59.44) | 21.56(16.09,27.35) | -51.51(-66.48,-25.30) | 49.09(36.07,65.62) | 22.79(17.01,28.91) | -1.34(-1.83,-0.85) |
| Japan | 5767.83(5597.63,5916.43) | 1865.83(1754.76,1967.13) | -67.65(-69.85,-65.91) | 24.98(24.24,25.62) | 12.08(11.36,12.74) | -2.50(-2.73,-2.27) |
| Republic of Korea | 3951.76(3120.54,5198.01) | 543.98(395.64,733.91) | -86.23(-90.88,-78.46) | 34.75(27.44,45.71) | 8.96(6.51,12.08) | -4.52(-4.77,-4.27) |
| Republic of Singapore | 188.16(159.81,225.73) | 96.00(79.23,115.32) | -48.98(-59.86,-34.63) | 28.98(24.61,34.76) | 11.82(9.76,14.20) | -2.36(-2.70,-2.02) |
| Australia | 848.31(747.27,966.56) | 327.52(262.80,392.74) | -61.39(-70.38,-51.80) | 22.41(19.74,25.53) | 6.90(5.53,8.27) | -3.59(-4.09,-3.08) |
| New Zealand | 124.11(107.58,141.76) | 82.45(69.94,100.10) | -33.57(-46.07,-16.72) | 15.51(13.45,17.72) | 8.40(7.12,10.20) | -2.57(-3.58,-1.55) |
| Principality of Andorra | 5.49(3.50,7.74) | 1.56(1.10,2.18) | -71.58(-81.77,-53.21) | 57.76(36.86,81.49) | 15.34(10.78,21.40) | -3.93(-4.18,-3.68) |
| Republic of Austria | 247.62(226.11,270.40) | 115.16(100.53,132.74) | -53.49(-59.58,-46.05) | 18.36(16.77,20.05) | 8.88(7.75,10.23) | -1.98(-2.25,-1.71) |
| Kingdom of Belgium | 351.64(304.34,402.51) | 253.43(203.91,314.59) | -27.93(-44.82,-7.74) | 19.47(16.85,22.29) | 13.25(10.66,16.45) | -1.98(-2.25,-1.72) |
| Republic of Cyprus | 45.36(33.38,58.32) | 19.27(13.90,26.11) | -57.53(-70.83,-34.69) | 22.92(16.86,29.46) | 8.81(6.35,11.94) | -2.37(-3.27,-1.45) |
| Kingdom of Denmark | 167.55(145.95,193.98) | 76.36(61.78,94.16) | -54.42(-64.85,-41.13) | 18.97(16.52,21.96) | 8.00(6.47,9.87) | -3.18(-3.43,-2.92) |
| Republic of Finland | 197.03(164.90,232.92) | 110.13(85.82,137.95) | -44.11(-58.02,-26.47) | 20.42(17.09,24.14) | 13.00(10.13,16.29) | -2.19(-2.61,-1.77) |
| French Republic | 2504.11(2229.07,2777.52) | 1373.76(1156.73,1635.58) | -45.14(-55.01,-33.99) | 21.38(19.03,23.71) | 11.83(9.97,14.09) | -2.29(-2.50,-2.08) |
| Federal Republic of Germany | 2454.28(2195.26,2757.54) | 1138.30(980.33,1335.45) | -53.62(-60.88,-44.81) | 18.96(16.96,21.30) | 9.51(8.19,11.16) | -2.04(-2.27,-1.80) |
| Hellenic Republic | 185.84(167.80,204.23) | 77.73(66.92,89.72) | -58.17(-64.98,-49.82) | 9.18(8.29,10.09) | 5.57(4.80,6.43) | -1.28(-1.63,-0.93) |
| Republic of Iceland | 13.41(11.26,15.55) | 8.49(6.41,10.95) | -36.67(-53.20,-13.10) | 21.14(17.74,24.51) | 12.58(9.49,16.21) | -1.20(-1.92,-0.48) |
| Ireland | 207.87(174.24,242.67) | 85.33(66.53,107.67) | -58.95(-69.92,-44.39) | 21.16(17.73,24.70) | 8.56(6.67,10.80) | -2.82(-3.21,-2.43) |
| State of Israel | 484.33(429.68,547.62) | 372.59(307.78,455.50) | -23.07(-38.46,-3.59) | 31.59(28.03,35.72) | 14.18(11.71,17.33) | -2.38(-2.80,-1.97) |
| Republic of Italy | 2960.86(2773.64,3152.95) | 1000.16(882.12,1146.00) | -66.22(-70.49,-61.62) | 32.08(30.05,34.16) | 13.16(11.61,15.08) | -3.34(-3.59,-3.09) |
| Grand Duchy of Luxembourg | 13.43(12.03,15.02) | 9.47(7.32,11.63) | -29.48(-45.52,-12.14) | 20.33(18.20,22.73) | 9.36(7.23,11.49) | -3.91(-4.41,-3.40) |
| Republic of Malta | 16.89(12.00,20.25) | 21.15(15.39,29.20) | 25.25(-10.52,80.47) | 19.30(13.71,23.14) | 33.04(24.05,45.61) | -0.30(-1.09,0.50) |
| Kingdom of the Netherlands | 597.97(525.11,676.54) | 281.60(231.89,331.63) | -52.91(-62.57,-42.42) | 21.94(19.27,24.82) | 10.50(8.65,12.36) | -2.36(-2.63,-2.09) |
| Kingdom of Norway | 209.51(193.76,227.44) | 64.26(55.83,74.38) | -69.33(-73.77,-64.21) | 26.24(24.27,28.49) | 6.96(6.04,8.05) | -3.84(-4.29,-3.39) |
| Portuguese Republic | 774.40(666.15,890.38) | 173.57(139.73,209.05) | -77.59(-82.63,-71.32) | 36.60(31.48,42.08) | 12.74(10.26,15.35) | -4.34(-4.75,-3.94) |
| Kingdom of Spain | 2113.80(1805.40,2433.71) | 739.16(600.80,928.13) | -65.03(-72.84,-54.85) | 26.98(23.04,31.06) | 11.41(9.27,14.32) | -3.22(-3.44,-3.00) |
| Kingdom of Sweden | 238.33(211.61,270.44) | 117.68(99.92,138.90) | -50.62(-60.20,-39.32) | 15.43(13.70,17.51) | 6.46(5.49,7.63) | -1.86(-2.53,-1.19) |
| Swiss Confederation | 275.51(240.89,317.25) | 128.27(105.77,155.90) | -53.44(-62.79,-41.78) | 23.84(20.84,27.45) | 9.63(7.94,11.70) | -3.31(-3.66,-2.96) |
| United Kingdom of Great Britain and Northern Ireland | 1846.41(1777.99,1923.32) | 938.85(859.66,1022.16) | -49.15(-53.10,-45.05) | 16.91(16.28,17.61) | 7.97(7.30,8.67) | -2.37(-2.67,-2.07) |
| Argentine Republic | 3644.19(3208.26,4144.74) | 1915.06(1572.28,2302.72) | -47.45(-58.77,-33.44) | 35.96(31.65,40.89) | 18.81(15.44,22.61) | -1.72(-2.02,-1.41) |
| Republic of Chile | 1150.74(1023.25,1292.83) | 407.24(343.22,485.22) | -64.61(-71.40,-56.73) | 28.97(25.76,32.55) | 11.15(9.40,13.29) | -2.90(-3.21,-2.59) |
| Eastern Republic of Uruguay | 285.76(246.57,331.40) | 130.48(105.69,162.60) | -54.34(-64.97,-40.73) | 34.91(30.12,40.48) | 19.78(16.03,24.65) | -2.23(-2.76,-1.69) |
| Canada | 1284.12(1129.46,1449.59) | 385.34(316.03,466.71) | -69.99(-76.69,-62.23) | 22.33(19.64,25.20) | 6.24(5.12,7.56) | -4.86(-5.29,-4.43) |
| United States of America | 9141.93(8881.89,9465.38) | 3856.03(3551.86,4157.01) | -57.82(-60.89,-54.85) | 16.35(15.89,16.93) | 6.49(5.98,6.99) | -3.05(-3.21,-2.90) |
| Antigua and Barbuda | 5.86(4.87,6.92) | 4.53(3.99,5.20) | -22.68(-36.61,-4.34) | 32.19(26.76,38.07) | 26.79(23.58,30.75) | -0.53(-1.04,-0.01) |
| Commonwealth of the Bahamas | 36.20(30.78,42.15) | 22.51(17.95,28.30) | -37.82(-52.37,-18.29) | 44.88(38.16,52.25) | 27.73(22.11,34.86) | -1.81(-2.23,-1.40) |
| Barbados | 47.64(41.46,54.61) | 17.75(13.06,23.36) | -62.74(-72.33,-49.93) | 76.39(66.49,87.57) | 37.70(27.73,49.60) | -1.15(-1.65,-0.65) |
| Belize | 36.66(32.41,40.80) | 18.92(16.06,22.34) | -48.41(-57.71,-37.09) | 44.78(39.59,49.84) | 15.36(13.04,18.15) | -2.76(-3.06,-2.45) |
| Republic of Cuba | 1458.87(1303.12,1633.56) | 465.15(372.92,570.19) | -68.12(-74.83,-59.68) | 58.26(52.04,65.24) | 26.18(20.99,32.09) | -2.04(-2.39,-1.68) |
| Commonwealth of Dominica | 15.93(12.39,20.34) | 13.87(10.06,18.80) | -12.94(-42.90,19.96) | 64.19(49.94,81.98) | 101.35(73.50,137.37) | 1.80(1.55,2.05) |
| Dominican Republic | 1655.76(1206.33,2088.65) | 810.84(539.44,1164.29) | -51.03(-67.95,-26.23) | 61.43(44.75,77.49) | 27.59(18.36,39.62) | -2.70(-3.01,-2.39) |
| Grenada | 40.74(32.71,50.50) | 17.83(14.43,21.96) | -56.24(-67.29,-41.98) | 121.96(97.92,151.16) | 81.68(66.09,100.60) | -0.69(-1.01,-0.38) |
| Republic of Guyana | 120.43(96.65,144.81) | 67.43(51.53,86.97) | -44.01(-59.51,-24.16) | 40.97(32.88,49.27) | 31.60(24.15,40.76) | 0.75(0.23,1.27) |
| Republic of Haiti | 6531.48(1780.38,10536.87) | 5527.03(2347.52,8912.17) | -15.38(-45.08,38.80) | 240.74(65.62,388.37) | 126.98(53.93,204.75) | -1.60(-1.88,-1.32) |
| Jamaica | 781.59(656.93,908.44) | 254.82(199.79,322.32) | -67.40(-75.45,-58.13) | 93.58(78.65,108.77) | 43.64(34.21,55.20) | -2.36(-2.70,-2.01) |
| Saint Lucia | 37.28(31.46,44.43) | 15.62(12.43,19.54) | -58.10(-68.88,-44.81) | 72.33(61.04,86.21) | 52.62(41.85,65.80) | -1.40(-1.64,-1.15) |
| Saint Vincent and the Grenadines | 44.73(37.64,52.64) | 18.57(15.65,22.17) | -58.49(-66.89,-48.13) | 108.87(91.62,128.13) | 74.41(62.72,88.85) | -0.99(-1.36,-0.62) |
| Republic of Suriname | 81.46(48.62,105.37) | 60.43(42.20,81.85) | -25.82(-54.42,33.32) | 62.53(37.32,80.89) | 42.18(29.46,57.13) | -1.00(-1.33,-0.67) |
| Republic of Trinidad and Tobago | 276.41(241.26,315.47) | 89.70(70.97,114.39) | -67.55(-74.96,-57.29) | 68.03(59.38,77.64) | 32.93(26.05,41.99) | -1.88(-2.28,-1.47) |
| Plurinational State of Bolivia | 2857.42(2014.14,3851.02) | 1588.99(1167.02,2141.13) | -44.39(-64.81,-7.95) | 106.39(74.99,143.38) | 45.58(33.47,61.41) | -2.82(-2.93,-2.71) |
| Republic of Ecuador | 1508.31(1338.42,1717.51) | 1231.54(985.68,1533.06) | -18.35(-36.30,2.98) | 39.02(34.63,44.43) | 24.29(19.44,30.23) | -1.42(-2.00,-0.85) |
| Republic of Peru | 5666.18(4616.09,7468.33) | 3037.16(2213.53,4104.63) | -46.40(-62.91,-22.03) | 68.26(55.61,89.97) | 31.84(23.21,43.04) | -2.20(-2.40,-2.01) |
| Republic of Colombia | 6574.80(5652.43,7516.53) | 2519.46(1997.88,3159.98) | -61.68(-70.65,-51.04) | 56.37(48.46,64.45) | 23.74(18.82,29.77) | -1.83(-2.22,-1.44) |
| Republic of Costa Rica | 432.61(380.23,501.13) | 271.14(218.81,340.29) | -37.33(-51.47,-17.78) | 38.48(33.82,44.58) | 26.65(21.51,33.45) | -1.19(-1.37,-1.01) |
| Republic of El Salvador | 694.17(546.75,871.82) | 296.22(226.71,384.92) | -57.33(-68.86,-40.16) | 32.17(25.34,40.40) | 16.29(12.46,21.16) | -1.95(-2.19,-1.71) |
| Republic of Guatemala | 1416.16(1284.24,1565.40) | 655.70(538.72,813.70) | -53.70(-62.77,-42.02) | 34.87(31.62,38.55) | 13.29(10.92,16.49) | -2.21(-2.52,-1.91) |
| Republic of Honduras | 575.91(436.71,743.05) | 268.74(143.32,455.47) | -53.34(-74.44,-18.74) | 26.07(19.77,33.63) | 8.20(4.37,13.90) | -3.87(-4.13,-3.62) |
| United Mexican States | 14495.09(13181.16,16051.54) | 5564.45(4752.36,6515.45) | -61.61(-68.36,-53.91) | 43.38(39.45,48.04) | 17.35(14.82,20.32) | -2.44(-2.76,-2.13) |
| Republic of Nicaragua | 789.42(595.74,1017.35) | 244.53(183.47,326.59) | -69.02(-78.58,-56.02) | 43.35(32.71,55.86) | 12.35(9.26,16.49) | -3.14(-3.43,-2.86) |
| Republic of Panama | 334.57(288.46,388.71) | 376.24(307.95,452.47) | 12.46(-11.60,41.73) | 40.12(34.59,46.61) | 32.62(26.70,39.23) | -0.62(-0.82,-0.42) |
| Bolivarian Republic of Venezuela | 3040.30(2837.81,3261.55) | 2214.80(1731.25,2771.11) | -27.15(-42.99,-8.10) | 42.86(40.00,45.98) | 33.44(26.14,41.83) | -0.75(-1.22,-0.28) |
| Federative Republic of Brazil | 24227.17(21559.59,26907.74) | 7954.86(6438.17,9378.74) | -67.17(-72.99,-60.63) | 46.64(41.51,51.80) | 16.51(13.36,19.46) | -2.78(-3.25,-2.30) |
| Republic of Paraguay | 685.38(516.09,894.41) | 545.66(386.30,773.07) | -20.39(-48.08,20.24) | 41.05(30.91,53.57) | 27.18(19.24,38.50) | -0.98(-1.40,-0.55) |
| People's Democratic Republic of Algeria | 6750.50(4855.81,8774.61) | 3683.90(2684.48,4994.12) | -45.43(-62.44,-22.40) | 62.94(45.28,81.82) | 27.69(20.18,37.54) | -2.43(-2.56,-2.30) |
| Kingdom of Bahrain | 40.96(30.80,52.60) | 36.66(26.62,49.85) | -10.48(-39.18,28.97) | 25.09(18.87,32.22) | 12.36(8.97,16.80) | -2.33(-2.72,-1.95) |
| Arab Republic of Egypt | 8055.13(6033.22,13575.16) | 3580.80(2221.73,8029.06) | -55.55(-71.33,-28.41) | 36.31(27.20,61.20) | 9.72(6.03,21.79) | -3.97(-4.56,-3.37) |
| Islamic Republic of Iran | 7862.73(6115.66,10181.00) | 3442.06(2687.25,4522.35) | -56.22(-70.34,-35.40) | 30.97(24.09,40.11) | 17.06(13.32,22.41) | -1.24(-1.77,-0.70) |
| Republic of Iraq | 5749.50(3367.06,8154.29) | 3474.17(2408.15,4878.76) | -39.57(-63.59,15.19) | 69.81(40.88,99.00) | 25.81(17.89,36.24) | -2.74(-3.08,-2.39) |
| Hashemite Kingdom of Jordan | 888.52(659.37,1199.41) | 1331.43(985.99,1800.83) | 49.85(-2.96,122.85) | 54.40(40.37,73.43) | 36.65(27.14,49.57) | -1.60(-1.98,-1.22) |
| State of Kuwait | 350.08(301.13,410.07) | 172.39(132.34,213.75) | -50.76(-62.24,-36.25) | 63.15(54.32,73.97) | 20.39(15.65,25.28) | -1.73(-2.67,-0.78) |
| Lebanese Republic | 541.64(374.37,732.47) | 316.08(221.84,445.30) | -41.64(-62.55,-3.42) | 51.79(35.79,70.03) | 24.73(17.36,34.84) | -2.49(-2.83,-2.15) |
| State of Libya | 1134.29(761.33,1503.12) | 1081.52(755.02,1480.62) | -4.65(-37.84,52.84) | 62.64(42.04,83.00) | 72.51(50.62,99.26) | 0.99(0.69,1.29) |
| Kingdom of Morocco | 4631.99(2952.67,6525.31) | 1944.13(1328.18,2864.73) | -58.03(-74.67,-23.15) | 47.33(30.17,66.68) | 19.85(13.56,29.26) | -2.37(-2.59,-2.15) |
| Palestine | 282.10(198.15,386.94) | 323.41(241.30,435.21) | 14.65(-27.05,86.78) | 29.13(20.46,39.96) | 17.32(12.92,23.31) | -0.94(-1.38,-0.50) |
| Sultanate of Oman | 306.09(197.74,440.73) | 224.76(164.18,289.22) | -26.57(-55.32,15.94) | 36.42(23.53,52.44) | 18.38(13.42,23.65) | -1.05(-1.50,-0.60) |
| State of Qatar | 19.13(13.62,27.48) | 42.91(29.95,61.49) | 124.28(41.36,279.66) | 15.30(10.89,21.98) | 8.69(6.06,12.45) | -1.09(-1.58,-0.60) |
| Kingdom of Saudi Arabia | 2636.95(1503.18,3814.71) | 948.29(566.53,1366.47) | -64.04(-79.14,-38.00) | 40.24(22.94,58.21) | 12.53(7.49,18.06) | -3.74(-3.84,-3.64) |
| Syrian Arab Republic | 1941.21(1416.61,2482.01) | 638.67(482.68,866.33) | -67.10(-77.76,-50.25) | 32.78(23.92,41.91) | 17.43(13.18,23.65) | -1.95(-2.58,-1.31) |
| Republic of Tunisia | 2394.35(1675.28,3118.31) | 825.25(586.92,1121.16) | -65.53(-78.63,-44.49) | 77.10(53.95,100.41) | 29.84(21.22,40.54) | -2.72(-2.83,-2.60) |
| Republic of Turkey | 12836.74(7782.56,17444.71) | 3999.77(3004.57,5283.18) | -68.84(-79.21,-47.60) | 62.65(37.98,85.14) | 21.60(16.22,28.53) | -3.37(-3.66,-3.08) |
| United Arab Emirates | 139.85(88.63,213.46) | 120.13(88.32,165.89) | -14.10(-48.53,51.75) | 23.73(15.04,36.22) | 8.97(6.60,12.39) | -2.70(-3.05,-2.35) |
| Republic of Yemen | 2768.05(1552.00,5258.54) | 2895.43(1919.89,4562.57) | 4.60(-33.17,105.20) | 39.02(21.88,74.12) | 21.00(13.92,33.09) | -1.88(-2.01,-1.74) |
| Islamic Republic of Afghanistan | 2472.06(864.07,4143.00) | 4246.14(2684.38,6970.21) | 71.77(13.28,284.64) | 57.38(20.06,96.17) | 29.90(18.90,49.08) | -1.76(-2.03,-1.48) |
| People's Republic of Bangladesh | 47093.96(20169.39,70118.11) | 17444.32(11465.90,28988.02) | -62.96(-75.88,-24.52) | 96.28(41.24,143.36) | 38.12(25.05,63.34) | -2.77(-2.91,-2.63) |
| Kingdom of Bhutan | 198.03(88.87,315.79) | 69.41(40.10,131.05) | -64.95(-84.24,26.68) | 75.53(33.90,120.44) | 37.08(21.42,70.02) | -2.73(-3.04,-2.42) |
| Republic of India | 166972.41(99052.82,224263.48) | 67096.50(52313.80,86213.80) | -59.82(-70.59,-21.39) | 51.14(30.34,68.68) | 18.31(14.28,23.53) | -3.32(-3.55,-3.09) |
| Federal Democratic Republic of Nepal | 6187.69(2774.03,9364.96) | 2448.25(1550.67,4202.57) | -60.43(-74.96,-10.57) | 73.44(32.93,111.15) | 26.53(16.81,45.55) | -2.95(-3.19,-2.71) |
| Islamic Republic of Pakistan | 47845.44(34279.68,63555.81) | 91655.86(62965.53,130514.42) | 91.57(29.06,208.82) | 97.16(69.61,129.07) | 107.27(73.69,152.75) | 0.78(0.60,0.96) |
| Republic of Angola | 4703.44(995.13,7831.36) | 5021.65(3071.08,7472.91) | 6.77(-33.25,297.87) | 99.76(21.11,166.10) | 32.94(20.14,49.02) | -3.37(-3.69,-3.05) |
| Central African Republic | 910.99(257.89,1513.29) | 1139.34(568.37,1785.06) | 25.07(-19.59,135.49) | 74.51(21.09,123.77) | 49.89(24.89,78.16) | -1.02(-1.18,-0.87) |
| Republic of the Congo | 575.55(243.29,881.13) | 549.66(388.71,744.20) | -4.50(-36.80,96.90) | 54.66(23.11,83.68) | 28.49(20.15,38.57) | -2.03(-2.25,-1.81) |
| Democratic Republic of the Congo | 11068.64(3635.49,16225.08) | 9585.11(6096.10,13840.43) | -13.40(-43.09,115.79) | 62.52(20.54,91.65) | 25.22(16.04,36.42) | -2.44(-2.67,-2.22) |
| Republic of Equatorial Guinea | 129.49(43.24,203.71) | 171.88(84.53,328.90) | 32.73(-37.65,255.52) | 65.76(21.96,103.45) | 29.38(14.45,56.22) | -3.29(-3.63,-2.94) |
| Gabonese Republic | 172.41(103.98,247.98) | 210.33(135.26,312.39) | 21.99(-25.63,104.53) | 42.31(25.52,60.85) | 32.91(21.16,48.88) | -0.23(-0.46,0.00) |
| Republic of Burundi | 5781.21(3564.11,8940.42) | 4582.71(2489.41,8465.05) | -20.73(-50.34,67.82) | 220.56(135.98,341.09) | 78.28(42.52,144.60) | -2.80(-3.04,-2.55) |
| Union of the Comoros | 289.97(154.96,417.57) | 228.93(150.02,344.38) | -21.05(-52.83,49.77) | 136.33(72.86,196.33) | 95.33(62.47,143.40) | -1.25(-1.62,-0.88) |
| Republic of Djibouti | 215.94(142.05,311.47) | 320.57(190.79,537.97) | 48.45(-8.03,131.96) | 124.02(81.58,178.89) | 77.59(46.18,130.21) | -1.41(-1.80,-1.01) |
| State of Eritrea | 2437.88(1597.04,3357.97) | 2428.78(1453.44,4165.78) | -0.37(-38.76,95.76) | 153.14(100.32,210.94) | 96.20(57.57,165.01) | -1.43(-1.60,-1.26) |
| Federal Democratic Republic of Ethiopia | 52822.17(13975.63,82042.95) | 37694.23(26300.20,52216.50) | -28.64(-50.55,131.75) | 216.81(57.36,336.74) | 84.99(59.30,117.74) | -3.35(-3.63,-3.07) |
| Republic of Kenya | 5422.47(3565.74,7784.03) | 5936.81(4454.86,7555.36) | 9.49(-22.86,65.37) | 48.54(31.92,69.69) | 31.81(23.87,40.48) | -0.21(-0.72,0.31) |
| Republic of Madagascar | 7696.33(5491.06,10148.43) | 8173.41(5533.49,11700.36) | 6.20(-28.09,73.99) | 141.06(100.64,186.01) | 69.66(47.16,99.72) | -1.81(-2.02,-1.60) |
| Republic of Malawi | 24424.20(17655.44,32189.06) | 22003.69(10995.54,46258.79) | -9.91(-53.16,76.27) | 536.84(388.06,707.51) | 270.85(135.35,569.42) | -1.85(-2.13,-1.57) |
| Republic of Mauritius | 22.12(20.22,24.16) | 23.65(20.71,26.65) | 6.94(-9.34,24.99) | 6.70(6.13,7.32) | 11.40(9.98,12.85) | 0.54(-0.09,1.18) |
| Republic of Mozambique | 1858.35(1013.16,4605.30) | 1728.77(846.79,4160.29) | -6.97(-47.79,134.17) | 29.95(16.33,74.23) | 12.12(5.94,29.16) | -2.45(-2.76,-2.14) |
| Republic of Rwanda | 8291.23(5564.22,11575.42) | 4510.72(3066.44,7506.06) | -45.60(-65.74,-0.65) | 244.37(164.00,341.17) | 90.75(61.69,151.01) | -3.75(-4.06,-3.44) |
| Republic of Seychelles | 4.18(3.12,5.96) | 2.53(1.83,3.39) | -39.41(-60.57,-9.14) | 17.63(13.13,25.12) | 10.83(7.80,14.48) | 0.10(-0.85,1.06) |
| Federal Republic of Somalia | 5492.87(2968.85,8107.47) | 7540.10(3800.76,12100.48) | 37.27(-13.91,133.65) | 141.00(76.21,208.12) | 73.00(36.80,117.15) | -1.68(-2.06,-1.29) |
| United Republic of Tanzania | 23027.97(15776.43,30613.37) | 28171.53(18569.50,43830.24) | 22.34(-21.10,91.92) | 190.70(130.65,253.52) | 115.44(76.10,179.61) | -1.05(-1.33,-0.78) |
| Republic of Uganda | 18046.19(12372.25,25140.95) | 37563.42(22580.24,61705.64) | 108.15(32.80,223.82) | 214.33(146.95,298.60) | 189.37(113.84,311.08) | -0.22(-0.47,0.02) |
| Republic of Zambia | 7829.53(5401.74,10387.42) | 8338.29(5001.90,13967.83) | 6.50(-34.94,117.04) | 208.54(143.87,276.66) | 100.81(60.47,168.87) | -2.18(-2.34,-2.03) |
| Republic of Botswana | 147.65(98.48,221.23) | 256.17(164.63,380.38) | 73.51(11.86,166.10) | 25.01(16.68,37.47) | 36.69(23.58,54.47) | 2.00(1.60,2.41) |
| Kingdom of Lesotho | 152.27(101.73,230.65) | 248.15(157.42,359.68) | 62.96(5.86,156.70) | 22.31(14.90,33.79) | 39.36(24.97,57.05) | 2.84(2.46,3.22) |
| Republic of Namibia | 277.09(175.68,400.43) | 471.06(284.40,717.00) | 70.00(4.94,204.38) | 46.12(29.24,66.66) | 57.07(34.45,86.86) | 1.69(1.10,2.30) |
| Republic of South Africa | 3715.33(2503.52,5576.92) | 4022.72(3285.10,5016.58) | 8.27(-19.01,48.86) | 27.29(18.39,40.97) | 26.46(21.61,32.99) | -0.31(-0.68,0.05) |
| Kingdom of Eswatini | 154.77(88.23,238.62) | 178.92(113.31,277.81) | 15.60(-21.48,83.16) | 40.12(22.87,61.86) | 43.36(27.46,67.33) | 0.61(0.32,0.90) |
| Republic of Zimbabwe | 1871.94(904.54,2716.50) | 5650.93(2567.75,8184.76) | 201.88(89.87,354.99) | 38.87(18.78,56.40) | 89.79(40.80,130.04) | 5.22(4.03,6.42) |
| Republic of Benin | 2749.21(1791.93,4022.79) | 4997.59(3141.59,7172.44) | 81.78(8.13,205.82) | 113.52(73.99,166.11) | 82.19(51.67,117.96) | -0.80(-0.95,-0.66) |
| Burkina Faso | 5301.70(3430.75,7634.18) | 8898.03(6146.28,12335.88) | 67.83(11.06,161.75) | 112.35(72.70,161.77) | 85.79(59.26,118.93) | -0.51(-0.71,-0.31) |
| Republic of Cameroon | 5222.59(3455.27,7200.70) | 12028.32(8023.55,16699.25) | 130.31(50.64,257.16) | 106.98(70.78,147.49) | 89.32(59.58,124.00) | -0.19(-0.40,0.02) |
| Republic of Cabo Verde | 25.82(14.98,43.09) | 81.86(34.47,115.40) | 217.02(-7.06,531.37) | 16.41(9.52,27.39) | 57.16(24.07,80.59) | 2.74(1.77,3.71) |
| Republic of Chad | 2675.95(1636.23,4050.82) | 7606.41(4744.22,10985.02) | 184.25(78.66,365.24) | 91.44(55.91,138.42) | 84.38(52.63,121.85) | 0.10(-0.04,0.23) |
| Republic of Côte d'Ivoire | 6415.92(4529.66,8901.27) | 10995.49(7375.21,18253.39) | 71.38(11.75,167.40) | 112.49(79.42,156.07) | 95.02(63.73,157.74) | -0.20(-0.43,0.03) |
| Republic of the Gambia | 564.52(377.82,780.77) | 742.73(483.60,1254.18) | 31.57(-17.04,107.06) | 122.38(81.91,169.27) | 74.76(48.68,126.25) | -1.75(-2.04,-1.46) |
| Republic of Ghana | 10676.87(5011.37,16303.32) | 5146.53(3332.52,8602.00) | -51.80(-73.69,26.43) | 158.96(74.61,242.73) | 39.95(25.87,66.77) | -5.74(-6.68,-4.80) |
| Republic of Guinea | 4154.44(2770.17,5784.85) | 4219.68(2606.69,8401.62) | 1.57(-36.86,93.20) | 150.97(100.67,210.22) | 69.80(43.12,138.97) | -1.91(-2.09,-1.74) |
| Republic of Guinea-Bissau | 692.32(431.94,1026.39) | 578.15(357.19,908.82) | -16.49(-45.49,54.94) | 143.52(89.54,212.77) | 64.37(39.77,101.19) | -2.31(-2.65,-1.96) |
| Republic of Liberia | 1779.84(992.79,2457.02) | 1579.24(1032.43,2242.50) | -11.27(-46.26,62.94) | 157.48(87.84,217.40) | 72.25(47.23,102.59) | -2.37(-3.08,-1.66) |
| Republic of Mali | 4047.14(2673.62,5933.12) | 5065.90(3025.94,9389.20) | 25.17(-21.16,127.69) | 98.00(64.74,143.67) | 43.76(26.14,81.11) | -2.27(-2.45,-2.09) |
| Islamic Republic of Mauritania | 626.48(419.31,890.52) | 856.72(557.23,1203.04) | 36.75(-14.11,115.29) | 67.77(45.36,96.34) | 46.23(30.07,64.92) | -1.39(-1.68,-1.11) |
| Republic of the Niger | 6575.93(3684.75,9659.84) | 7587.74(4450.54,11945.73) | 15.39(-29.89,122.58) | 161.85(90.69,237.75) | 59.45(34.87,93.59) | -3.25(-3.50,-2.99) |
| Federal Republic of Nigeria | 61011.32(38081.15,85319.48) | 109622.16(61684.54,157384.74) | 79.68(26.41,229.84) | 155.94(97.33,218.07) | 107.91(60.72,154.92) | -0.92(-1.05,-0.79) |
| Democratic Republic of Sao Tome and Principe | 63.30(44.80,89.91) | 28.20(16.44,46.60) | -55.45(-74.60,-12.44) | 111.70(79.06,158.66) | 36.23(21.12,59.87) | -3.15(-3.57,-2.72) |
| Republic of Senegal | 4082.49(2779.11,5711.87) | 3257.92(2214.89,4903.50) | -20.20(-48.18,24.10) | 111.81(76.11,156.43) | 51.22(34.82,77.09) | -2.46(-2.70,-2.21) |
| Republic of Sierra Leone | 2670.93(1593.26,3797.56) | 2788.55(1925.33,4078.35) | 4.40(-34.01,96.32) | 147.35(87.90,209.51) | 77.98(53.84,114.04) | -1.98(-2.14,-1.81) |
| Togolese Republic | 1518.44(1056.91,2163.82) | 1864.58(1228.44,2851.68) | 22.80(-21.38,109.32) | 86.15(59.97,122.77) | 56.35(37.12,86.18) | -1.23(-1.37,-1.08) |
| American Samoa | 2.36(1.67,3.40) | 2.89(1.81,4.53) | 22.50(-29.50,113.50) | 12.41(8.76,17.89) | 20.38(12.78,31.95) | 2.18(1.92,2.44) |
| Bermuda | 5.91(4.89,7.16) | 2.24(1.78,2.74) | -62.03(-71.87,-49.46) | 49.56(40.98,60.07) | 26.59(21.14,32.43) | -1.58(-1.96,-1.20) |
| Cook Islands | 0.69(0.45,0.98) | 0.44(0.23,0.84) | -37.05(-71.52,44.89) | 10.50(6.75,14.80) | 11.51(6.10,22.07) | -1.27(-1.88,-0.66) |
| Greenland | 7.23(3.31,10.33) | 1.68(1.11,2.40) | -76.76(-85.78,-59.77) | 50.83(23.28,72.60) | 14.29(9.47,20.39) | -3.84(-4.35,-3.33) |
| Guam | 9.91(8.09,12.25) | 6.52(4.66,8.93) | -34.21(-53.54,-8.87) | 23.76(19.39,29.37) | 17.82(12.75,24.42) | 1.64(0.73,2.56) |
| Principality of Monaco | 0.75(0.50,1.12) | 0.91(0.66,1.29) | 21.72(-16.70,98.80) | 21.27(14.11,31.69) | 18.33(13.24,25.89) | -1.83(-2.35,-1.31) |
| Republic of Nauru | 1.13(0.76,1.59) | 1.36(0.84,2.01) | 20.07(-27.36,100.04) | 26.78(18.05,37.63) | 34.12(21.14,50.58) | 0.70(0.22,1.18) |
| Republic of Niue | 0.16(0.11,0.23) | 0.53(0.36,0.79) | 231.93(109.65,448.73) | 19.71(13.20,28.50) | 136.33(92.68,203.11) | 2.69(1.40,3.99) |
| Northern Mariana Islands | 0.73(0.44,1.43) | 1.10(0.72,1.70) | 50.16(-21.17,185.10) | 6.02(3.63,11.74) | 9.75(6.39,15.12) | 2.75(1.96,3.54) |
| Republic of Palau | 0.45(0.30,0.62) | 0.23(0.16,0.36) | -47.91(-65.66,-16.55) | 9.86(6.63,13.60) | 7.18(4.86,11.13) | -0.84(-1.03,-0.66) |
| Puerto Rico | 441.87(388.22,502.14) | 55.91(46.20,66.88) | -87.35(-90.02,-83.87) | 44.37(38.99,50.43) | 12.58(10.40,15.05) | -3.50(-3.76,-3.24) |
| Saint Kitts and Nevis | 9.14(8.09,10.29) | 4.25(3.59,5.00) | -53.57(-61.87,-43.04) | 64.75(57.29,72.84) | 43.11(36.49,50.77) | -1.01(-1.22,-0.81) |
| Republic of San Marino | 2.65(1.92,3.55) | 0.94(0.67,1.35) | -64.38(-76.39,-40.64) | 64.70(46.79,86.65) | 21.48(15.13,30.70) | -3.25(-3.36,-3.14) |
| Tokelau | 0.12(0.08,0.18) | 0.46(0.29,0.73) | 279.25(116.96,583.46) | 20.07(12.50,30.33) | 117.06(73.58,186.64) | 1.54(-0.17,3.28) |
| Tuvalu | 0.95(0.53,1.43) | 0.62(0.43,0.89) | -34.99(-61.32,23.10) | 27.35(15.15,41.05) | 16.58(11.54,23.97) | -1.27(-1.43,-1.10) |
| United States Virgin Islands | 16.47(12.33,22.10) | 1.78(1.11,2.77) | -89.19(-93.87,-82.04) | 51.55(38.61,69.19) | 13.29(8.29,20.71) | -3.36(-3.82,-2.89) |
| Republic of South Sudan | 4720.59(2741.01,6758.07) | 6951.17(4282.50,9855.37) | 47.25(1.94,119.63) | 179.89(104.45,257.53) | 161.85(99.71,229.46) | 0.11(-0.61,0.83) |
| Republic of Sudan | 7885.98(3094.74,15744.96) | 6185.94(4060.84,8763.18) | -21.56(-61.37,77.58) | 88.68(34.80,177.06) | 37.29(24.48,52.82) | -2.48(-2.59,-2.36) |
